# Supplementary figures and images for: Auditory fear memory retrieval requires BLA-LS and LS-VMH circuitries via GABAergic and dopaminergic neurons (part 1 of 2)
Source: EMBO Rep. 2025 Mar 7;26(7):1816–34. doi: 10.1038/s44319-025-00403-x (PMC11977213; doi:10.1038/s44319-025-00403-x)

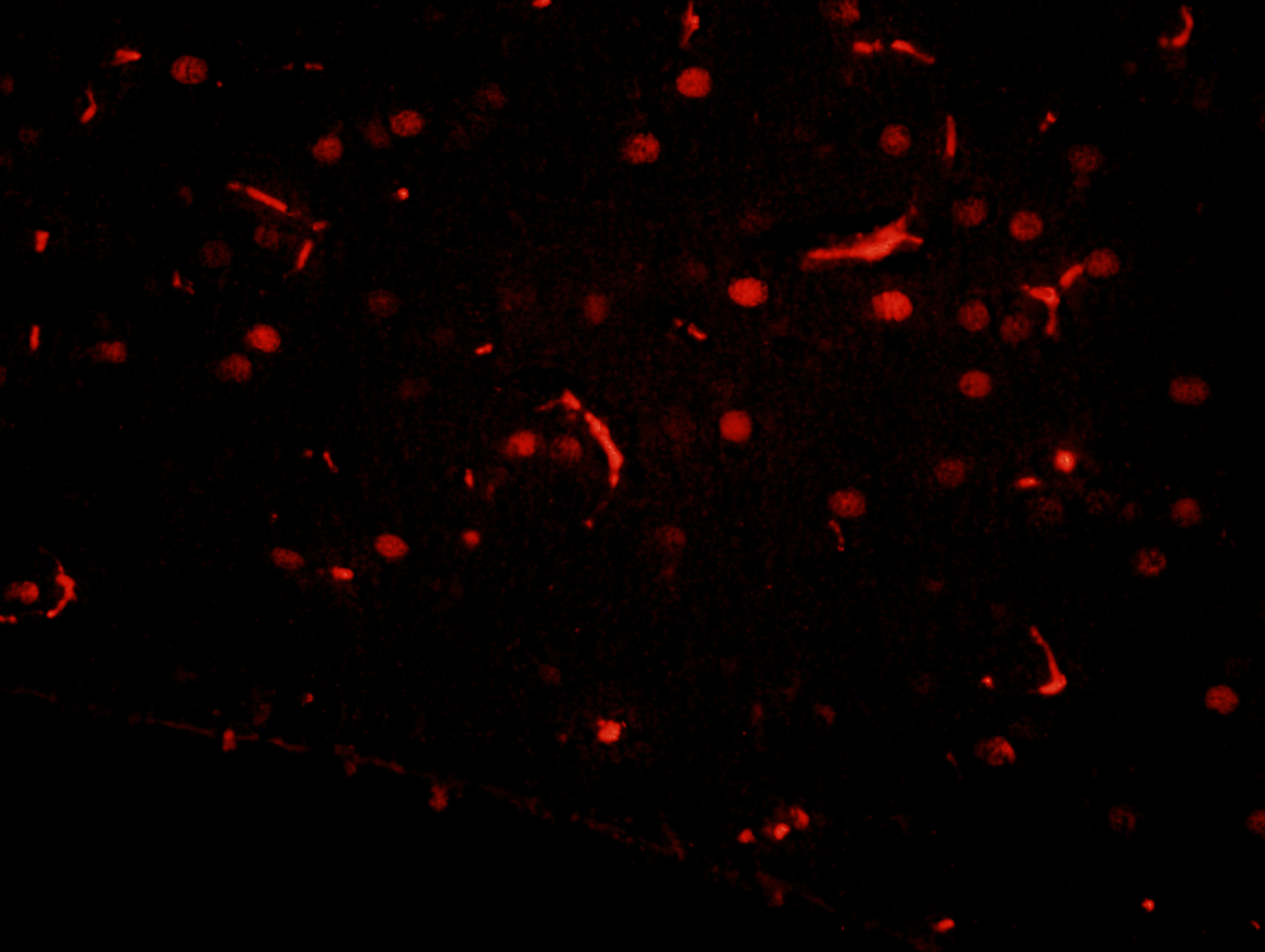

Supplement: Supplementary file 3 — Source data Fig. 1 [file 44319_2025_403_MOESM3_ESM.zip › Figure 1/1D/Tone/c-Fos.tif]

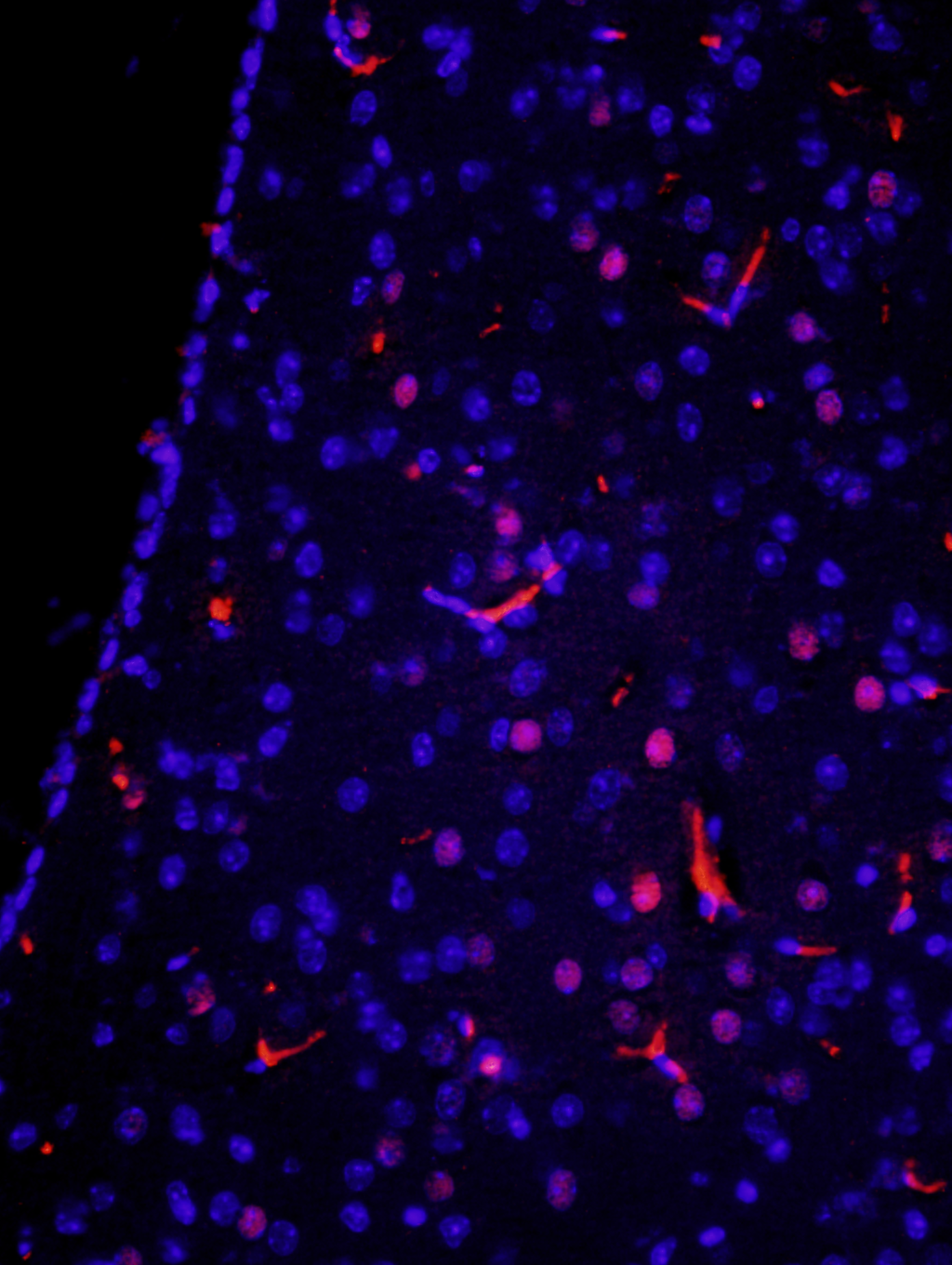

Supplement: Supplementary file 3 — Source data Fig. 1 [file 44319_2025_403_MOESM3_ESM.zip › Figure 1/1D/Tone/overlay.tif]

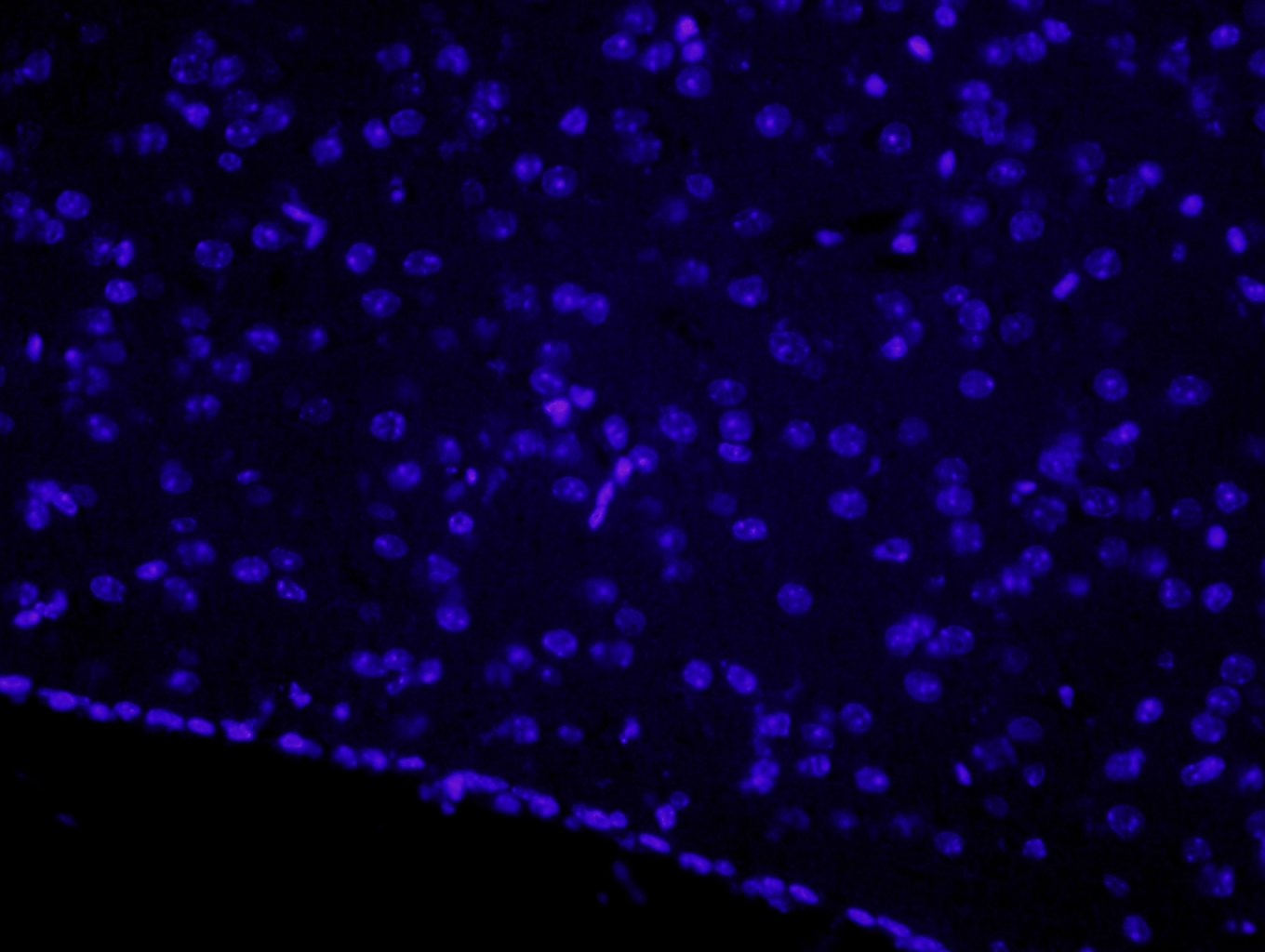

Supplement: Supplementary file 3 — Source data Fig. 1 [file 44319_2025_403_MOESM3_ESM.zip › Figure 1/1D/Tone/Hoechst.tif]

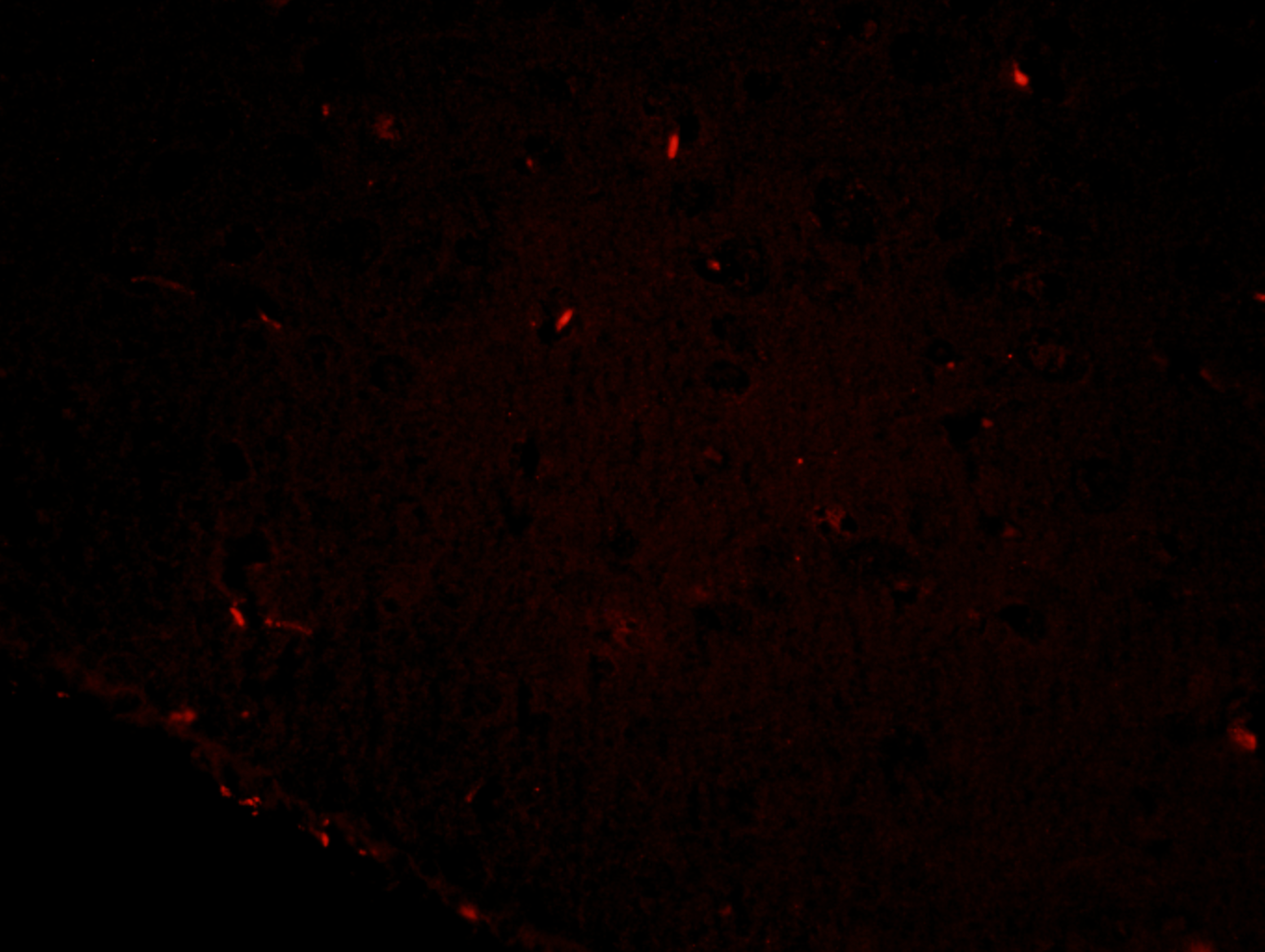

Supplement: Supplementary file 3 — Source data Fig. 1 [file 44319_2025_403_MOESM3_ESM.zip › Figure 1/1D/Control/c-Fos.tif]

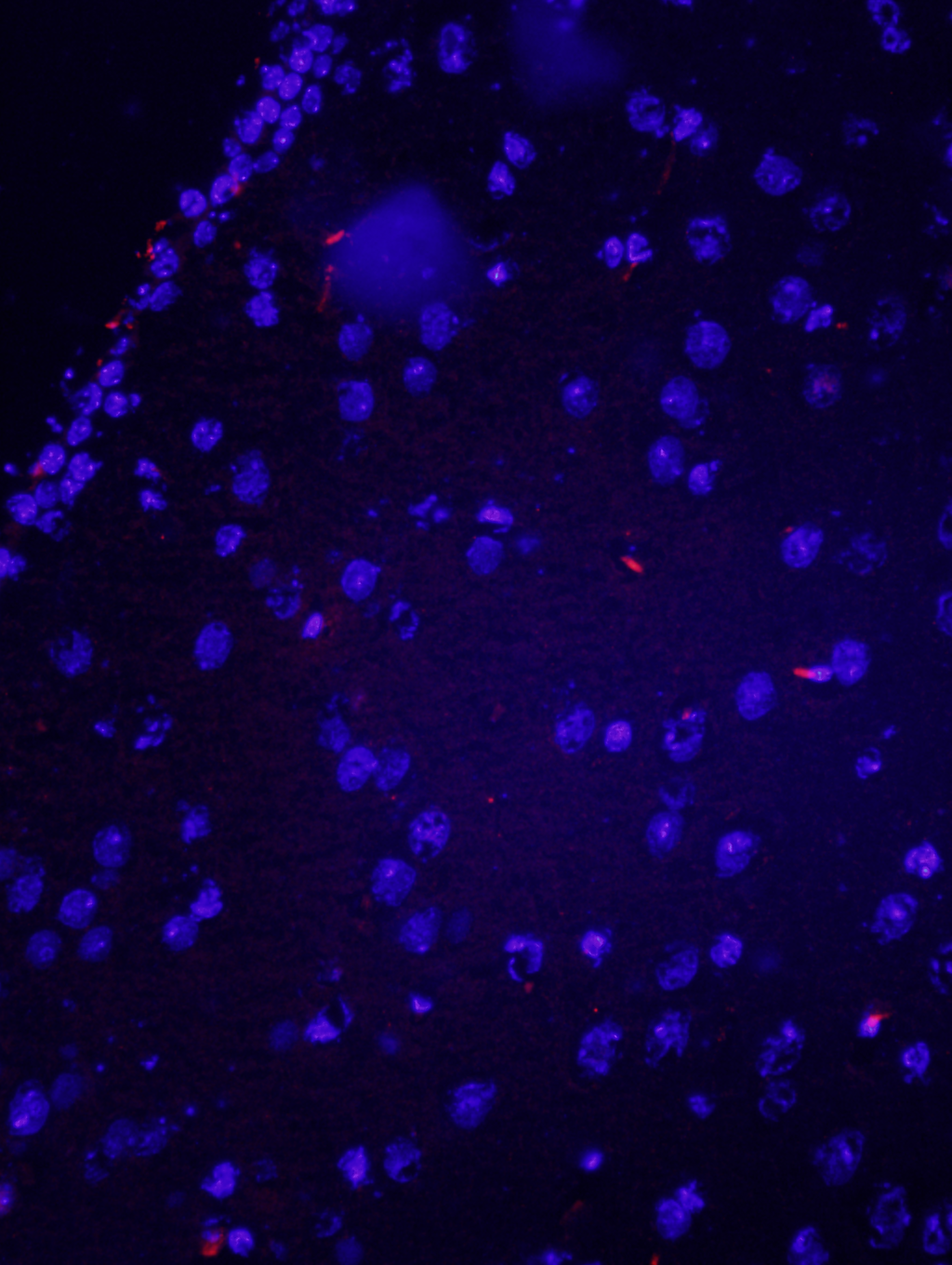

Supplement: Supplementary file 3 — Source data Fig. 1 [file 44319_2025_403_MOESM3_ESM.zip › Figure 1/1D/Control/overlay.tif]

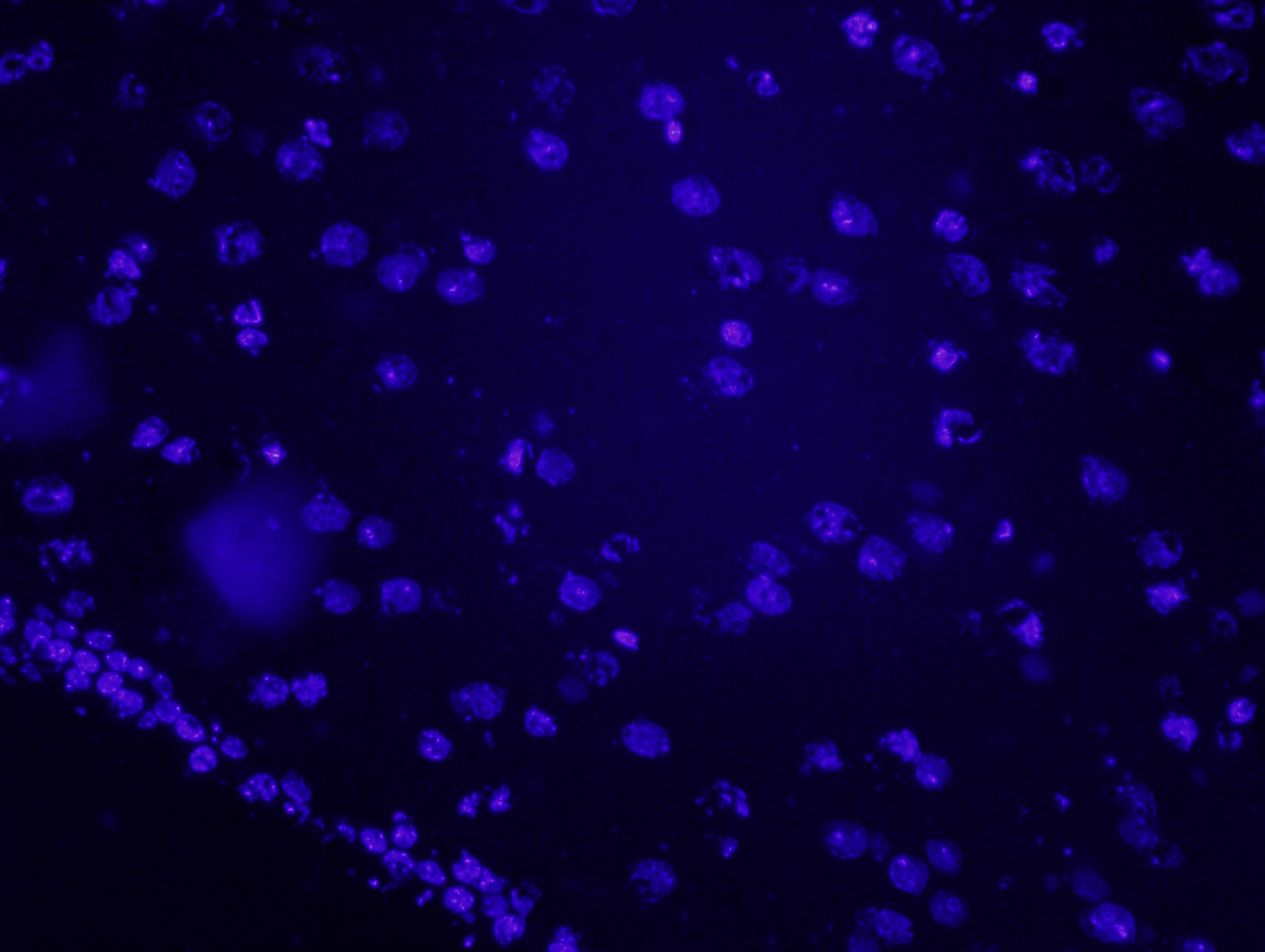

Supplement: Supplementary file 3 — Source data Fig. 1 [file 44319_2025_403_MOESM3_ESM.zip › Figure 1/1D/Control/Hoechst.tif]

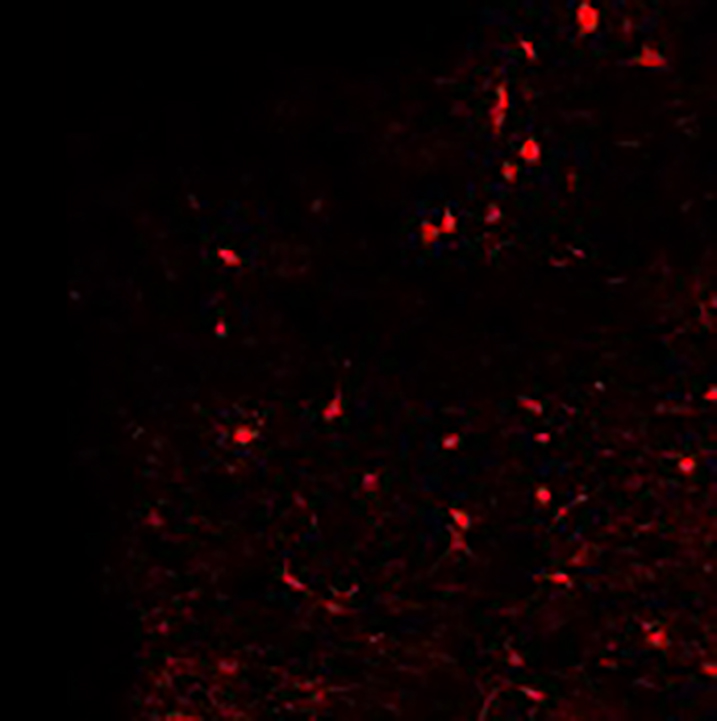

Supplement: Supplementary file 4 — Source data Fig. 2 [file 44319_2025_403_MOESM4_ESM.zip › Figure 2/2B/hM4Di-mCherry.jpg]

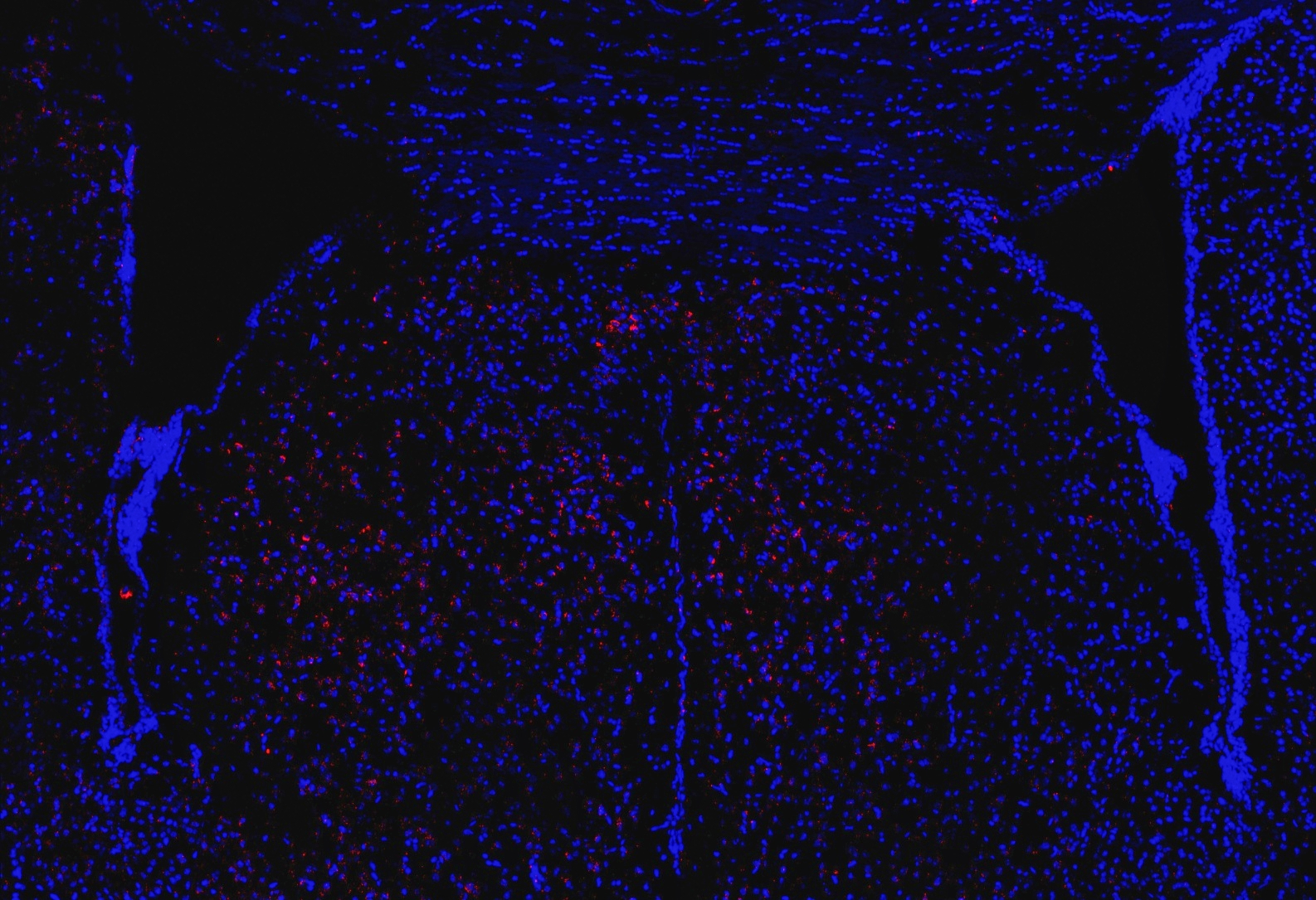

Supplement: Supplementary file 4 — Source data Fig. 2 [file 44319_2025_403_MOESM4_ESM.zip › Figure 2/2B/AAV injection position overlay.jpeg]

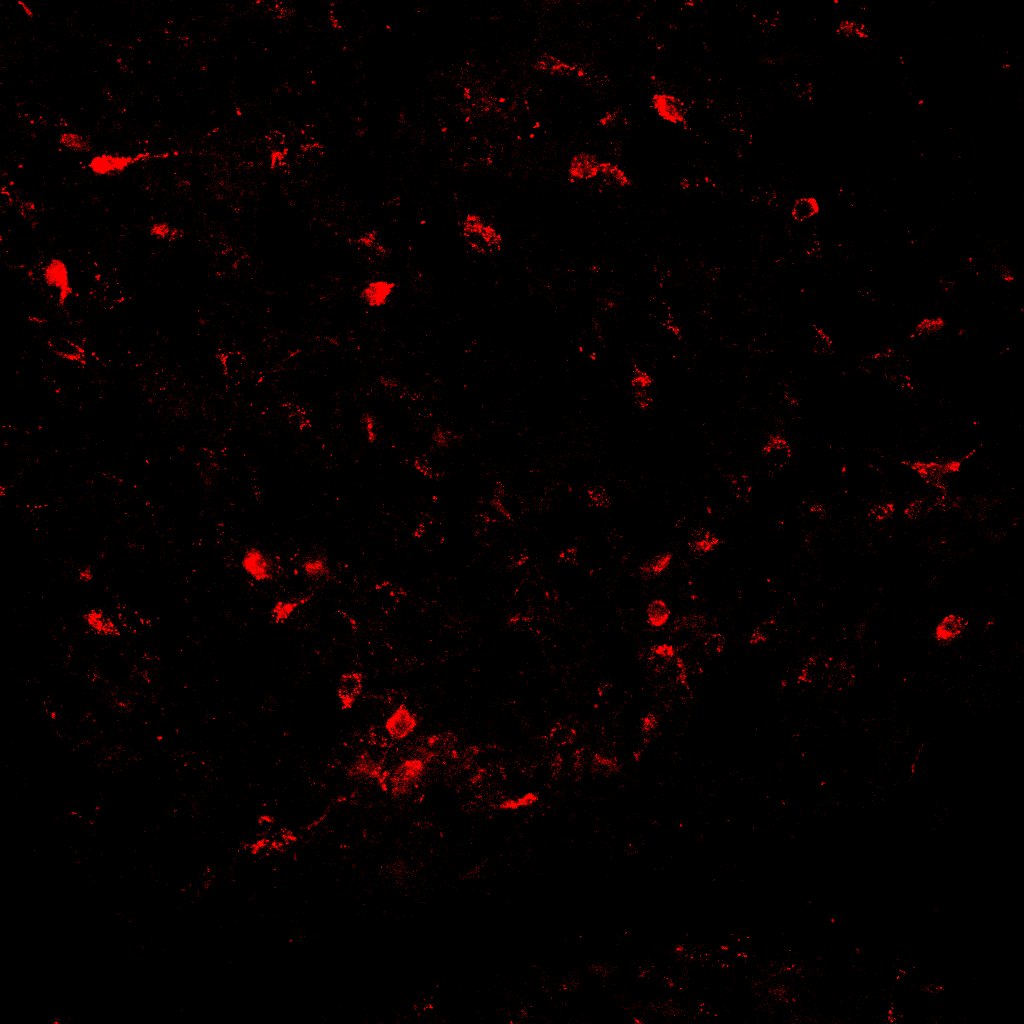

Supplement: Supplementary file 4 — Source data Fig. 2 [file 44319_2025_403_MOESM4_ESM.zip › Figure 2/2C/AAV-hM4DGi-mCherry+Compound 21/mCherry.tif]

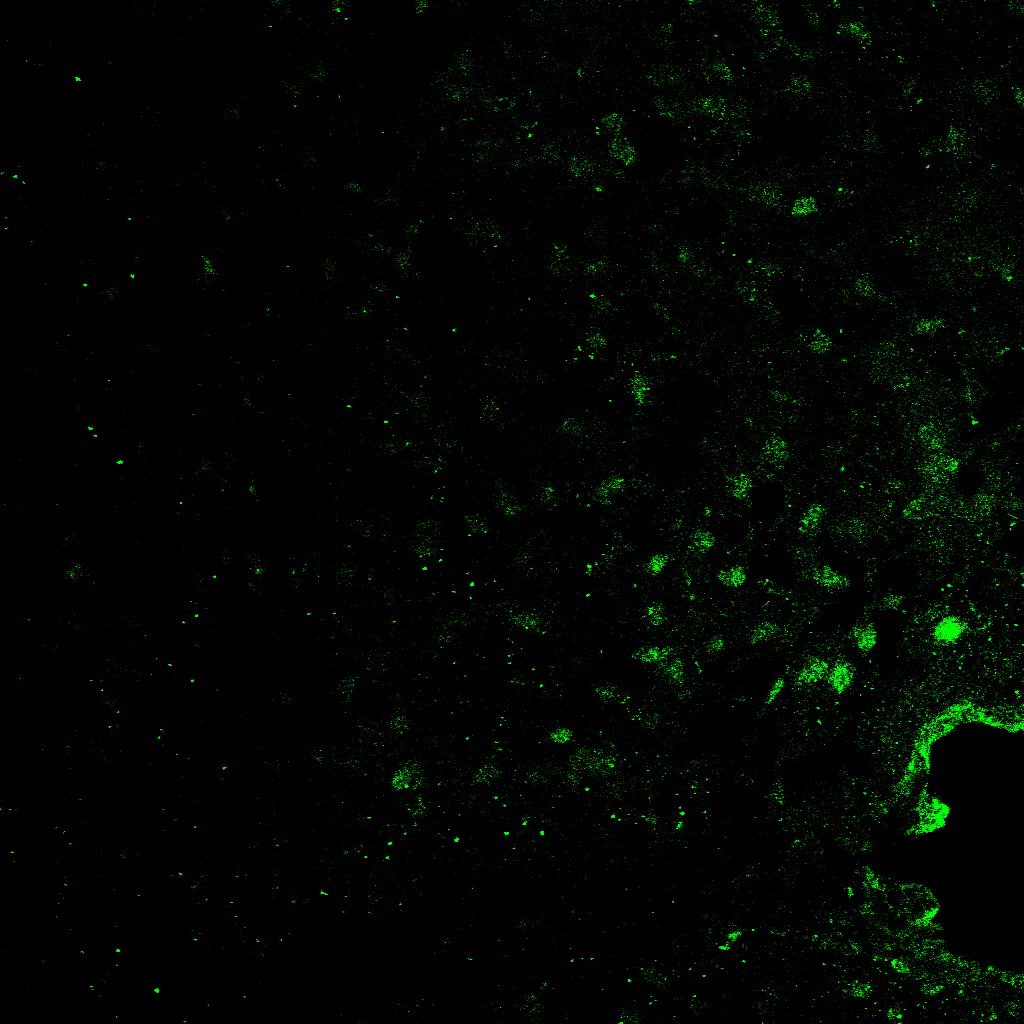

Supplement: Supplementary file 4 — Source data Fig. 2 [file 44319_2025_403_MOESM4_ESM.zip › Figure 2/2C/AAV-hM4DGi-mCherry+Compound 21/c-Fos.tif]

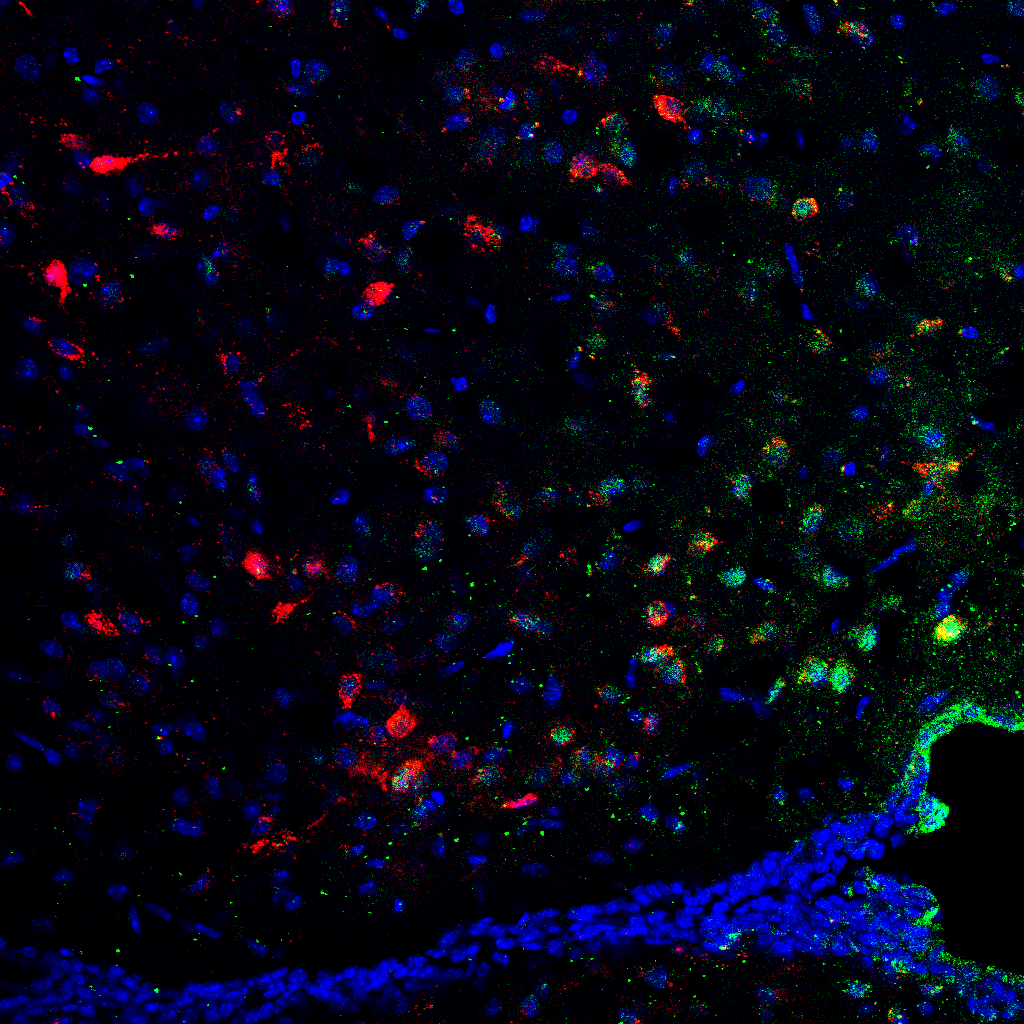

Supplement: Supplementary file 4 — Source data Fig. 2 [file 44319_2025_403_MOESM4_ESM.zip › Figure 2/2C/AAV-hM4DGi-mCherry+Compound 21/overlay.tif]

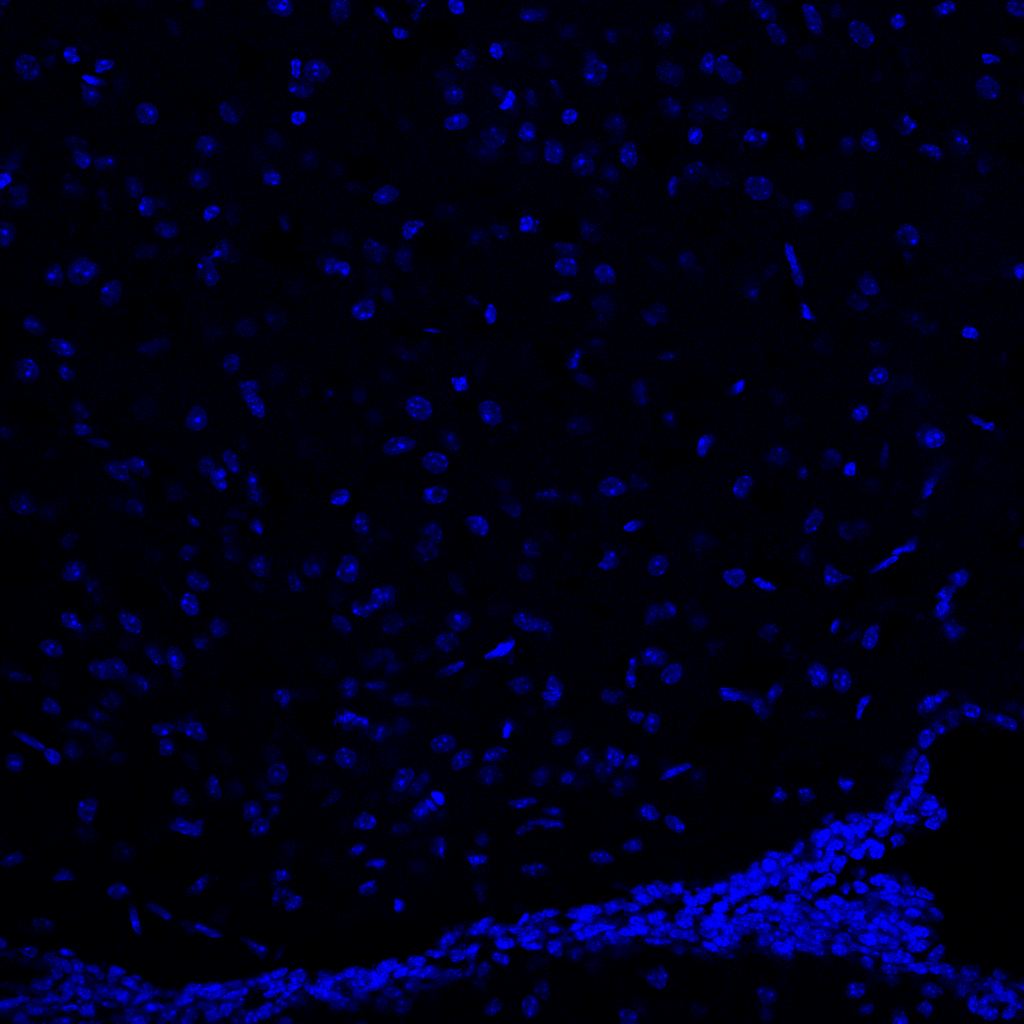

Supplement: Supplementary file 4 — Source data Fig. 2 [file 44319_2025_403_MOESM4_ESM.zip › Figure 2/2C/AAV-hM4DGi-mCherry+Compound 21/Hoechst.tif]

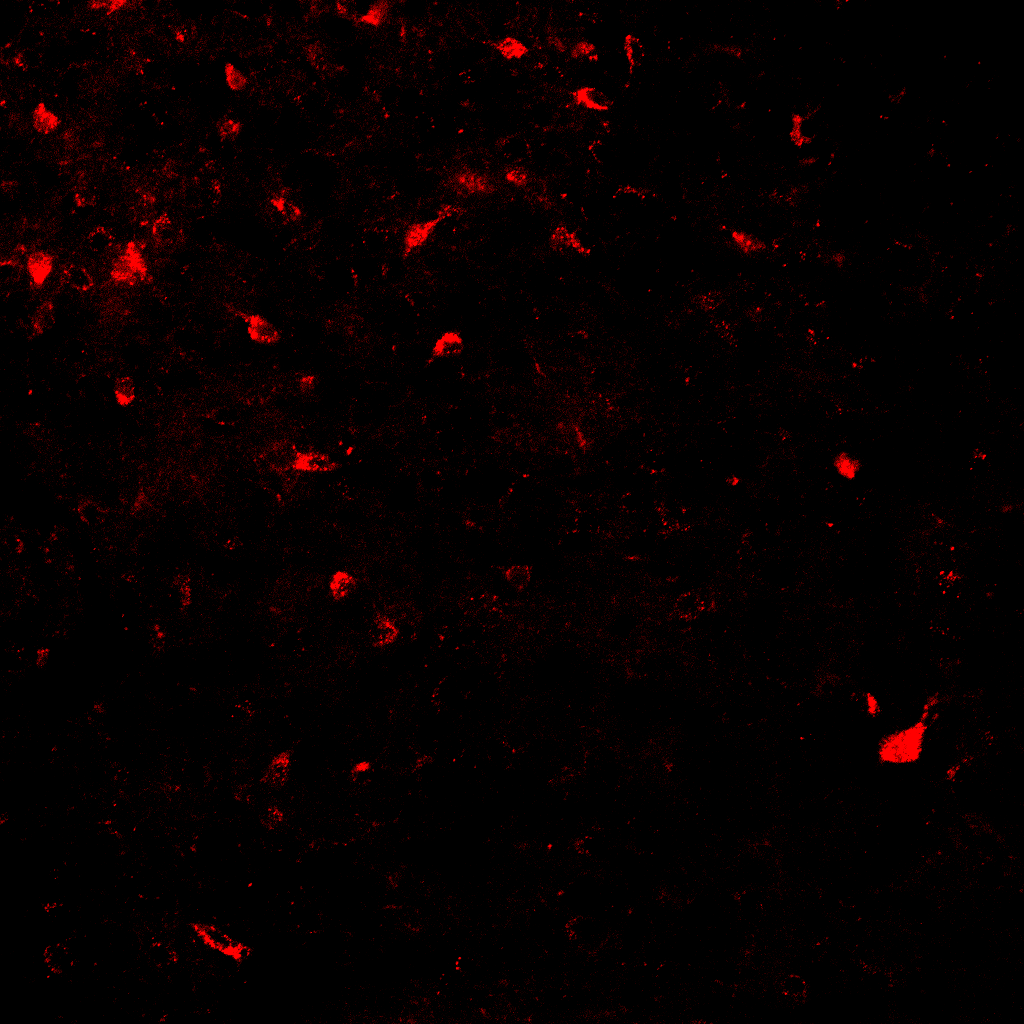

Supplement: Supplementary file 4 — Source data Fig. 2 [file 44319_2025_403_MOESM4_ESM.zip › Figure 2/2C/AAV-hM4Di-mCherry/mCherry.tif]

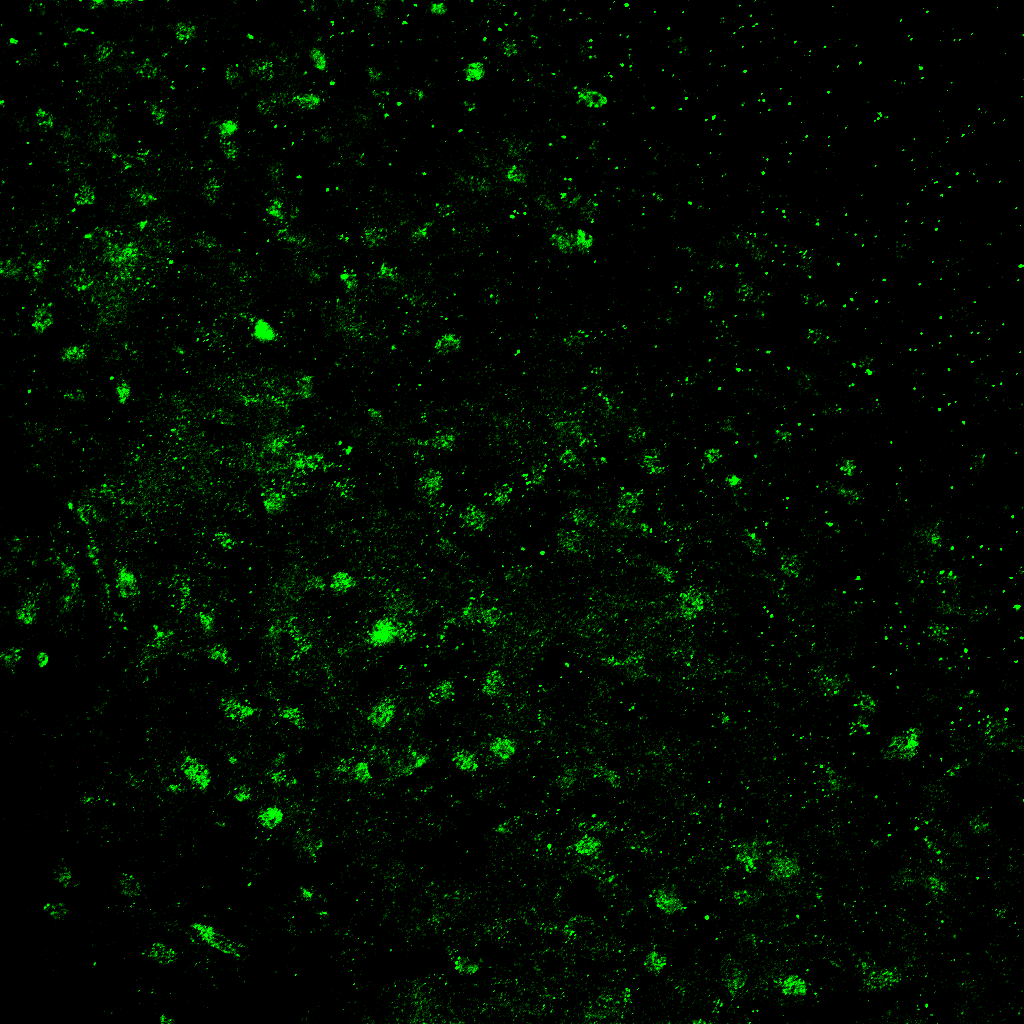

Supplement: Supplementary file 4 — Source data Fig. 2 [file 44319_2025_403_MOESM4_ESM.zip › Figure 2/2C/AAV-hM4Di-mCherry/c-Fos.tif]

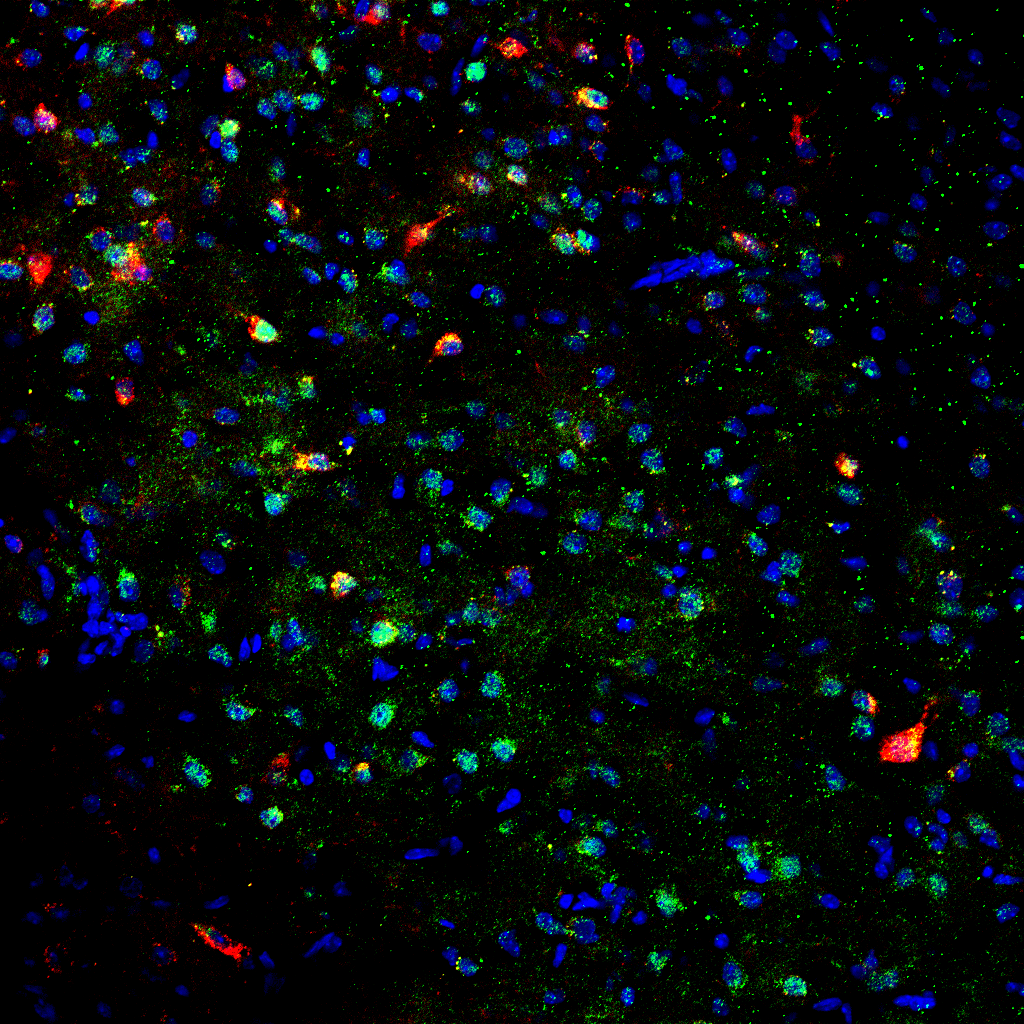

Supplement: Supplementary file 4 — Source data Fig. 2 [file 44319_2025_403_MOESM4_ESM.zip › Figure 2/2C/AAV-hM4Di-mCherry/overlay.tif]

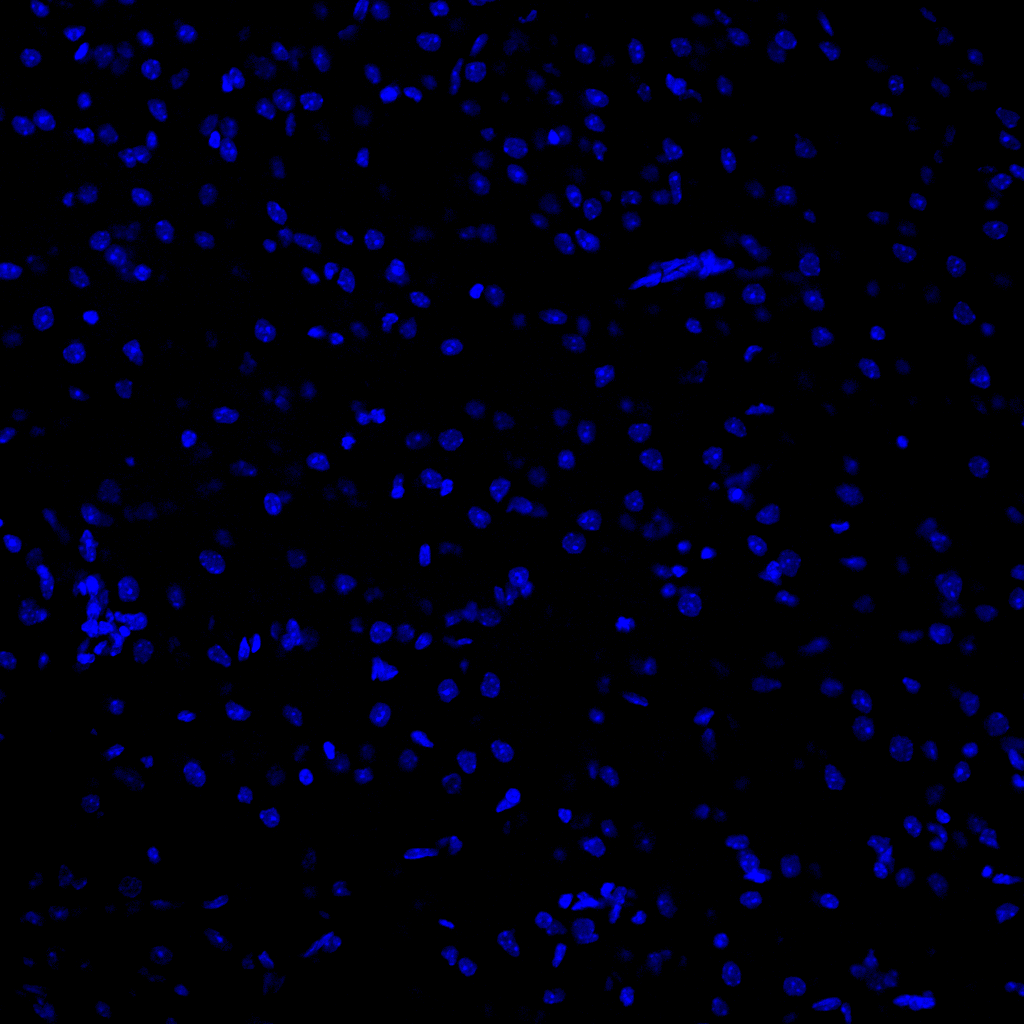

Supplement: Supplementary file 4 — Source data Fig. 2 [file 44319_2025_403_MOESM4_ESM.zip › Figure 2/2C/AAV-hM4Di-mCherry/Hoechst.tif]

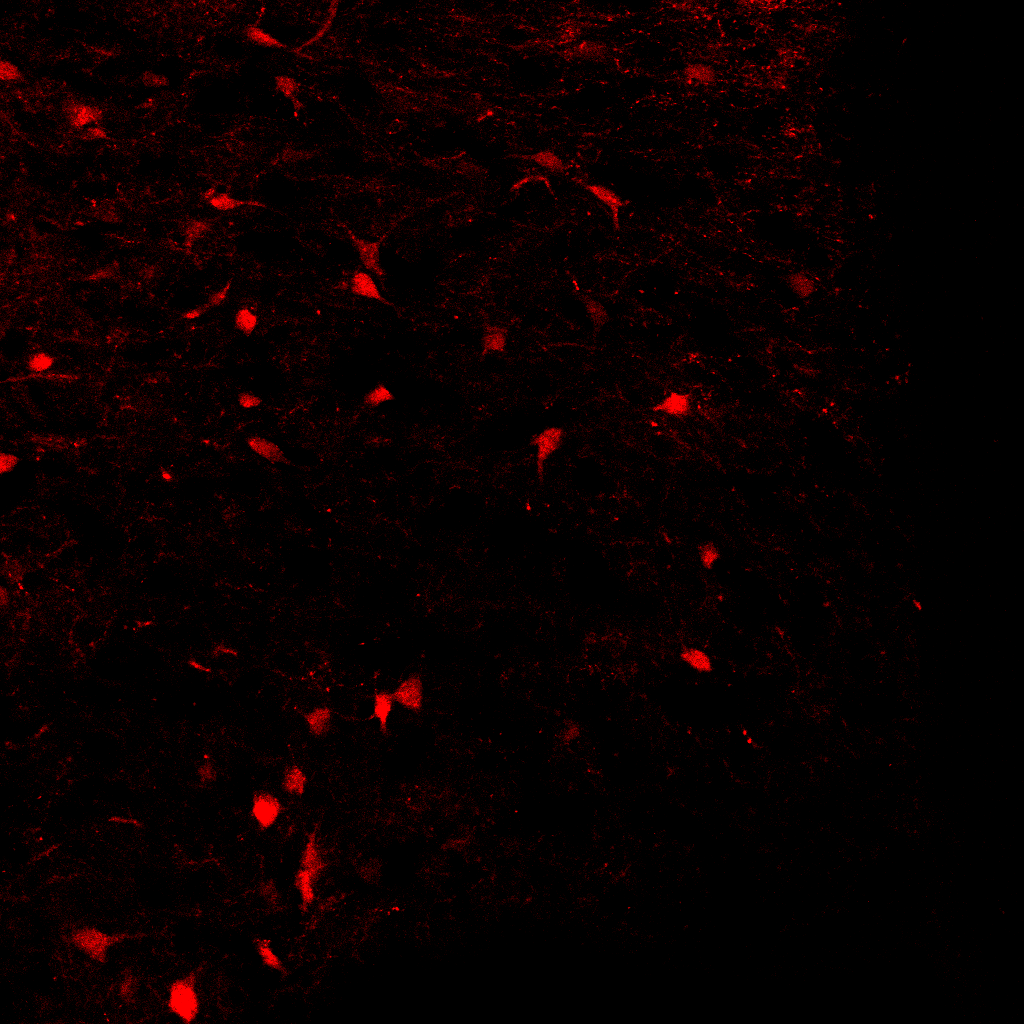

Supplement: Supplementary file 4 — Source data Fig. 2 [file 44319_2025_403_MOESM4_ESM.zip › Figure 2/2C/AAV-mCherry+Compound 21/mCherry.tif]

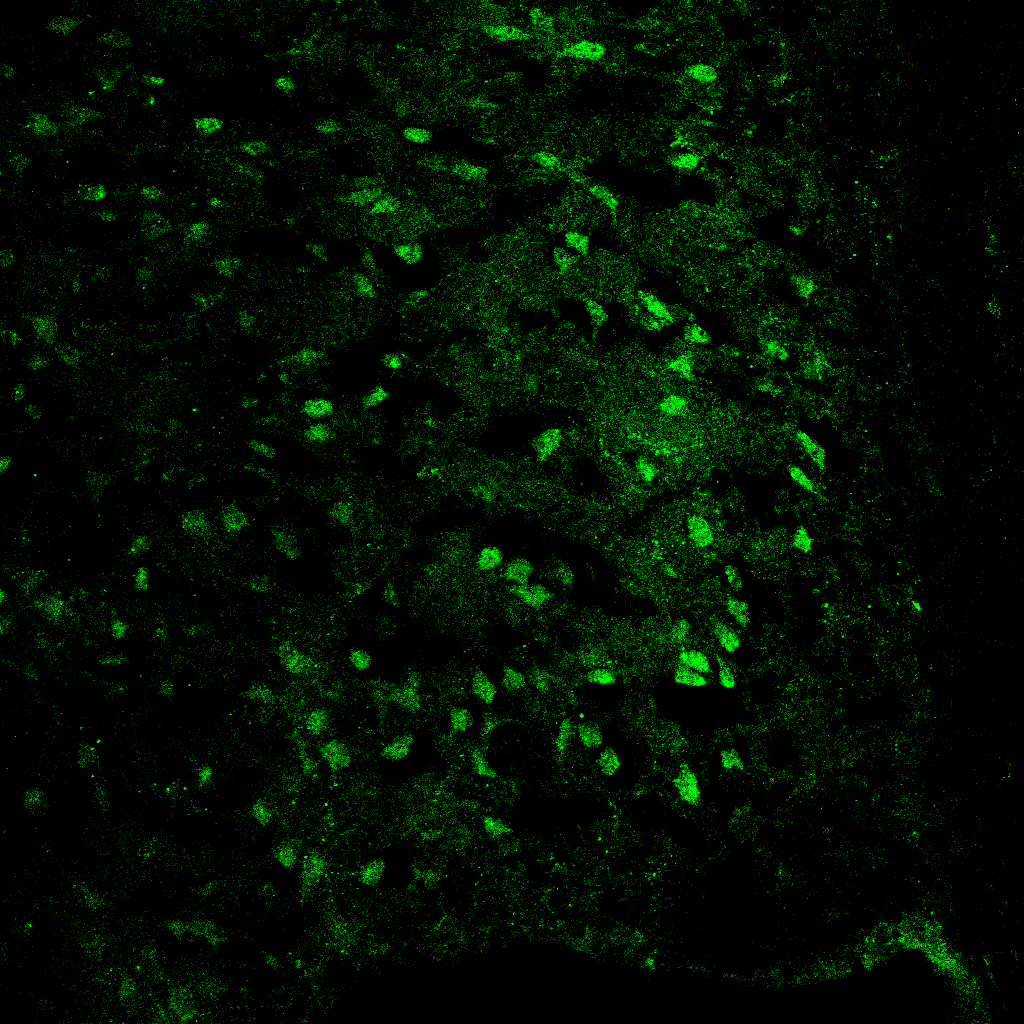

Supplement: Supplementary file 4 — Source data Fig. 2 [file 44319_2025_403_MOESM4_ESM.zip › Figure 2/2C/AAV-mCherry+Compound 21/c-Fos.tif]

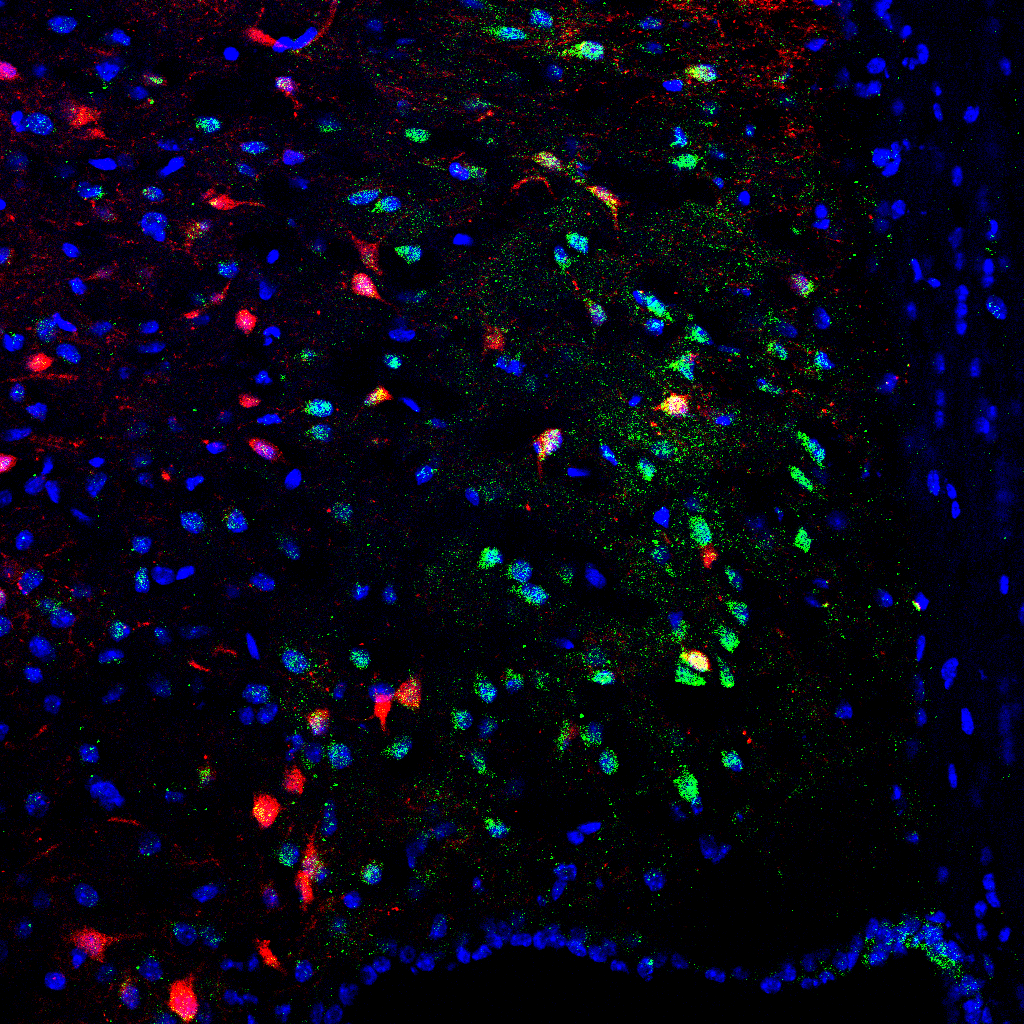

Supplement: Supplementary file 4 — Source data Fig. 2 [file 44319_2025_403_MOESM4_ESM.zip › Figure 2/2C/AAV-mCherry+Compound 21/overlay.tif]

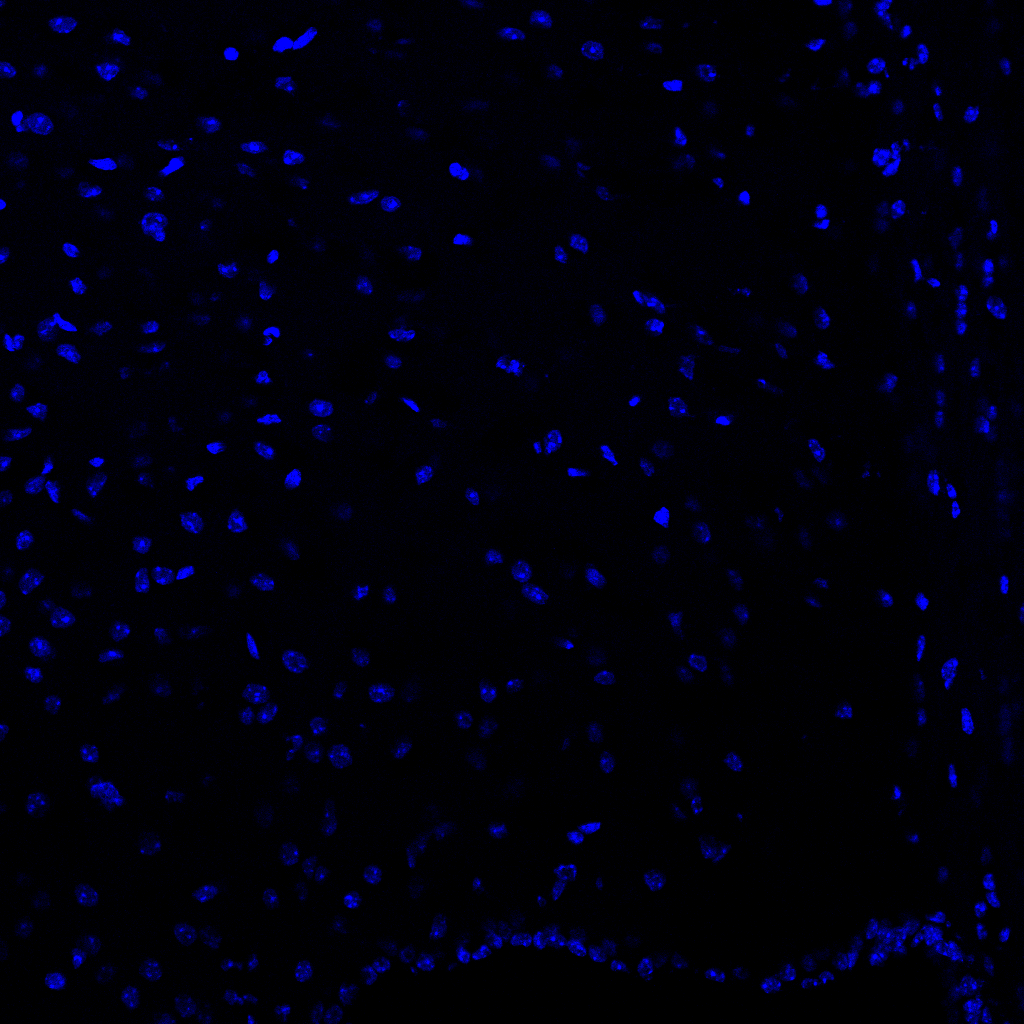

Supplement: Supplementary file 4 — Source data Fig. 2 [file 44319_2025_403_MOESM4_ESM.zip › Figure 2/2C/AAV-mCherry+Compound 21/Hoechst.tif]

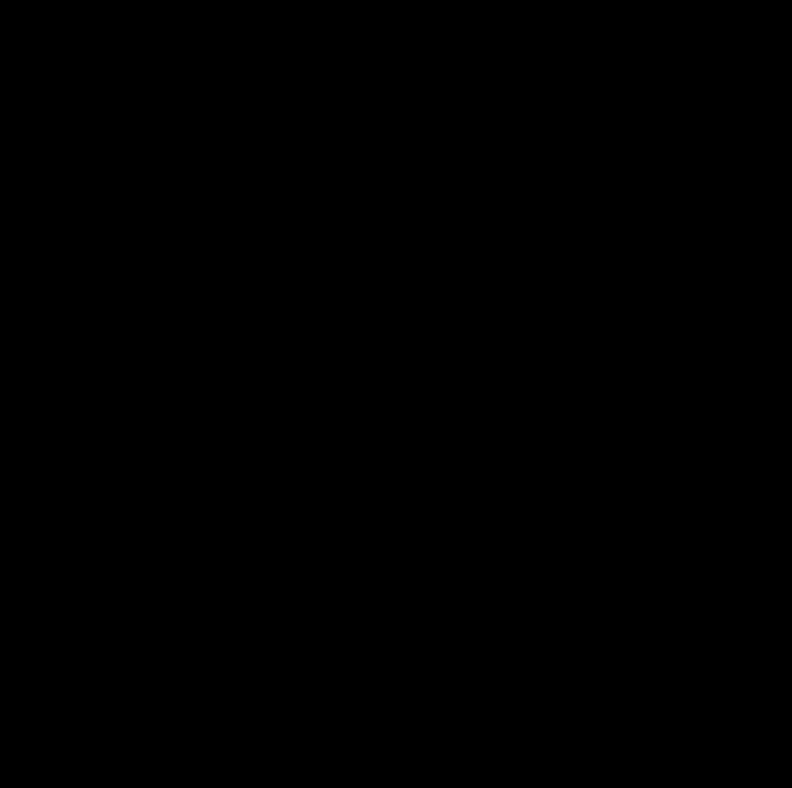

Supplement: Supplementary file 4 — Source data Fig. 2 [file 44319_2025_403_MOESM4_ESM.zip › Figure 2/2C/Control/mCherry.tif]

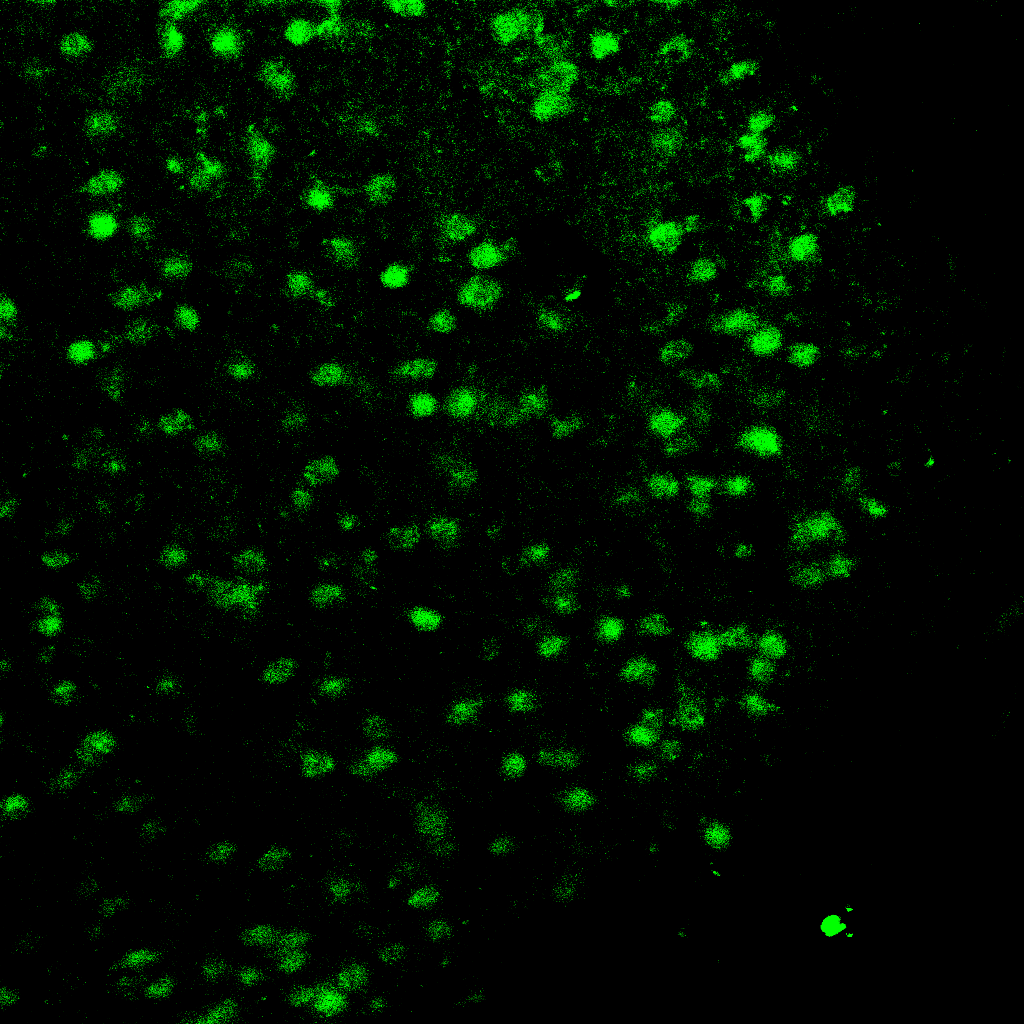

Supplement: Supplementary file 4 — Source data Fig. 2 [file 44319_2025_403_MOESM4_ESM.zip › Figure 2/2C/Control/c-Fos.tif]

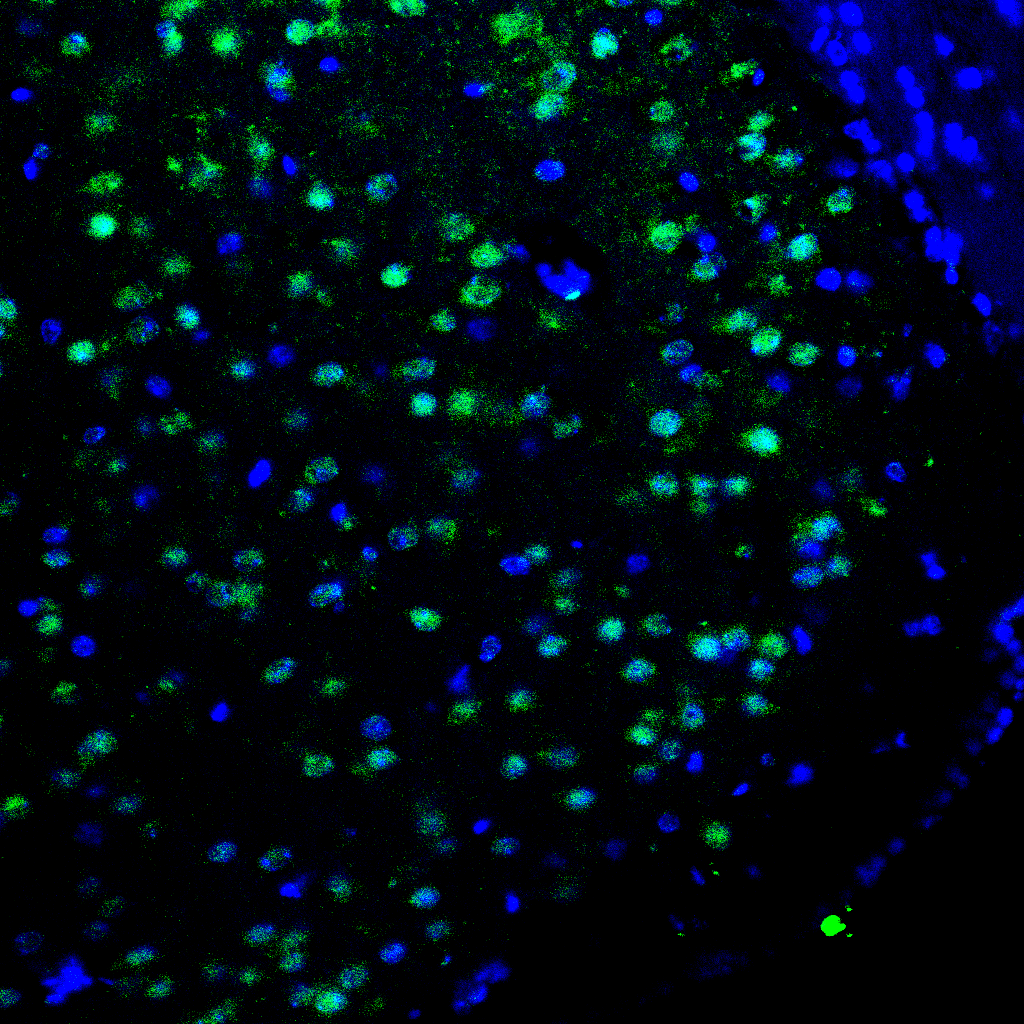

Supplement: Supplementary file 4 — Source data Fig. 2 [file 44319_2025_403_MOESM4_ESM.zip › Figure 2/2C/Control/overlay.tif]

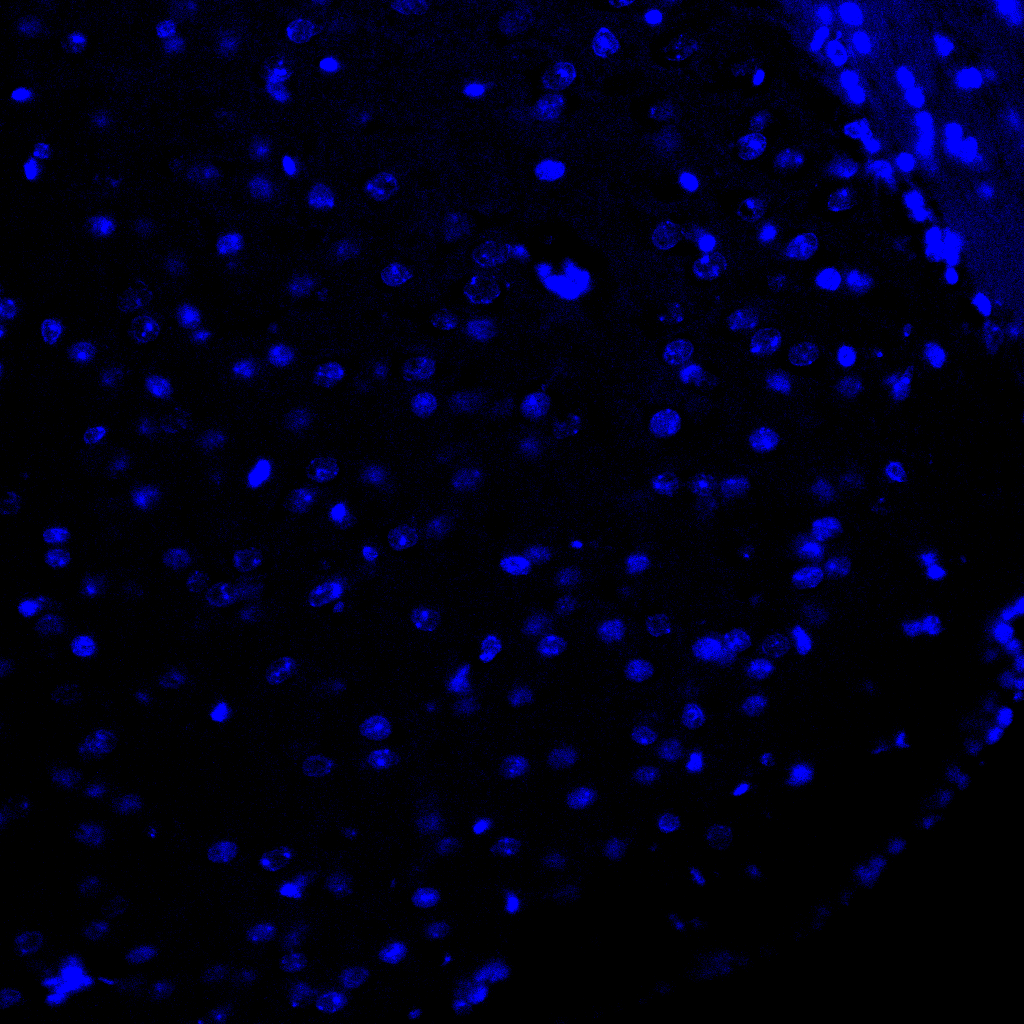

Supplement: Supplementary file 4 — Source data Fig. 2 [file 44319_2025_403_MOESM4_ESM.zip › Figure 2/2C/Control/Hoechst.tif]

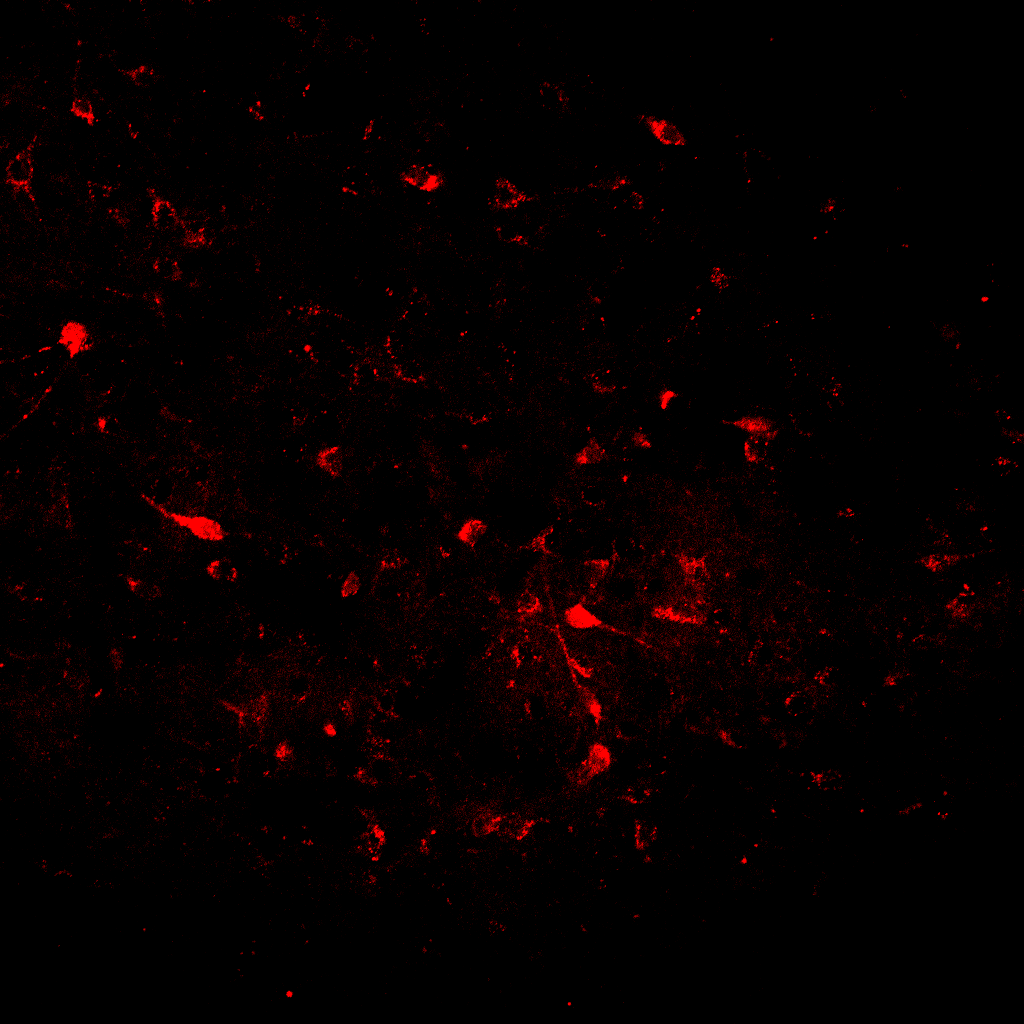

Supplement: Supplementary file 4 — Source data Fig. 2 [file 44319_2025_403_MOESM4_ESM.zip › Figure 2/2E/AAV-hM4Di-mCherry/mCherry.tif]

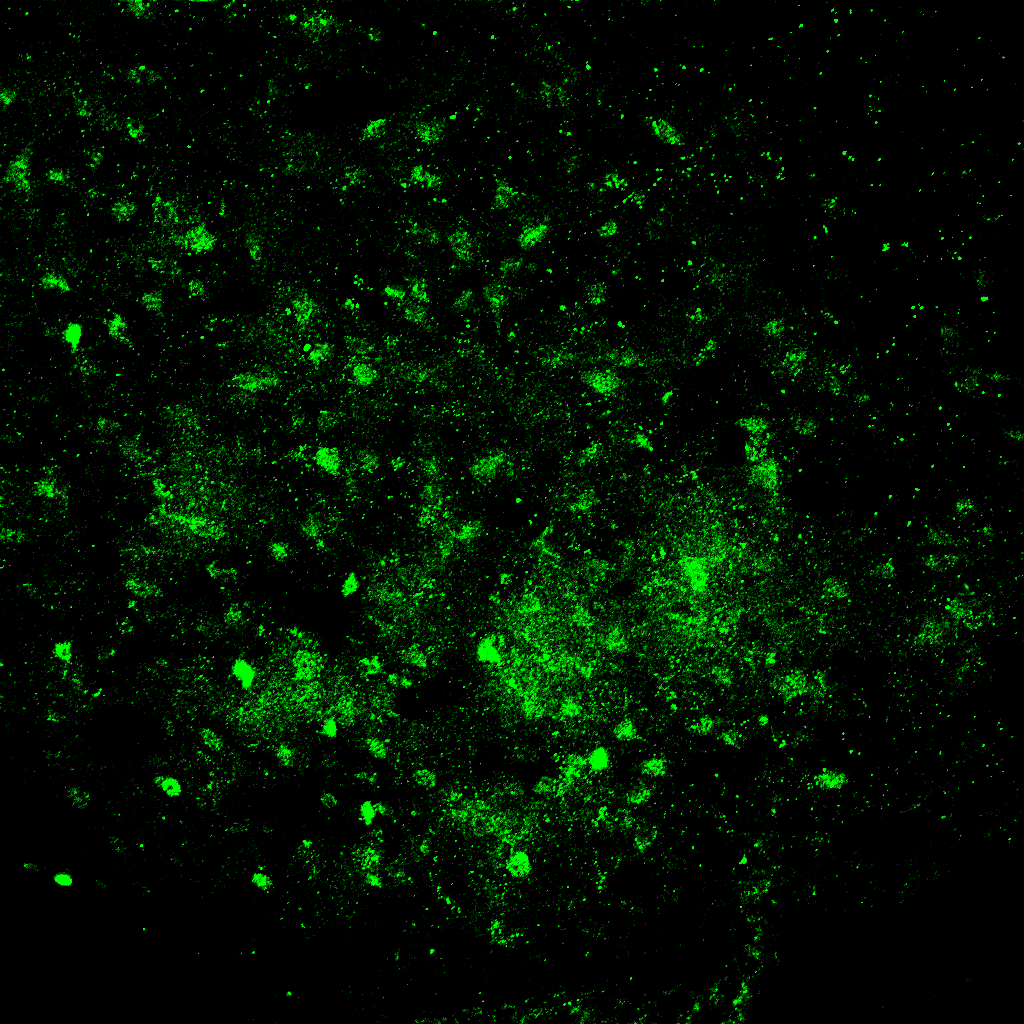

Supplement: Supplementary file 4 — Source data Fig. 2 [file 44319_2025_403_MOESM4_ESM.zip › Figure 2/2E/AAV-hM4Di-mCherry/c-Fos.tif]

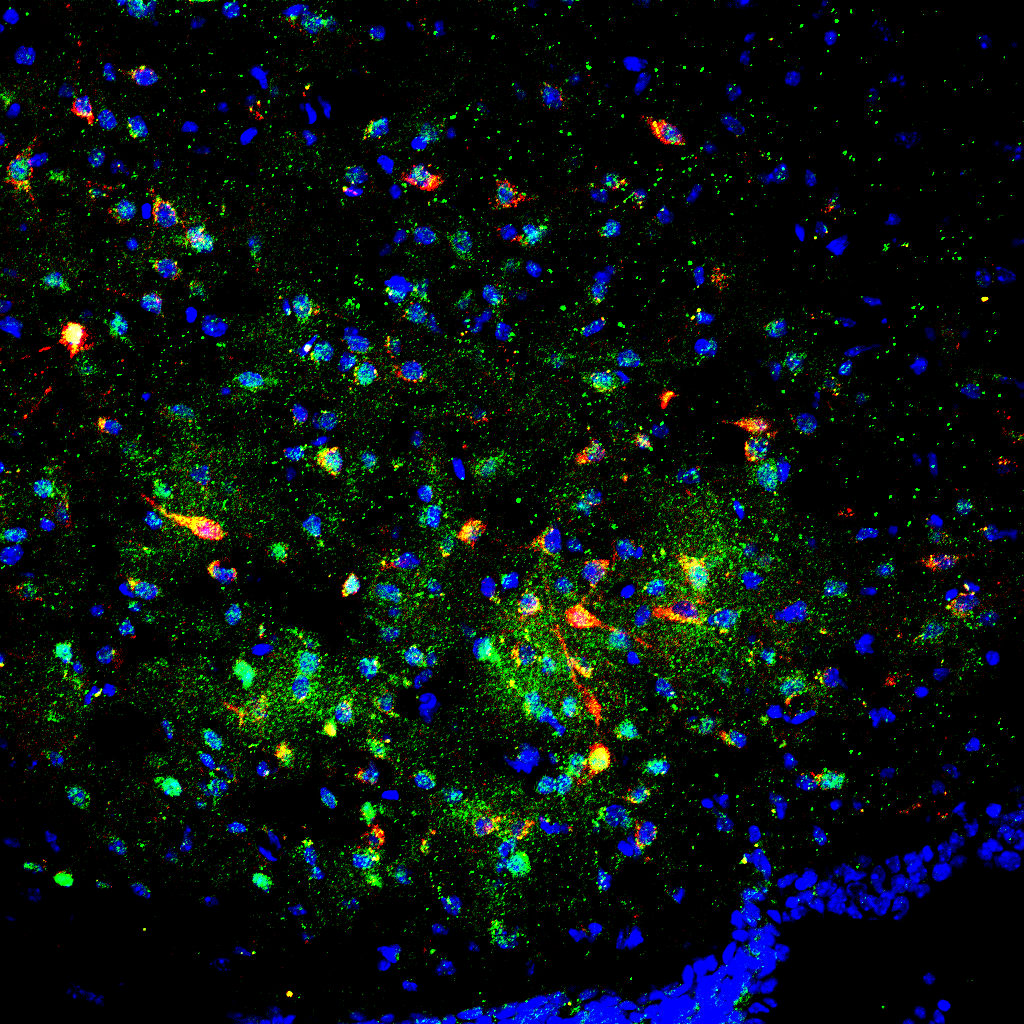

Supplement: Supplementary file 4 — Source data Fig. 2 [file 44319_2025_403_MOESM4_ESM.zip › Figure 2/2E/AAV-hM4Di-mCherry/overlay.tif]

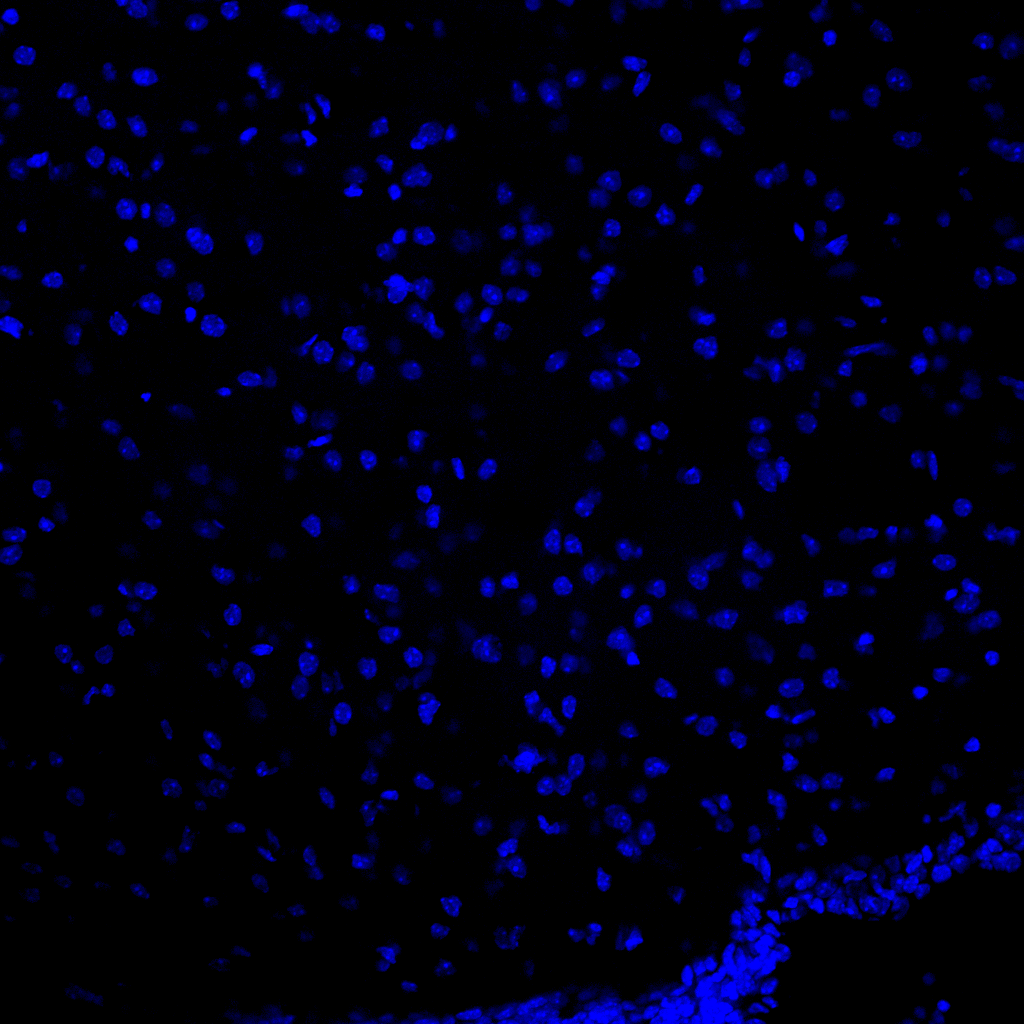

Supplement: Supplementary file 4 — Source data Fig. 2 [file 44319_2025_403_MOESM4_ESM.zip › Figure 2/2E/AAV-hM4Di-mCherry/Hoechst.tif]

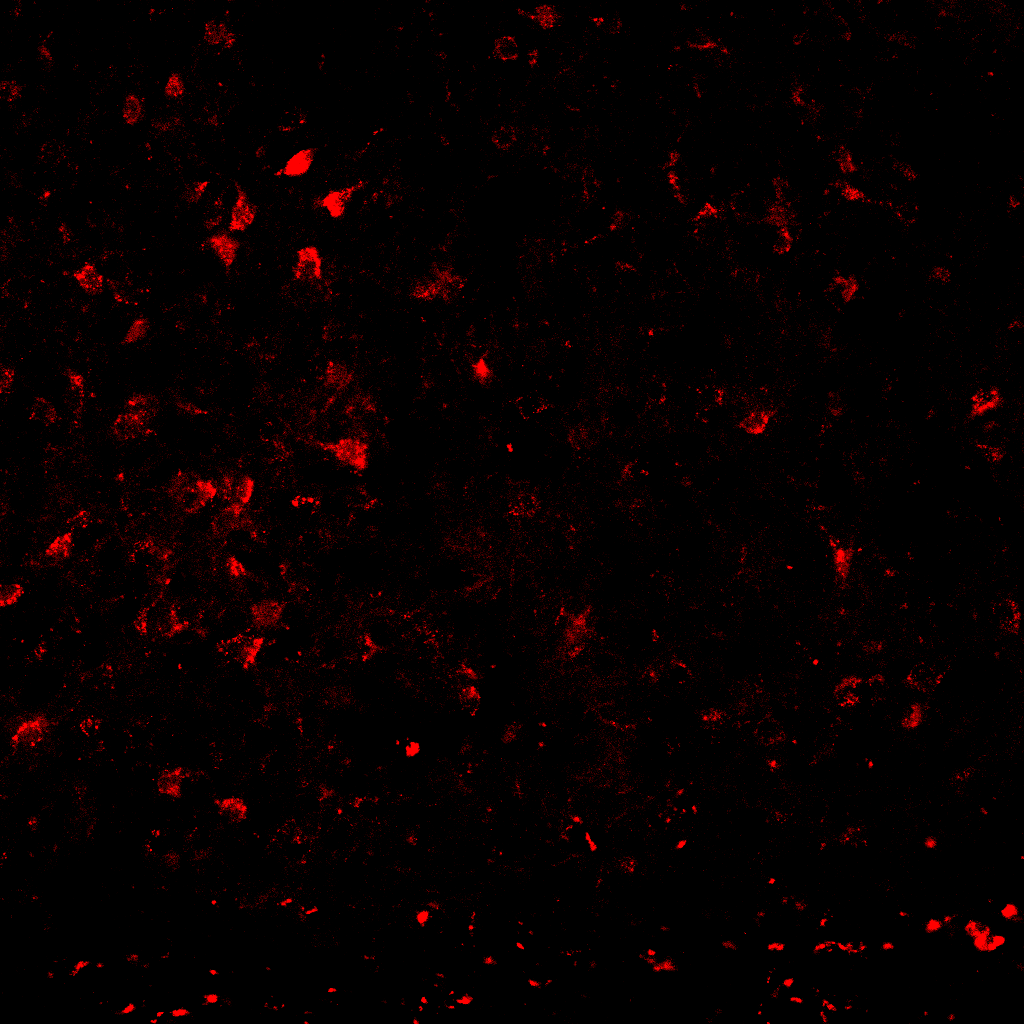

Supplement: Supplementary file 4 — Source data Fig. 2 [file 44319_2025_403_MOESM4_ESM.zip › Figure 2/2E/AAV-hM4Di-mCherry+Compound 21/mCherry.tif]

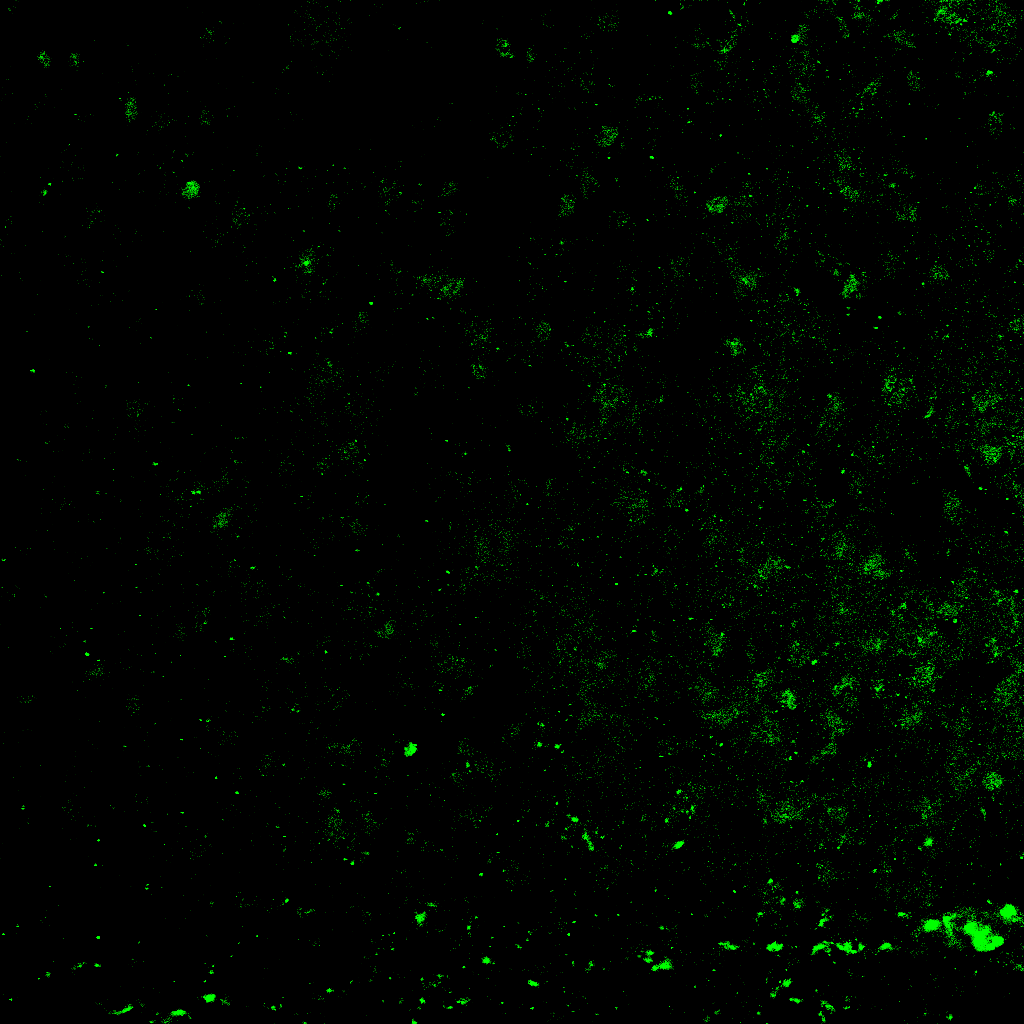

Supplement: Supplementary file 4 — Source data Fig. 2 [file 44319_2025_403_MOESM4_ESM.zip › Figure 2/2E/AAV-hM4Di-mCherry+Compound 21/c-Fos.tif]

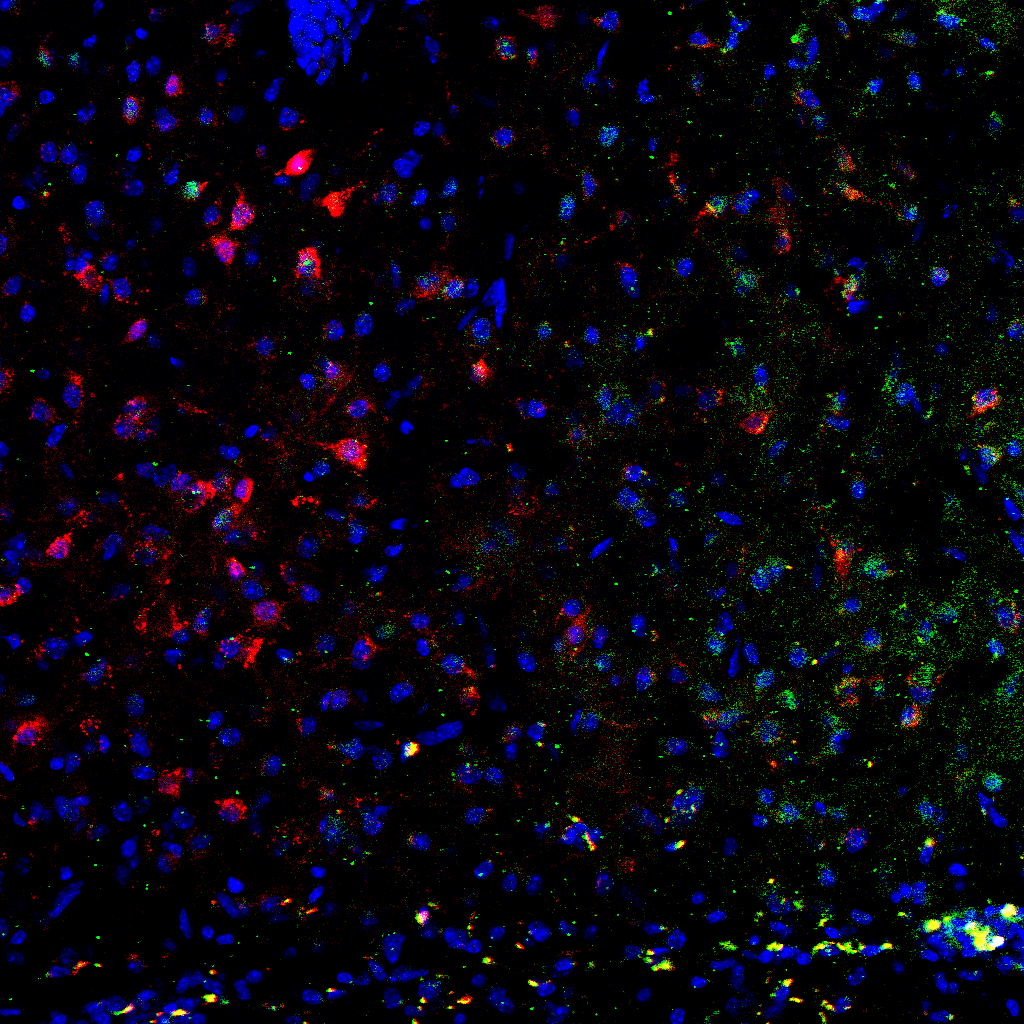

Supplement: Supplementary file 4 — Source data Fig. 2 [file 44319_2025_403_MOESM4_ESM.zip › Figure 2/2E/AAV-hM4Di-mCherry+Compound 21/overlay.tif]

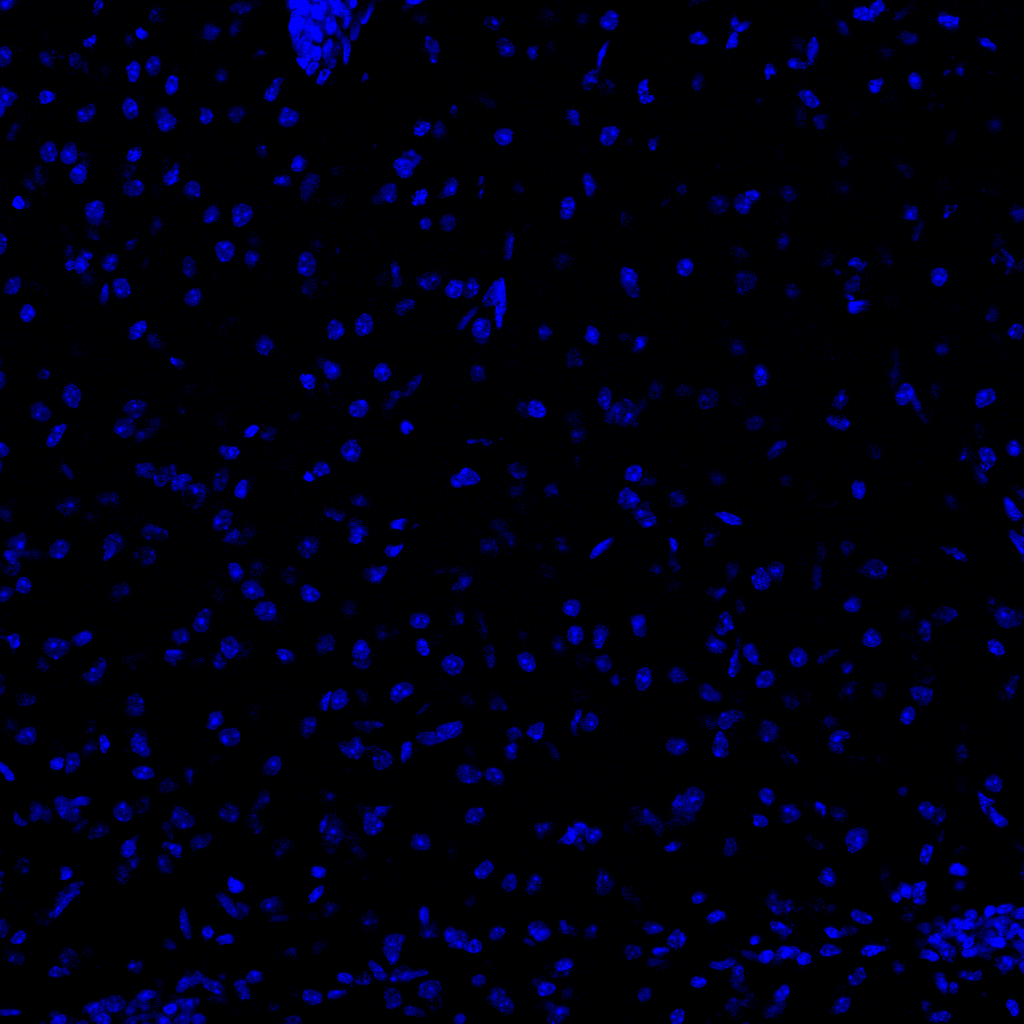

Supplement: Supplementary file 4 — Source data Fig. 2 [file 44319_2025_403_MOESM4_ESM.zip › Figure 2/2E/AAV-hM4Di-mCherry+Compound 21/Hoechst.tif]

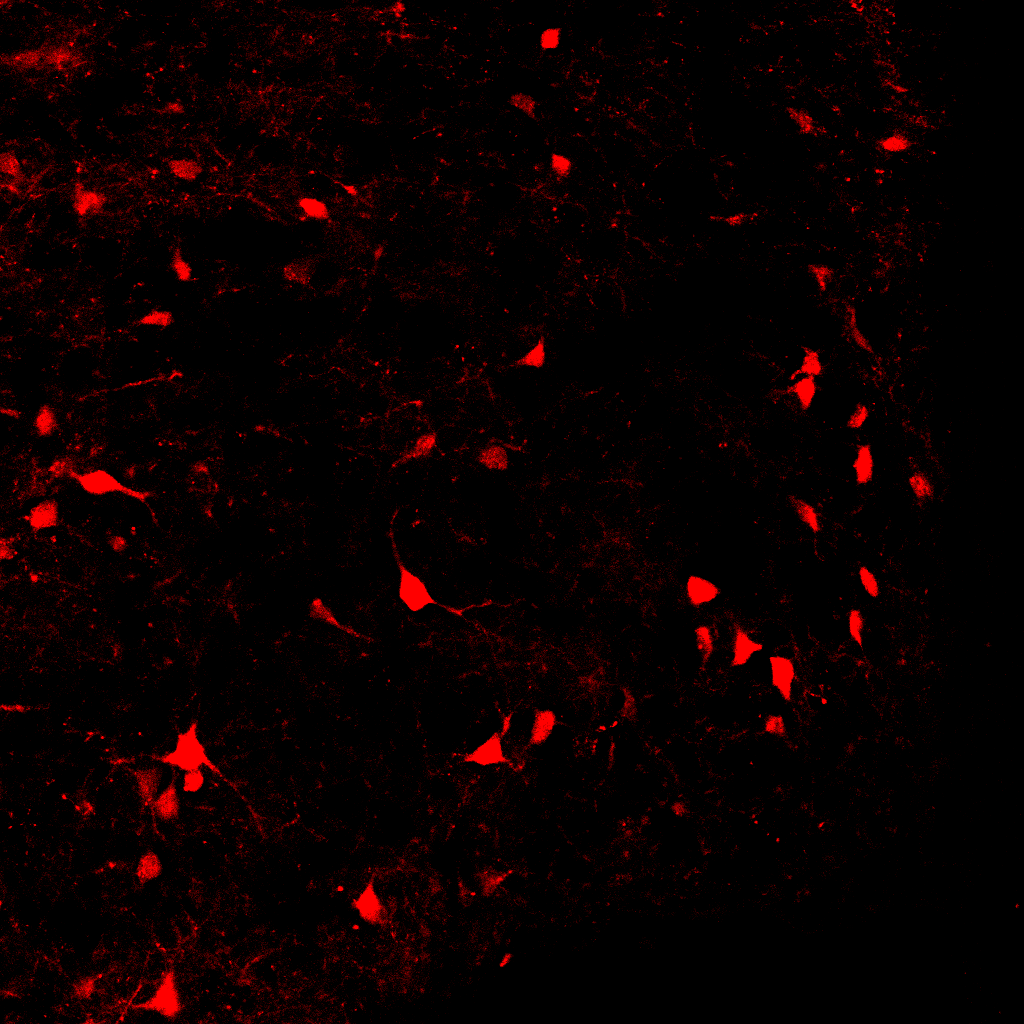

Supplement: Supplementary file 4 — Source data Fig. 2 [file 44319_2025_403_MOESM4_ESM.zip › Figure 2/2E/AAV-mCherry+Compound 21/mCherry.tif]

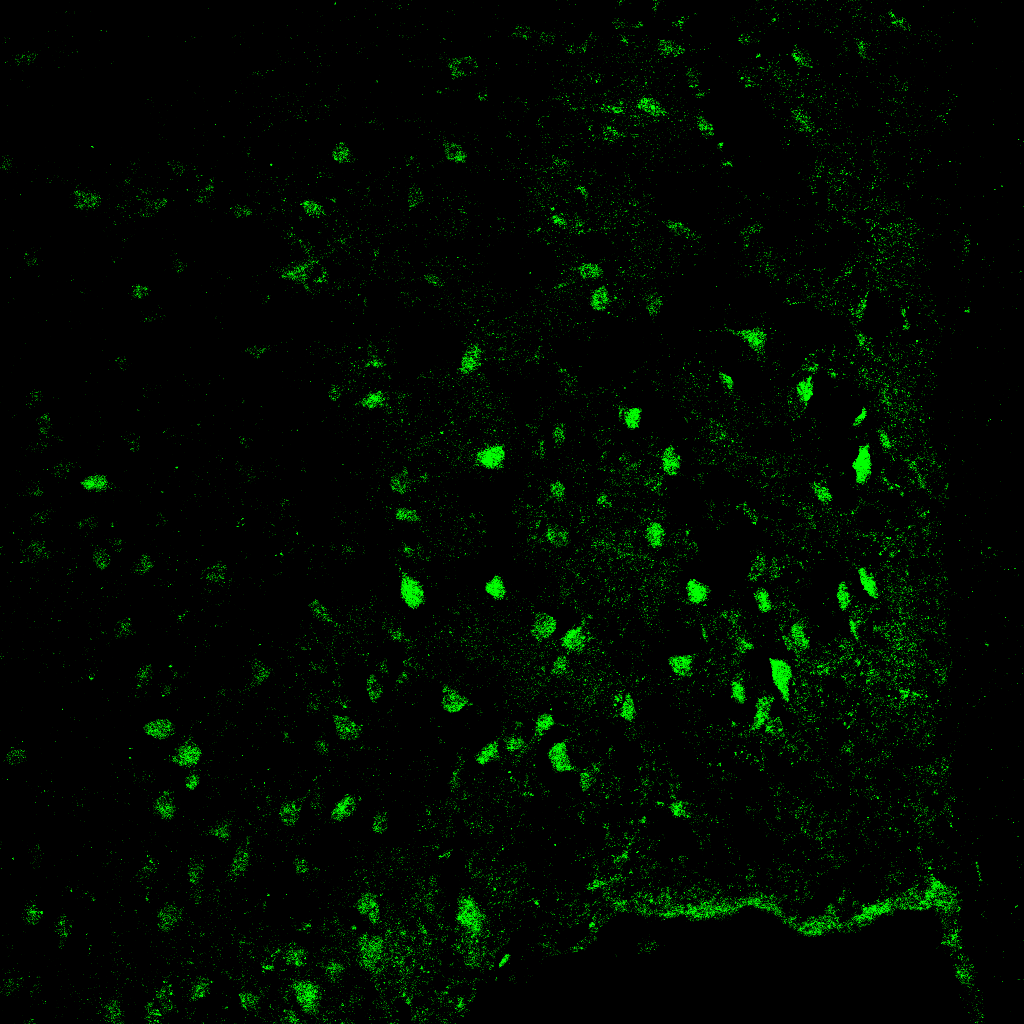

Supplement: Supplementary file 4 — Source data Fig. 2 [file 44319_2025_403_MOESM4_ESM.zip › Figure 2/2E/AAV-mCherry+Compound 21/c-Fos.tif]

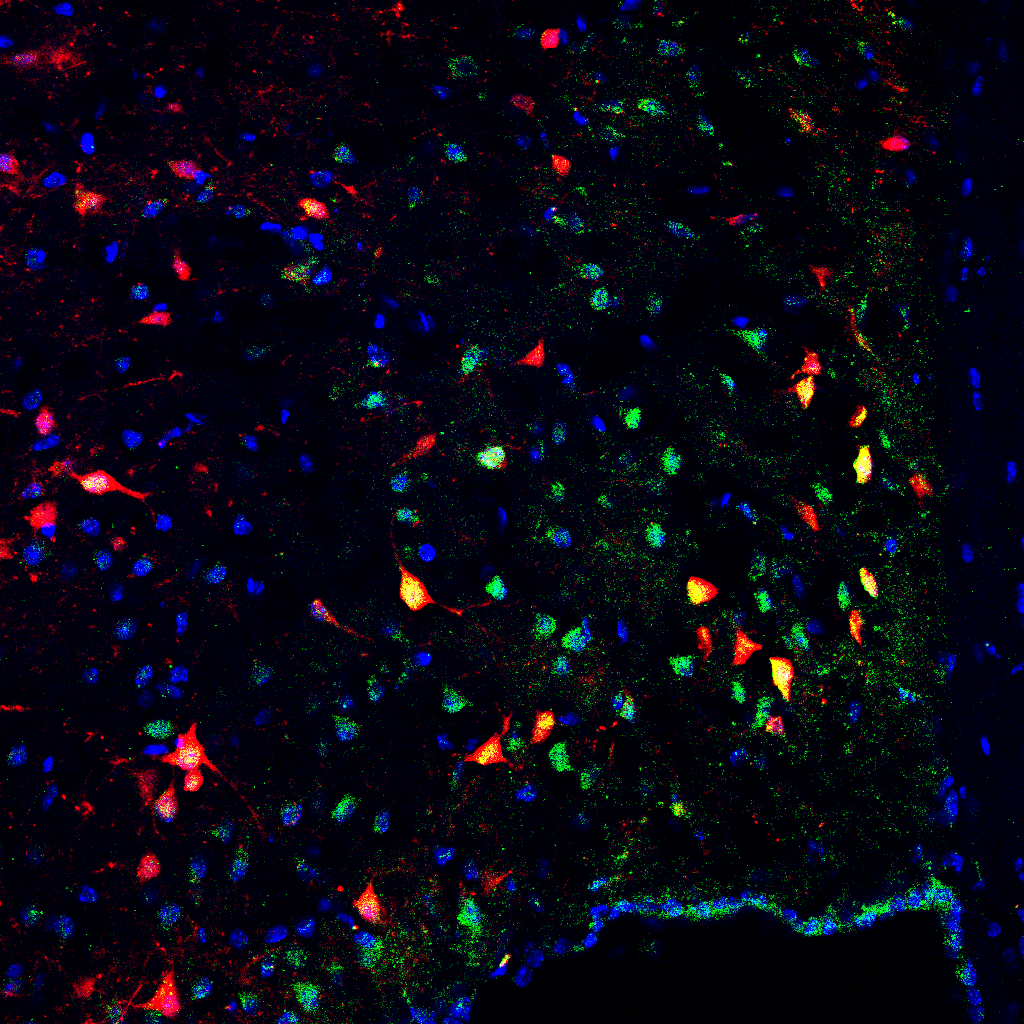

Supplement: Supplementary file 4 — Source data Fig. 2 [file 44319_2025_403_MOESM4_ESM.zip › Figure 2/2E/AAV-mCherry+Compound 21/overlay.tif]

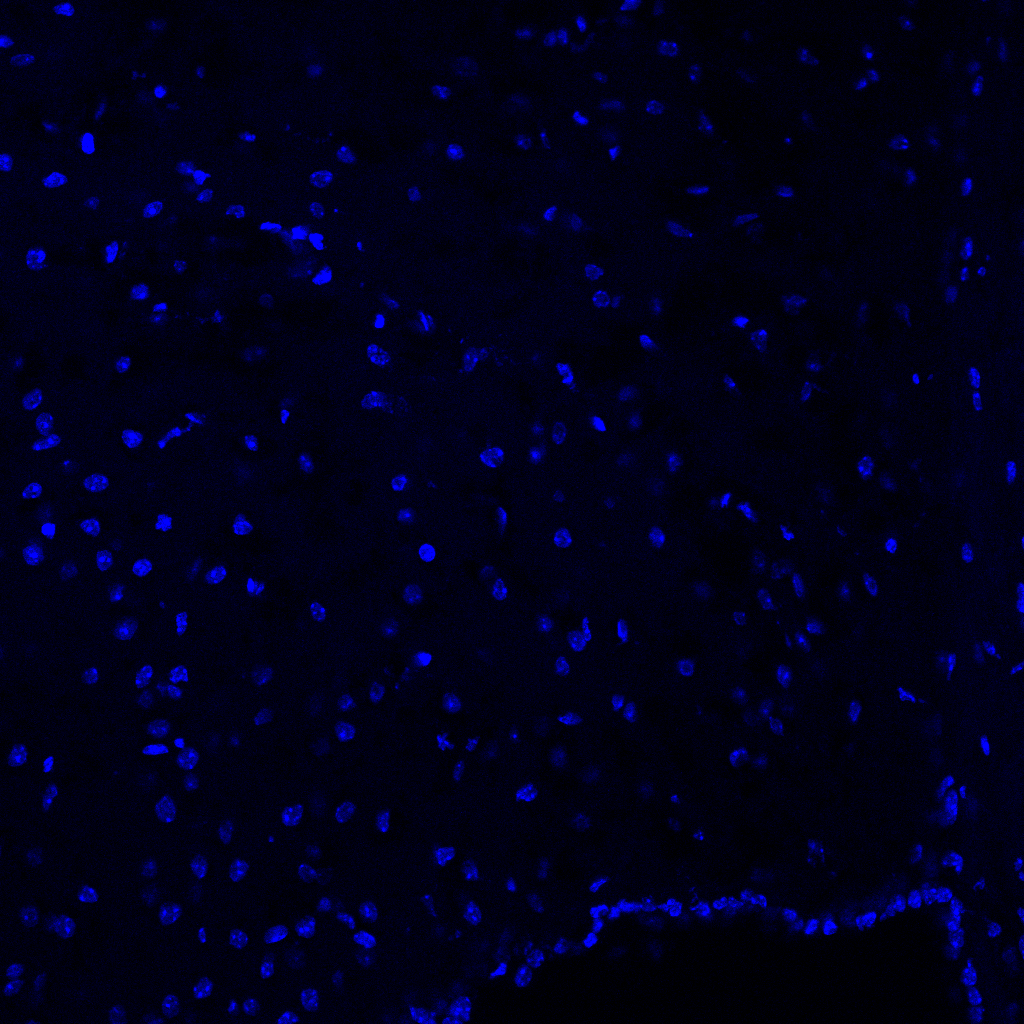

Supplement: Supplementary file 4 — Source data Fig. 2 [file 44319_2025_403_MOESM4_ESM.zip › Figure 2/2E/AAV-mCherry+Compound 21/Hoechst.tif]

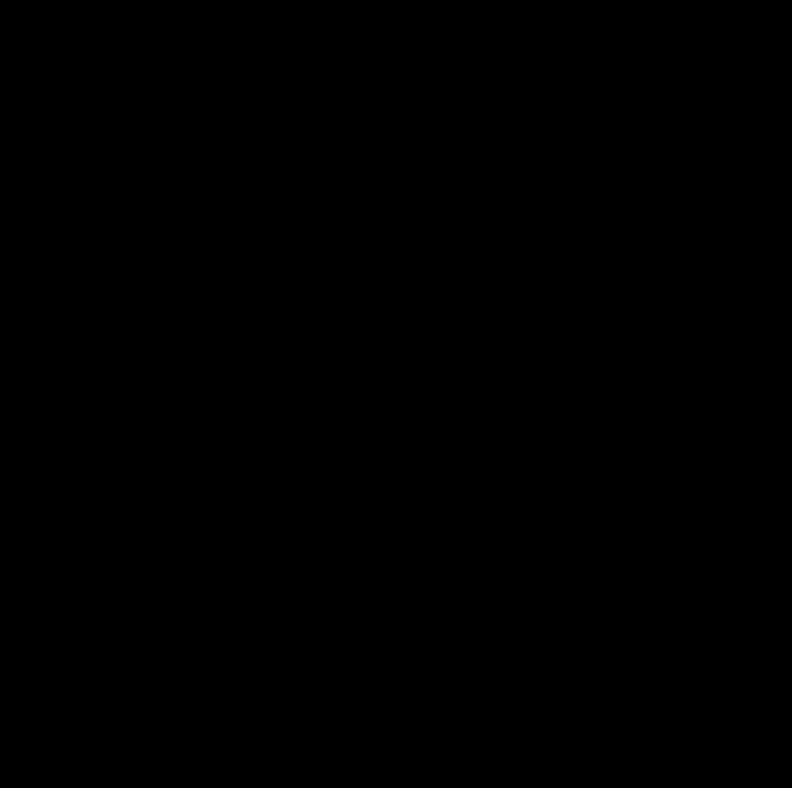

Supplement: Supplementary file 4 — Source data Fig. 2 [file 44319_2025_403_MOESM4_ESM.zip › Figure 2/2E/Control/mCherry.tif]

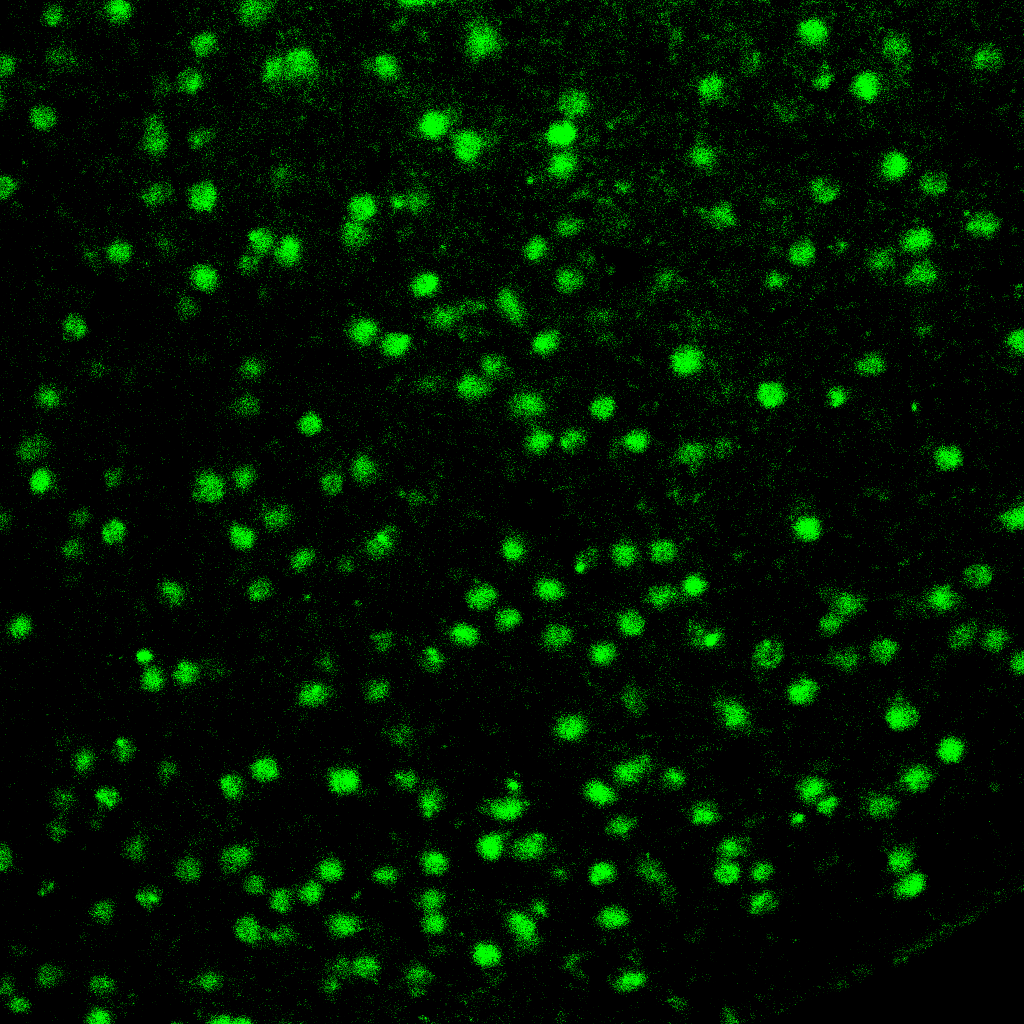

Supplement: Supplementary file 4 — Source data Fig. 2 [file 44319_2025_403_MOESM4_ESM.zip › Figure 2/2E/Control/c-Fos.tif]

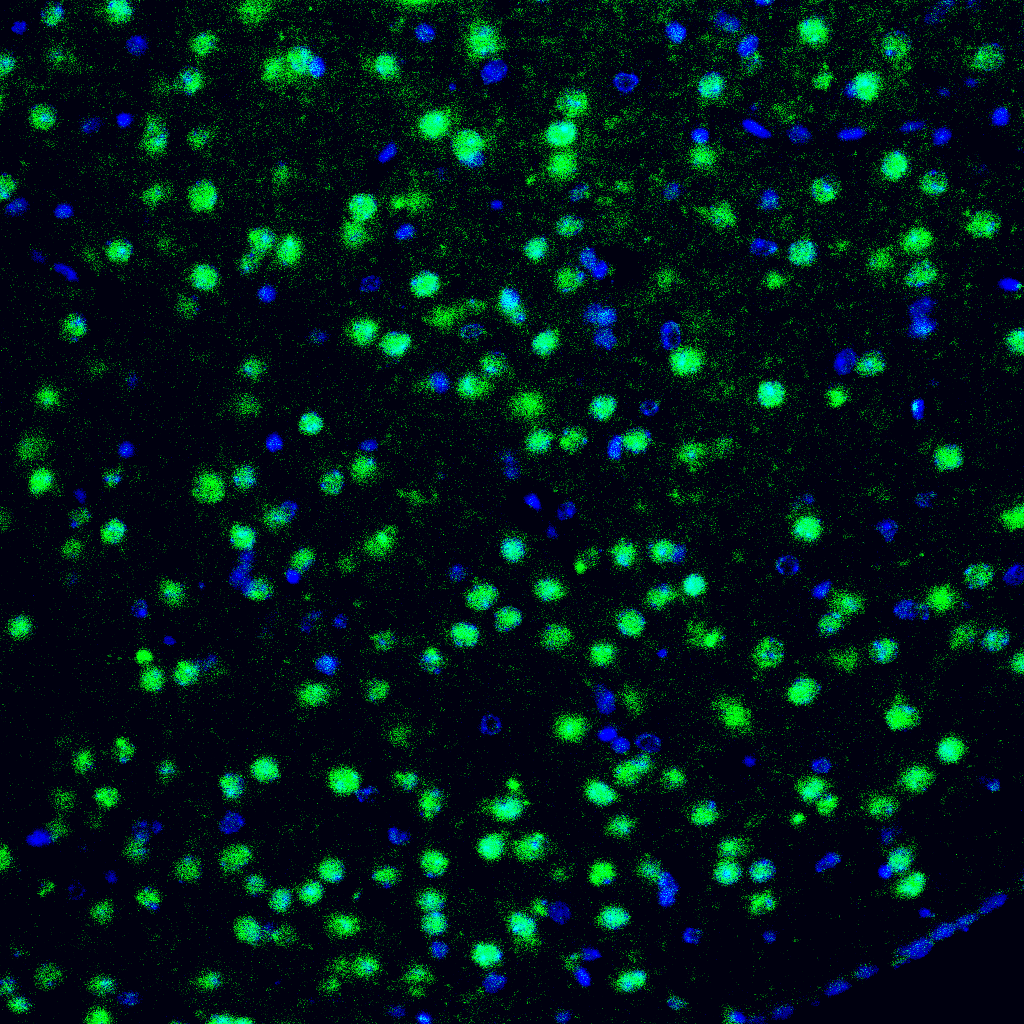

Supplement: Supplementary file 4 — Source data Fig. 2 [file 44319_2025_403_MOESM4_ESM.zip › Figure 2/2E/Control/overlay.tif]

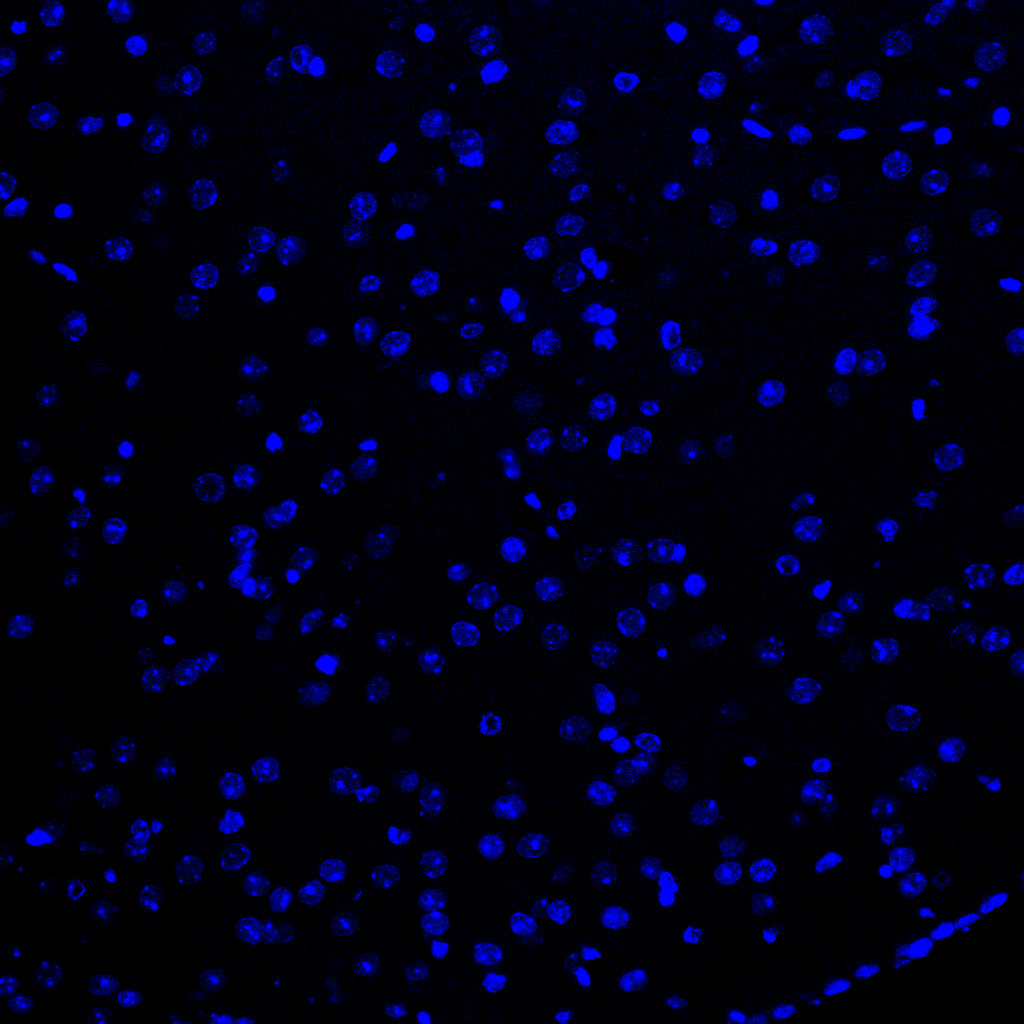

Supplement: Supplementary file 4 — Source data Fig. 2 [file 44319_2025_403_MOESM4_ESM.zip › Figure 2/2E/Control/Hoechst.tif]

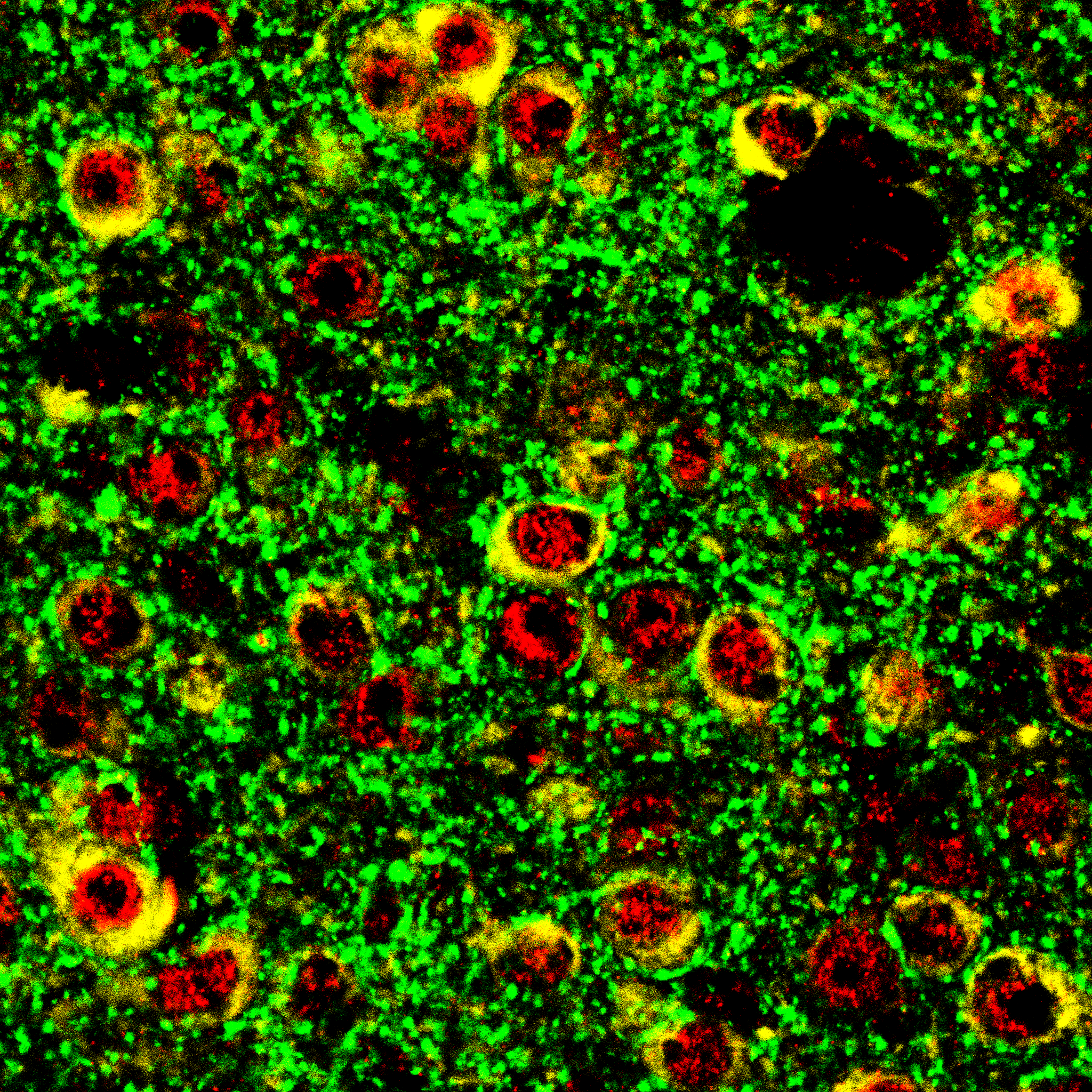

Supplement: Supplementary file 5 — Source data Fig. 3 [file 44319_2025_403_MOESM5_ESM.zip › Figure 3/3B/GAD 65 & 67/overlay 2.tif]

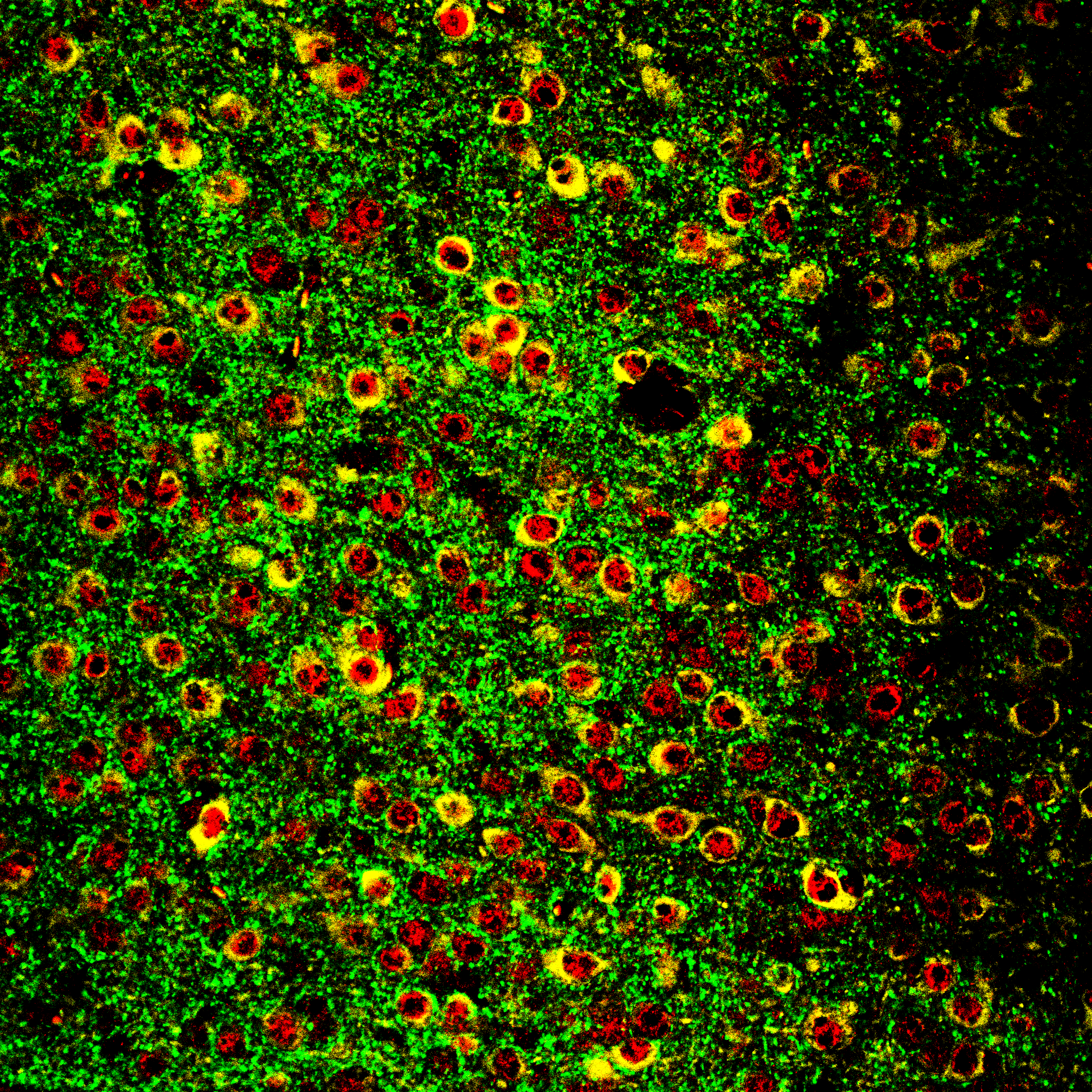

Supplement: Supplementary file 5 — Source data Fig. 3 [file 44319_2025_403_MOESM5_ESM.zip › Figure 3/3B/GAD 65 & 67/overlay 1.tif]

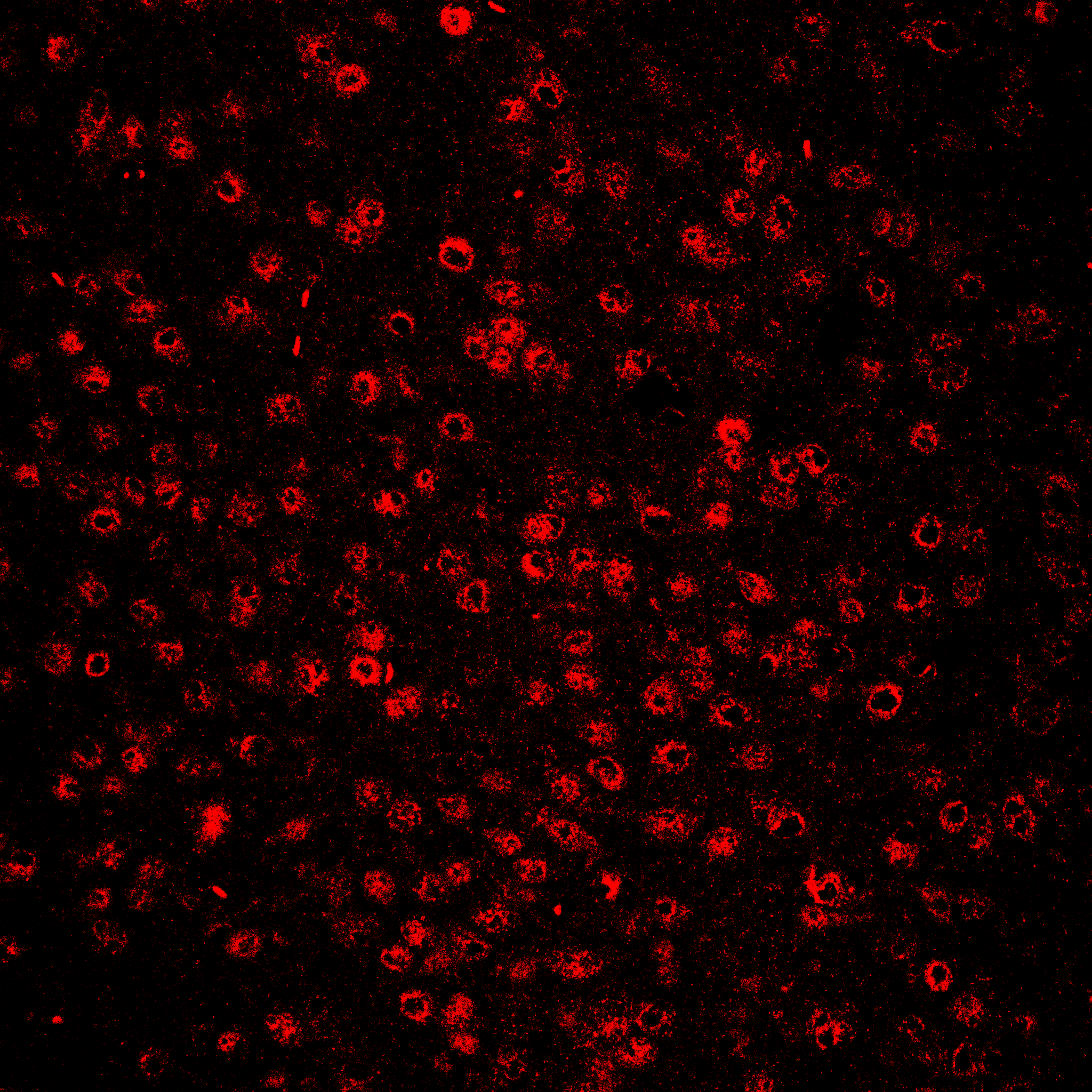

Supplement: Supplementary file 5 — Source data Fig. 3 [file 44319_2025_403_MOESM5_ESM.zip › Figure 3/3B/GAD 65 & 67/c-Fos.tif]

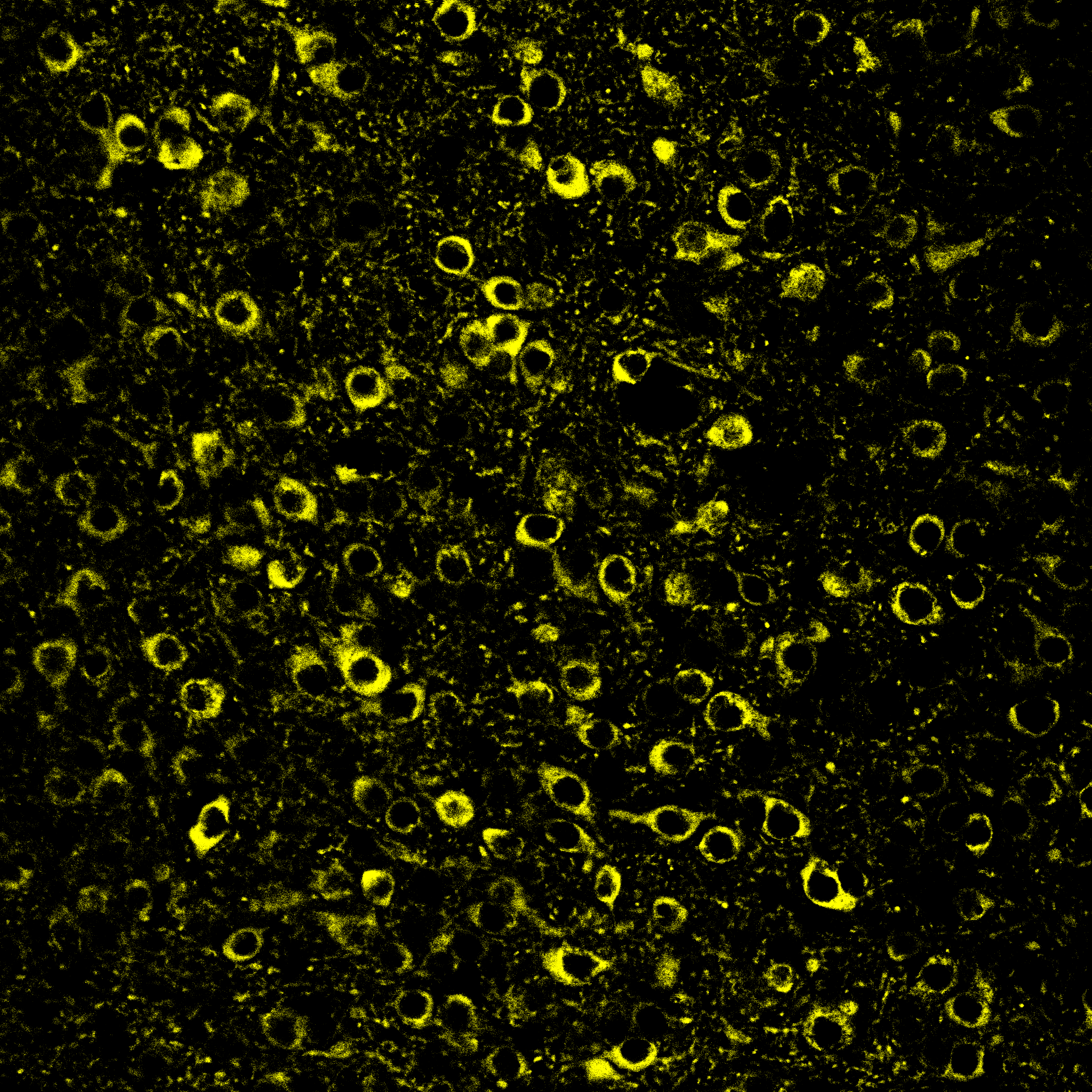

Supplement: Supplementary file 5 — Source data Fig. 3 [file 44319_2025_403_MOESM5_ESM.zip › Figure 3/3B/GAD 65 & 67/MAP2.tif]

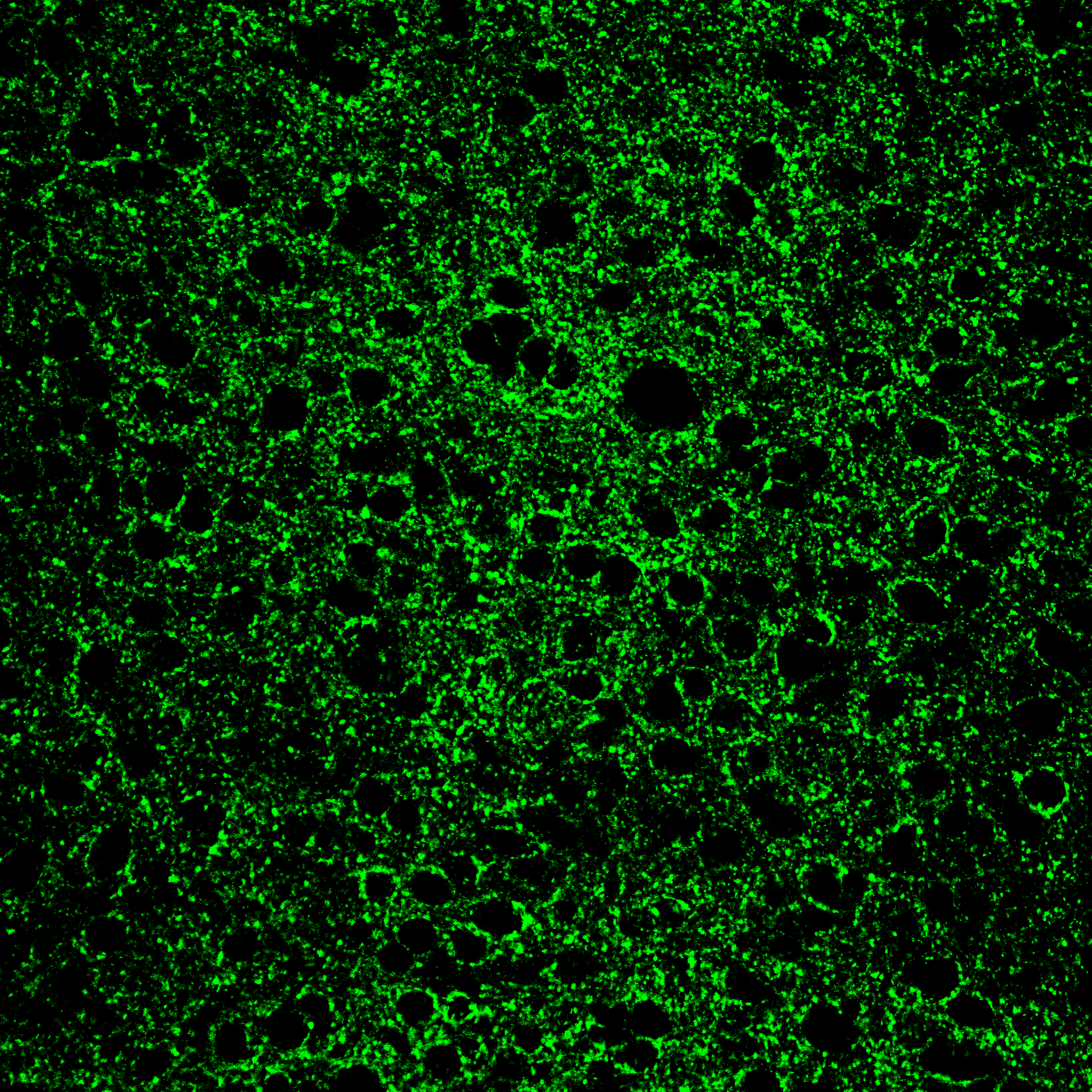

Supplement: Supplementary file 5 — Source data Fig. 3 [file 44319_2025_403_MOESM5_ESM.zip › Figure 3/3B/GAD 65 & 67/GAD.tif]

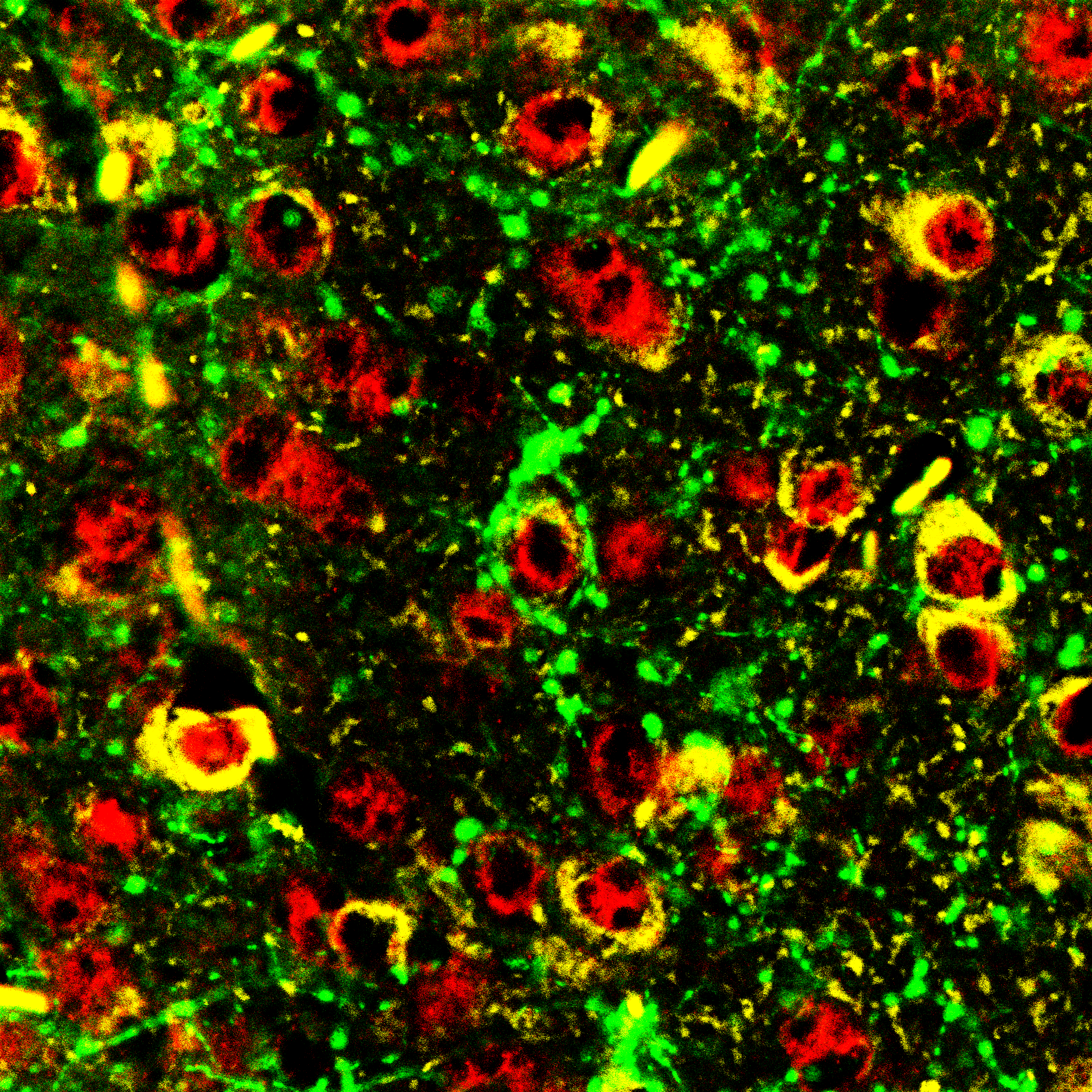

Supplement: Supplementary file 5 — Source data Fig. 3 [file 44319_2025_403_MOESM5_ESM.zip › Figure 3/3B/TH/overlay 2.tif]

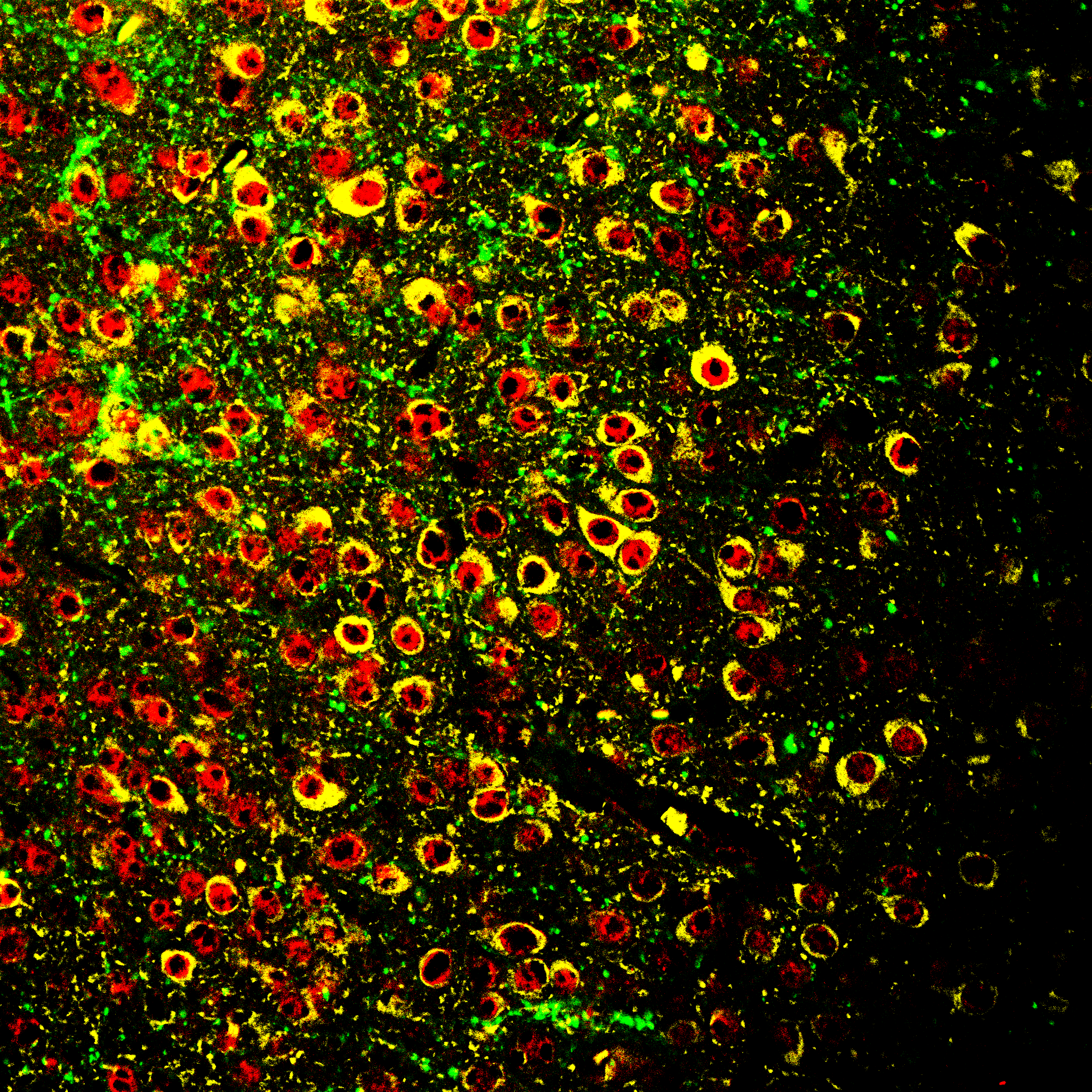

Supplement: Supplementary file 5 — Source data Fig. 3 [file 44319_2025_403_MOESM5_ESM.zip › Figure 3/3B/TH/overlay 1.tif]

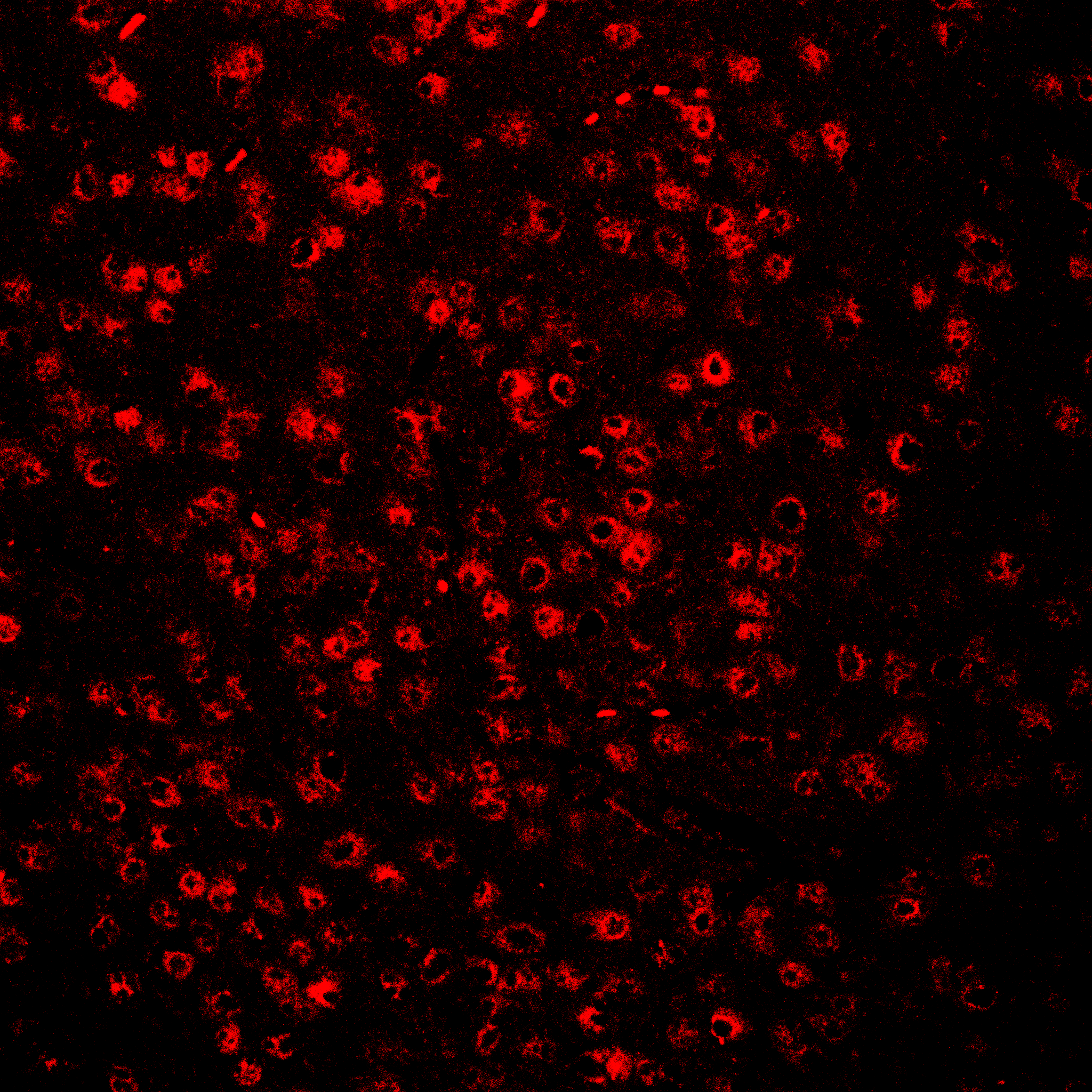

Supplement: Supplementary file 5 — Source data Fig. 3 [file 44319_2025_403_MOESM5_ESM.zip › Figure 3/3B/TH/c-Fos.tif]

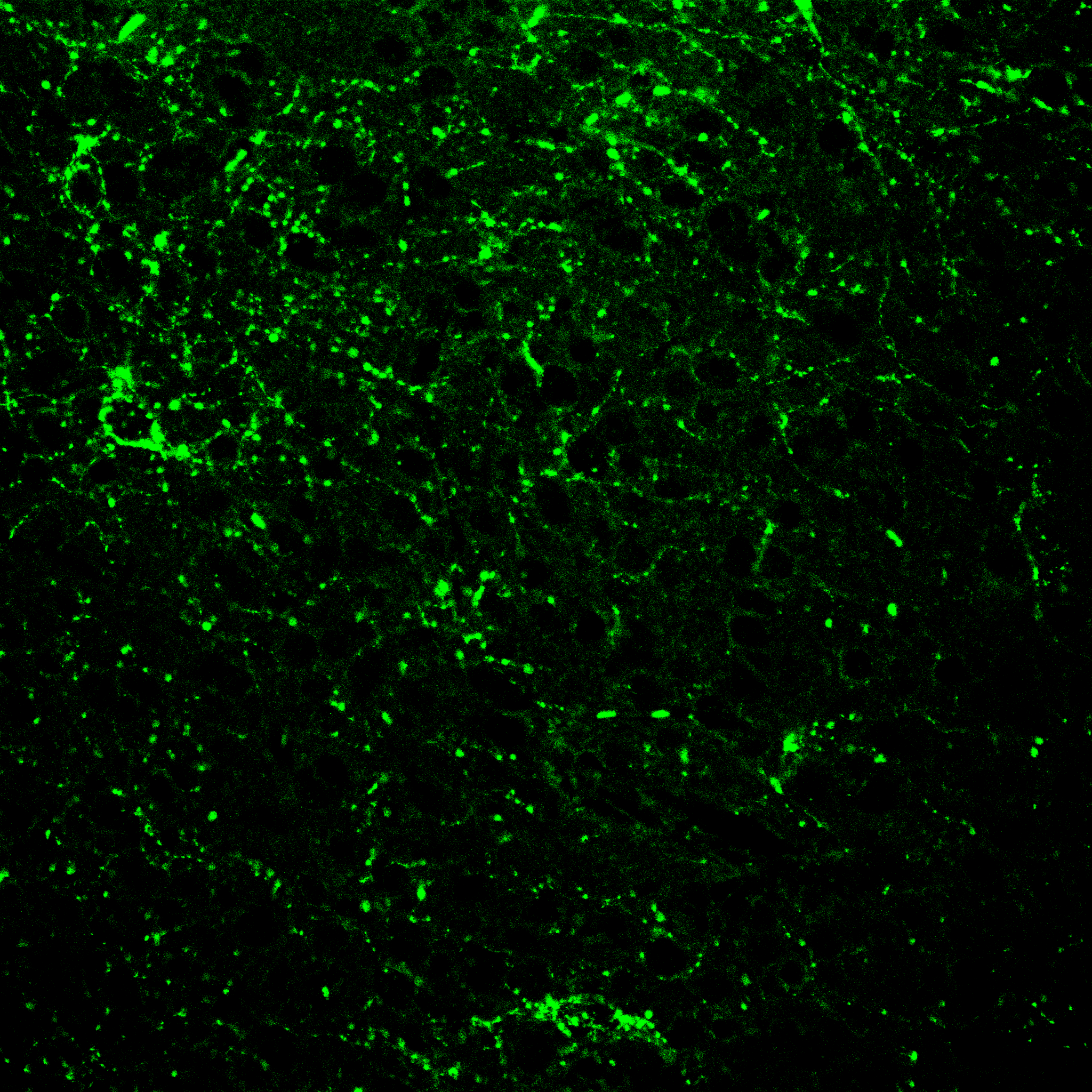

Supplement: Supplementary file 5 — Source data Fig. 3 [file 44319_2025_403_MOESM5_ESM.zip › Figure 3/3B/TH/TH.tif]

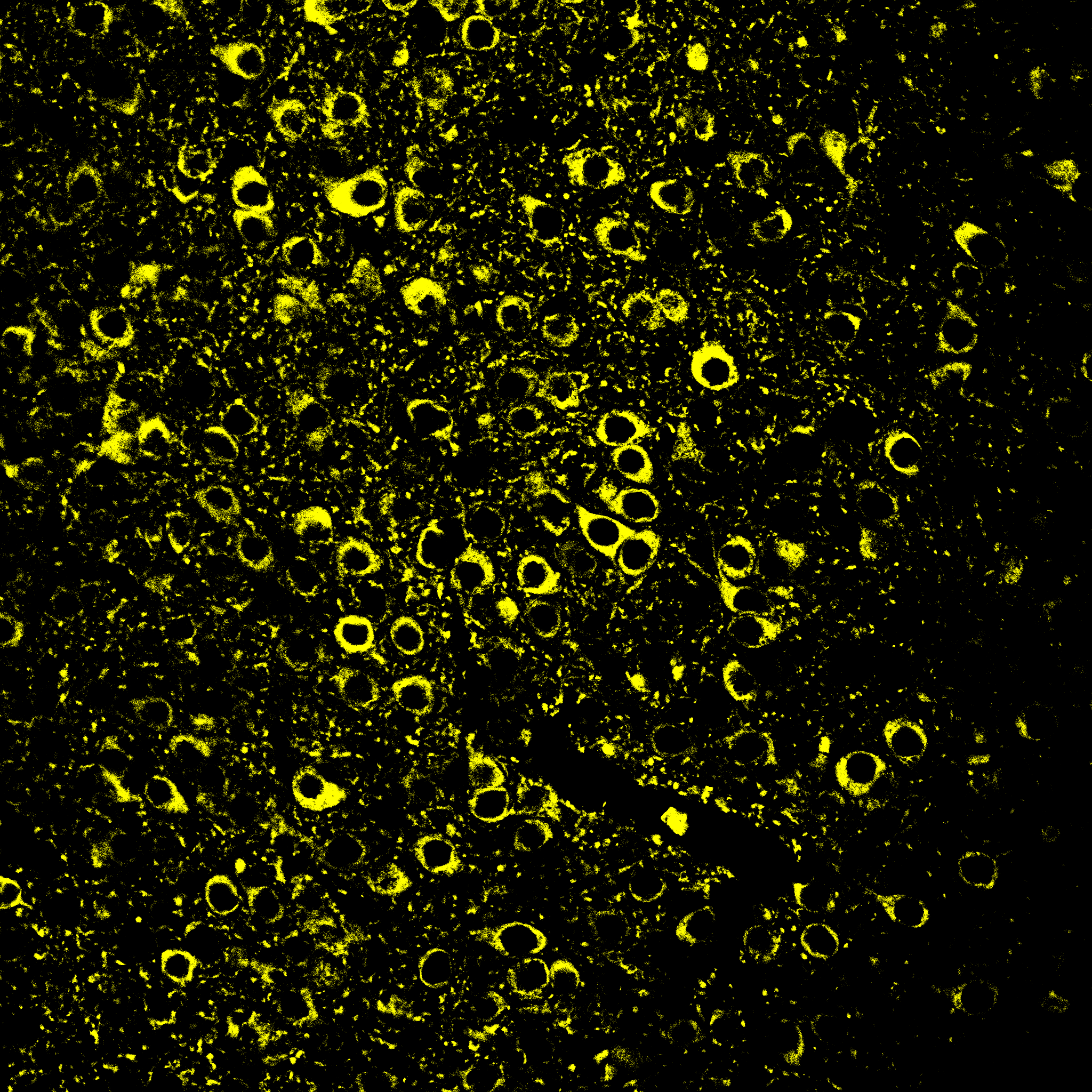

Supplement: Supplementary file 5 — Source data Fig. 3 [file 44319_2025_403_MOESM5_ESM.zip › Figure 3/3B/TH/MAP2.tif]

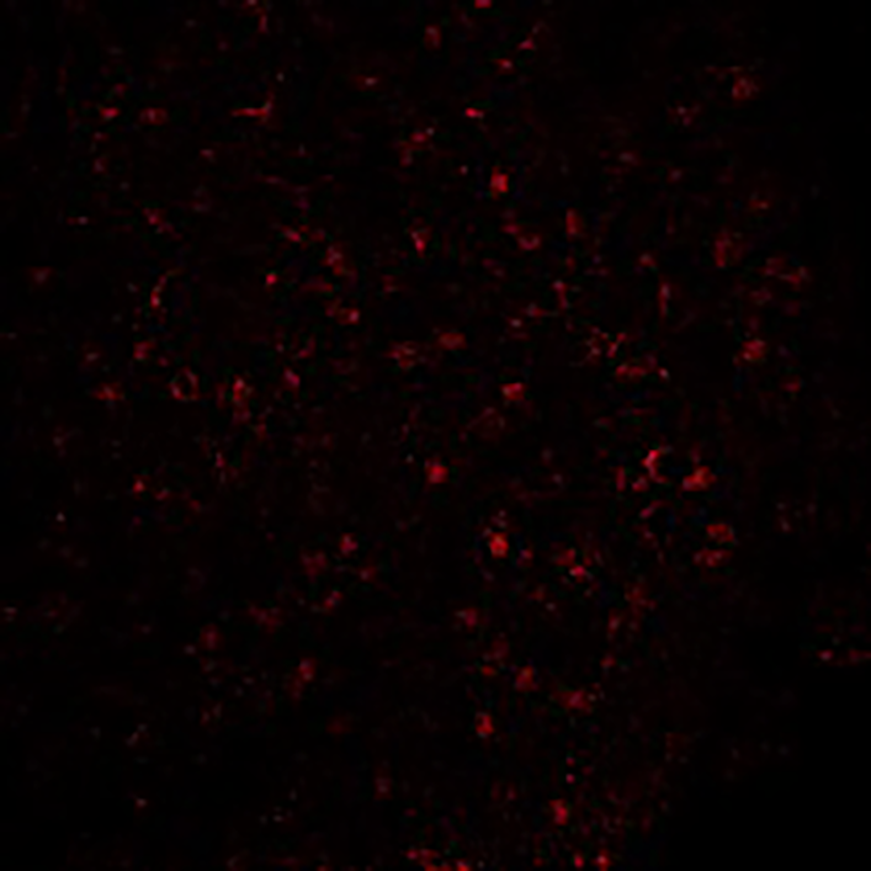

Supplement: Supplementary file 5 — Source data Fig. 3 [file 44319_2025_403_MOESM5_ESM.zip › Figure 3/3D/L-AMPT/c-Fos.tif]

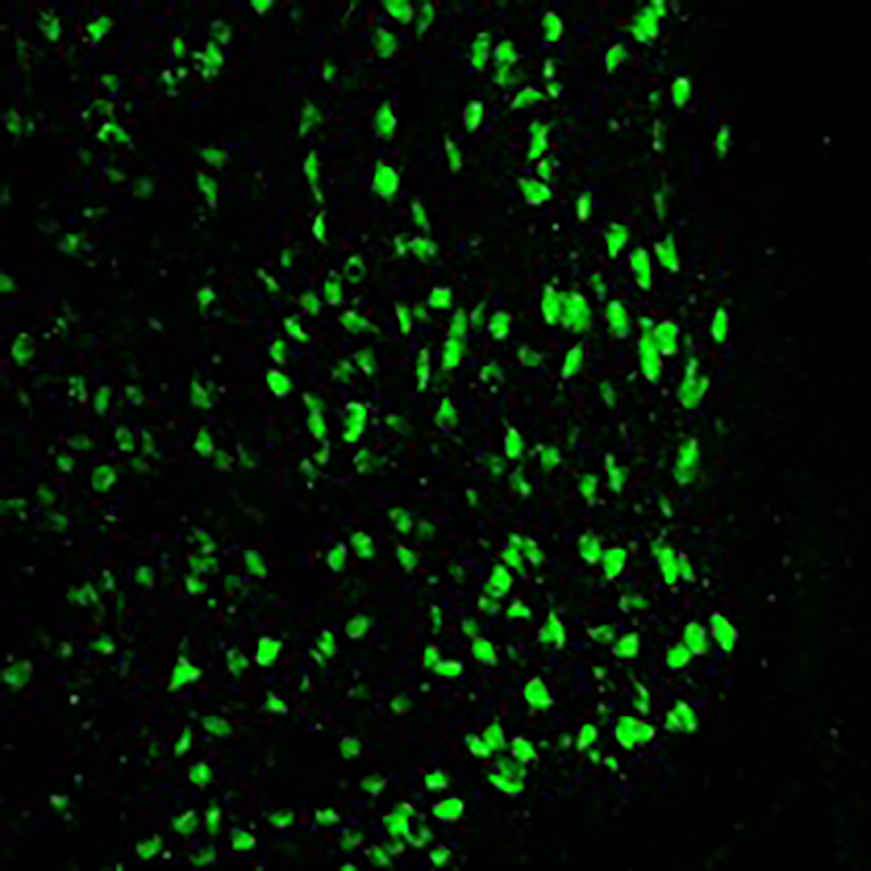

Supplement: Supplementary file 5 — Source data Fig. 3 [file 44319_2025_403_MOESM5_ESM.zip › Figure 3/3D/L-AMPT/NeuN.tif]

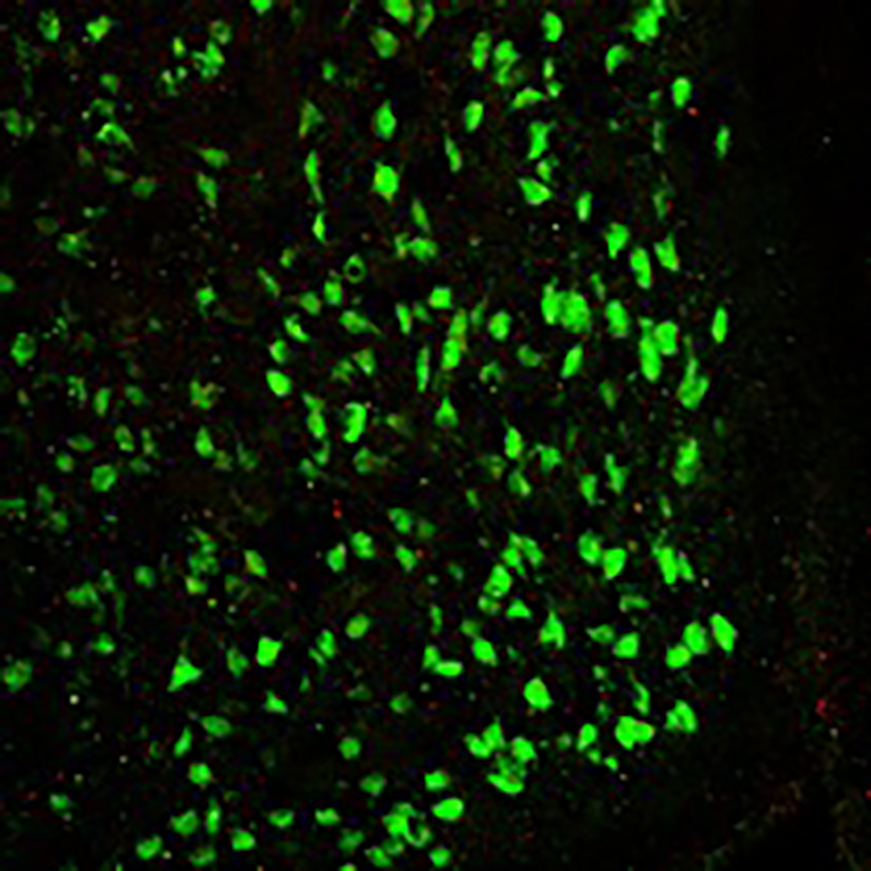

Supplement: Supplementary file 5 — Source data Fig. 3 [file 44319_2025_403_MOESM5_ESM.zip › Figure 3/3D/L-AMPT/overlay.tif]

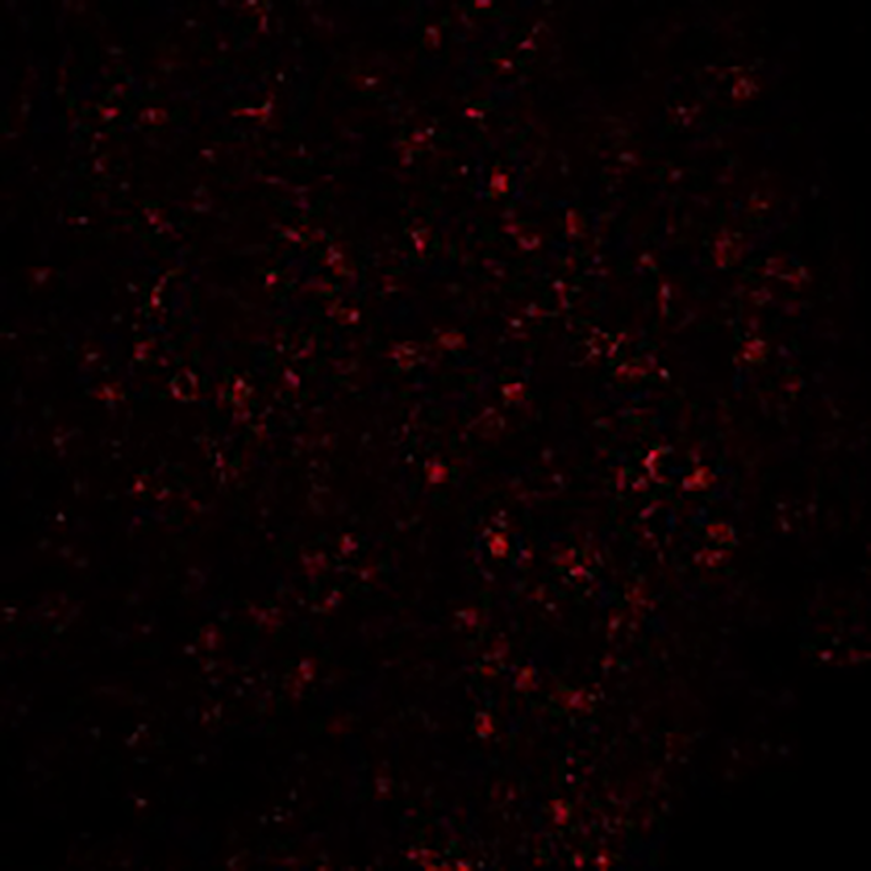

Supplement: Supplementary file 5 — Source data Fig. 3 [file 44319_2025_403_MOESM5_ESM.zip › Figure 3/3D/DLAG/c-Fos.tif]

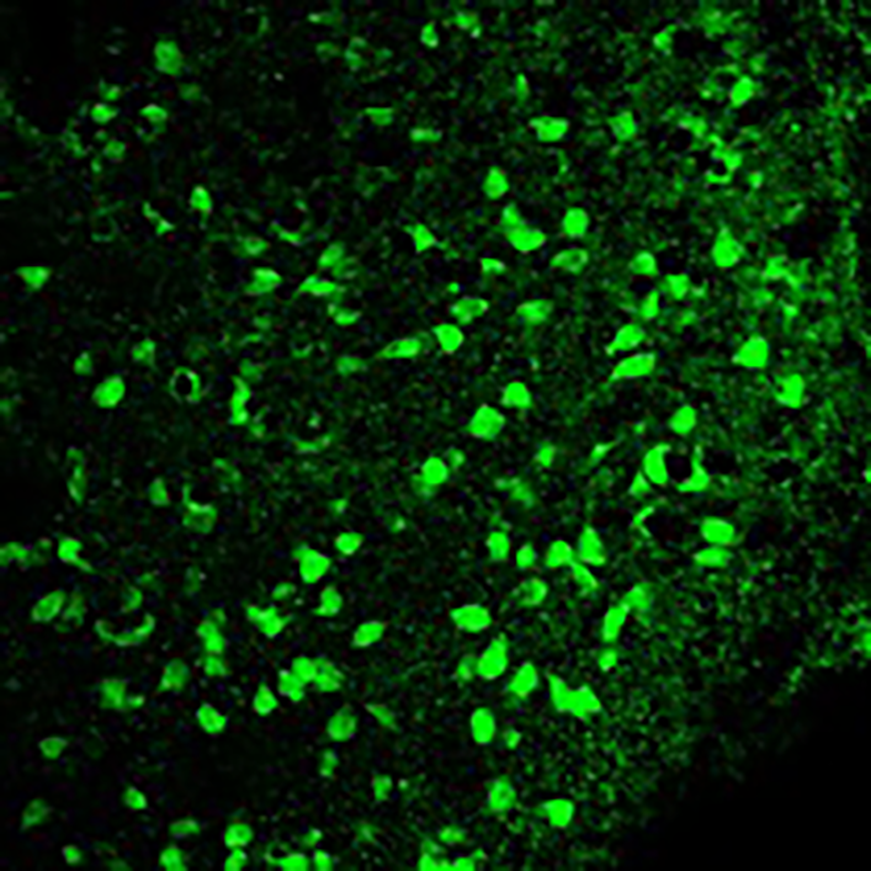

Supplement: Supplementary file 5 — Source data Fig. 3 [file 44319_2025_403_MOESM5_ESM.zip › Figure 3/3D/DLAG/NeuN.tif]

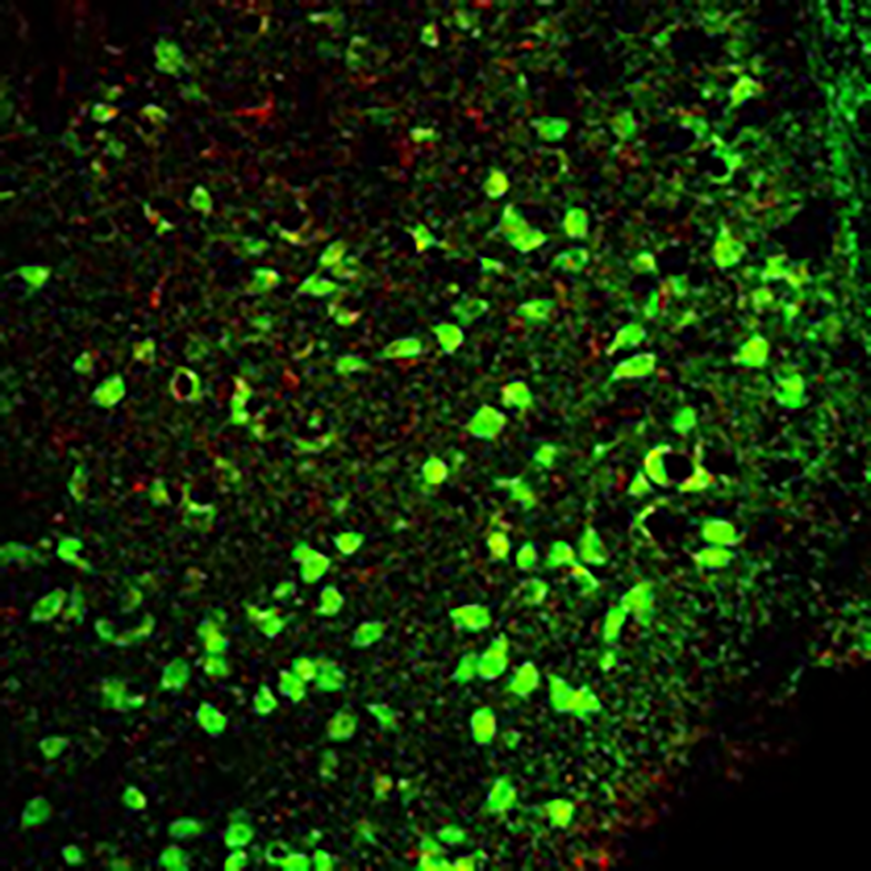

Supplement: Supplementary file 5 — Source data Fig. 3 [file 44319_2025_403_MOESM5_ESM.zip › Figure 3/3D/DLAG/overlay.tif]

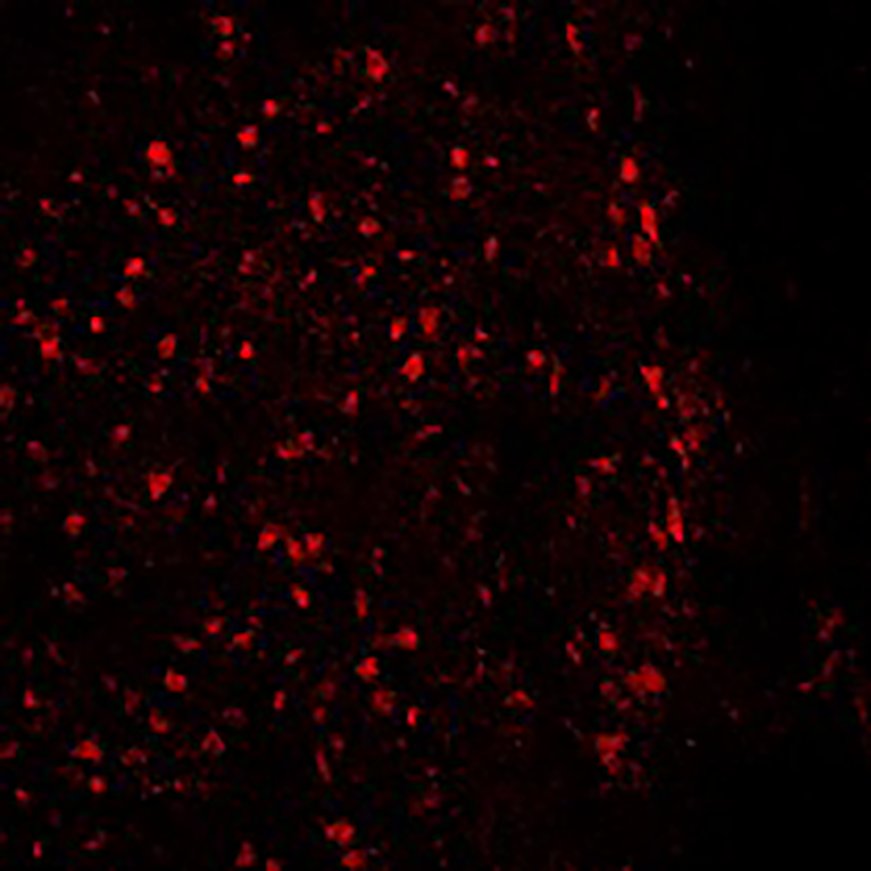

Supplement: Supplementary file 5 — Source data Fig. 3 [file 44319_2025_403_MOESM5_ESM.zip › Figure 3/3D/Vechicle/c-Fos.tif]

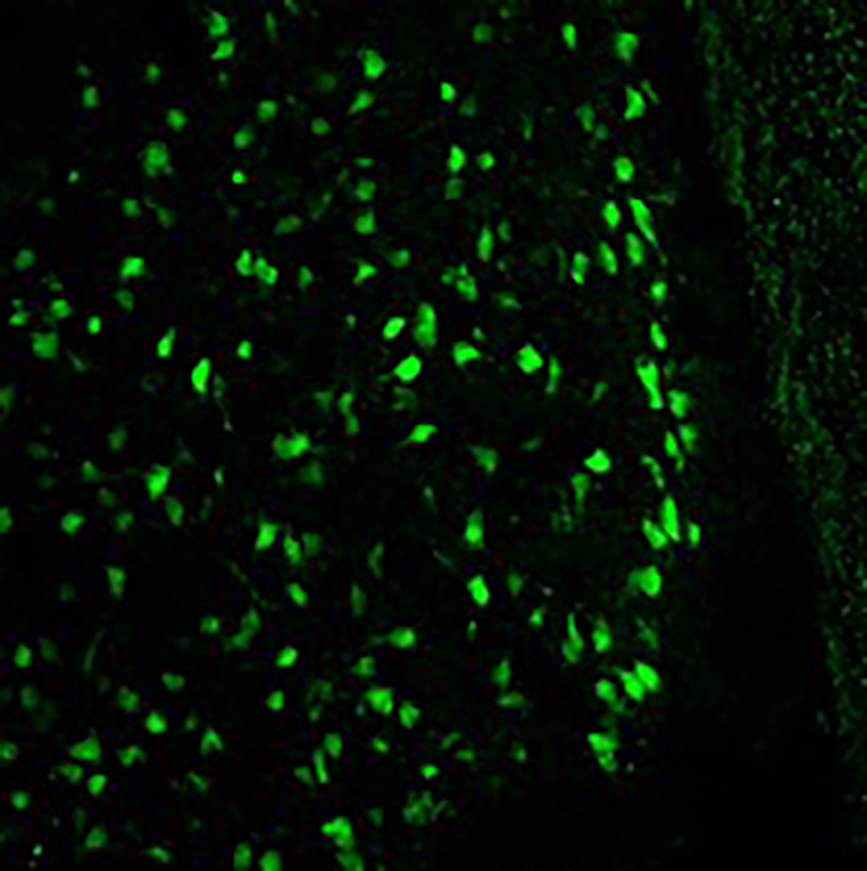

Supplement: Supplementary file 5 — Source data Fig. 3 [file 44319_2025_403_MOESM5_ESM.zip › Figure 3/3D/Vechicle/NeuN.tif]

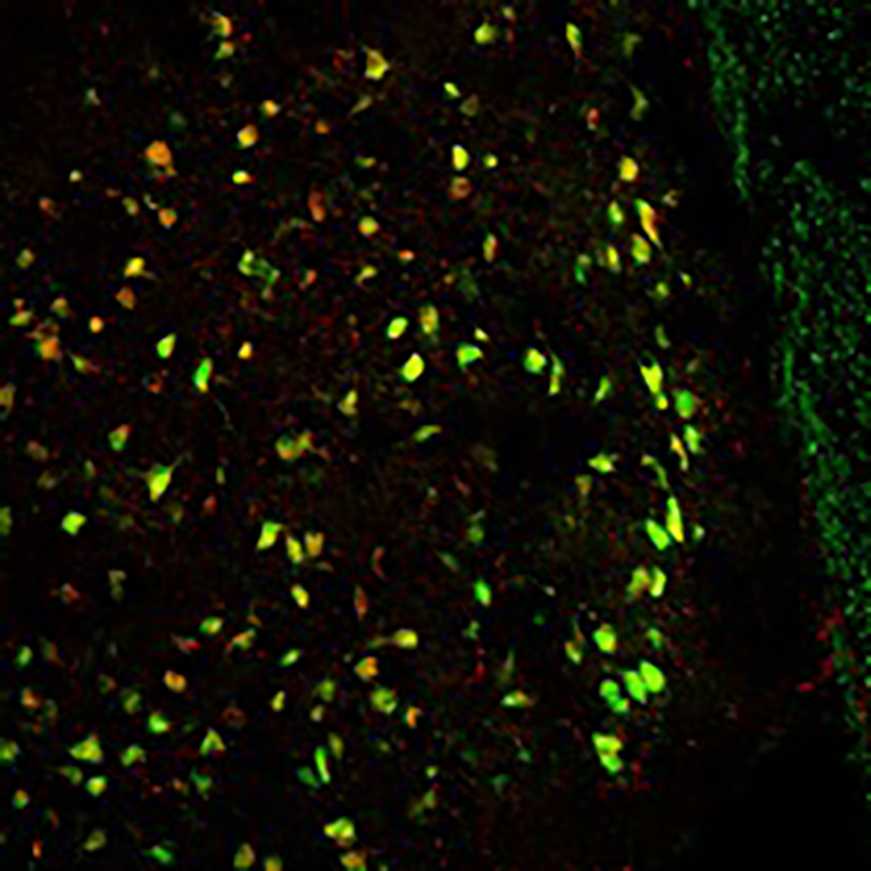

Supplement: Supplementary file 5 — Source data Fig. 3 [file 44319_2025_403_MOESM5_ESM.zip › Figure 3/3D/Vechicle/overlay.tif]

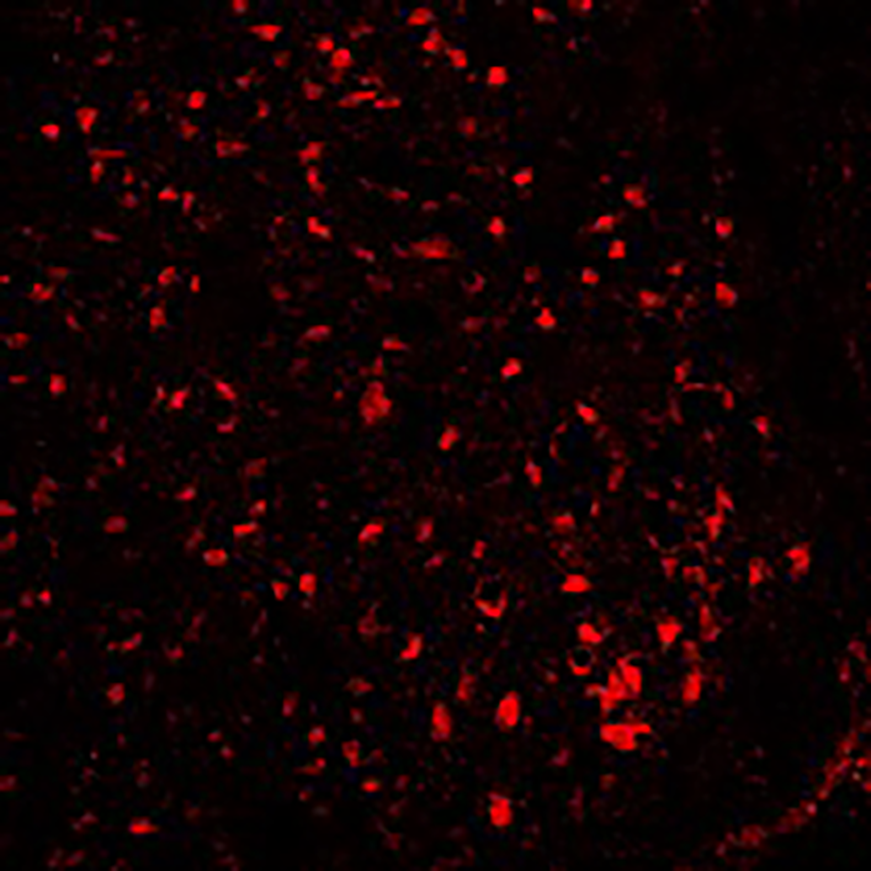

Supplement: Supplementary file 5 — Source data Fig. 3 [file 44319_2025_403_MOESM5_ESM.zip › Figure 3/3D/Control/c-Fos.tif]

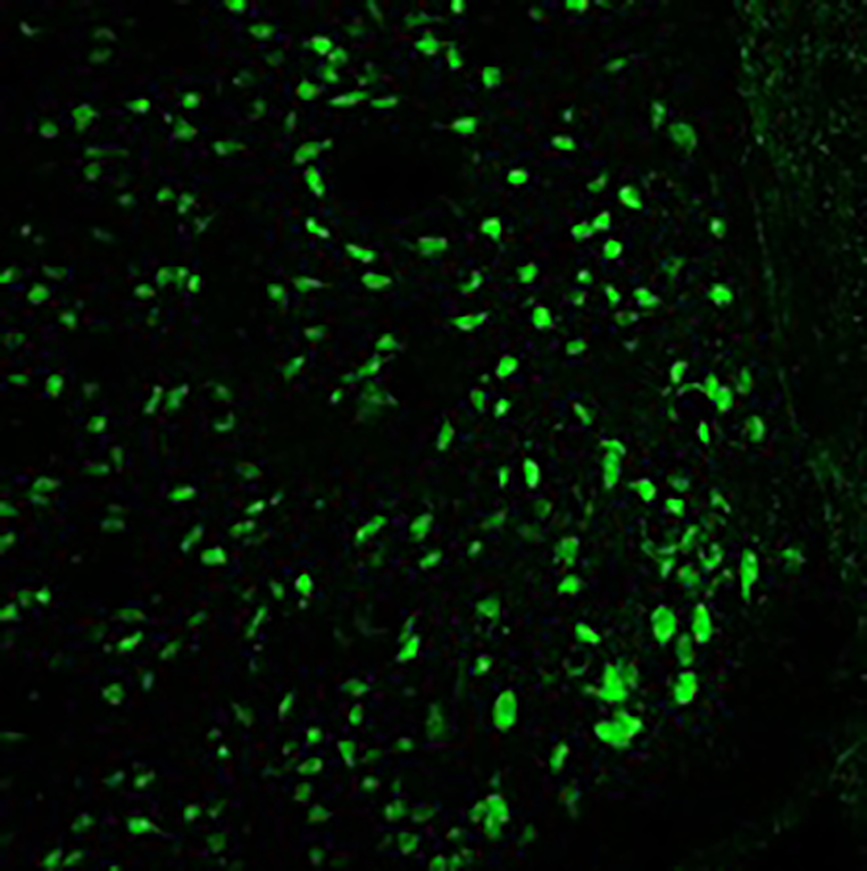

Supplement: Supplementary file 5 — Source data Fig. 3 [file 44319_2025_403_MOESM5_ESM.zip › Figure 3/3D/Control/NeuN.tif]

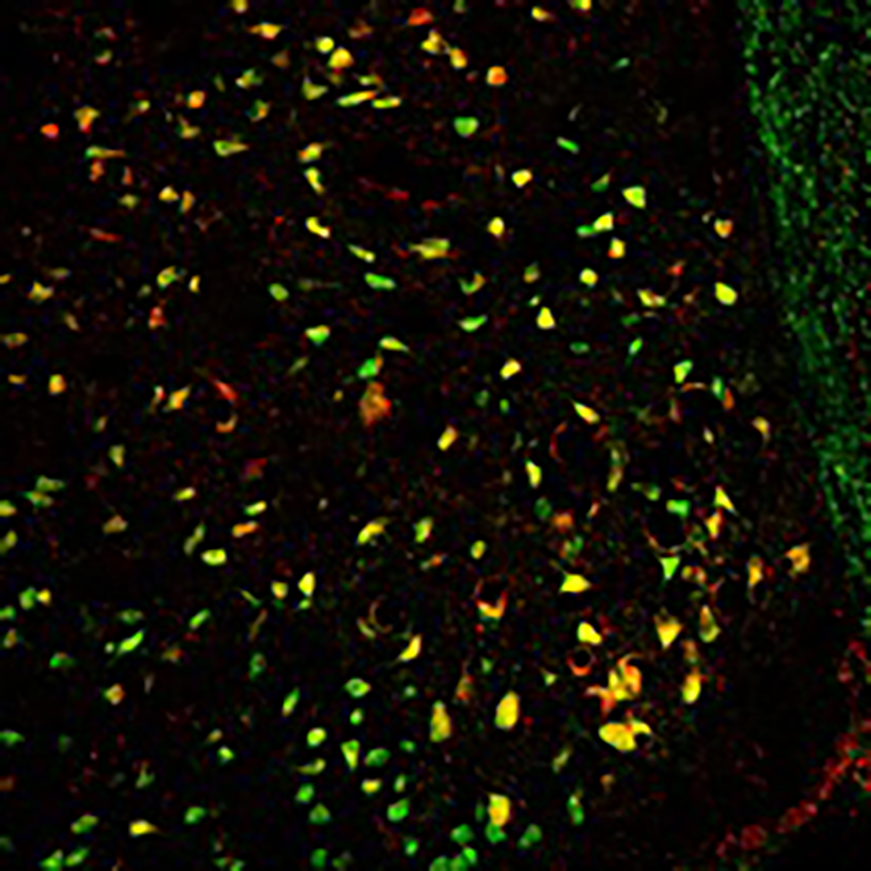

Supplement: Supplementary file 5 — Source data Fig. 3 [file 44319_2025_403_MOESM5_ESM.zip › Figure 3/3D/Control/overlay.tif]

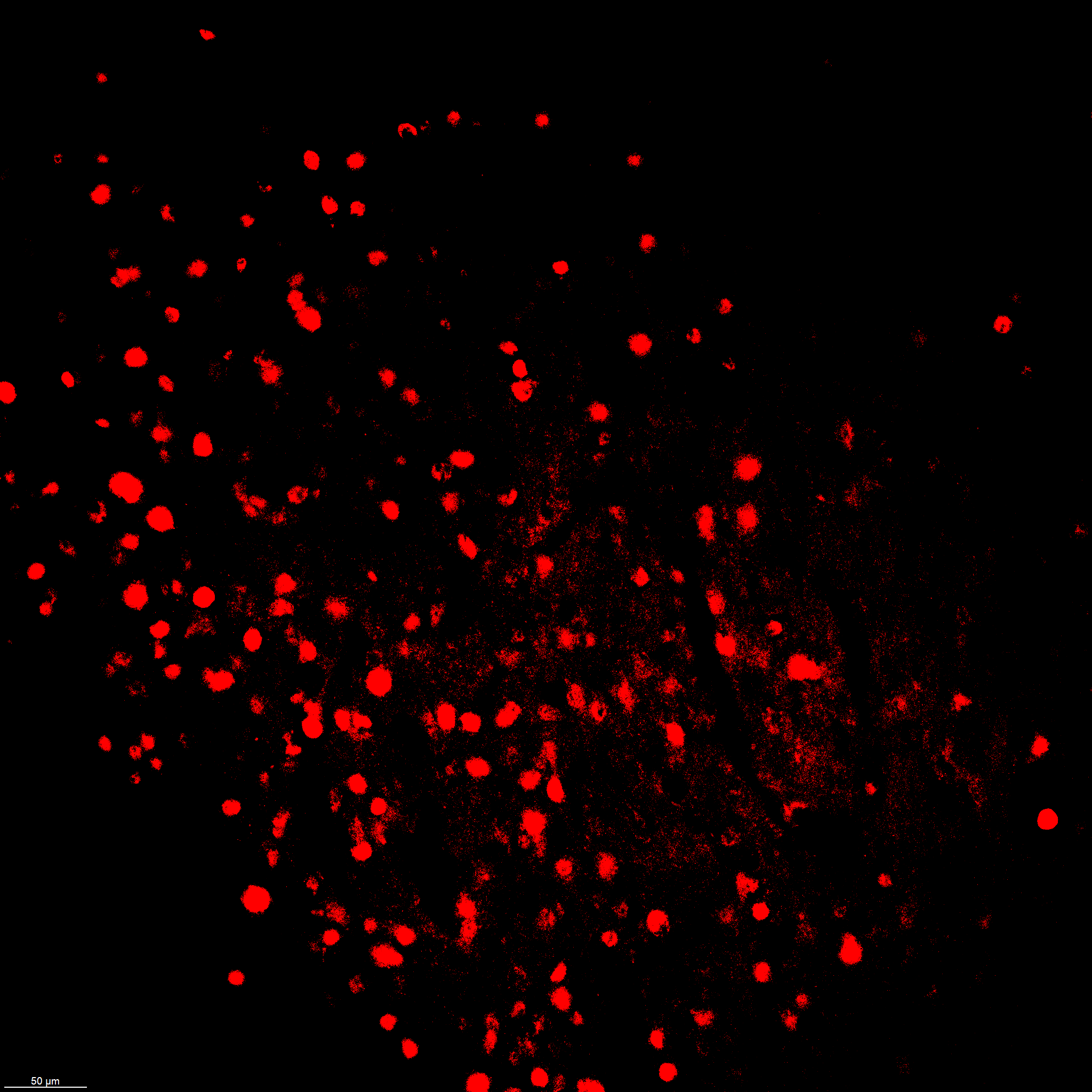

Supplement: Supplementary file 6 — Source data Fig. 4 [file 44319_2025_403_MOESM6_ESM.zip › Figure 4/4E/TH-V/c-fos.tif]

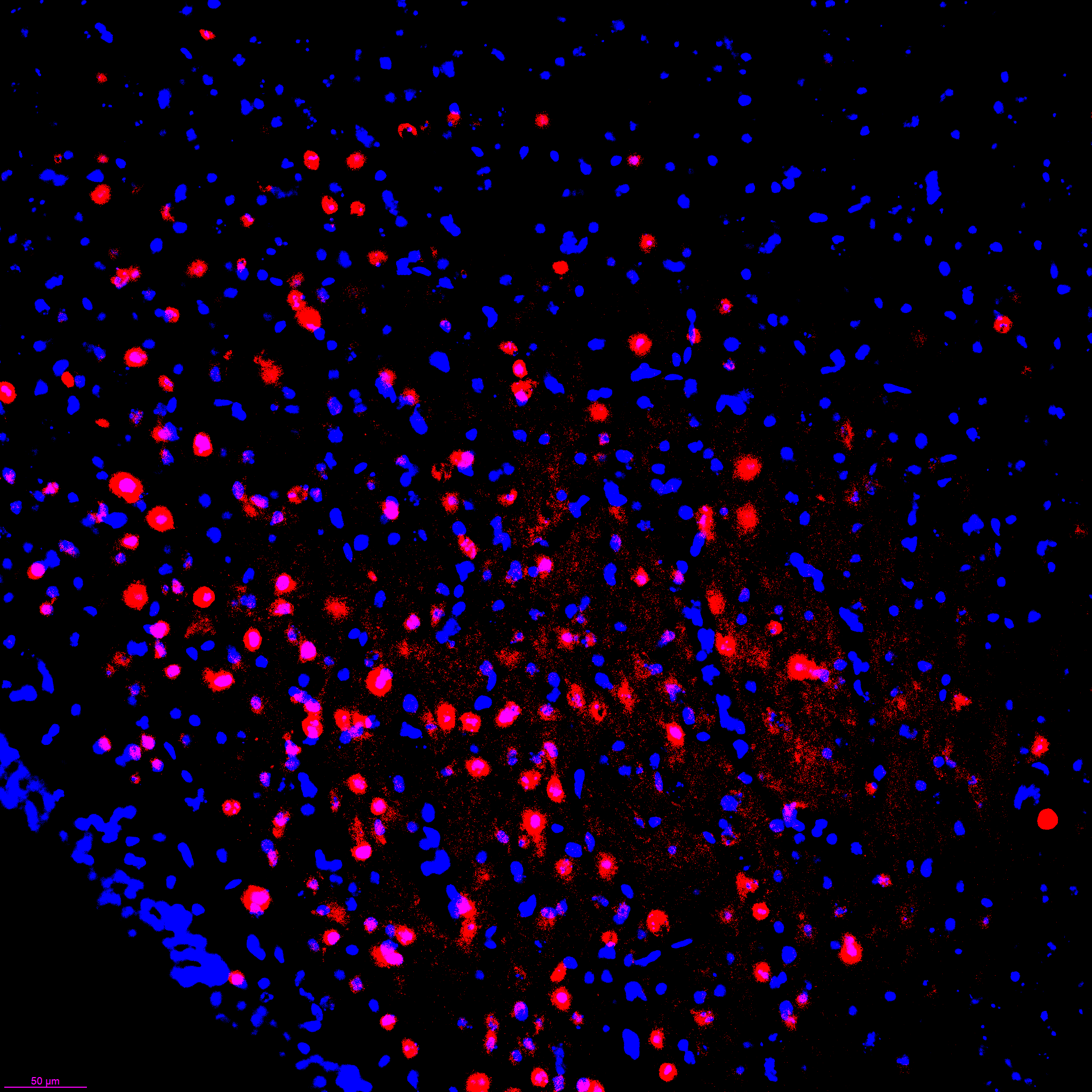

Supplement: Supplementary file 6 — Source data Fig. 4 [file 44319_2025_403_MOESM6_ESM.zip › Figure 4/4E/TH-V/overlay.tif]

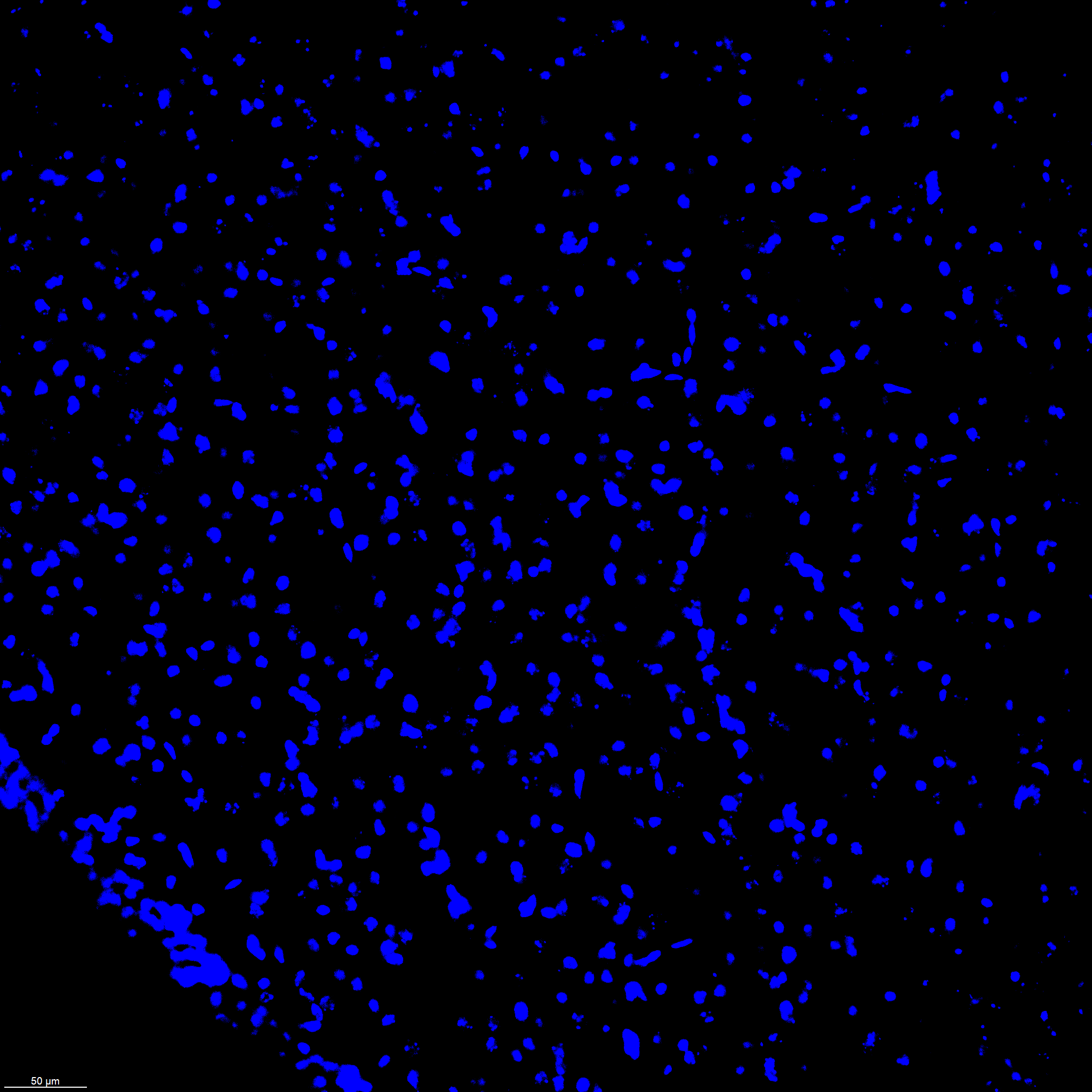

Supplement: Supplementary file 6 — Source data Fig. 4 [file 44319_2025_403_MOESM6_ESM.zip › Figure 4/4E/TH-V/Hoechst.tif]

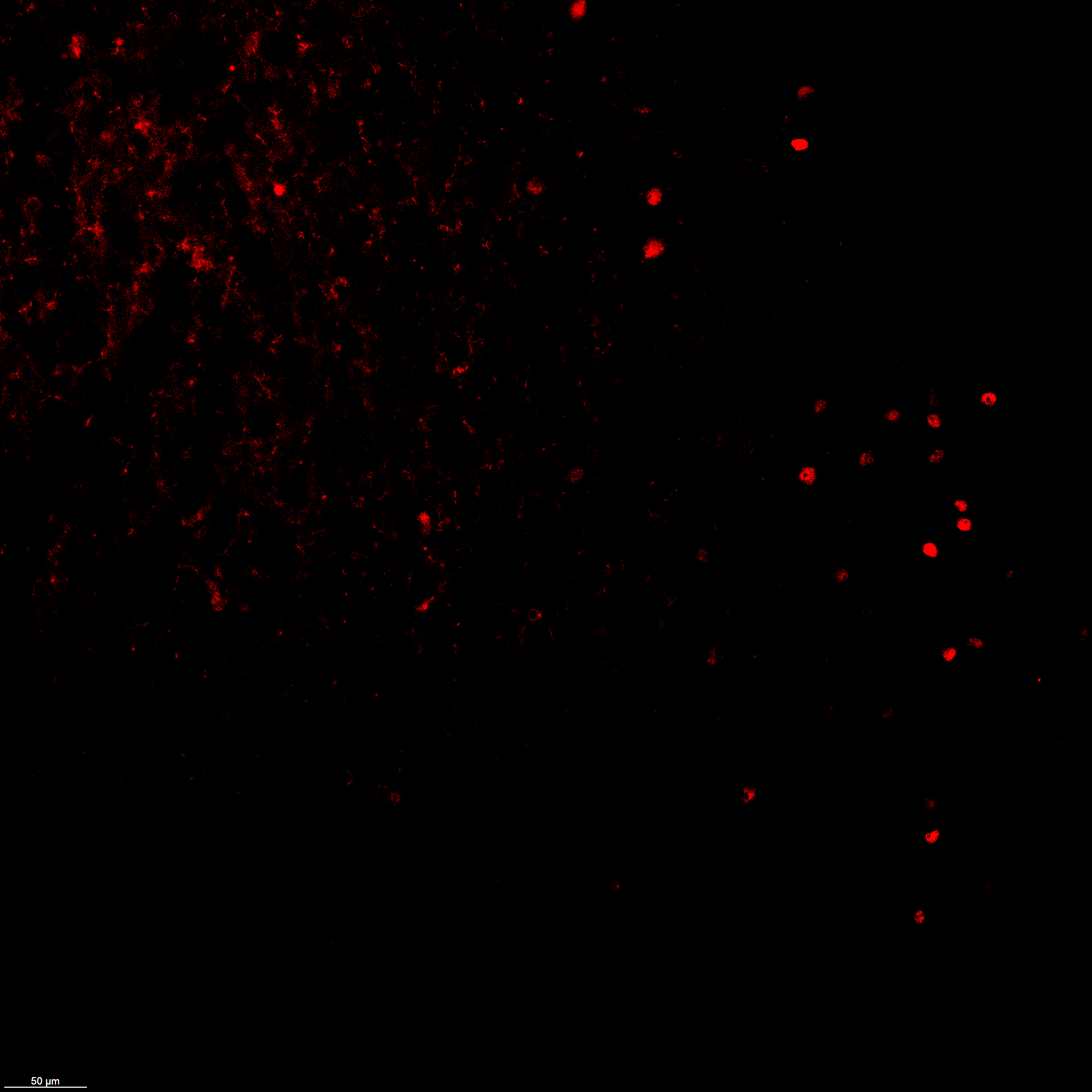

Supplement: Supplementary file 6 — Source data Fig. 4 [file 44319_2025_403_MOESM6_ESM.zip › Figure 4/4E/TH-C21/c-fos.tif]

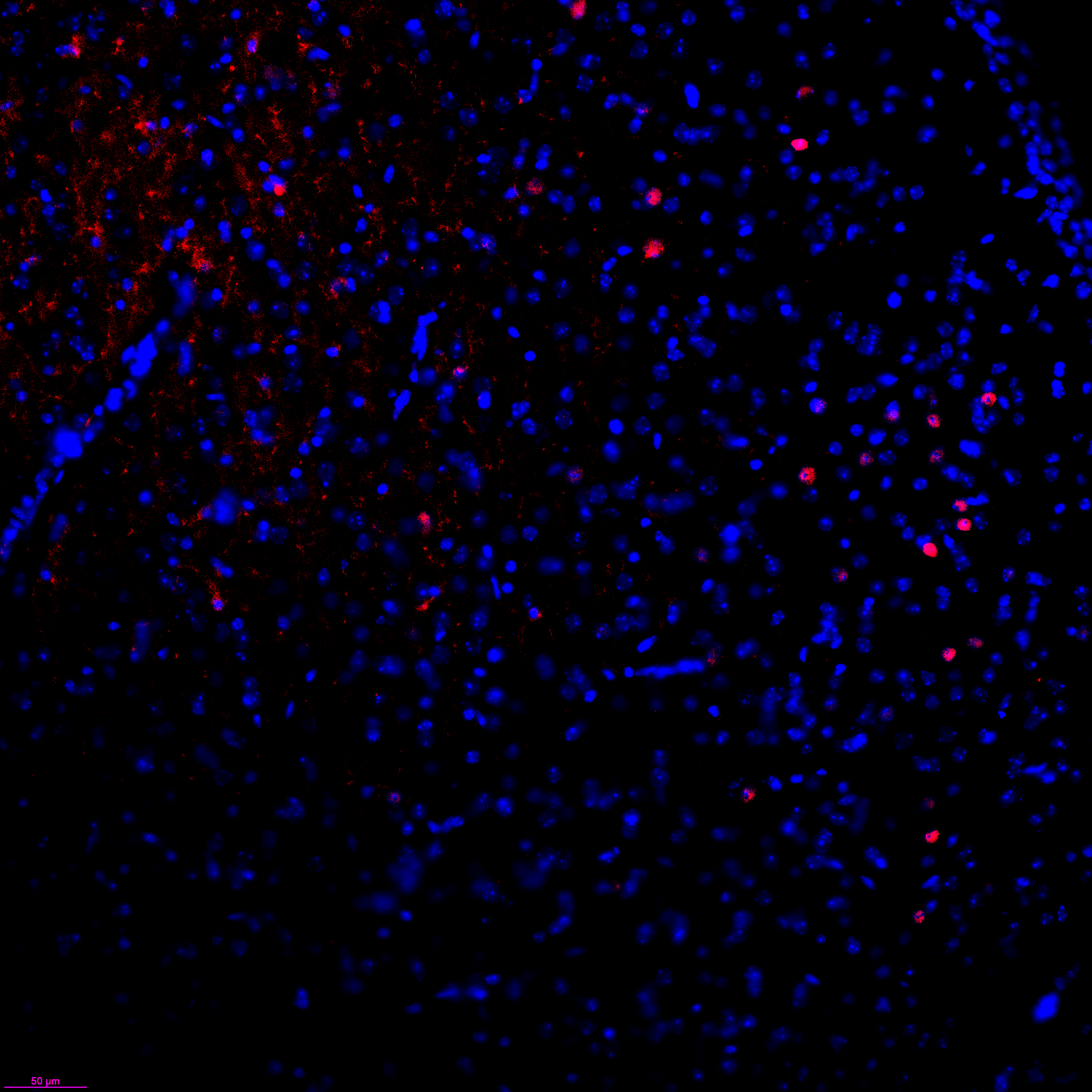

Supplement: Supplementary file 6 — Source data Fig. 4 [file 44319_2025_403_MOESM6_ESM.zip › Figure 4/4E/TH-C21/overlay.tif]

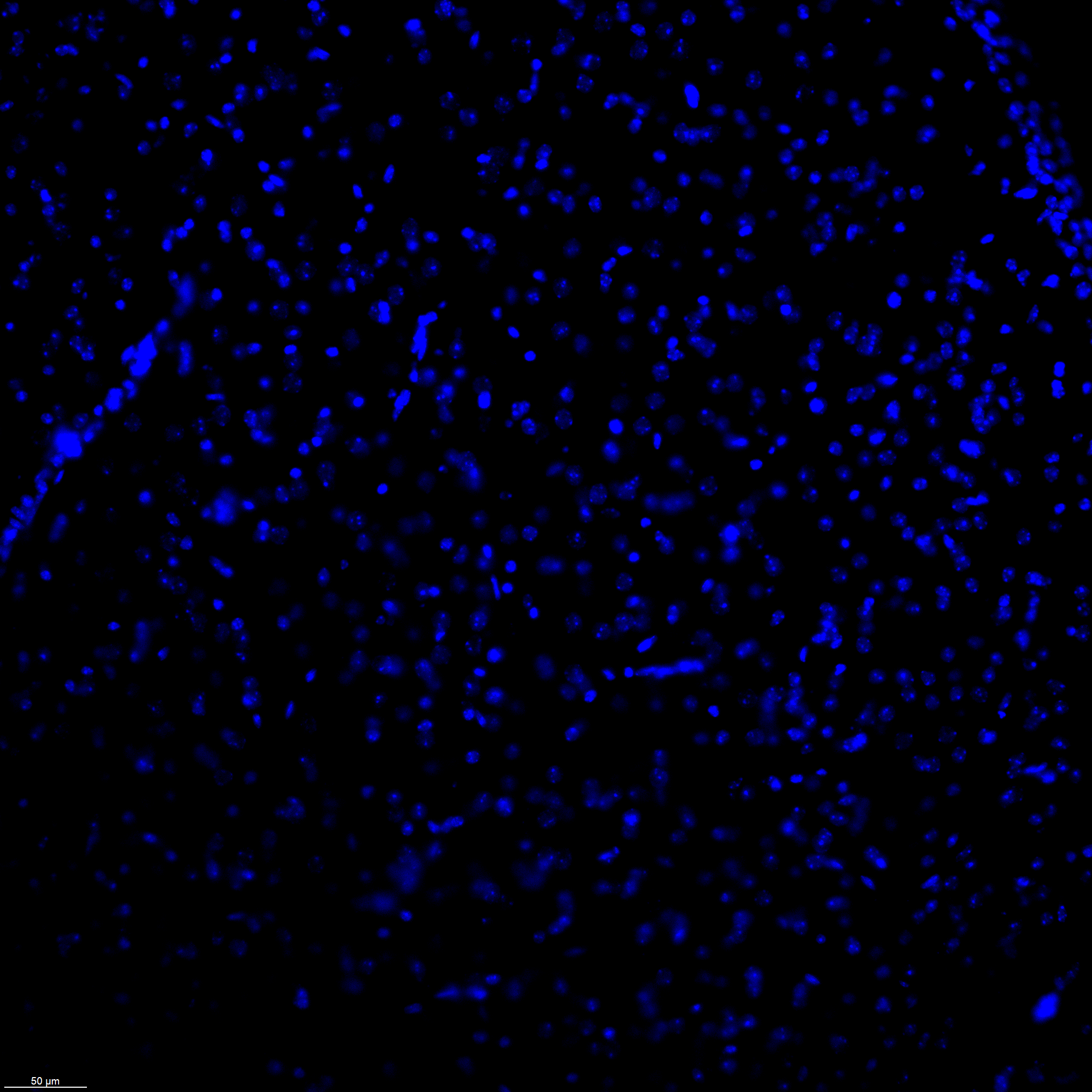

Supplement: Supplementary file 6 — Source data Fig. 4 [file 44319_2025_403_MOESM6_ESM.zip › Figure 4/4E/TH-C21/Hoechst.tif]

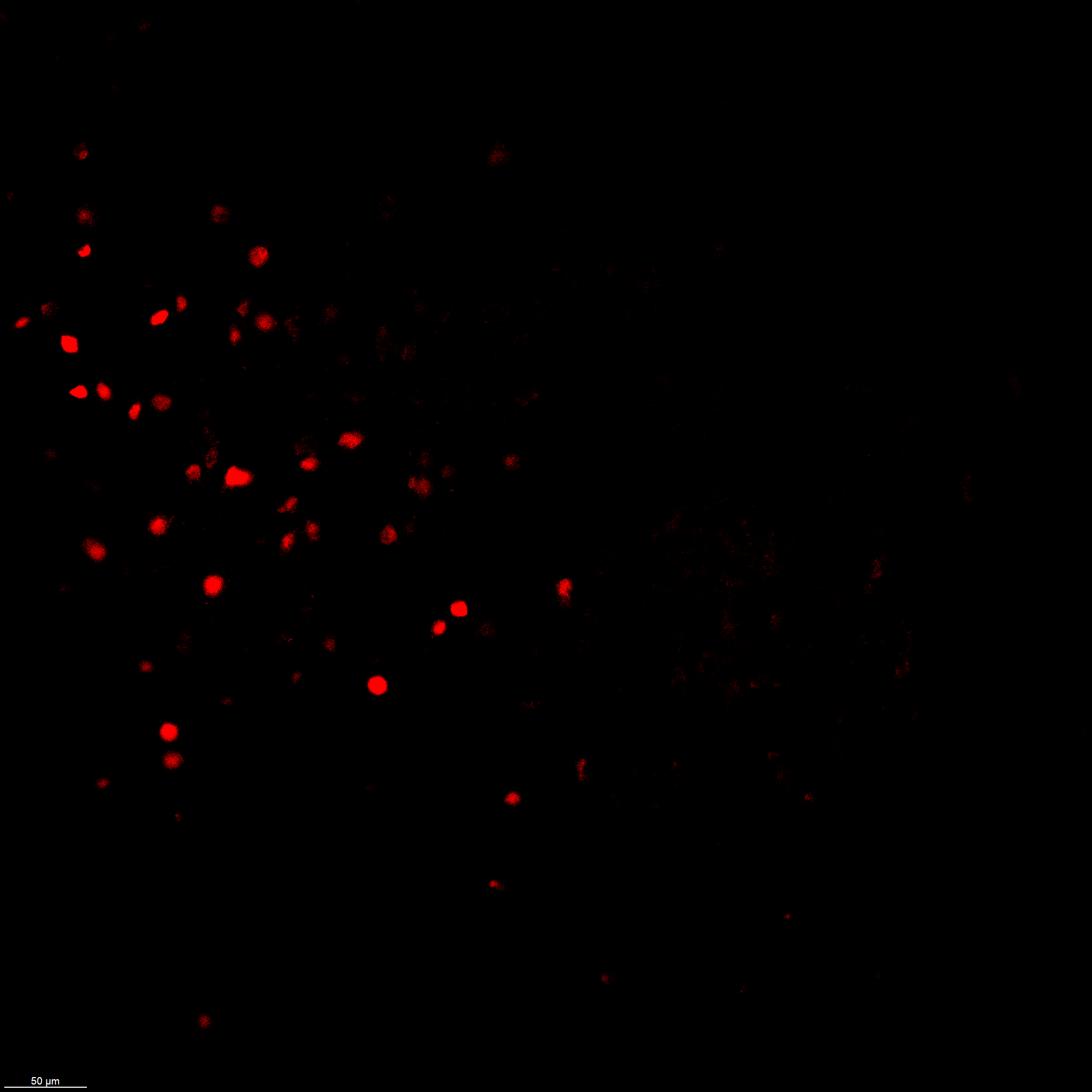

Supplement: Supplementary file 6 — Source data Fig. 4 [file 44319_2025_403_MOESM6_ESM.zip › Figure 4/4E/GAD2-C21/c-fos.tif]

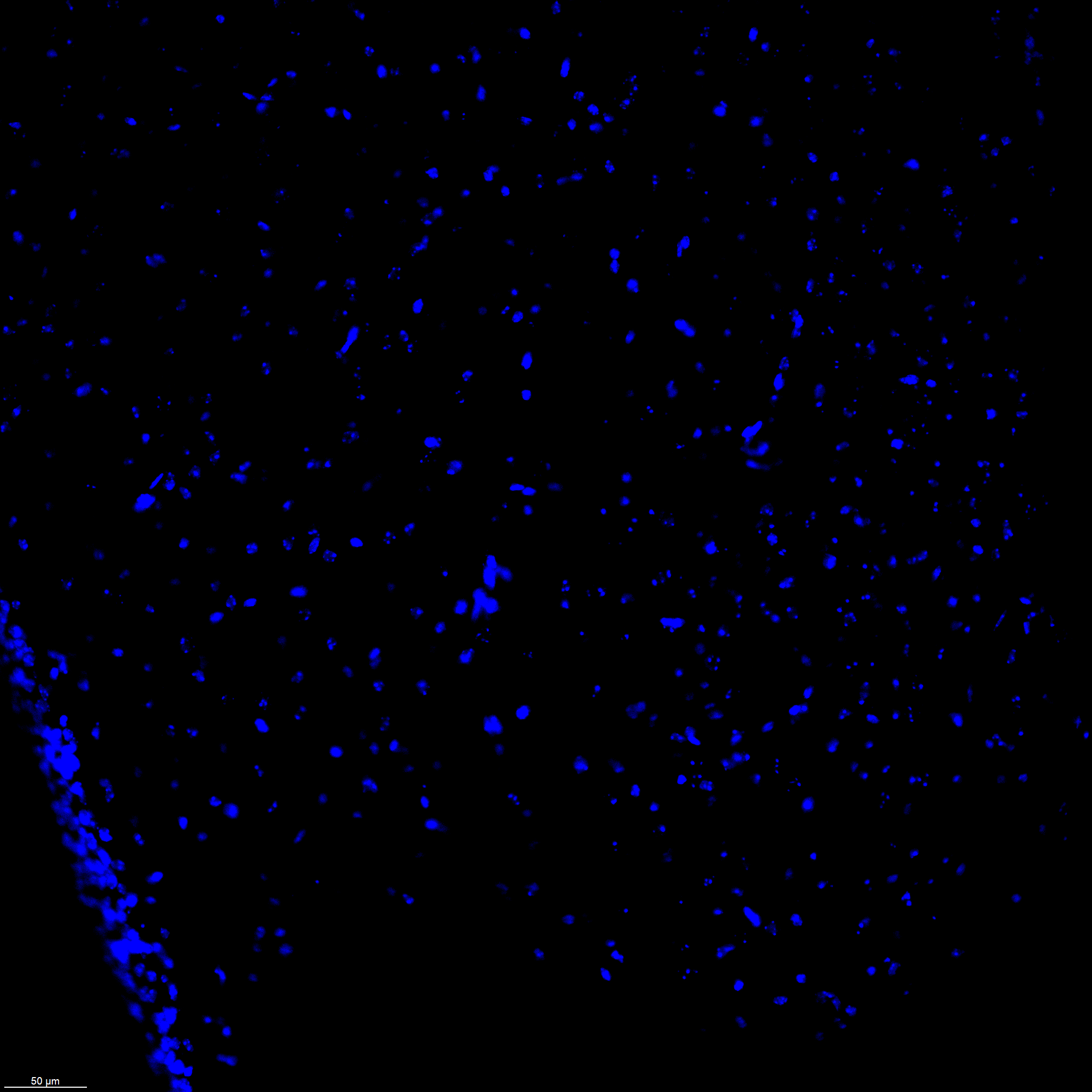

Supplement: Supplementary file 6 — Source data Fig. 4 [file 44319_2025_403_MOESM6_ESM.zip › Figure 4/4E/GAD2-C21/Hoechst1.tif]

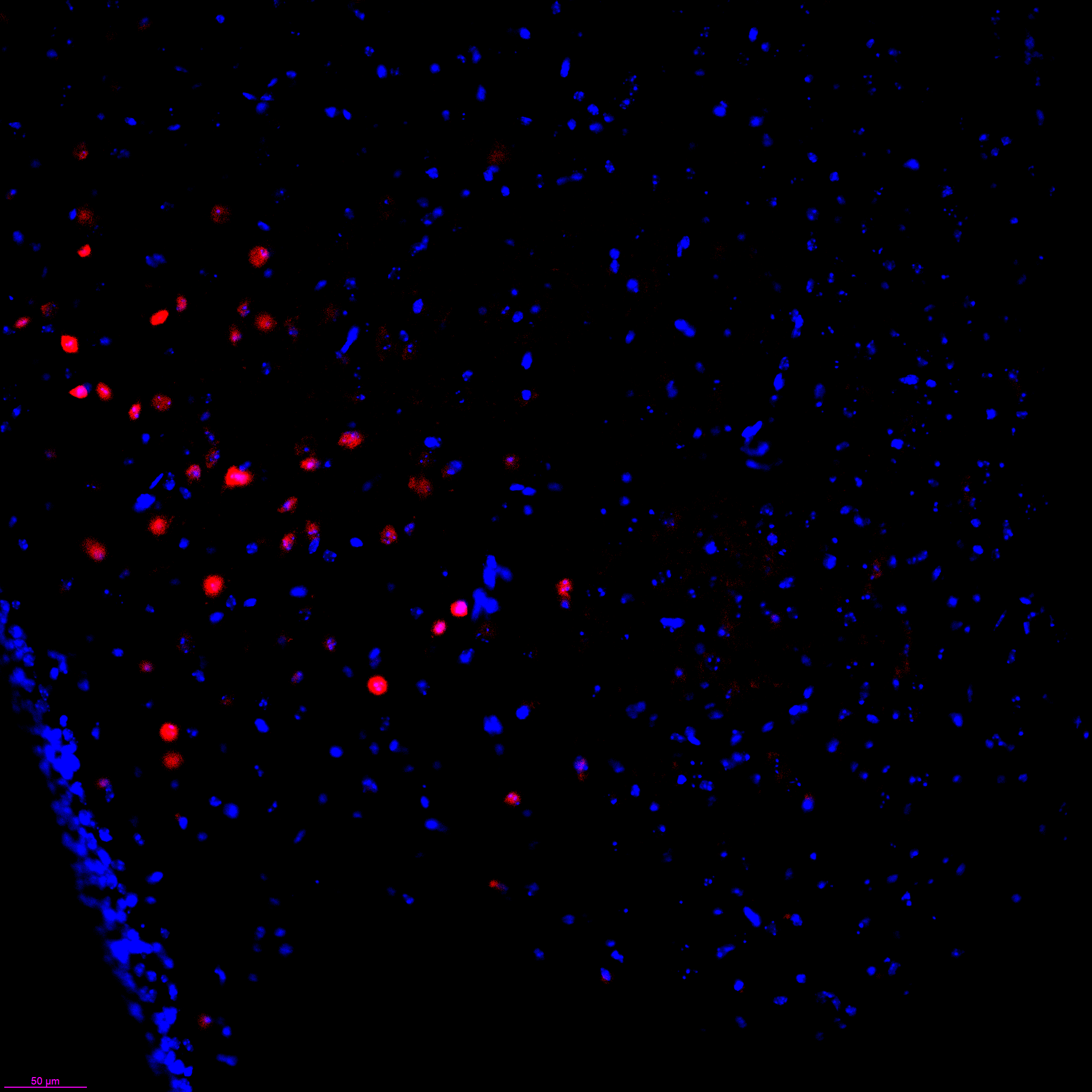

Supplement: Supplementary file 6 — Source data Fig. 4 [file 44319_2025_403_MOESM6_ESM.zip › Figure 4/4E/GAD2-C21/overlay.tif]

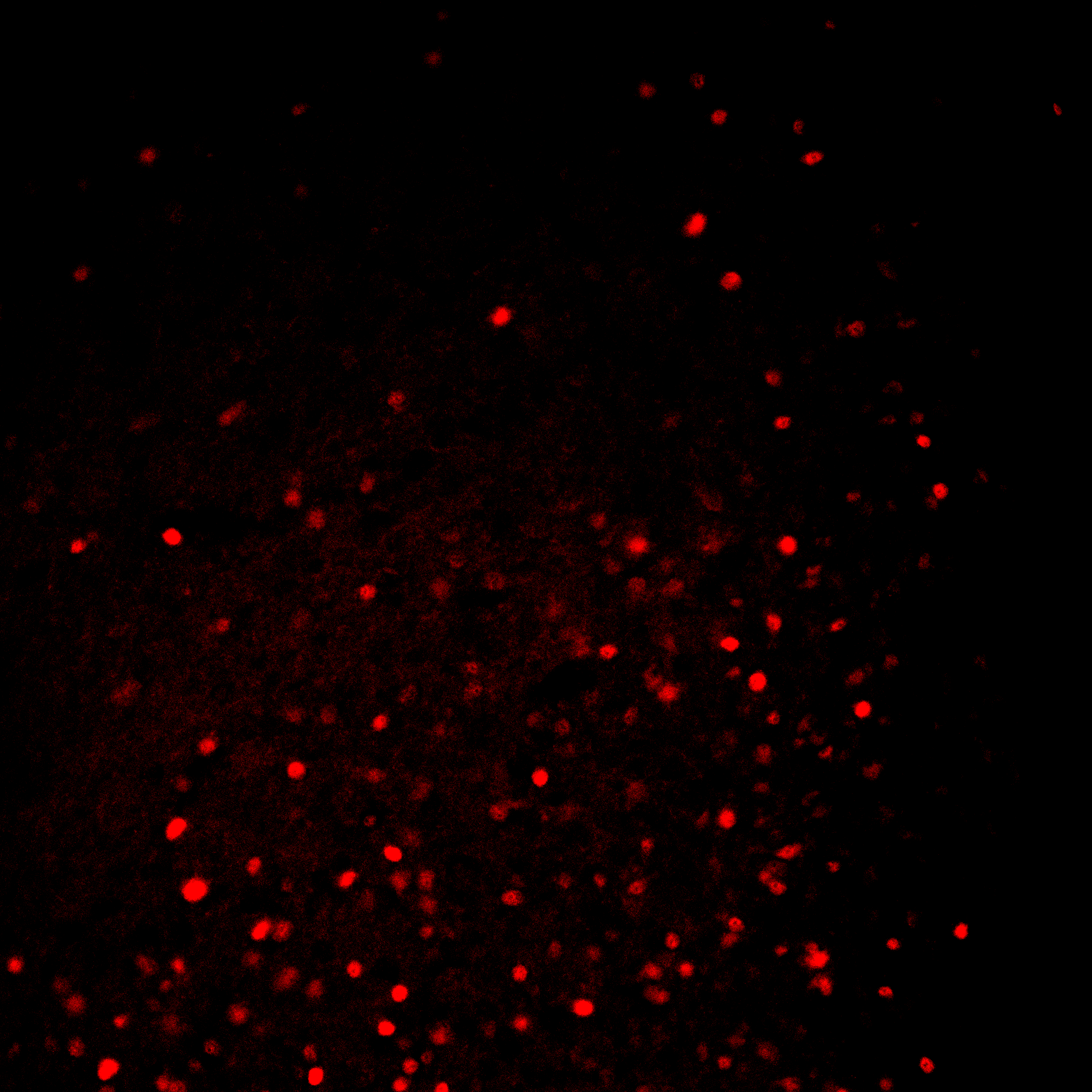

Supplement: Supplementary file 6 — Source data Fig. 4 [file 44319_2025_403_MOESM6_ESM.zip › Figure 4/4E/GAD2-V/c-fos.tif]

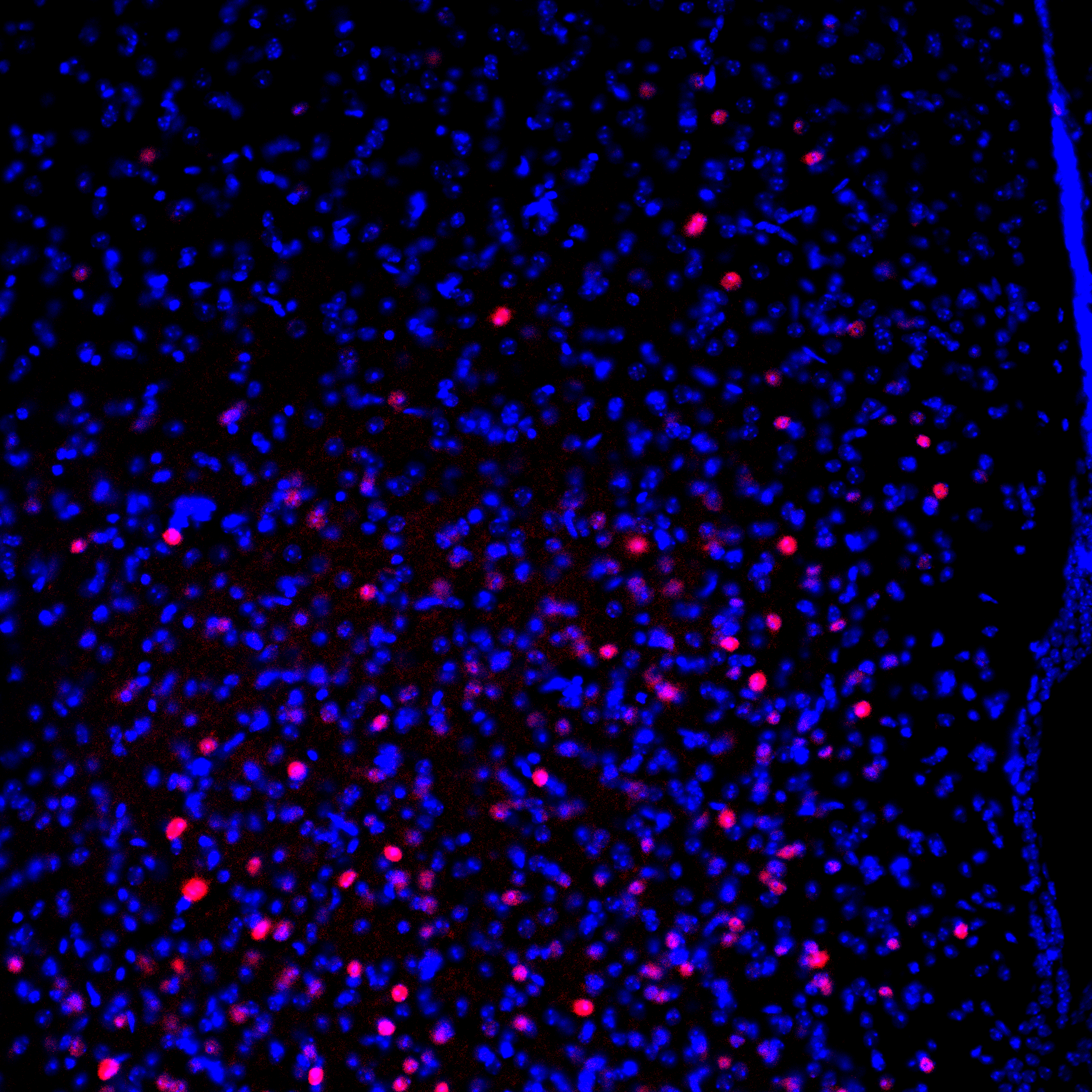

Supplement: Supplementary file 6 — Source data Fig. 4 [file 44319_2025_403_MOESM6_ESM.zip › Figure 4/4E/GAD2-V/overlay.tif]

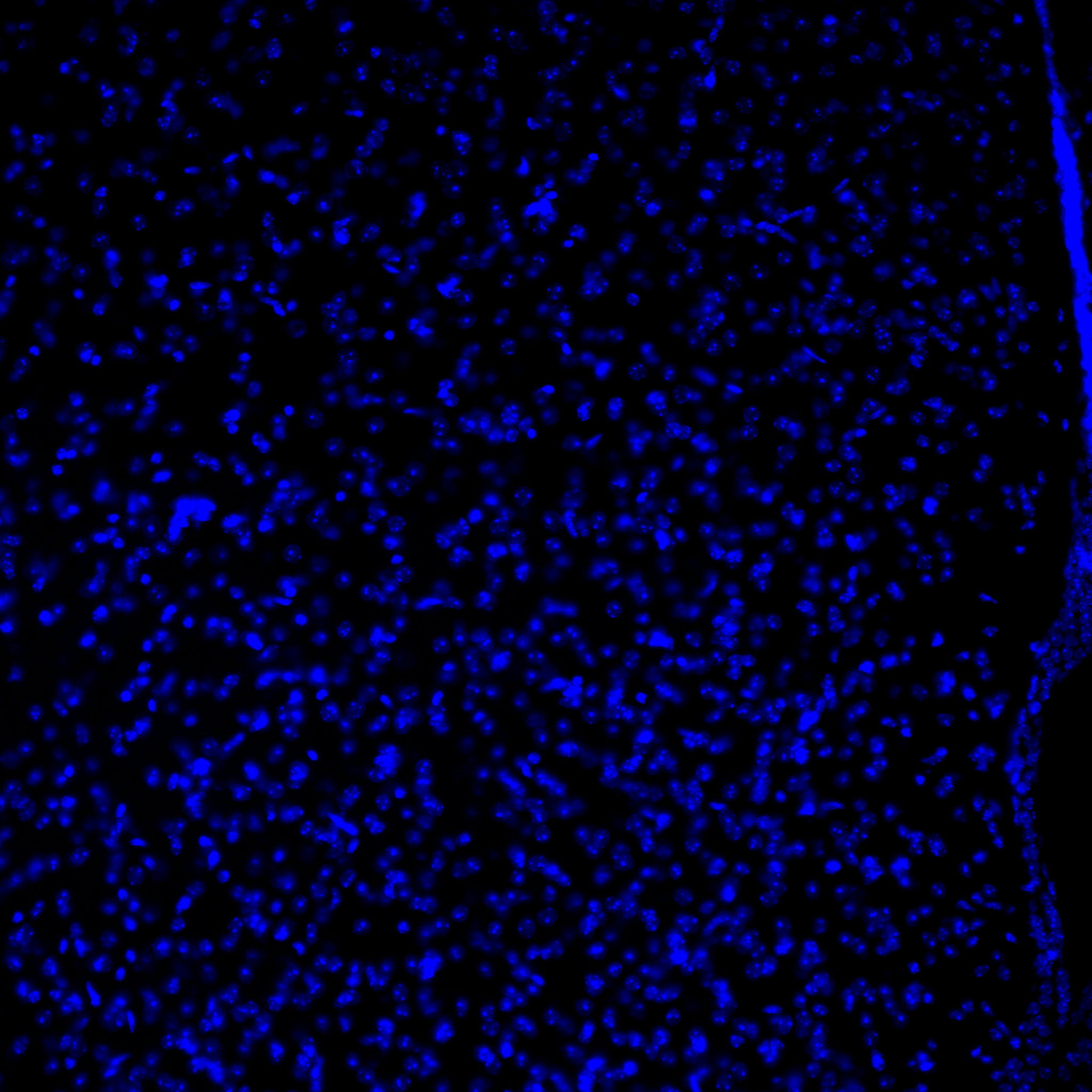

Supplement: Supplementary file 6 — Source data Fig. 4 [file 44319_2025_403_MOESM6_ESM.zip › Figure 4/4E/GAD2-V/Hoechst.tif]

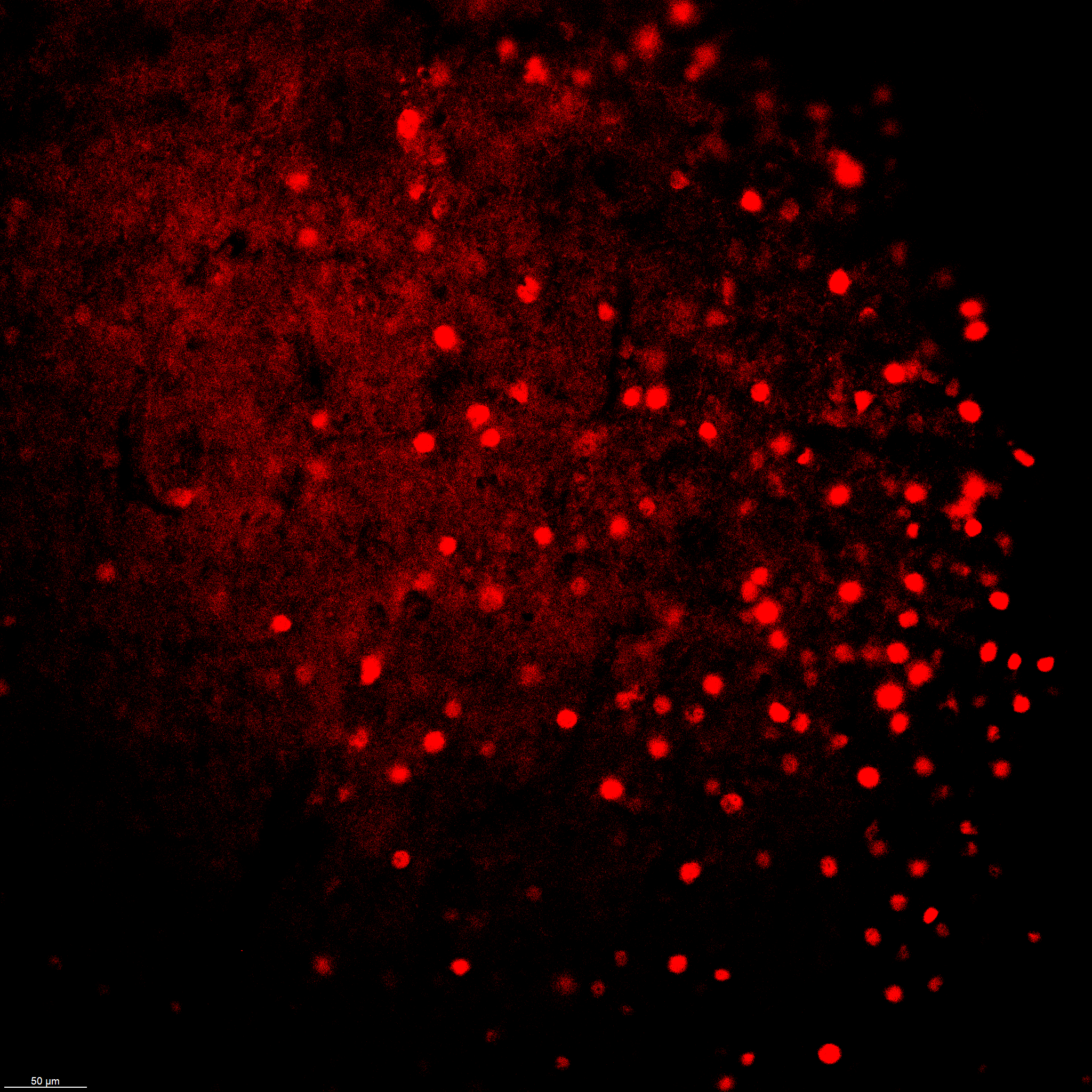

Supplement: Supplementary file 6 — Source data Fig. 4 [file 44319_2025_403_MOESM6_ESM.zip › Figure 4/4E/Control/cfos.tif]

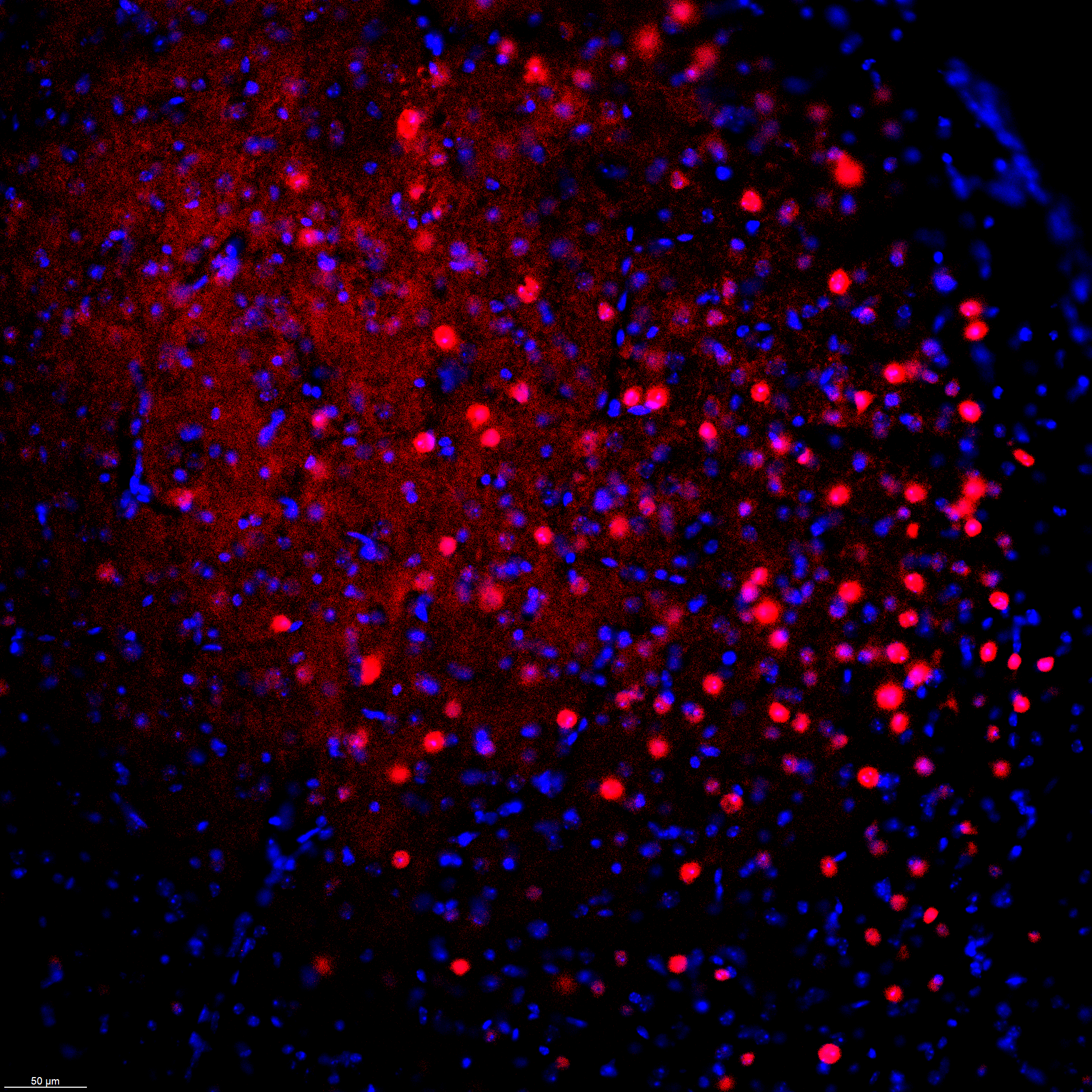

Supplement: Supplementary file 6 — Source data Fig. 4 [file 44319_2025_403_MOESM6_ESM.zip › Figure 4/4E/Control/overlay.tif]

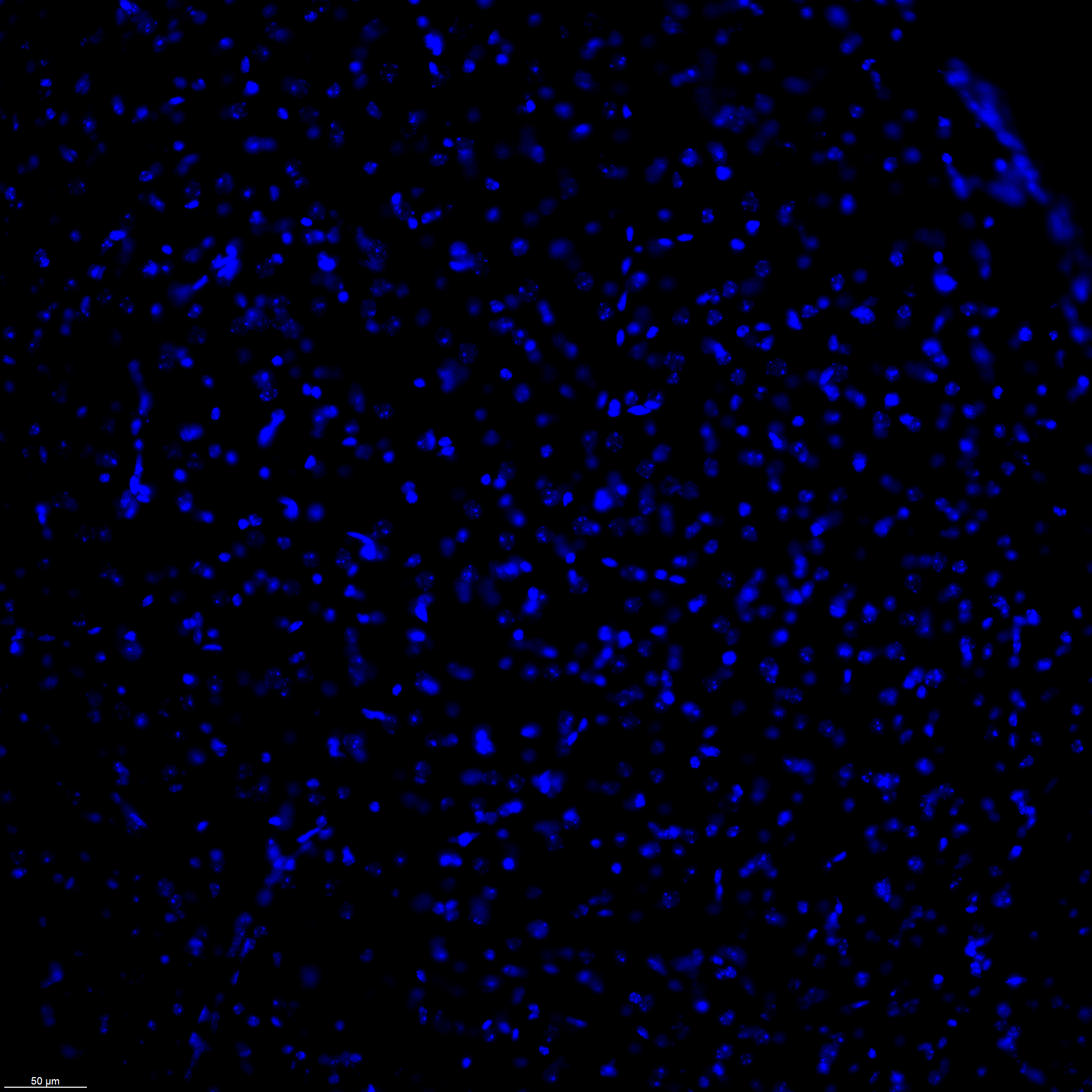

Supplement: Supplementary file 6 — Source data Fig. 4 [file 44319_2025_403_MOESM6_ESM.zip › Figure 4/4E/Control/Hoechst.tif]

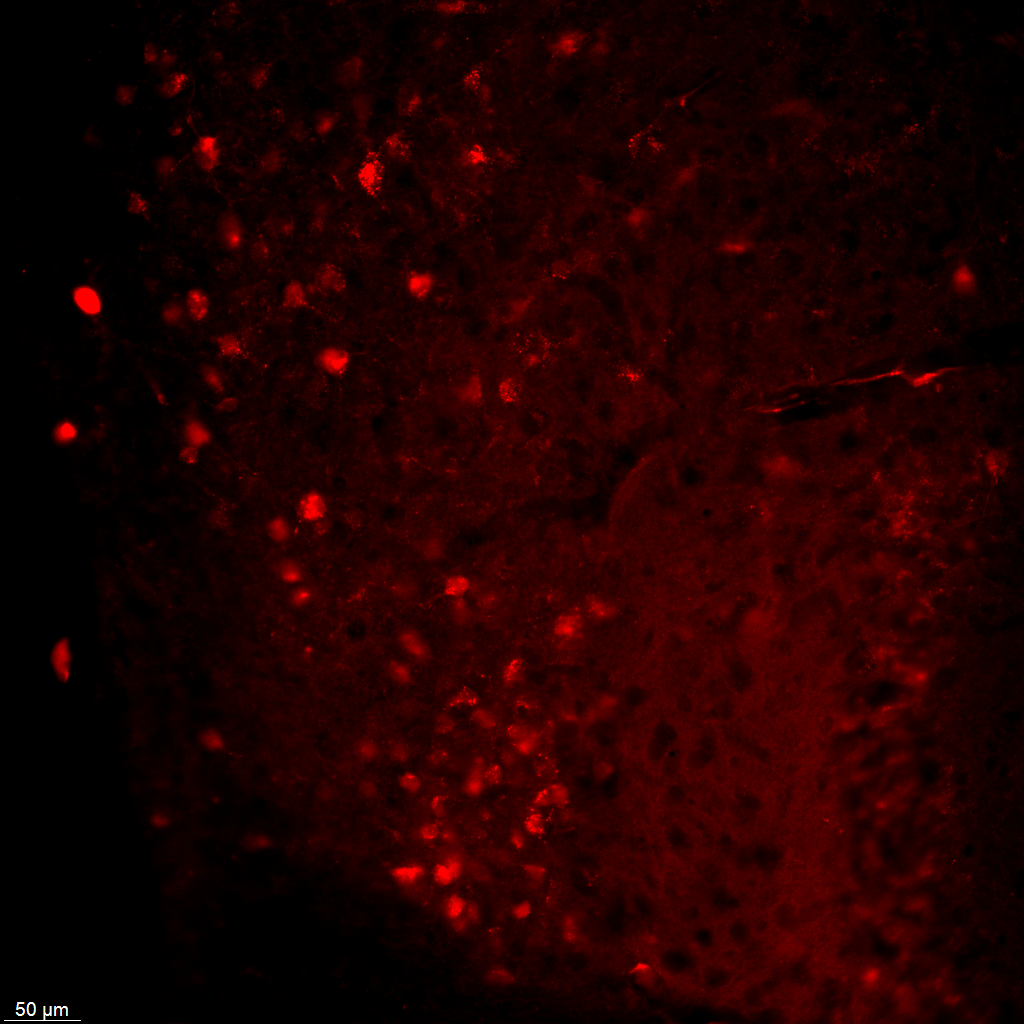

Supplement: Supplementary file 6 — Source data Fig. 4 [file 44319_2025_403_MOESM6_ESM.zip › Figure 4/4B/TH-Cre/HM4Di-mCherry.tif]

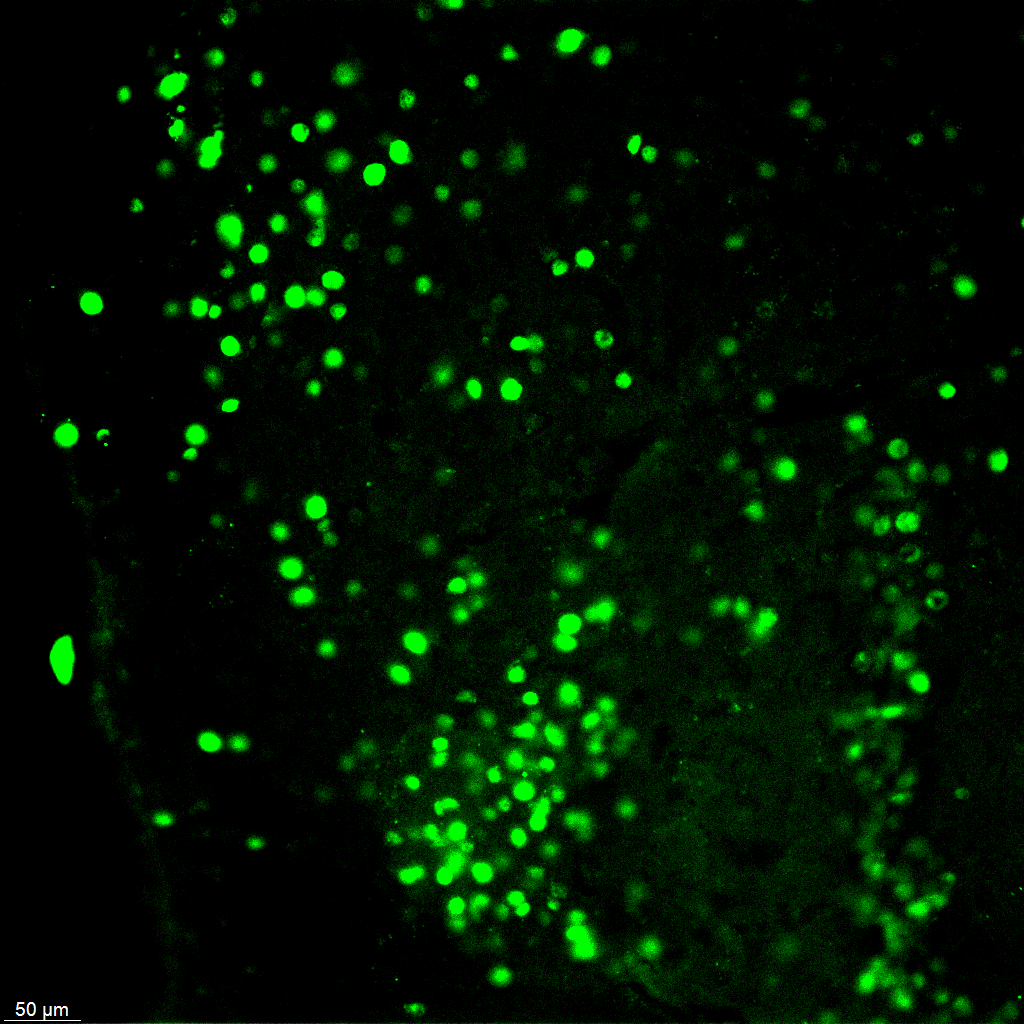

Supplement: Supplementary file 6 — Source data Fig. 4 [file 44319_2025_403_MOESM6_ESM.zip › Figure 4/4B/TH-Cre/TH-Cre.tif]

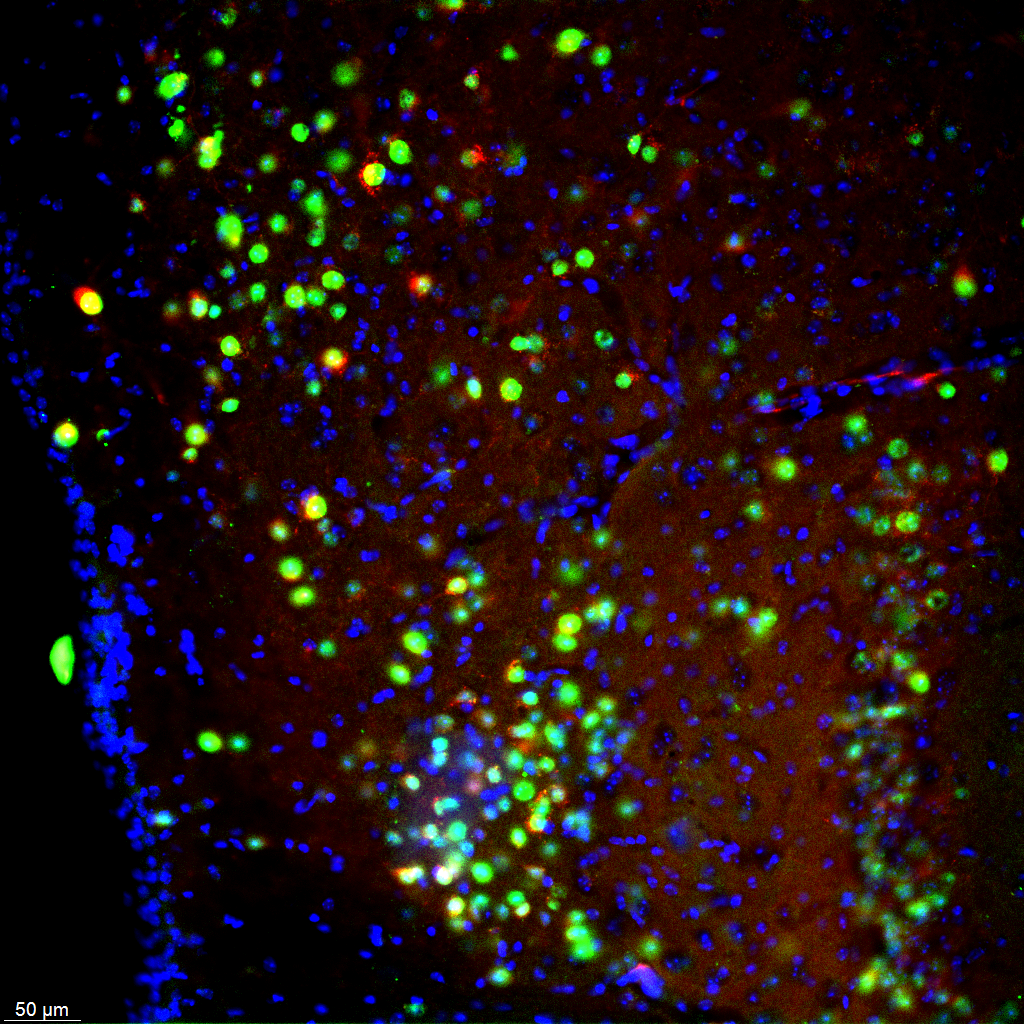

Supplement: Supplementary file 6 — Source data Fig. 4 [file 44319_2025_403_MOESM6_ESM.zip › Figure 4/4B/TH-Cre/overlay.tif]

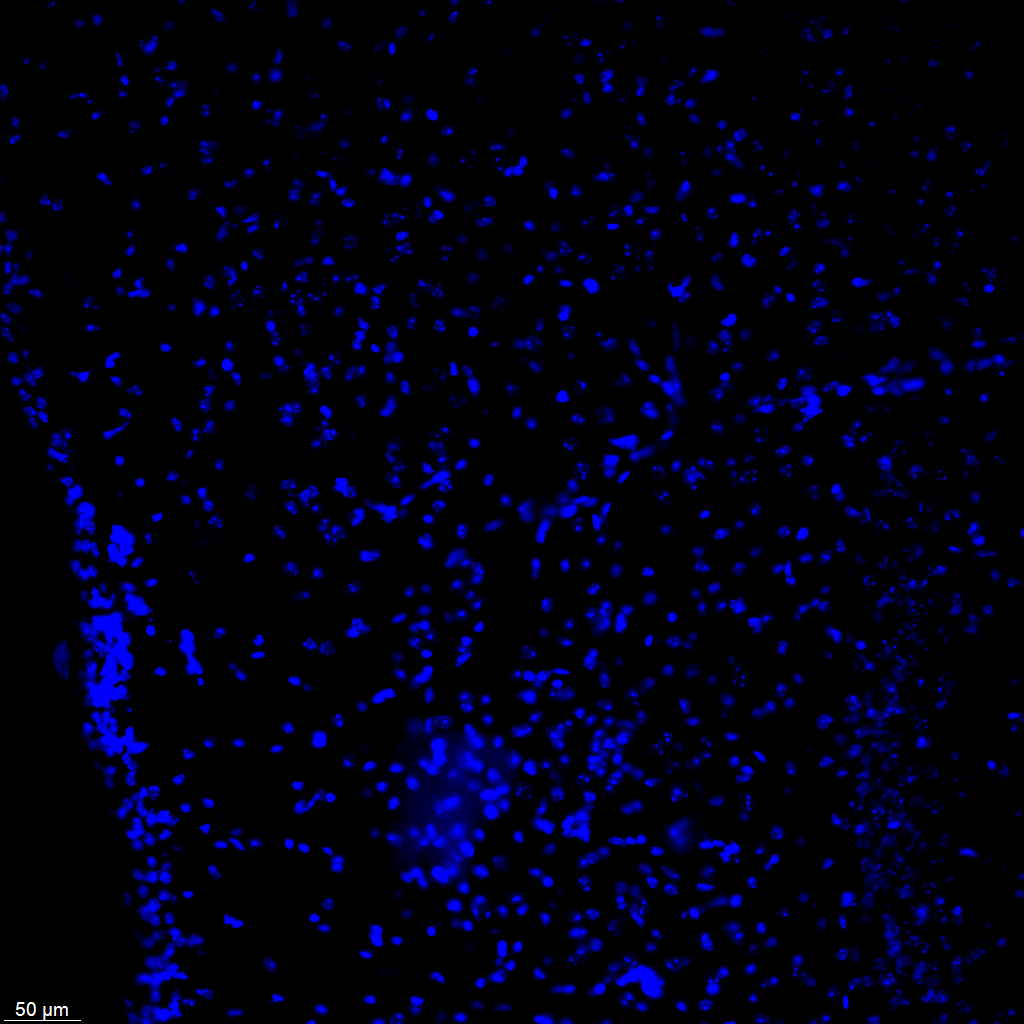

Supplement: Supplementary file 6 — Source data Fig. 4 [file 44319_2025_403_MOESM6_ESM.zip › Figure 4/4B/TH-Cre/Hoechst.tif]

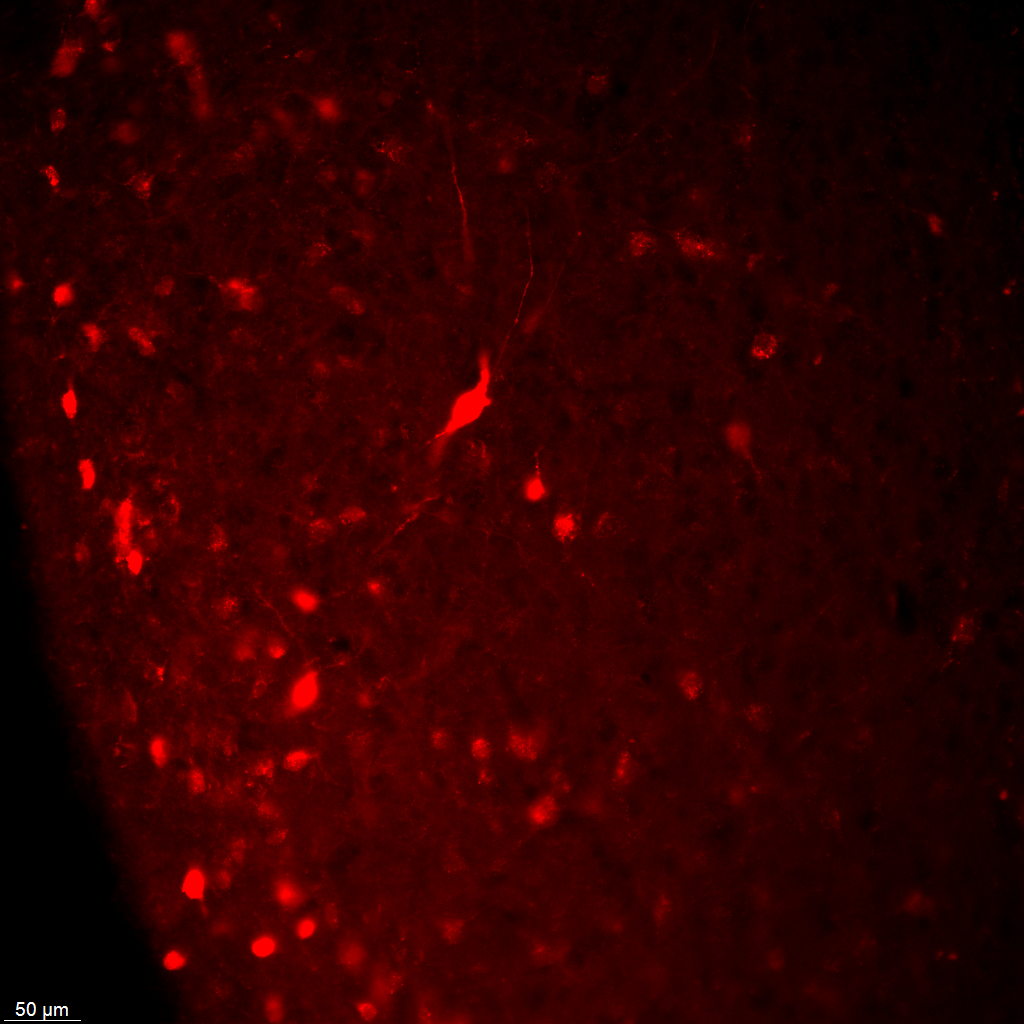

Supplement: Supplementary file 6 — Source data Fig. 4 [file 44319_2025_403_MOESM6_ESM.zip › Figure 4/4B/GAD2-Cre/HM4Di-mCherry.tif]

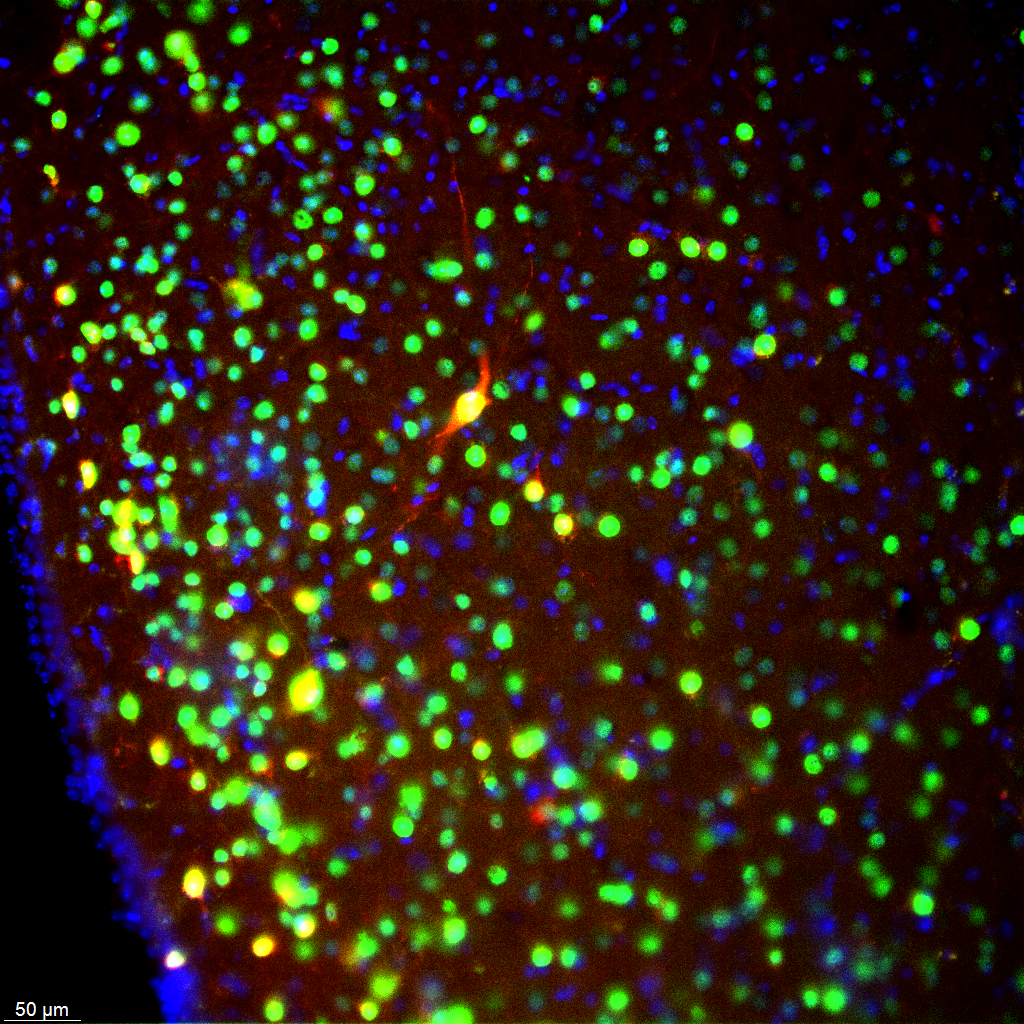

Supplement: Supplementary file 6 — Source data Fig. 4 [file 44319_2025_403_MOESM6_ESM.zip › Figure 4/4B/GAD2-Cre/overlay.tif]

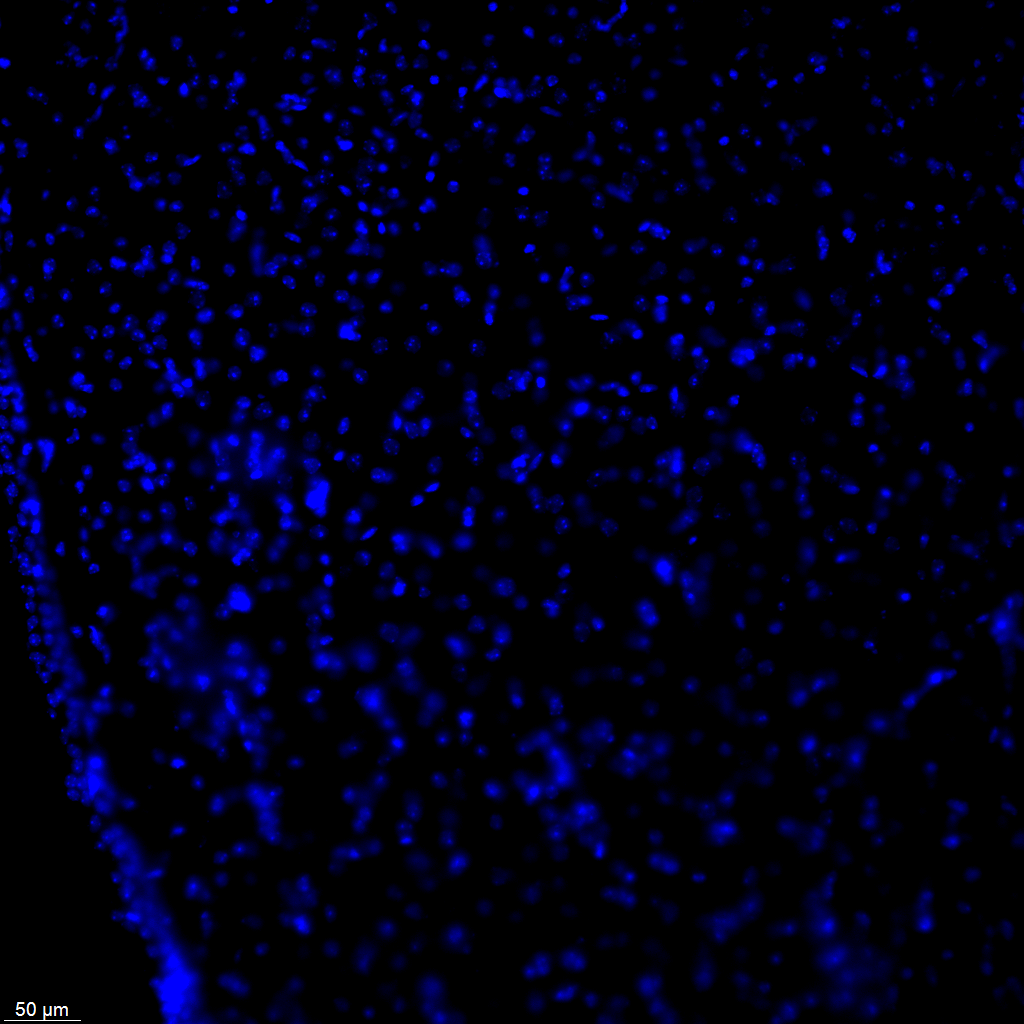

Supplement: Supplementary file 6 — Source data Fig. 4 [file 44319_2025_403_MOESM6_ESM.zip › Figure 4/4B/GAD2-Cre/Hoechst.tif]

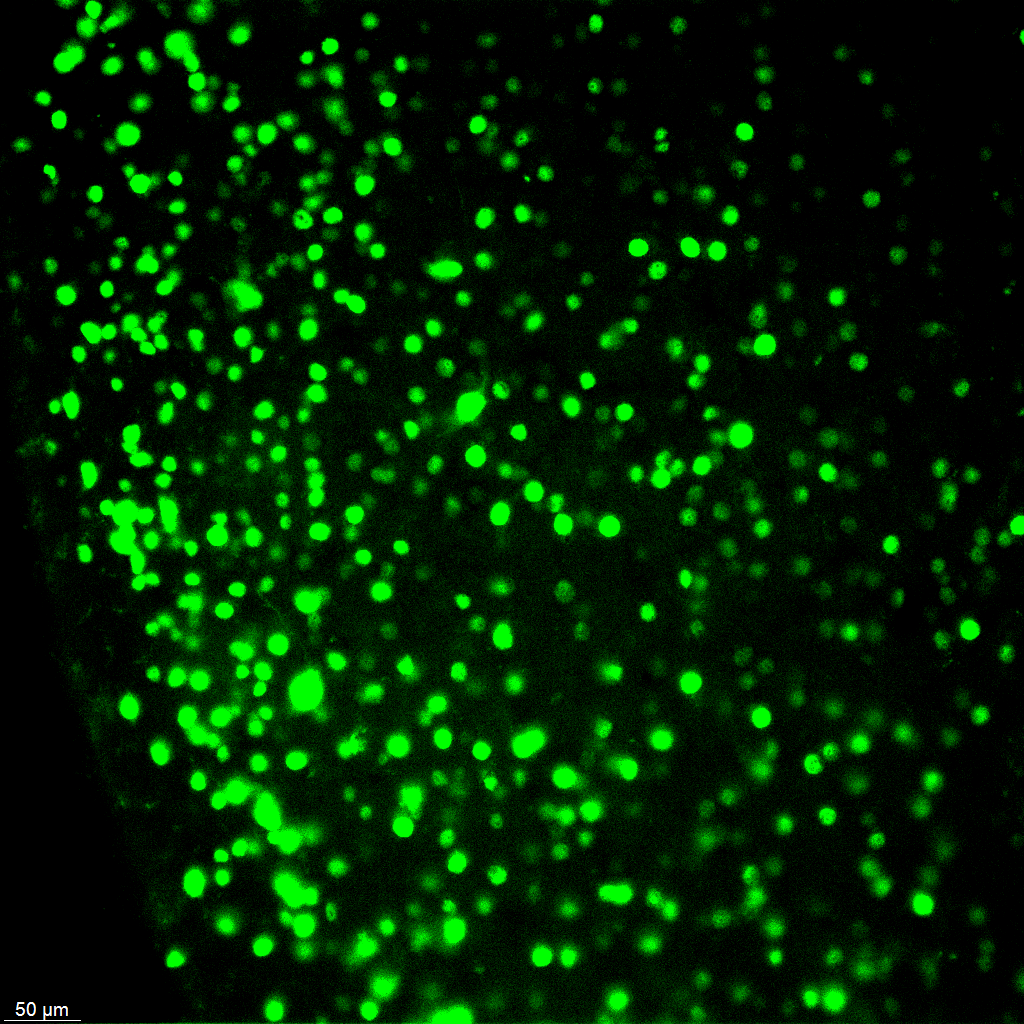

Supplement: Supplementary file 6 — Source data Fig. 4 [file 44319_2025_403_MOESM6_ESM.zip › Figure 4/4B/GAD2-Cre/GAD1-cre.tif]

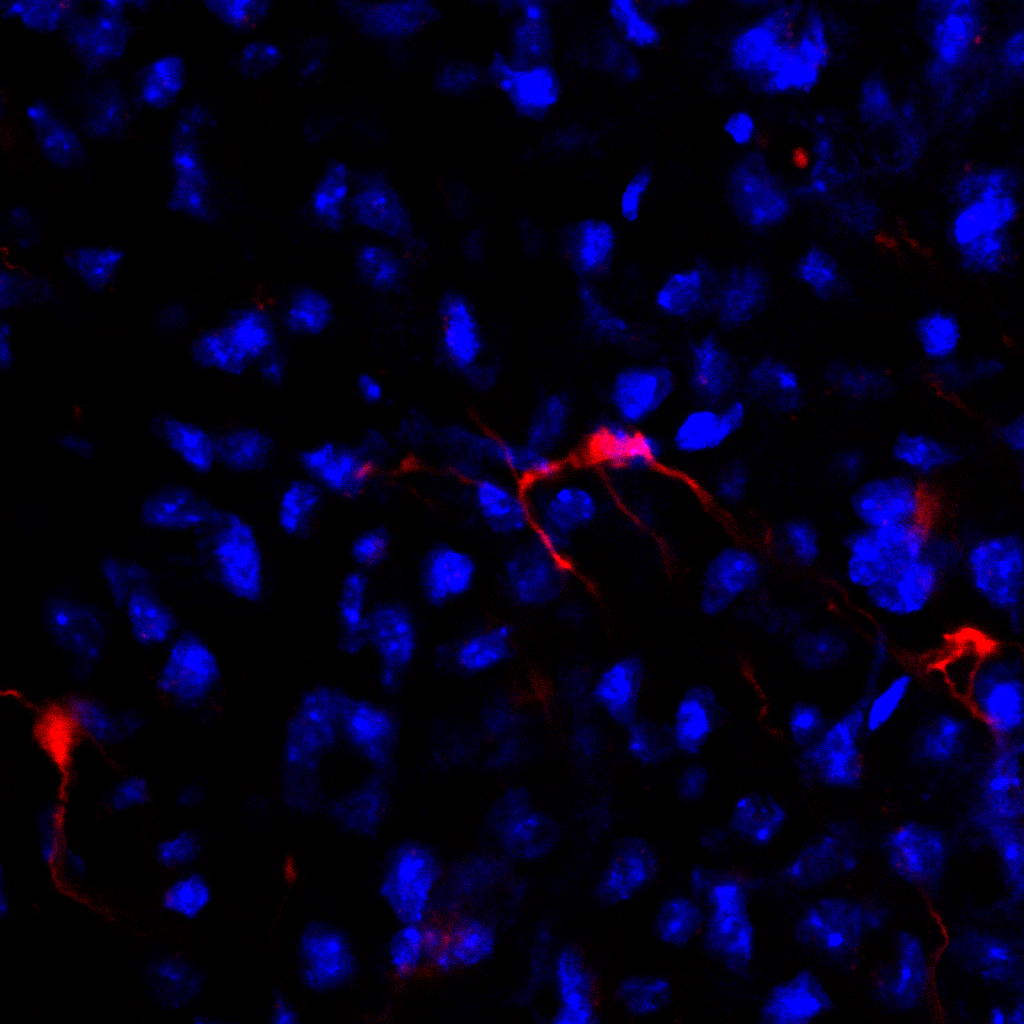

Supplement: Supplementary file 7 — Source data Fig. 5 [file 44319_2025_403_MOESM7_ESM.zip › Figure 5/5C/CAG-tdTomato/overlay 2.tif]

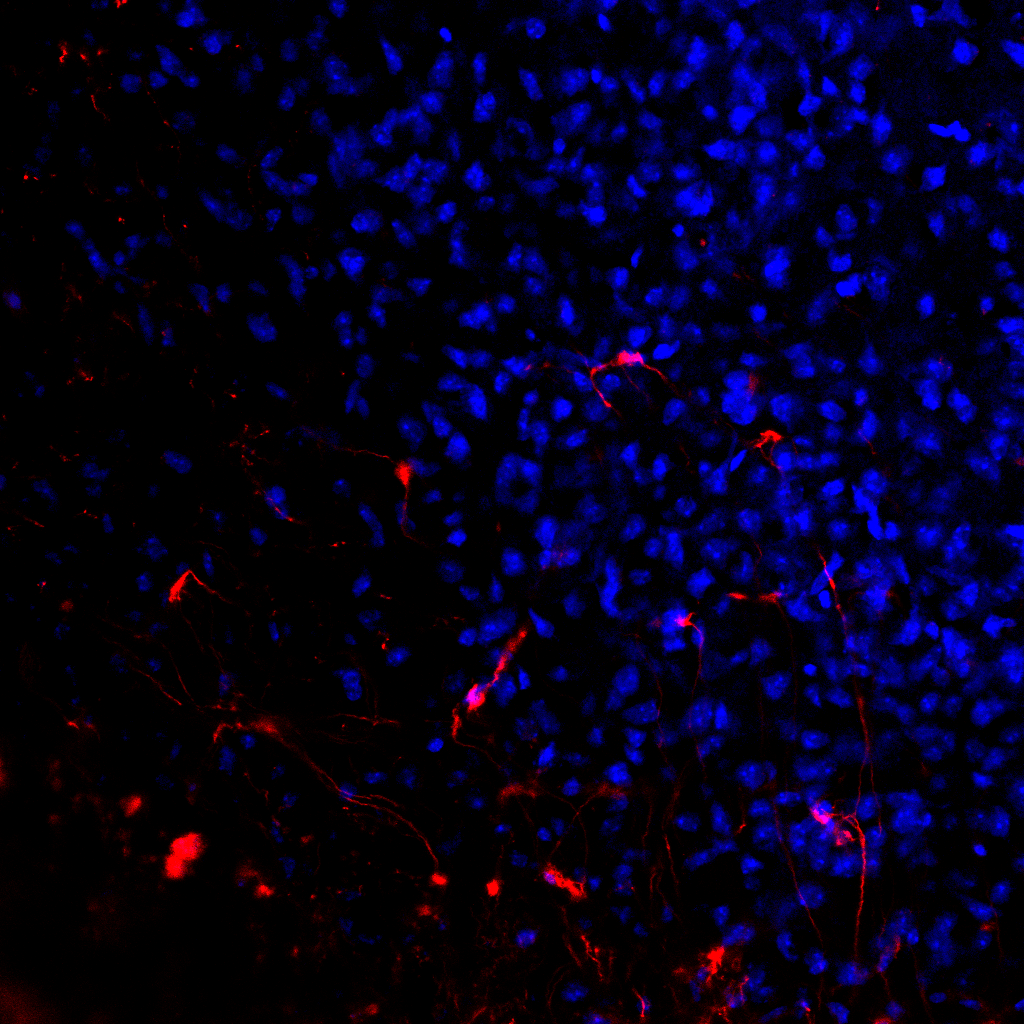

Supplement: Supplementary file 7 — Source data Fig. 5 [file 44319_2025_403_MOESM7_ESM.zip › Figure 5/5C/CAG-tdTomato/overlay 1.tif]

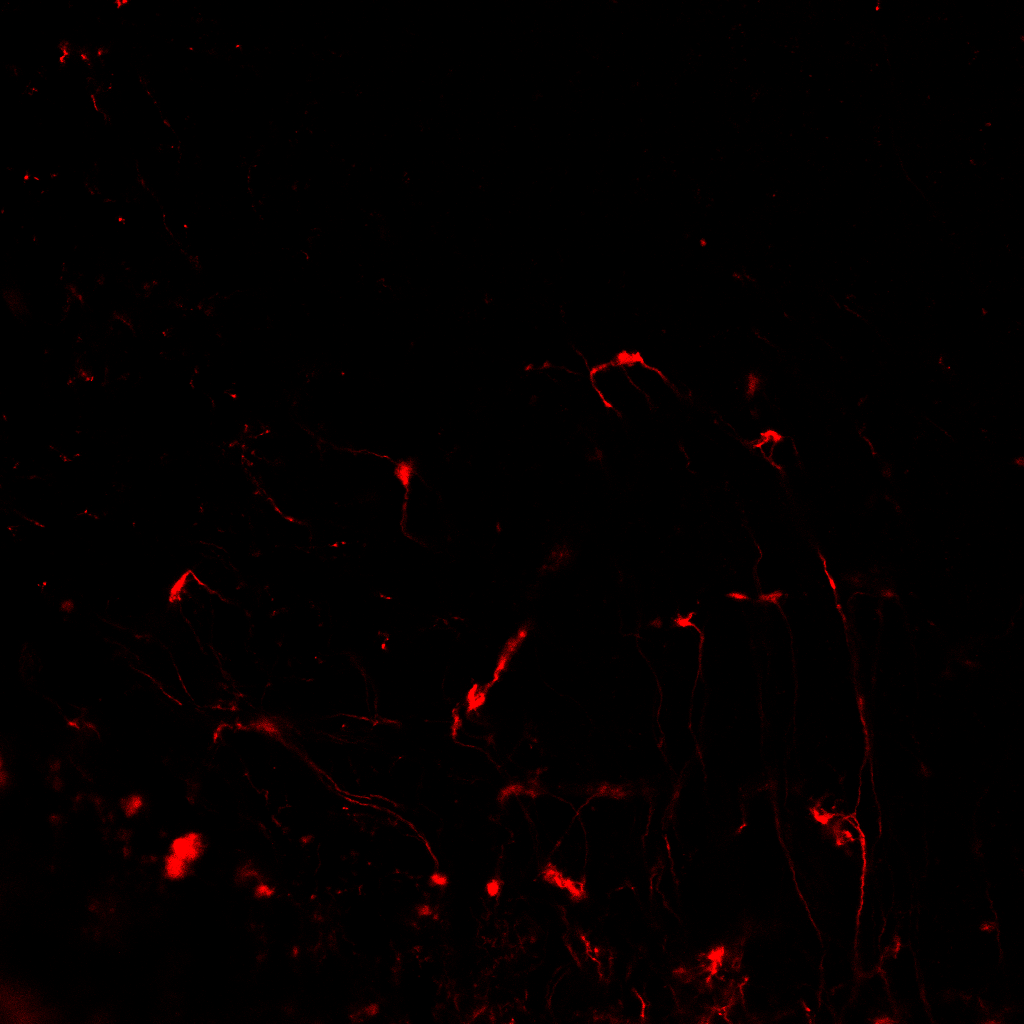

Supplement: Supplementary file 7 — Source data Fig. 5 [file 44319_2025_403_MOESM7_ESM.zip › Figure 5/5C/CAG-tdTomato/CAG-tdTomato.tif]

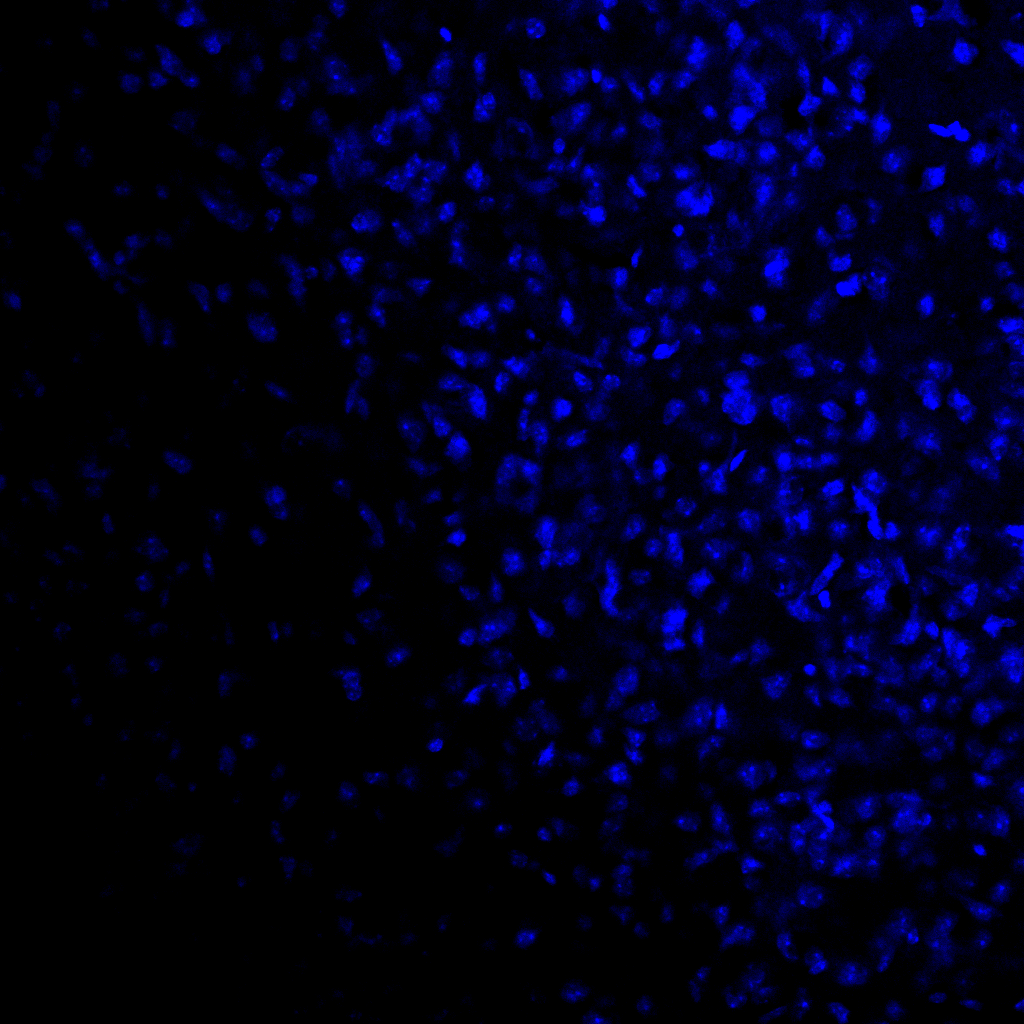

Supplement: Supplementary file 7 — Source data Fig. 5 [file 44319_2025_403_MOESM7_ESM.zip › Figure 5/5C/CAG-tdTomato/Hoechst.tif]

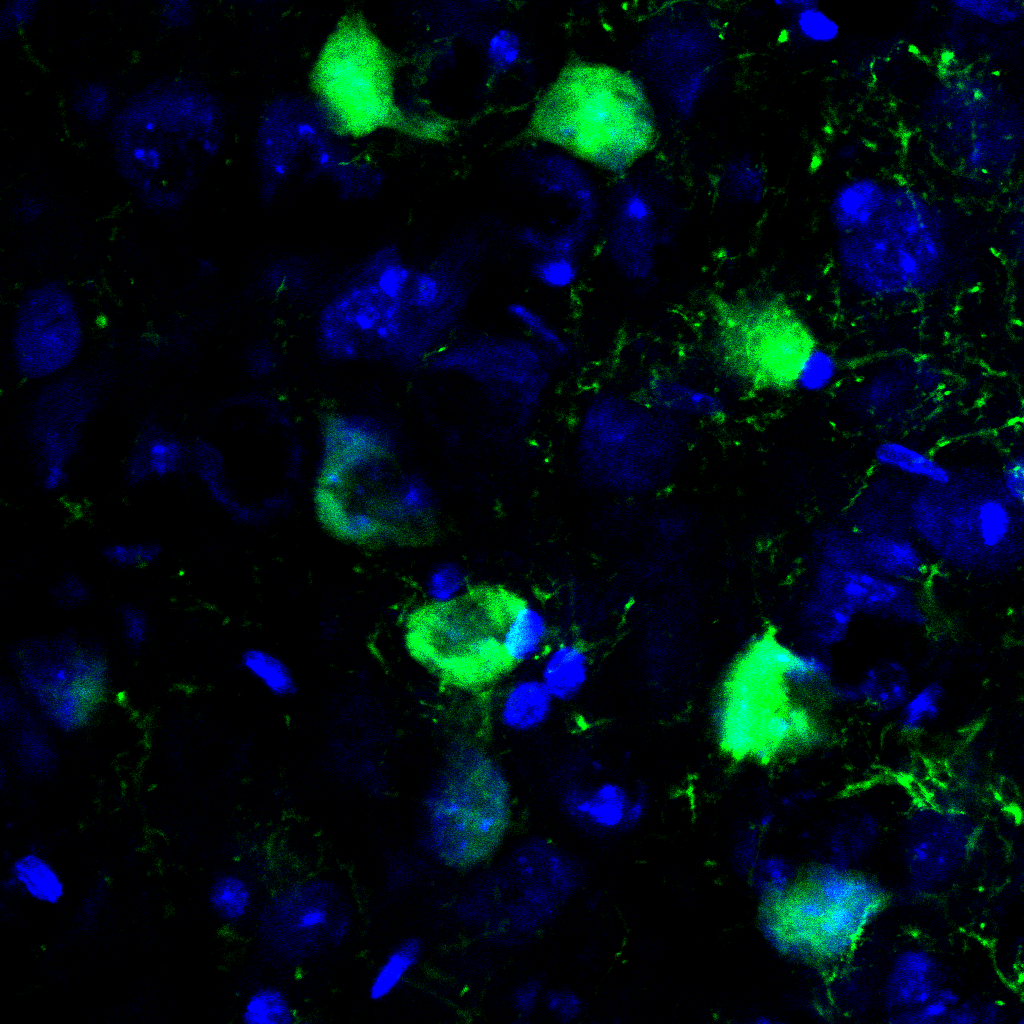

Supplement: Supplementary file 7 — Source data Fig. 5 [file 44319_2025_403_MOESM7_ESM.zip › Figure 5/5C/CAG-GFP/overlay 2.tif]

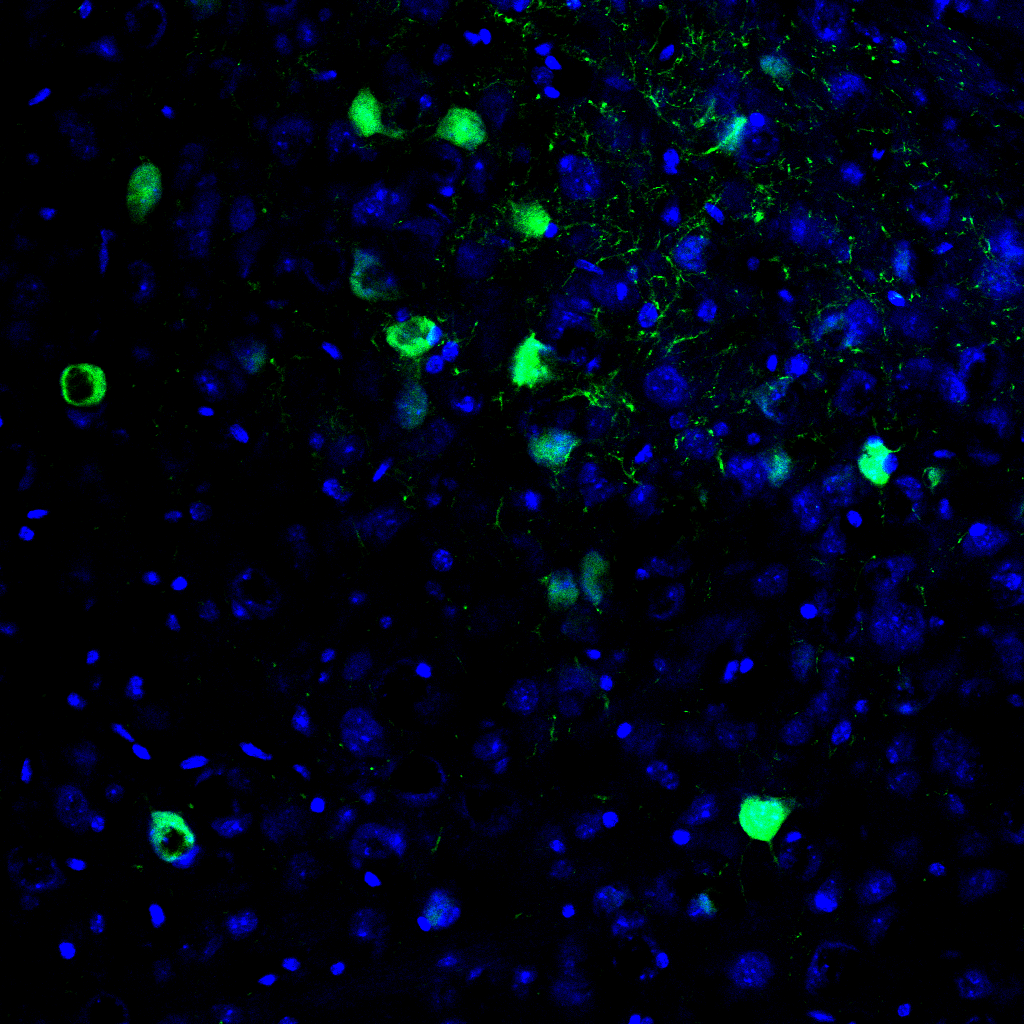

Supplement: Supplementary file 7 — Source data Fig. 5 [file 44319_2025_403_MOESM7_ESM.zip › Figure 5/5C/CAG-GFP/overlay 1.tif]

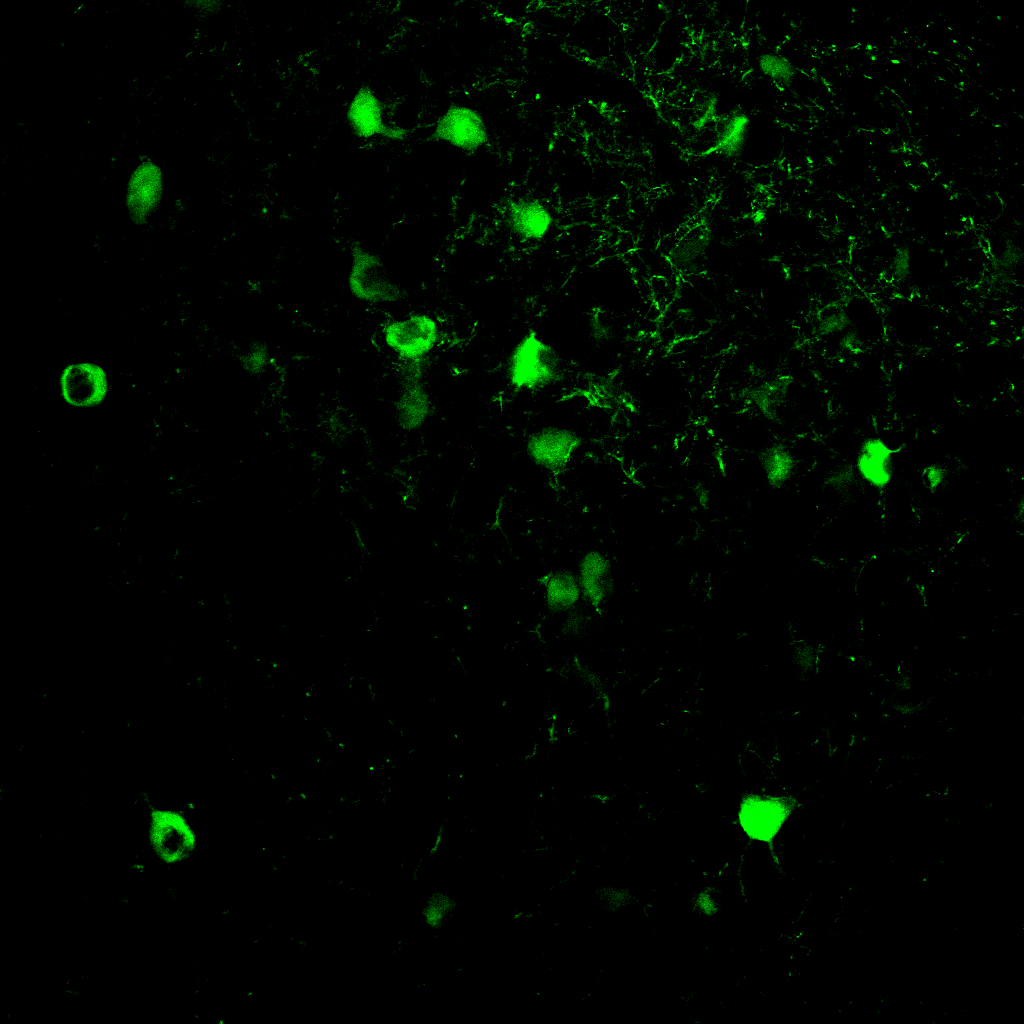

Supplement: Supplementary file 7 — Source data Fig. 5 [file 44319_2025_403_MOESM7_ESM.zip › Figure 5/5C/CAG-GFP/CAG-GFP.tif]

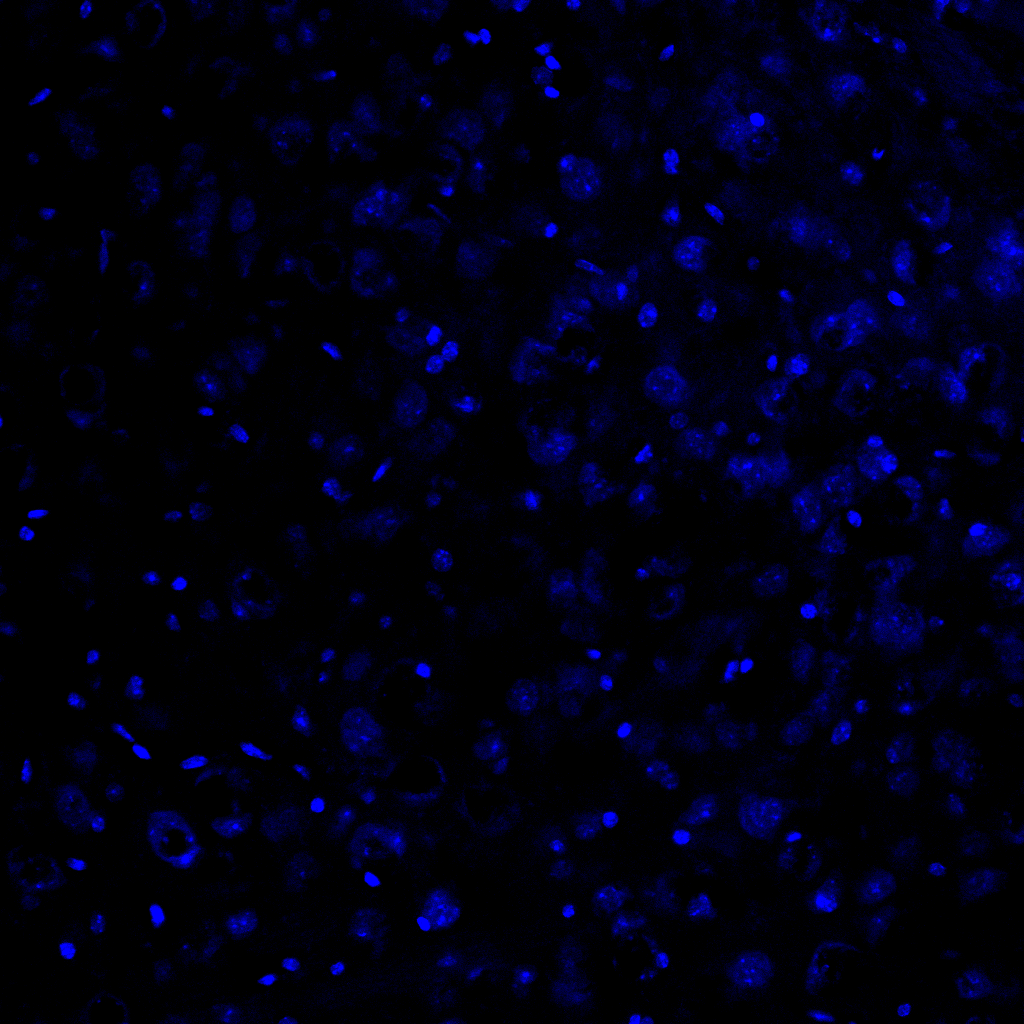

Supplement: Supplementary file 7 — Source data Fig. 5 [file 44319_2025_403_MOESM7_ESM.zip › Figure 5/5C/CAG-GFP/Hoechst.tif]

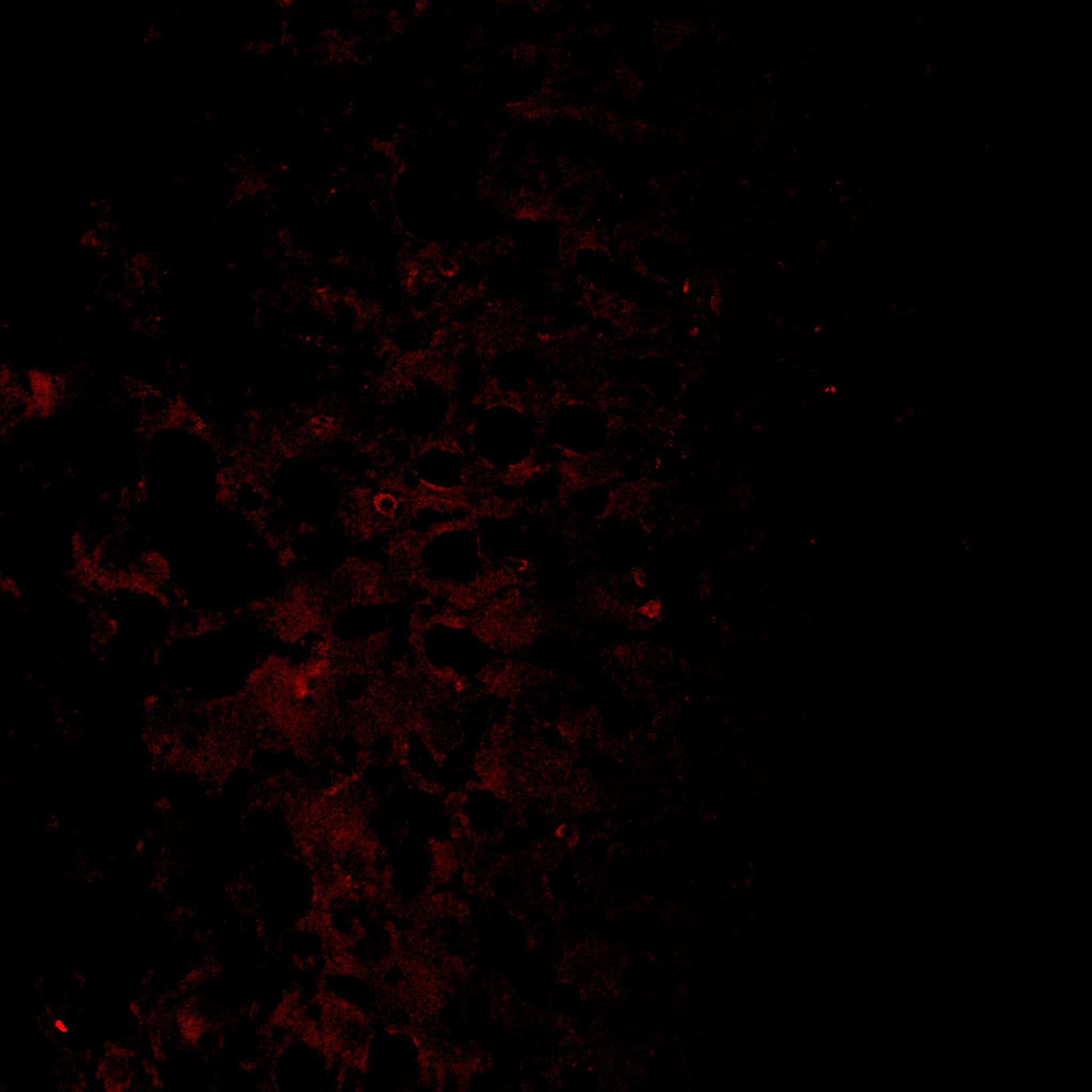

Supplement: Supplementary file 8 — Source data Fig. 6 [file 44319_2025_403_MOESM8_ESM.zip › Figure 6/6B/BLA/mCherry.tif]

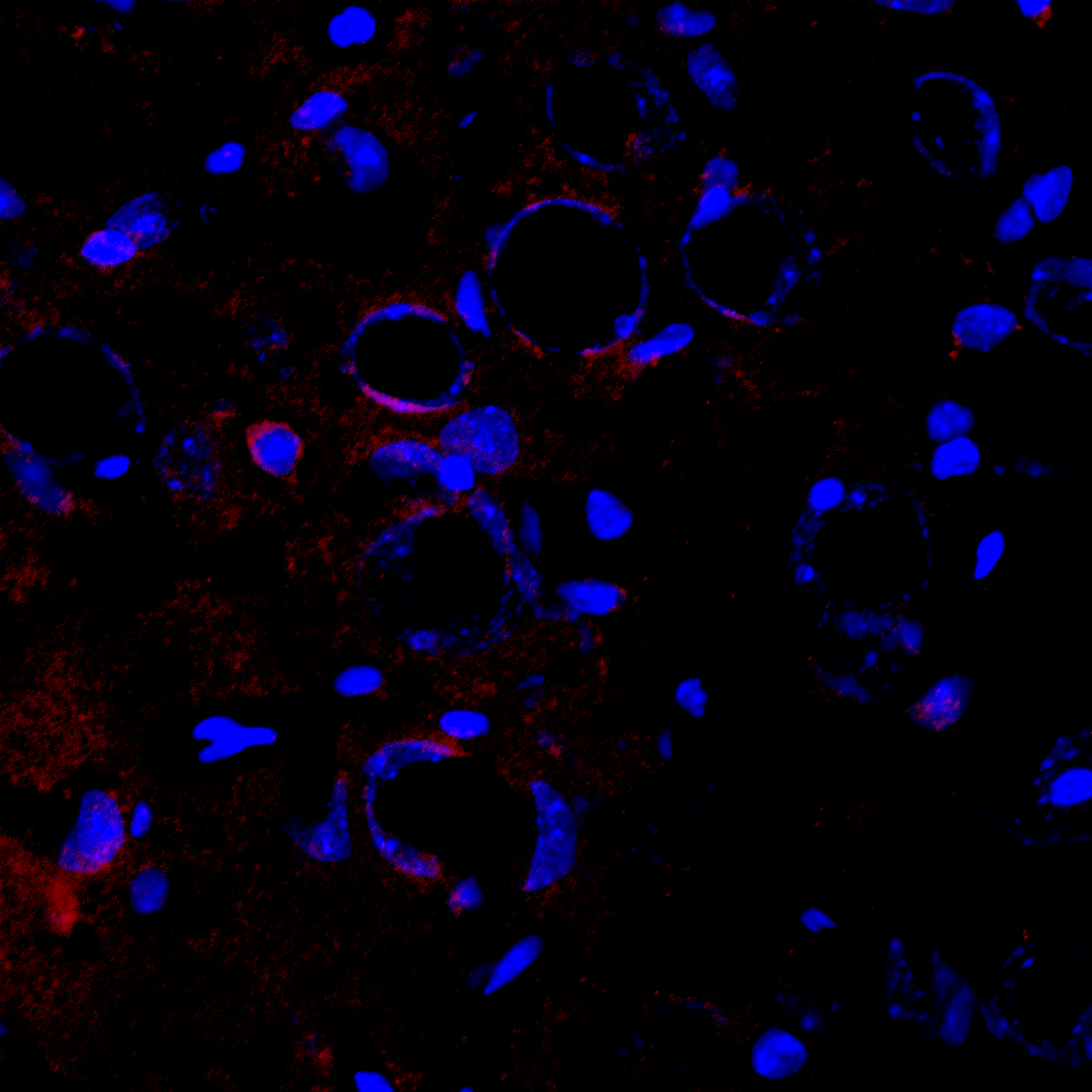

Supplement: Supplementary file 8 — Source data Fig. 6 [file 44319_2025_403_MOESM8_ESM.zip › Figure 6/6B/BLA/overlay 2.tif]

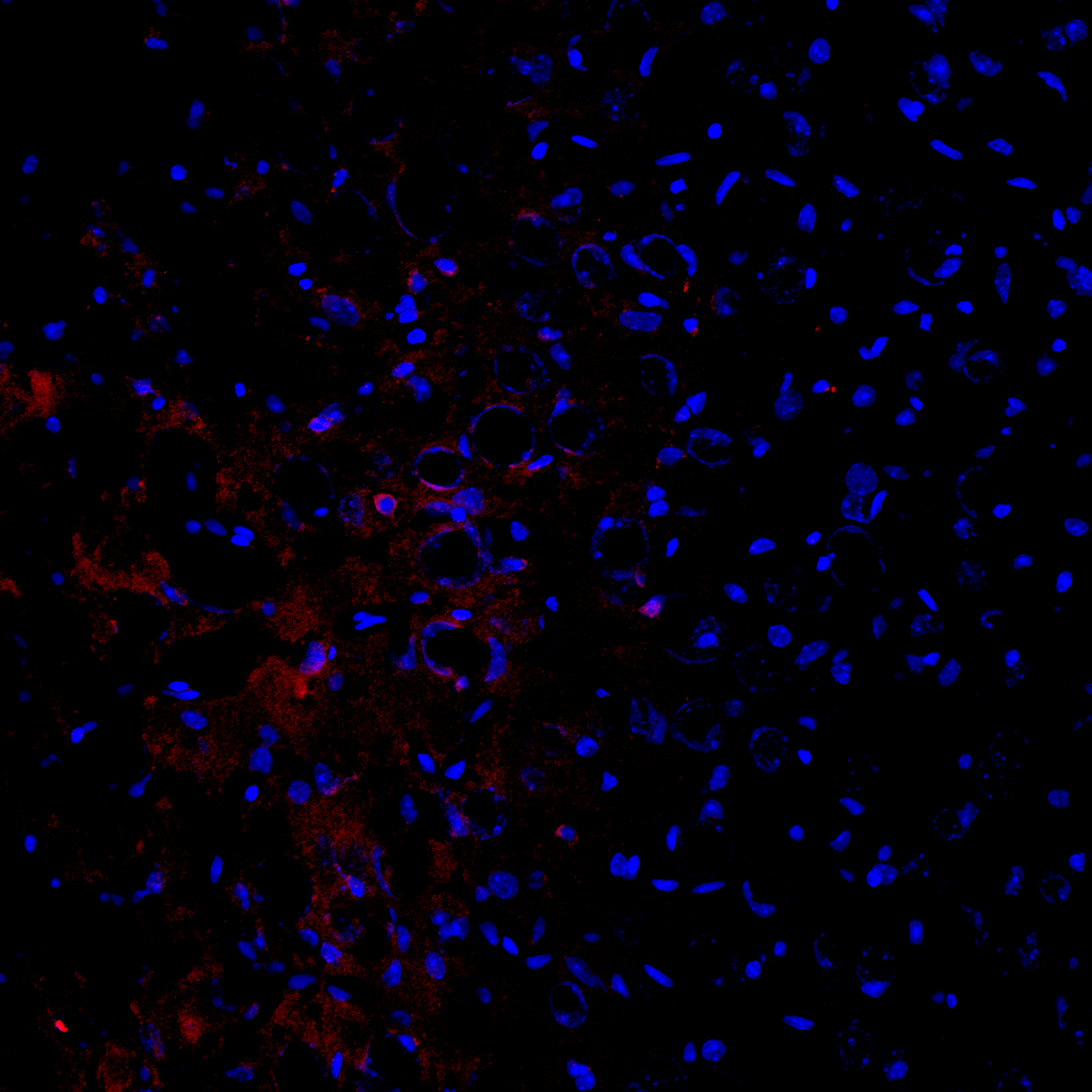

Supplement: Supplementary file 8 — Source data Fig. 6 [file 44319_2025_403_MOESM8_ESM.zip › Figure 6/6B/BLA/overlay 1.tif]

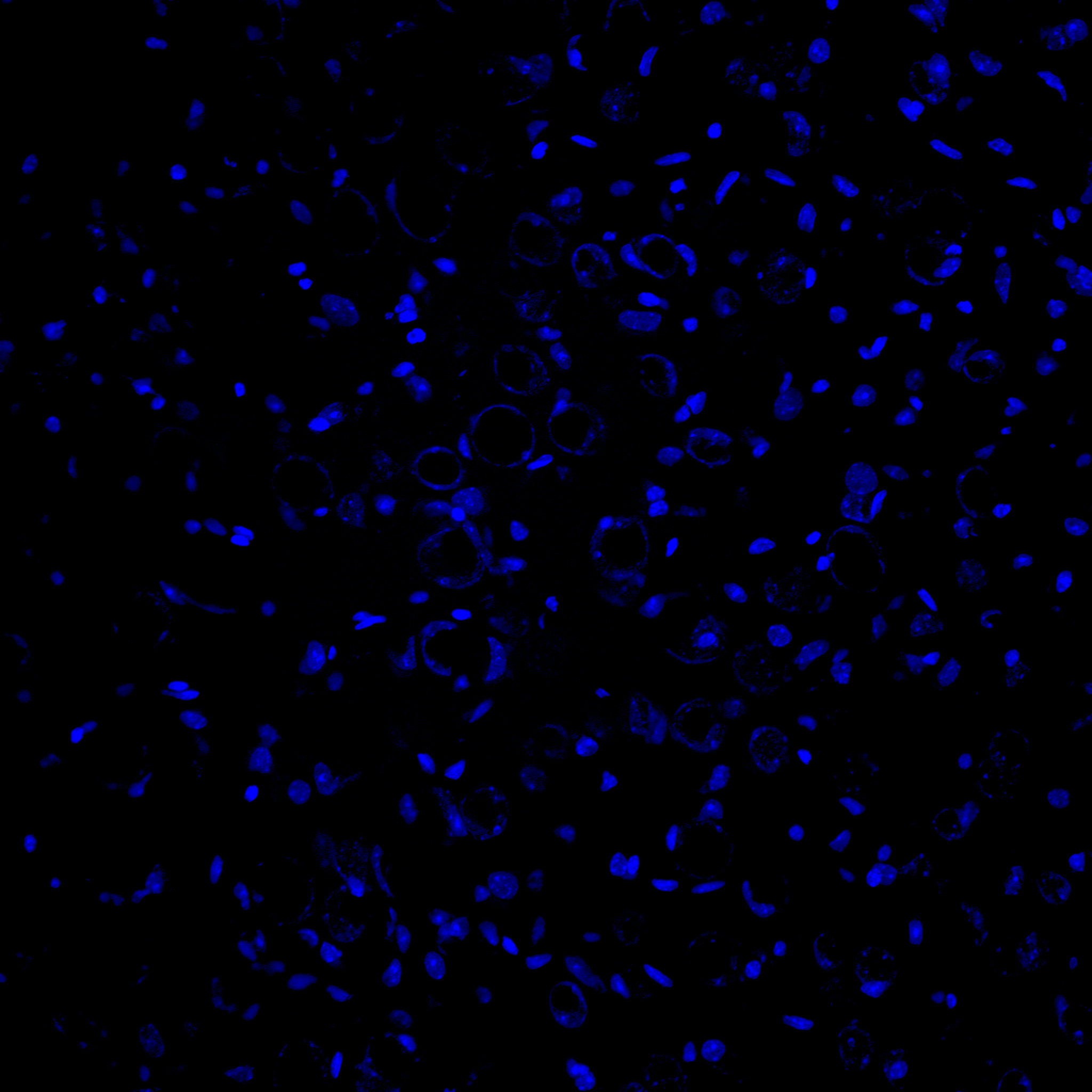

Supplement: Supplementary file 8 — Source data Fig. 6 [file 44319_2025_403_MOESM8_ESM.zip › Figure 6/6B/BLA/Hoechst.tif]

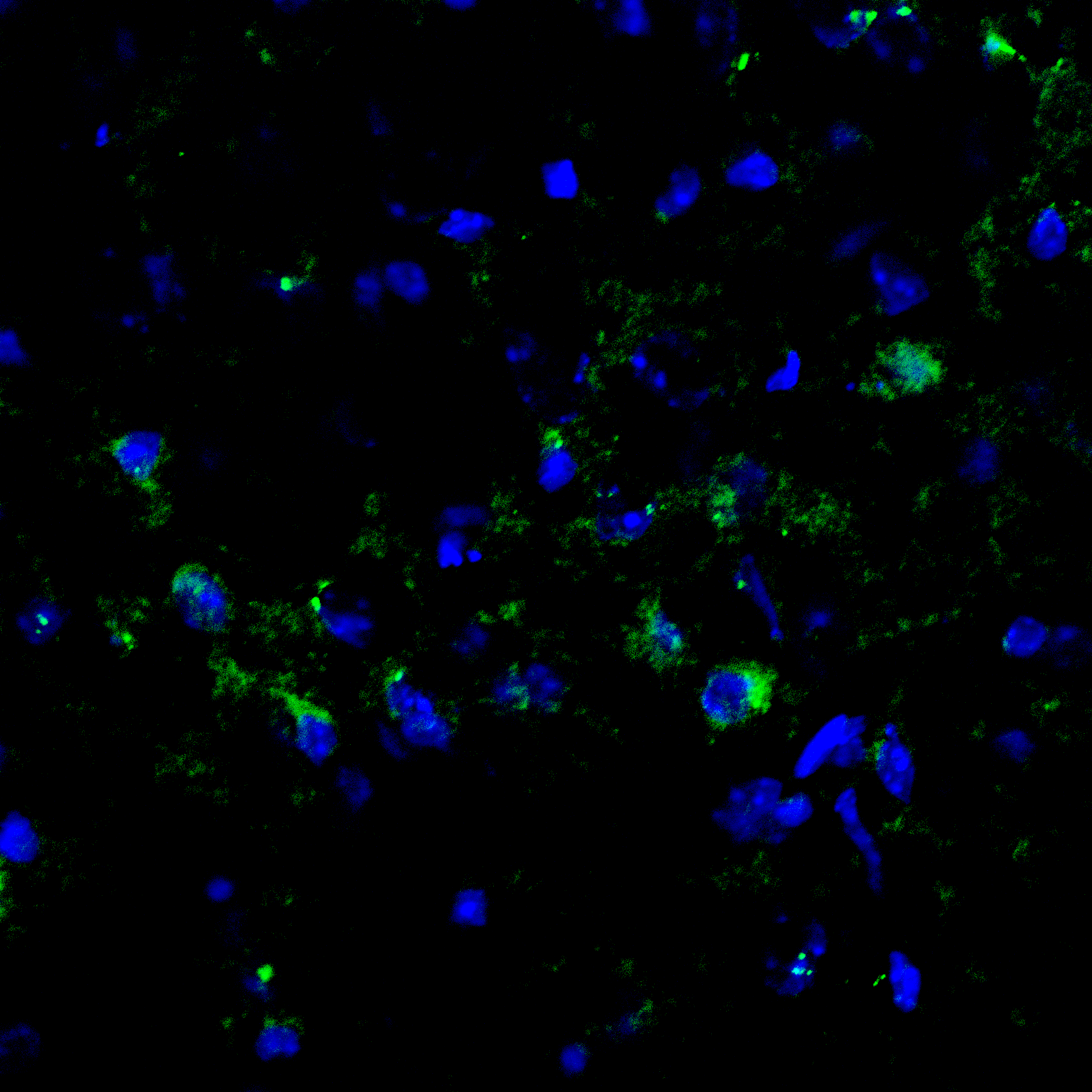

Supplement: Supplementary file 8 — Source data Fig. 6 [file 44319_2025_403_MOESM8_ESM.zip › Figure 6/6B/LS/overlay 2.tif]

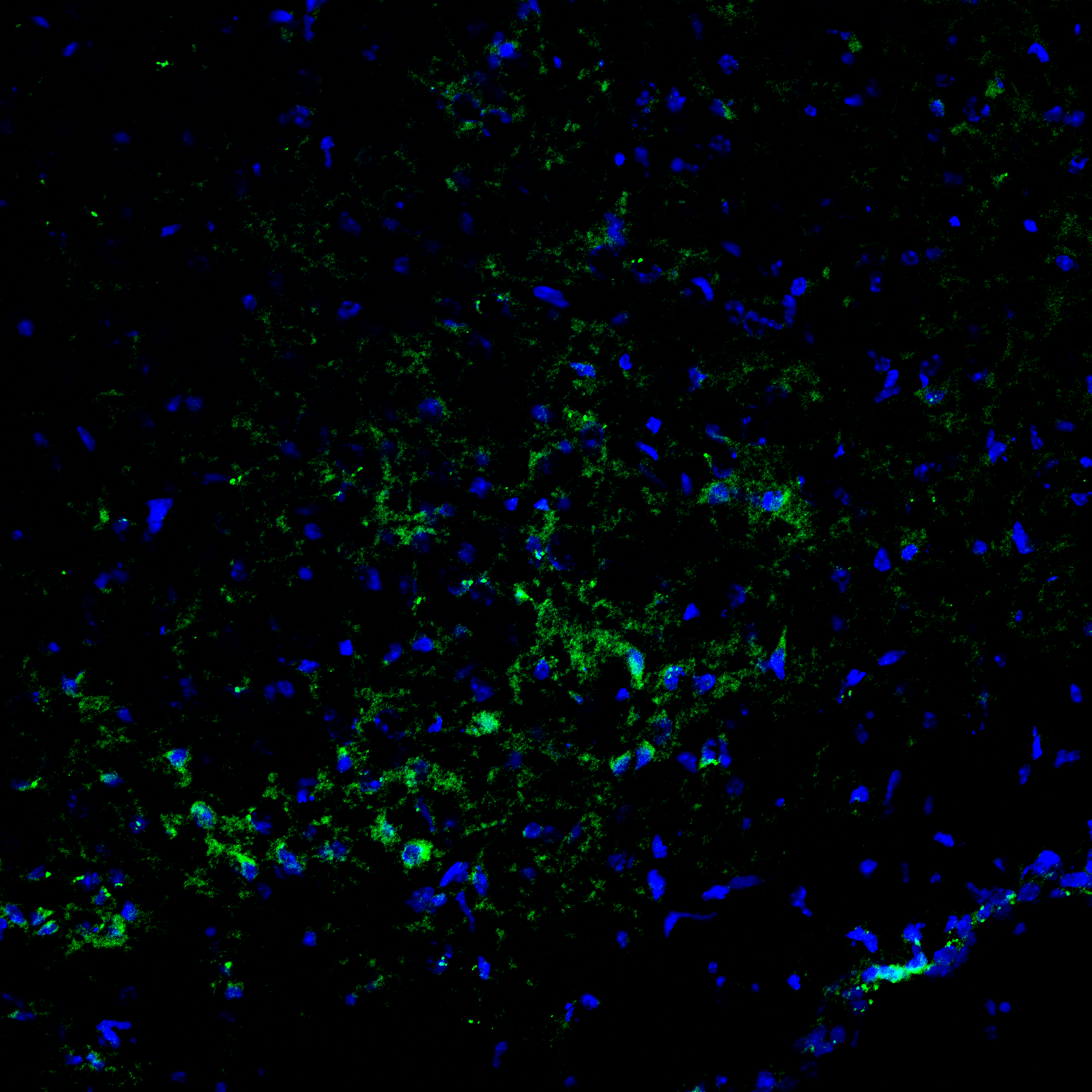

Supplement: Supplementary file 8 — Source data Fig. 6 [file 44319_2025_403_MOESM8_ESM.zip › Figure 6/6B/LS/overlay 1.tif]

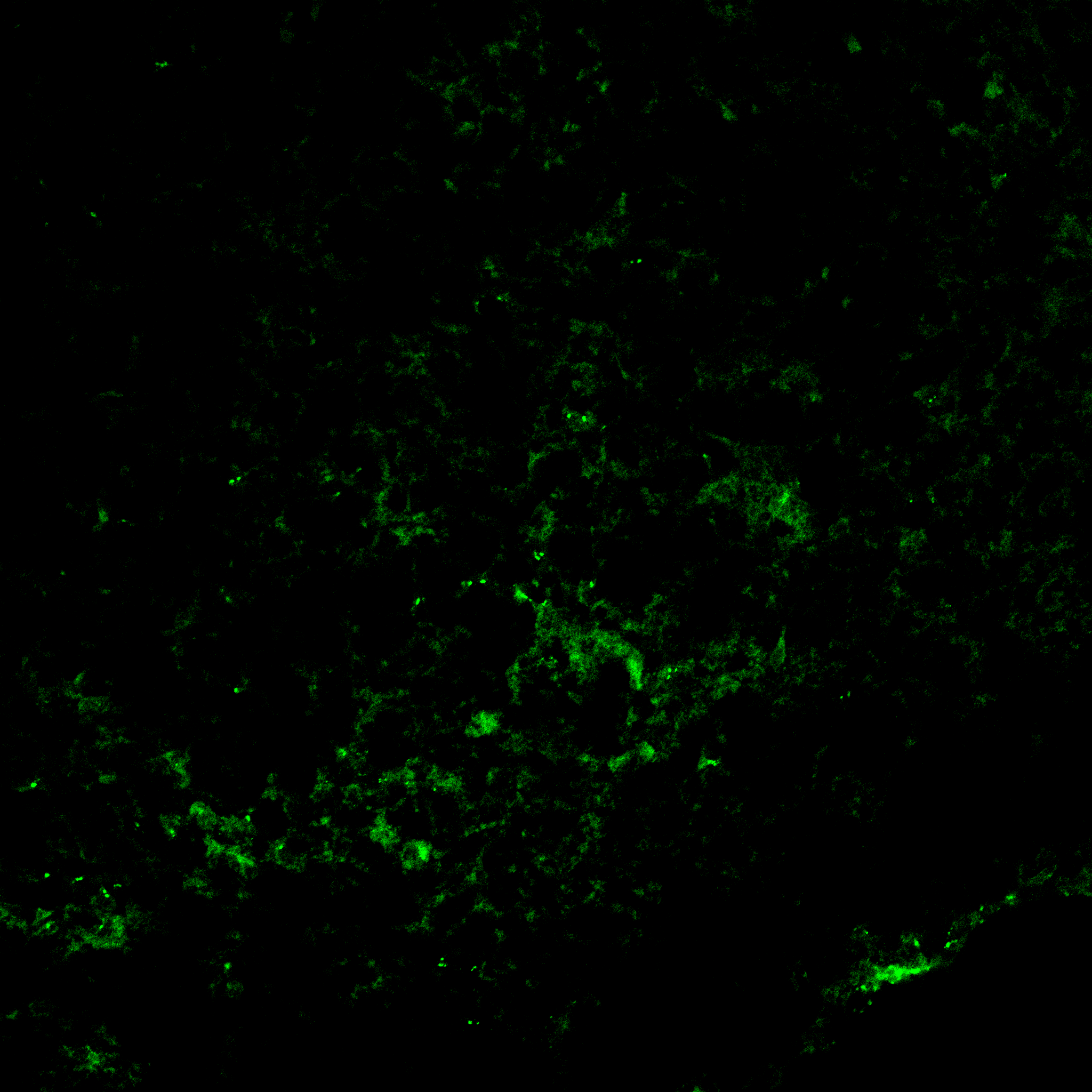

Supplement: Supplementary file 8 — Source data Fig. 6 [file 44319_2025_403_MOESM8_ESM.zip › Figure 6/6B/LS/Cre.tif]
