# Supplementary figures and images for: Auditory fear memory retrieval requires BLA-LS and LS-VMH circuitries via GABAergic and dopaminergic neurons (part 2 of 2)
Source: EMBO Rep. 2025 Mar 7;26(7):1816–34. doi: 10.1038/s44319-025-00403-x (PMC11977213; doi:10.1038/s44319-025-00403-x)

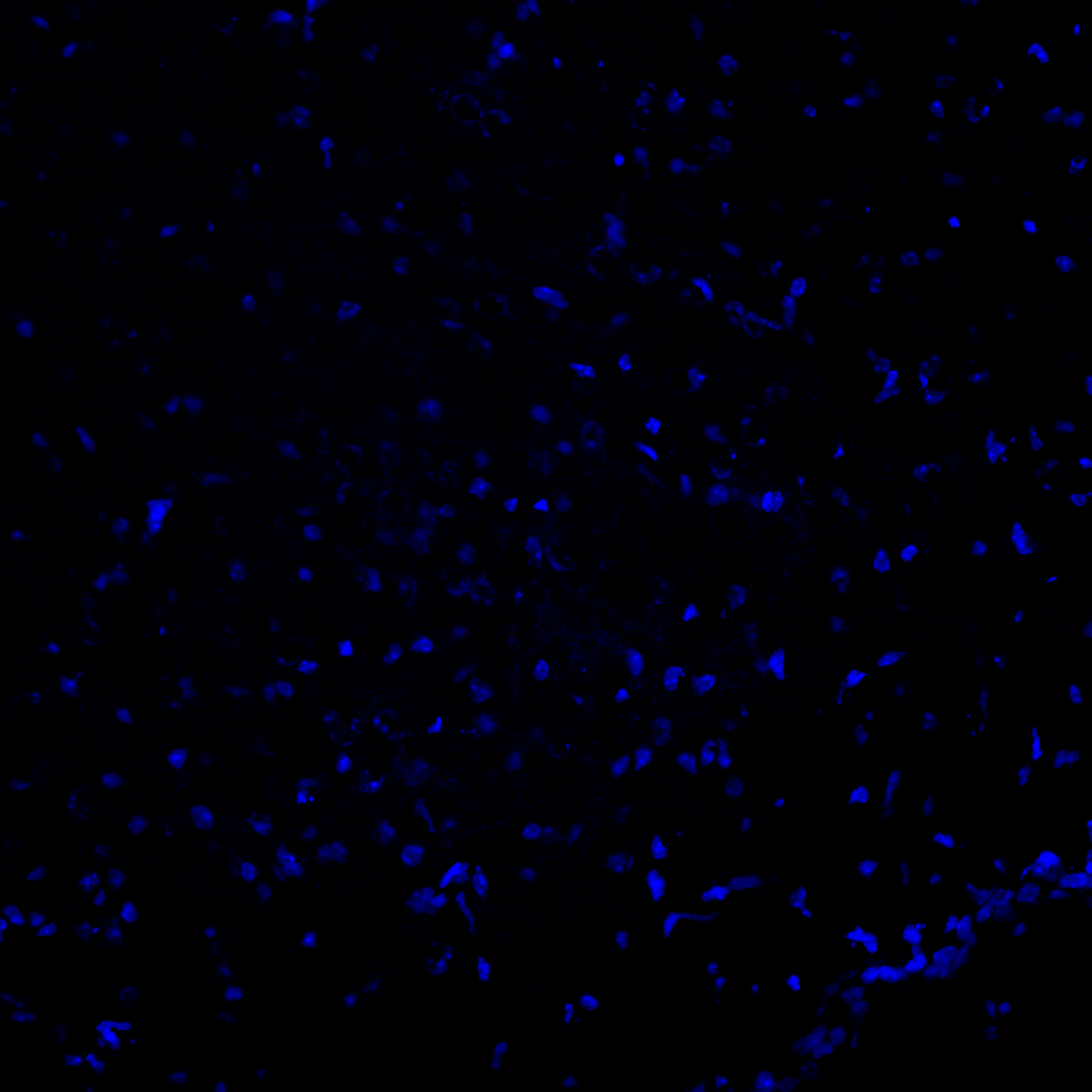

Supplement: Supplementary file 8 — Source data Fig. 6 [file 44319_2025_403_MOESM8_ESM.zip › Figure 6/6B/LS/Hoechst.tif]

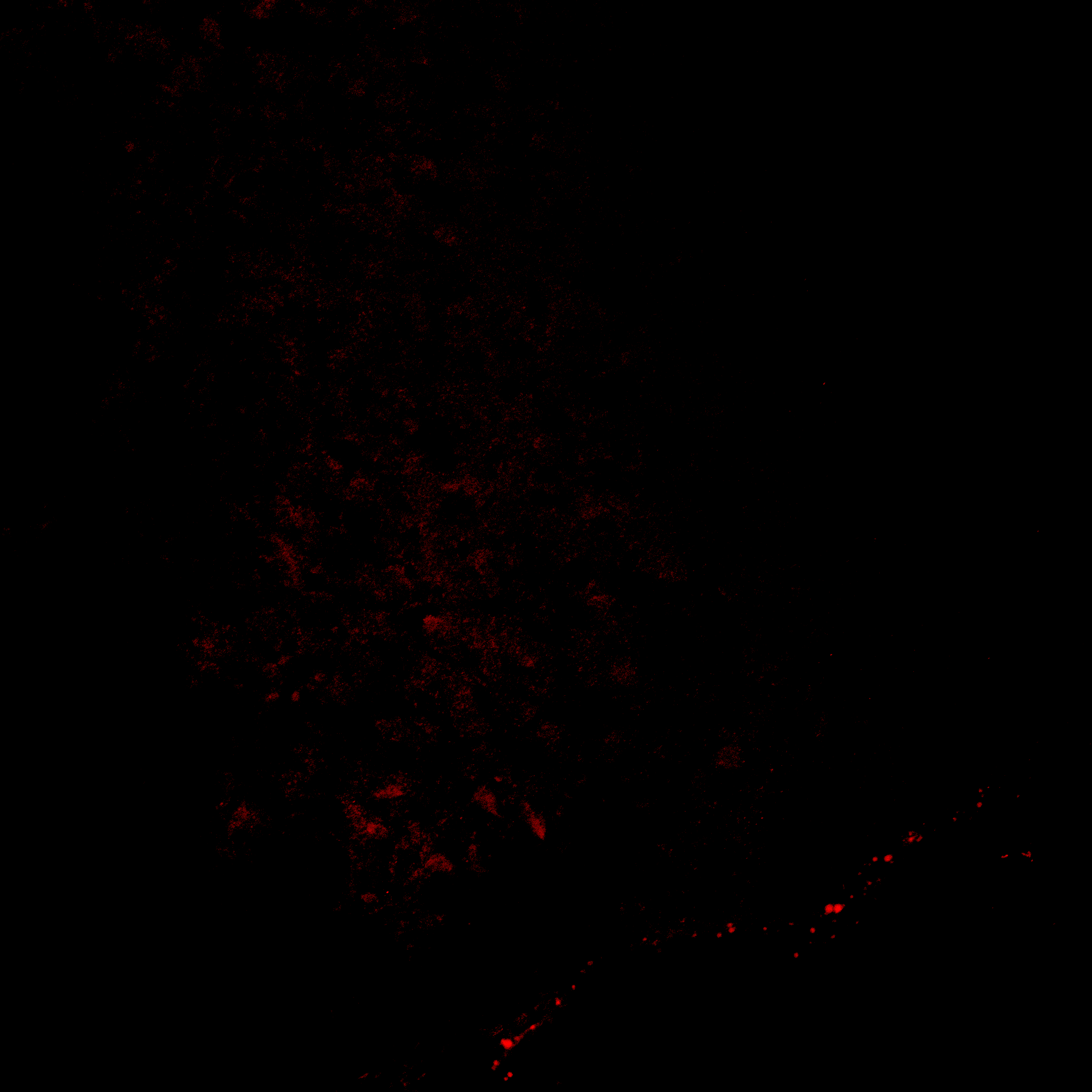

Supplement: Supplementary file 8 — Source data Fig. 6 [file 44319_2025_403_MOESM8_ESM.zip › Figure 6/6C/LS/mCherry.tif]

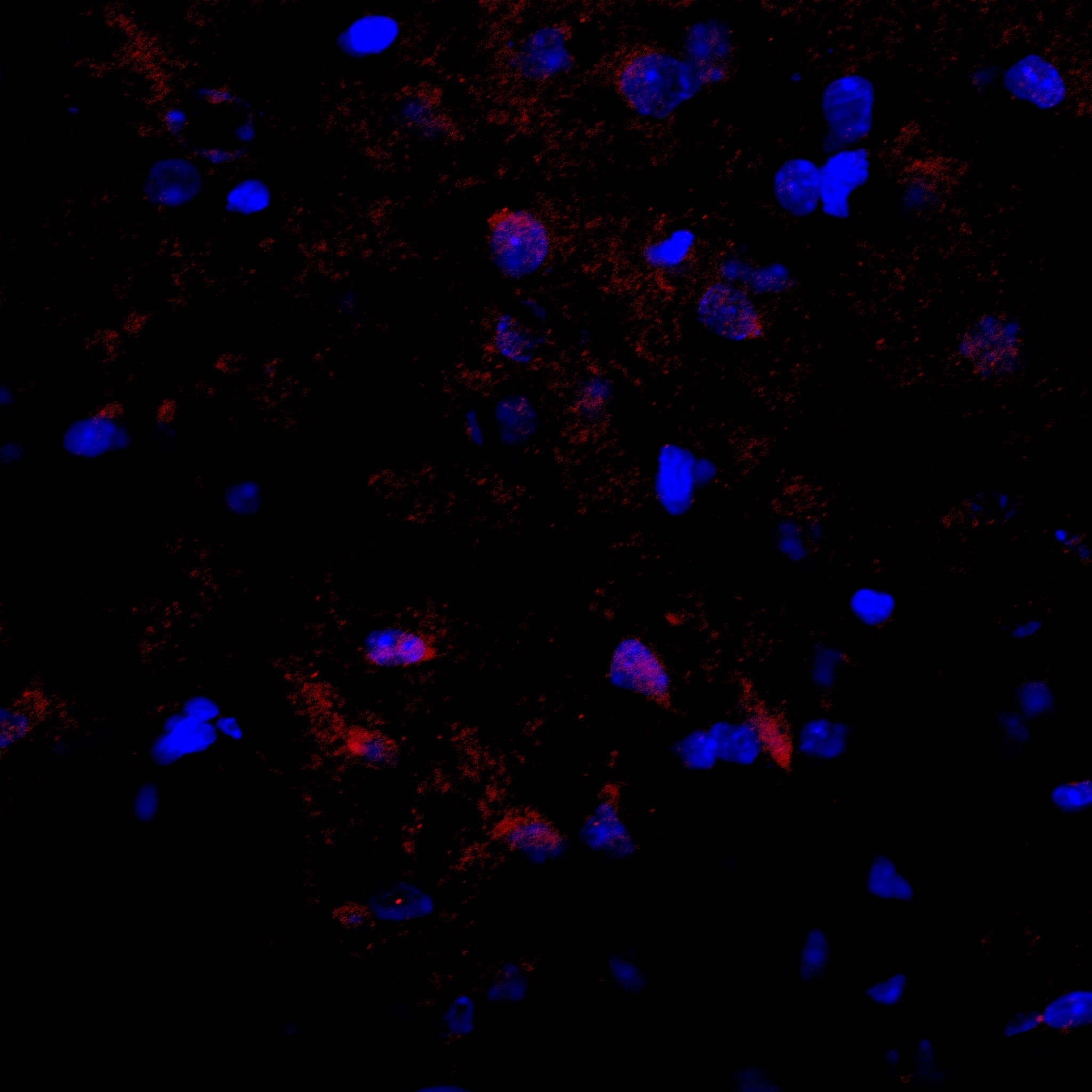

Supplement: Supplementary file 8 — Source data Fig. 6 [file 44319_2025_403_MOESM8_ESM.zip › Figure 6/6C/LS/overlay 2.tif]

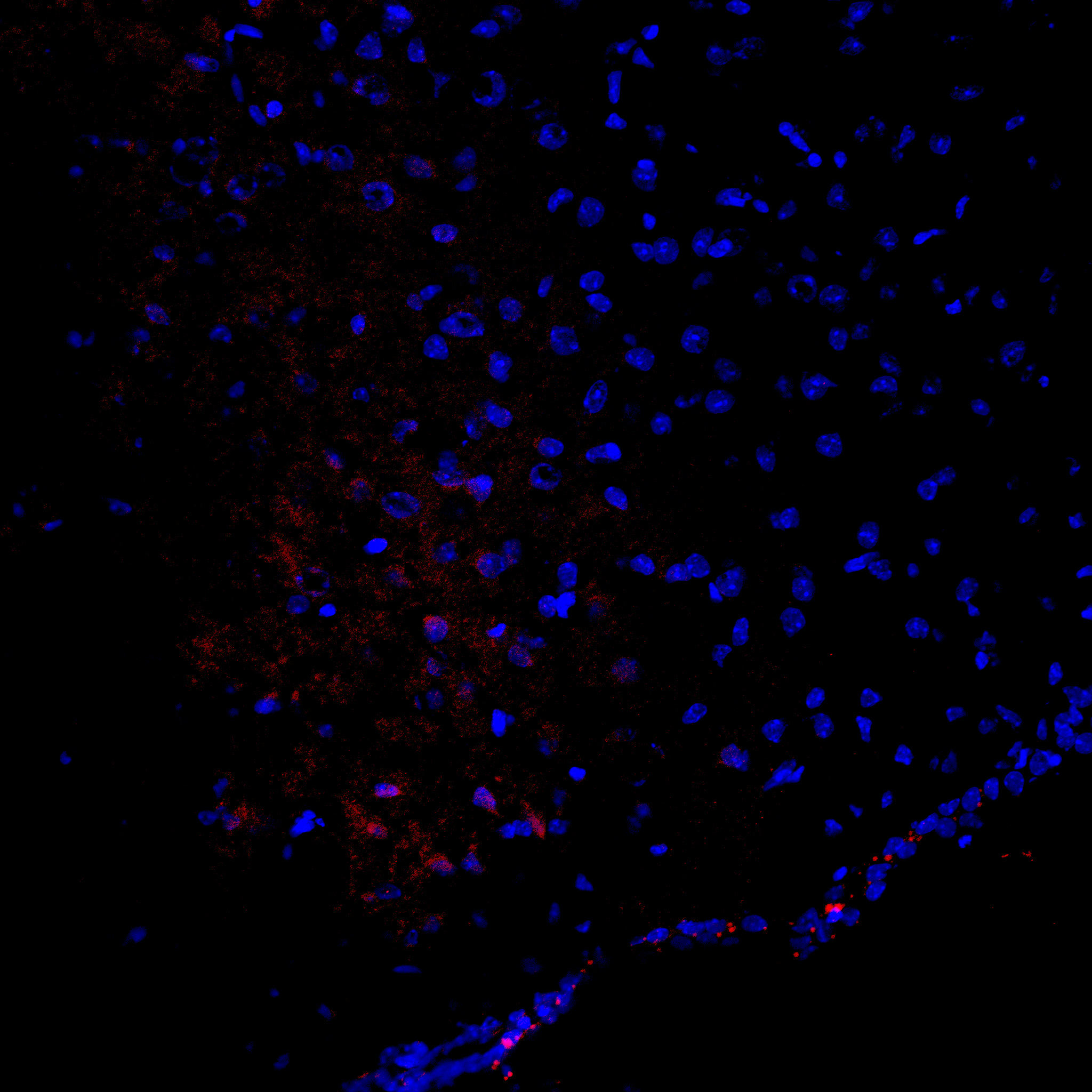

Supplement: Supplementary file 8 — Source data Fig. 6 [file 44319_2025_403_MOESM8_ESM.zip › Figure 6/6C/LS/overlay.tif]

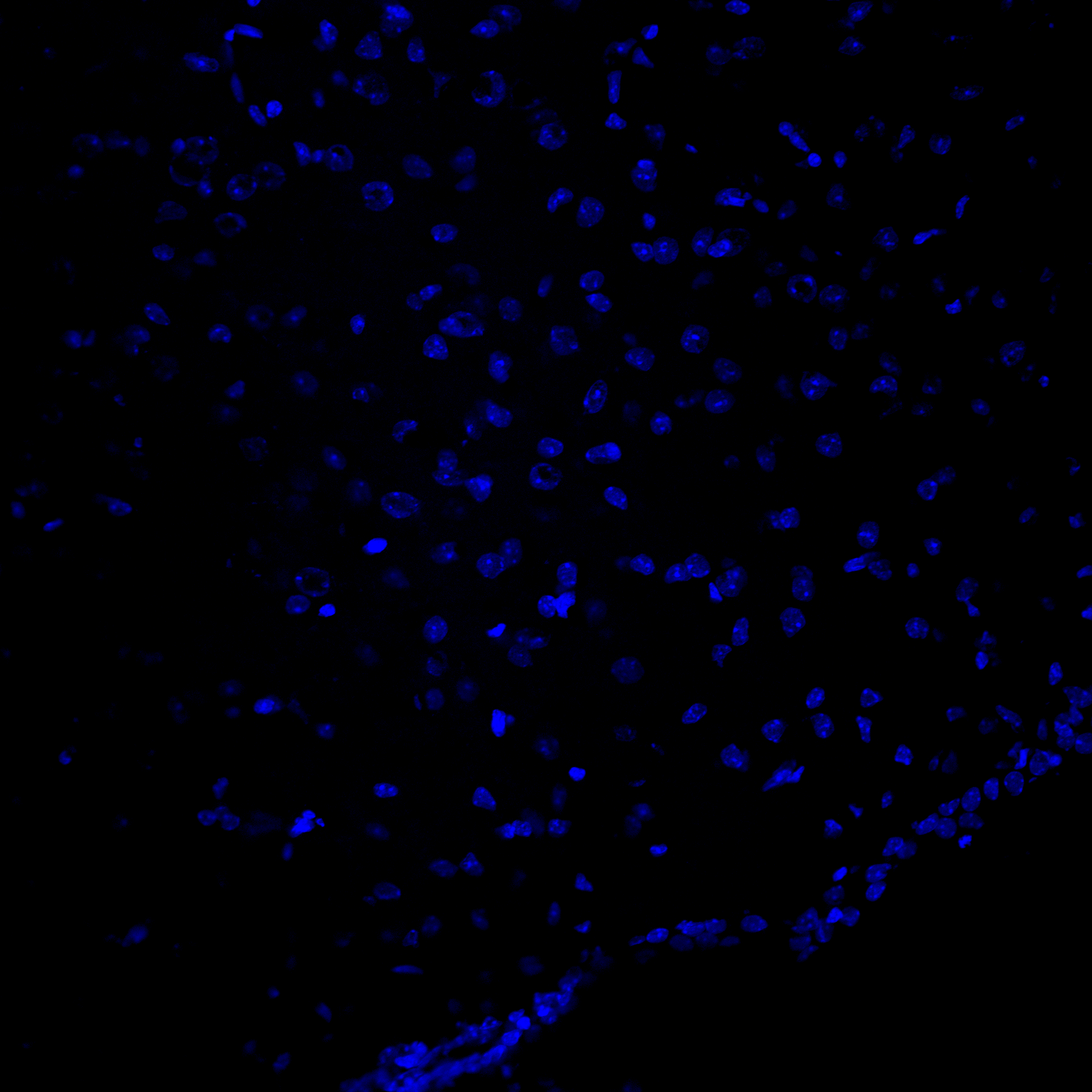

Supplement: Supplementary file 8 — Source data Fig. 6 [file 44319_2025_403_MOESM8_ESM.zip › Figure 6/6C/LS/Hoechst.tif]

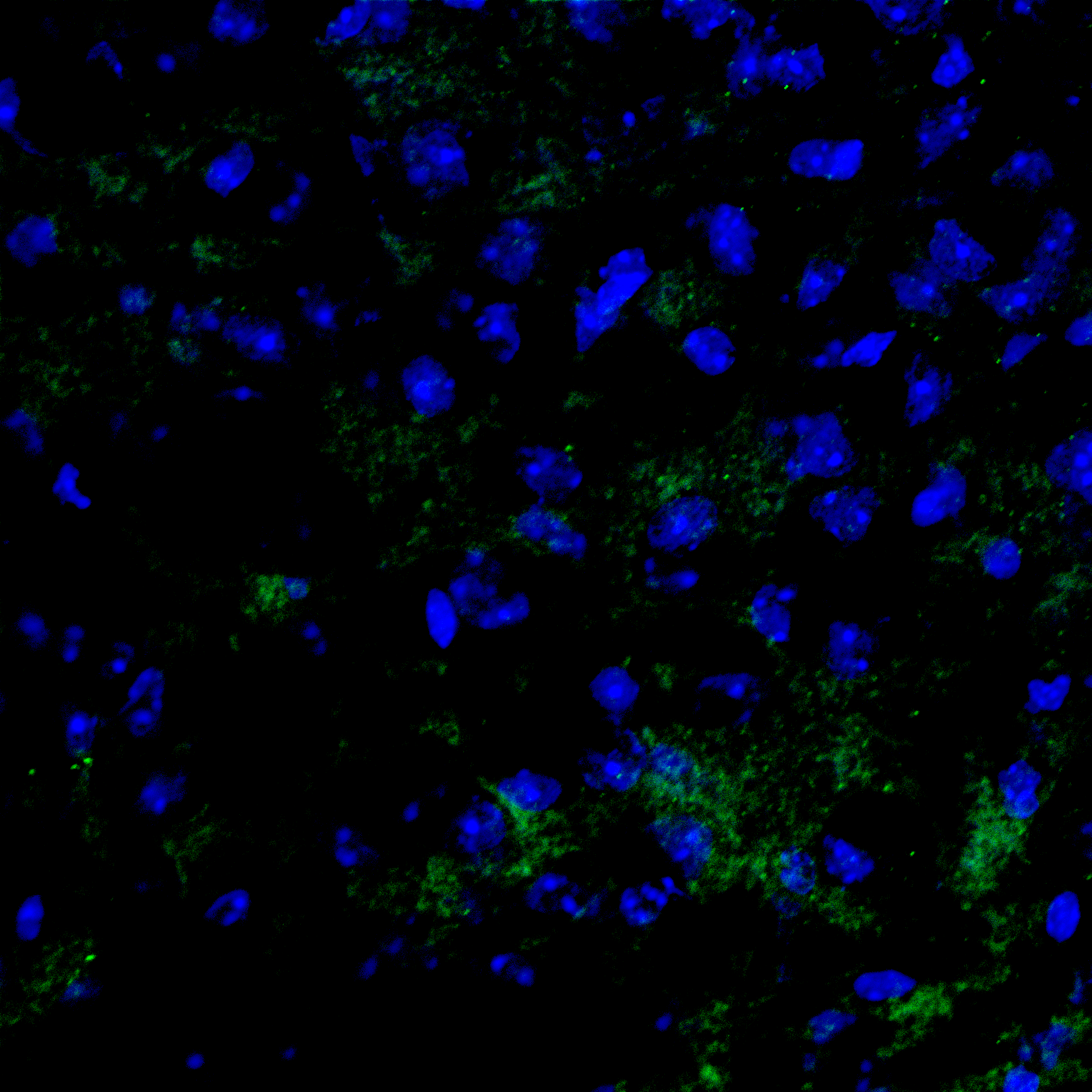

Supplement: Supplementary file 8 — Source data Fig. 6 [file 44319_2025_403_MOESM8_ESM.zip › Figure 6/6C/VMH/overlay 2.tif]

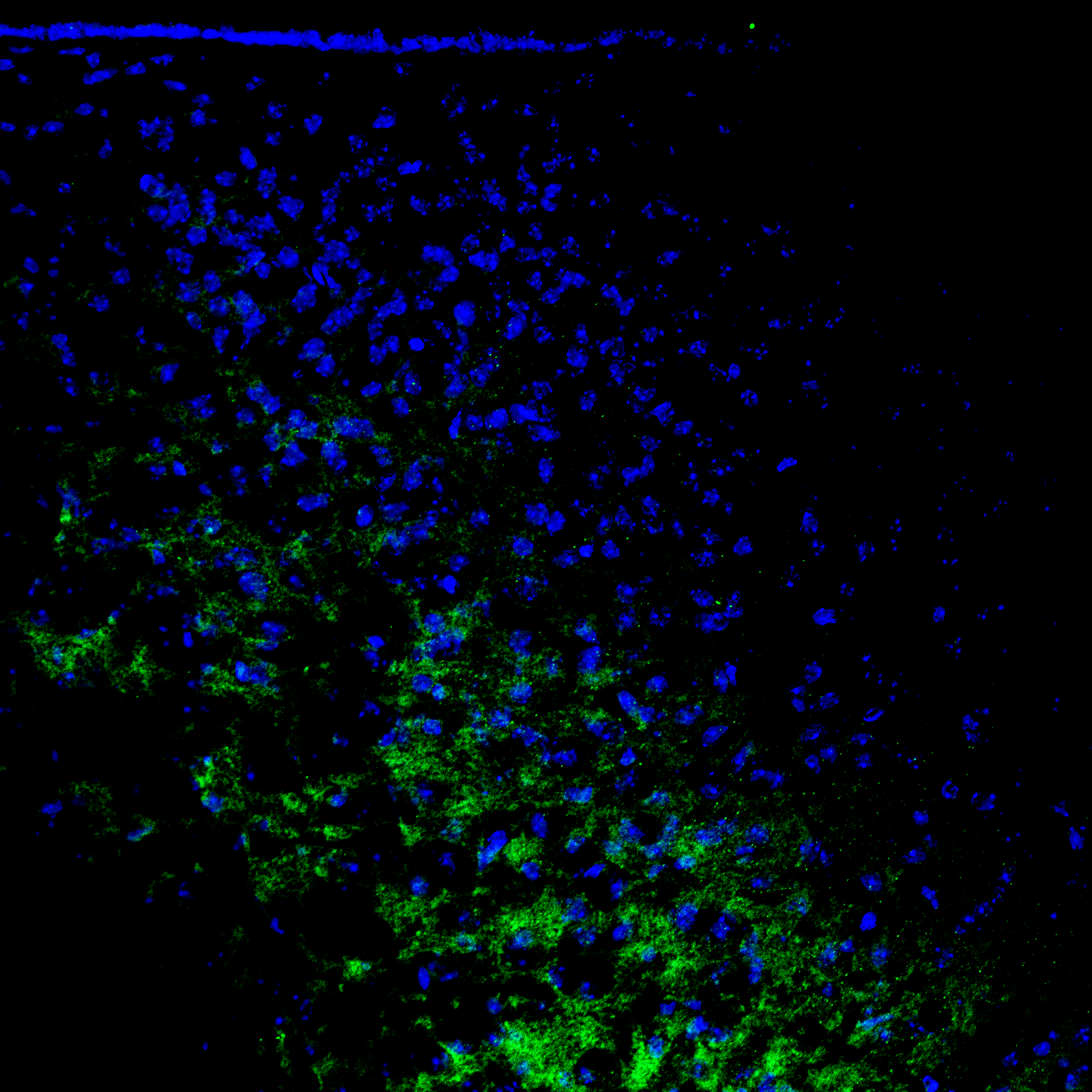

Supplement: Supplementary file 8 — Source data Fig. 6 [file 44319_2025_403_MOESM8_ESM.zip › Figure 6/6C/VMH/overlay 1.tif]

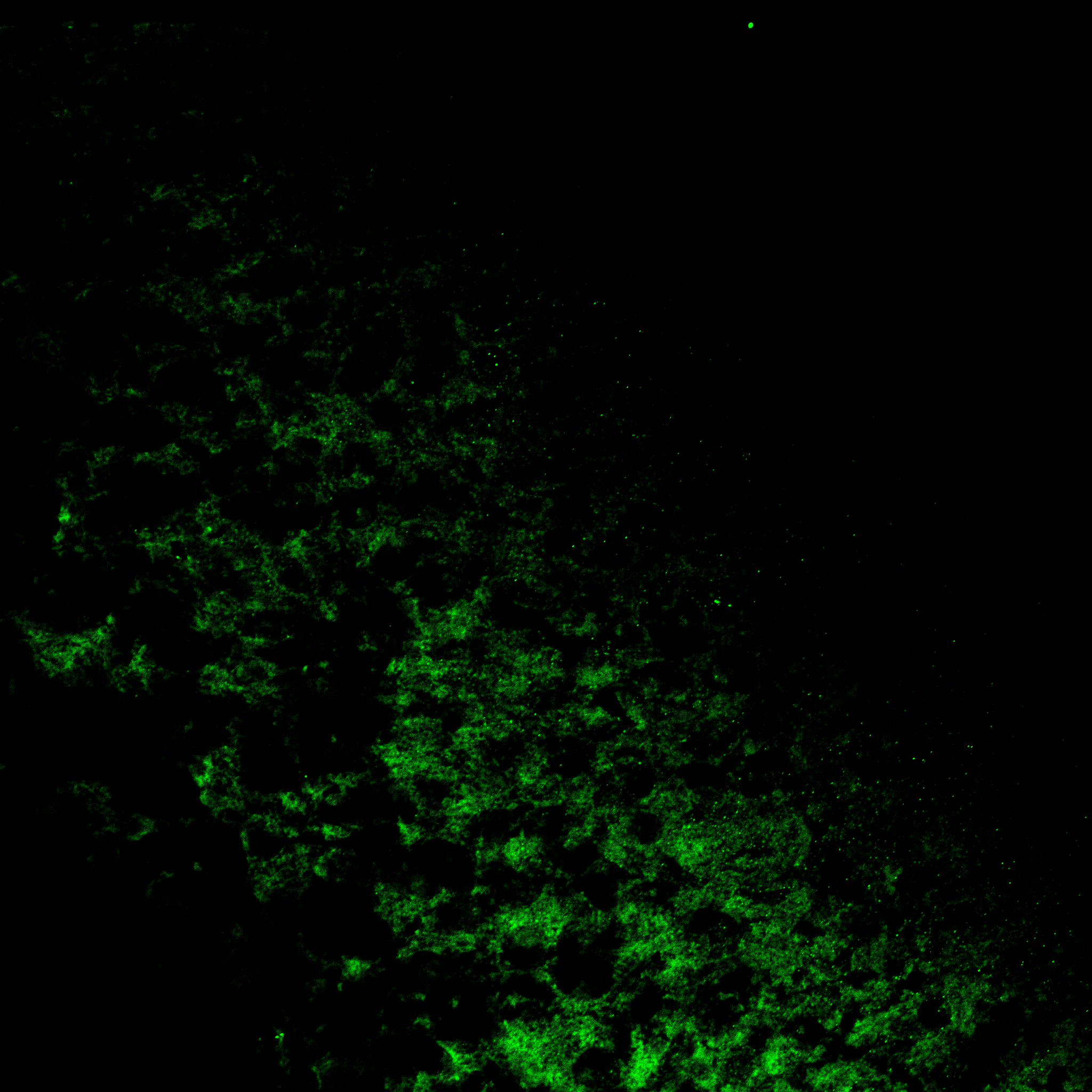

Supplement: Supplementary file 8 — Source data Fig. 6 [file 44319_2025_403_MOESM8_ESM.zip › Figure 6/6C/VMH/Cre .tif]

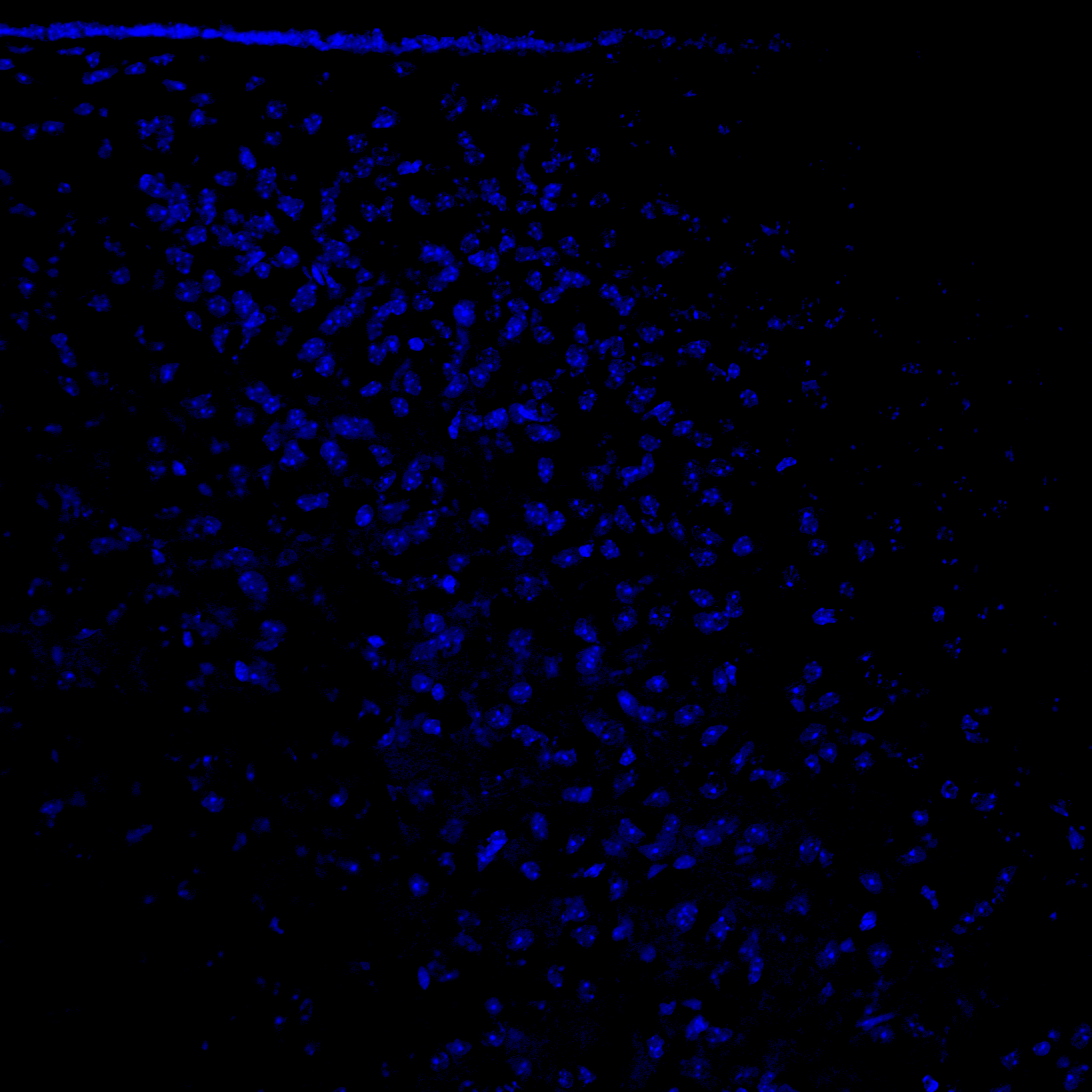

Supplement: Supplementary file 8 — Source data Fig. 6 [file 44319_2025_403_MOESM8_ESM.zip › Figure 6/6C/VMH/Hoechst.tif]

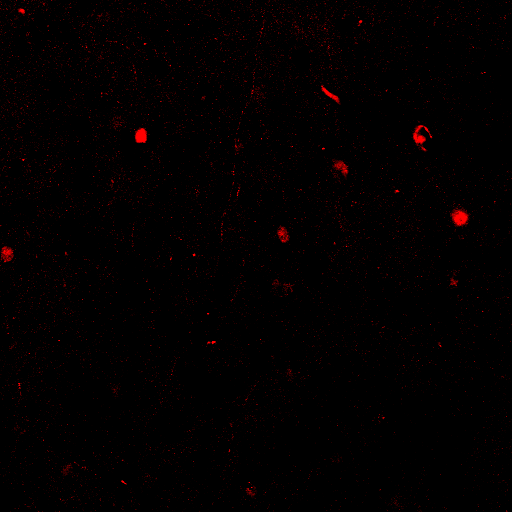

Supplement: Supplementary file 9 — Source data Fig. 7 [file 44319_2025_403_MOESM9_ESM.zip › Figure 7/7G/Context /c-Fos.tif]

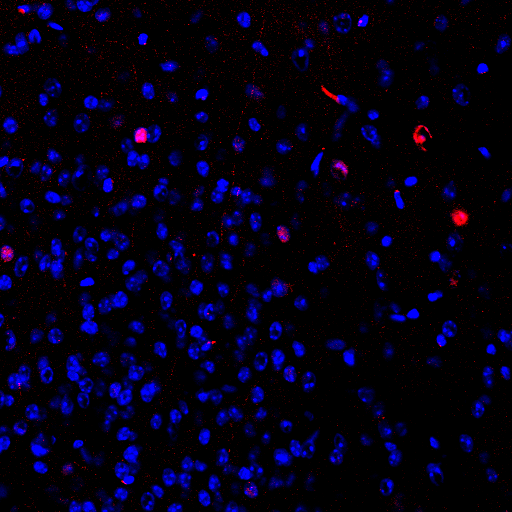

Supplement: Supplementary file 9 — Source data Fig. 7 [file 44319_2025_403_MOESM9_ESM.zip › Figure 7/7G/Context /overlay.tif]

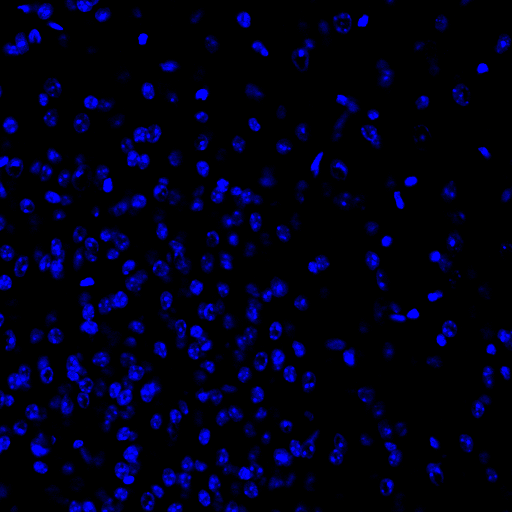

Supplement: Supplementary file 9 — Source data Fig. 7 [file 44319_2025_403_MOESM9_ESM.zip › Figure 7/7G/Context /Hoechst.tif]

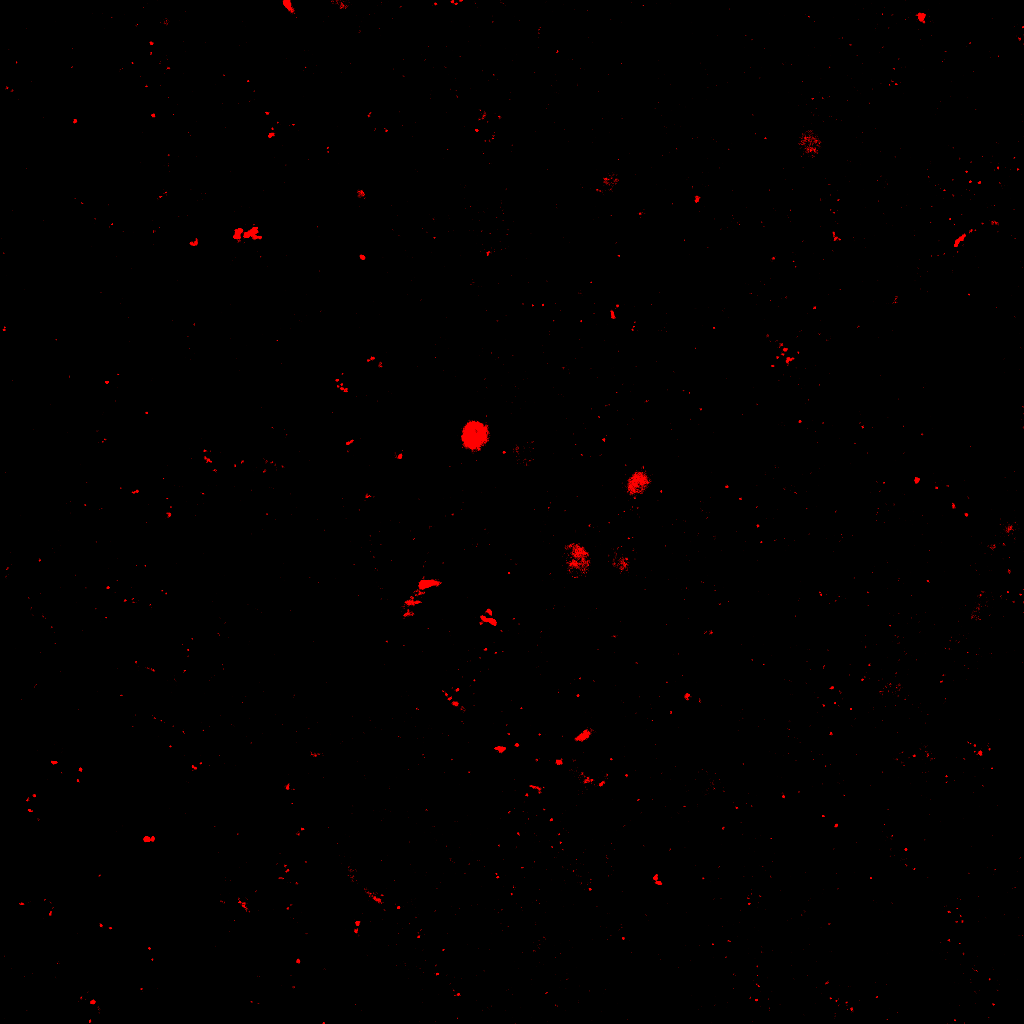

Supplement: Supplementary file 9 — Source data Fig. 7 [file 44319_2025_403_MOESM9_ESM.zip › Figure 7/7G/Control/c-Fos.tif]

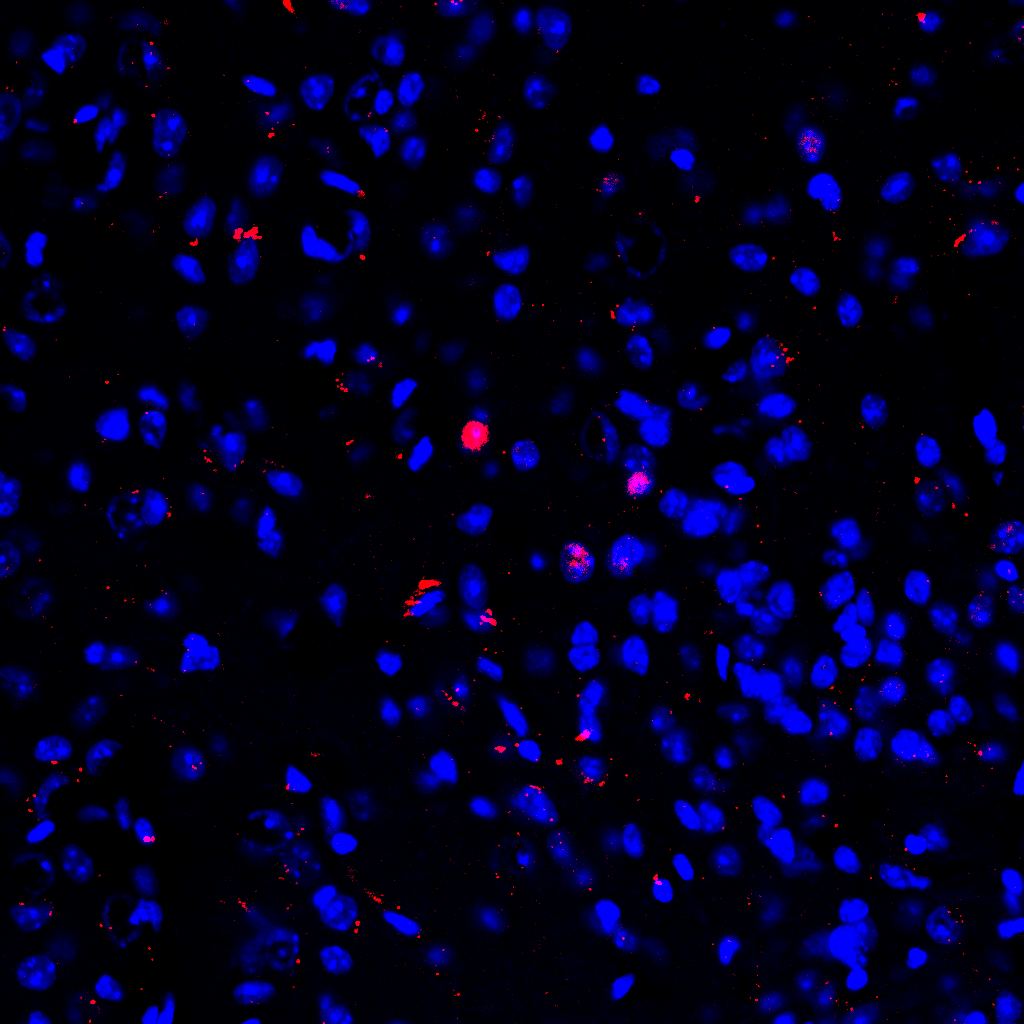

Supplement: Supplementary file 9 — Source data Fig. 7 [file 44319_2025_403_MOESM9_ESM.zip › Figure 7/7G/Control/overlay.tif]

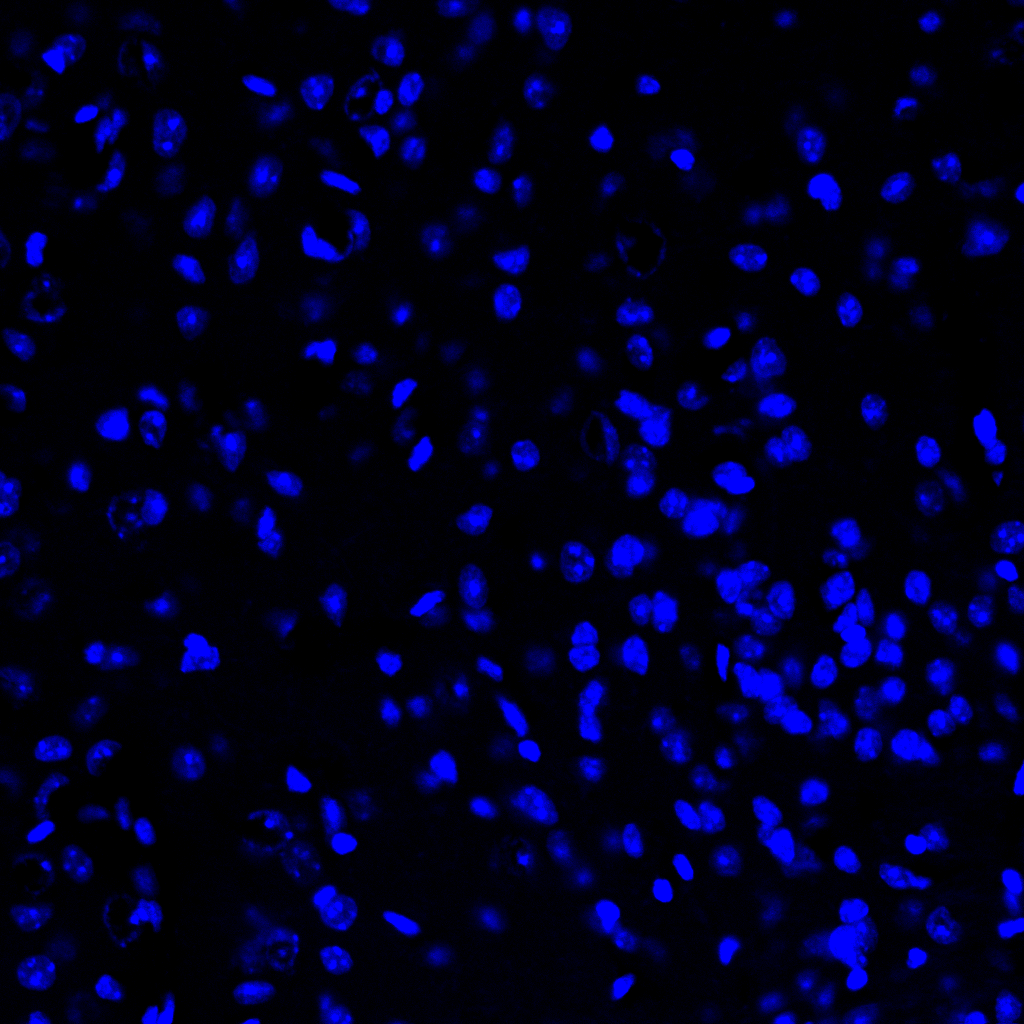

Supplement: Supplementary file 9 — Source data Fig. 7 [file 44319_2025_403_MOESM9_ESM.zip › Figure 7/7G/Control/Hoechst.tif]

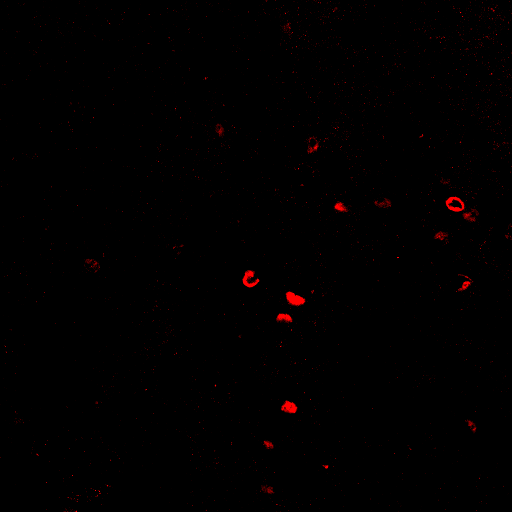

Supplement: Supplementary file 9 — Source data Fig. 7 [file 44319_2025_403_MOESM9_ESM.zip › Figure 7/7G/TMT/c-Fos.tif]

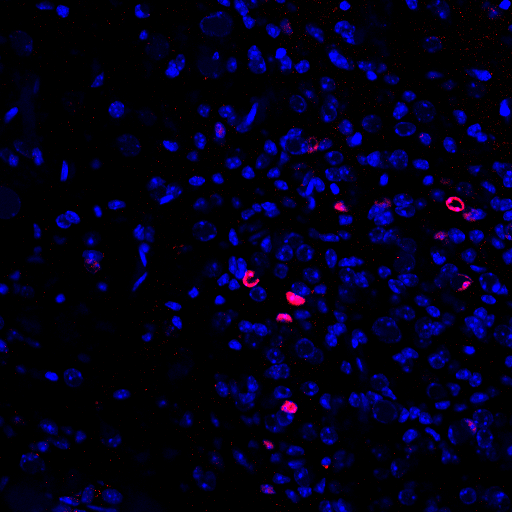

Supplement: Supplementary file 9 — Source data Fig. 7 [file 44319_2025_403_MOESM9_ESM.zip › Figure 7/7G/TMT/overlay.tif]

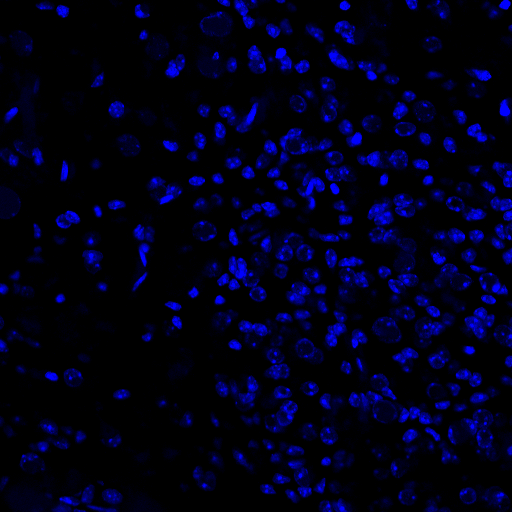

Supplement: Supplementary file 9 — Source data Fig. 7 [file 44319_2025_403_MOESM9_ESM.zip › Figure 7/7G/TMT/Hoechst.tif]

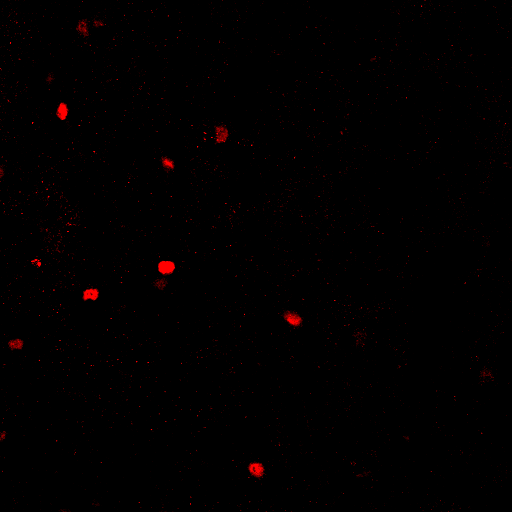

Supplement: Supplementary file 9 — Source data Fig. 7 [file 44319_2025_403_MOESM9_ESM.zip › Figure 7/7H/context/c-Fos.tif]

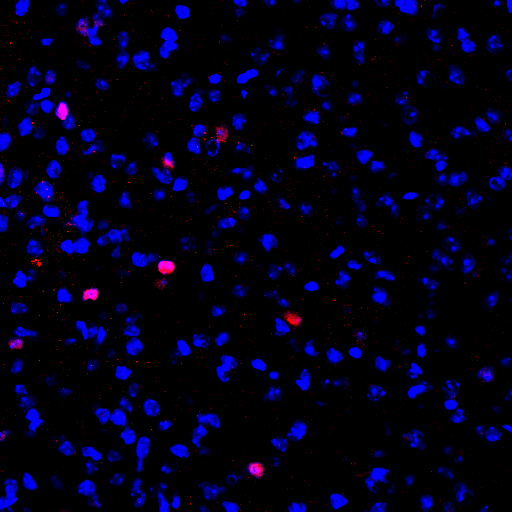

Supplement: Supplementary file 9 — Source data Fig. 7 [file 44319_2025_403_MOESM9_ESM.zip › Figure 7/7H/context/overlay.tif]

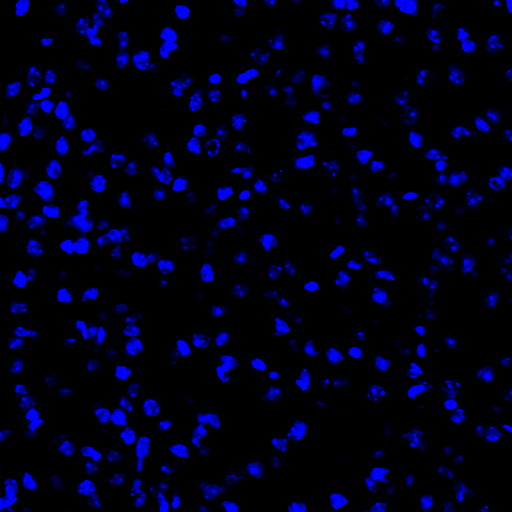

Supplement: Supplementary file 9 — Source data Fig. 7 [file 44319_2025_403_MOESM9_ESM.zip › Figure 7/7H/context/Hoechst.tif]

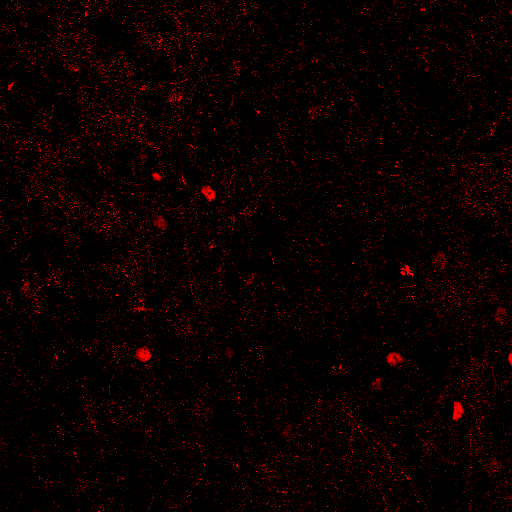

Supplement: Supplementary file 9 — Source data Fig. 7 [file 44319_2025_403_MOESM9_ESM.zip › Figure 7/7H/Control /c-Fos.tif]

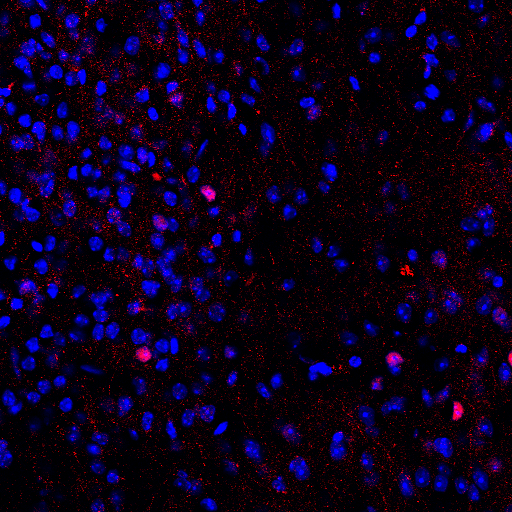

Supplement: Supplementary file 9 — Source data Fig. 7 [file 44319_2025_403_MOESM9_ESM.zip › Figure 7/7H/Control /overlay.tif]

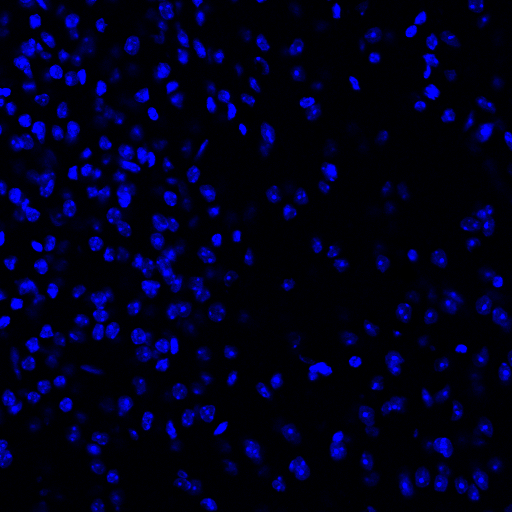

Supplement: Supplementary file 9 — Source data Fig. 7 [file 44319_2025_403_MOESM9_ESM.zip › Figure 7/7H/Control /Hoechst.tif]

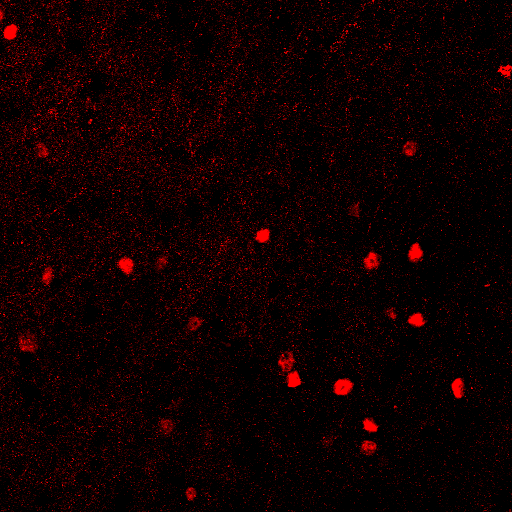

Supplement: Supplementary file 9 — Source data Fig. 7 [file 44319_2025_403_MOESM9_ESM.zip › Figure 7/7H/TMT/c-Fos.tif]

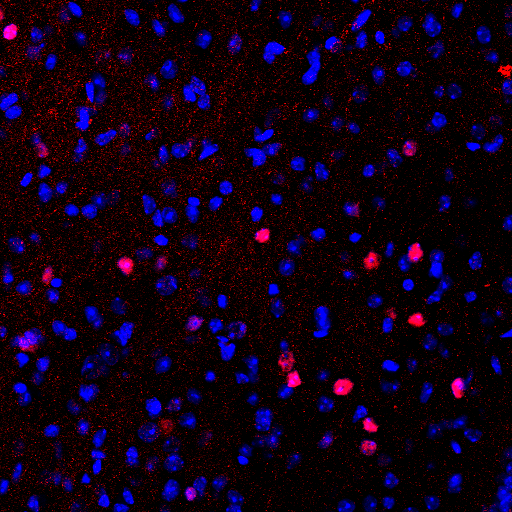

Supplement: Supplementary file 9 — Source data Fig. 7 [file 44319_2025_403_MOESM9_ESM.zip › Figure 7/7H/TMT/overlay.tif]

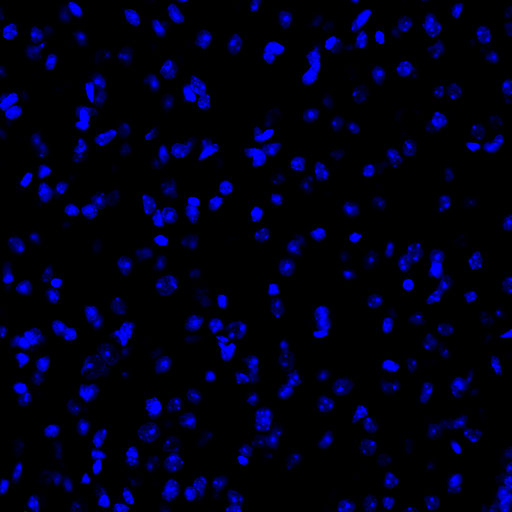

Supplement: Supplementary file 9 — Source data Fig. 7 [file 44319_2025_403_MOESM9_ESM.zip › Figure 7/7H/TMT/Hoechst.tif]

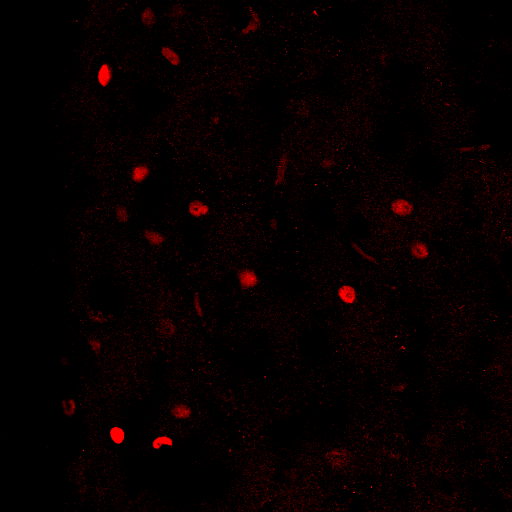

Supplement: Supplementary file 9 — Source data Fig. 7 [file 44319_2025_403_MOESM9_ESM.zip › Figure 7/7F/Context/LSD/c-Fos.tif]

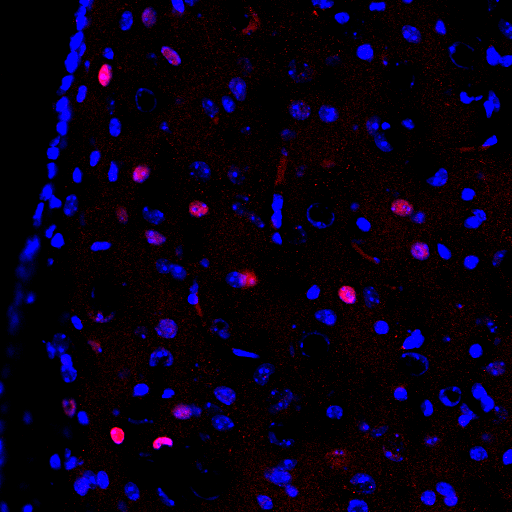

Supplement: Supplementary file 9 — Source data Fig. 7 [file 44319_2025_403_MOESM9_ESM.zip › Figure 7/7F/Context/LSD/overlay.tif]

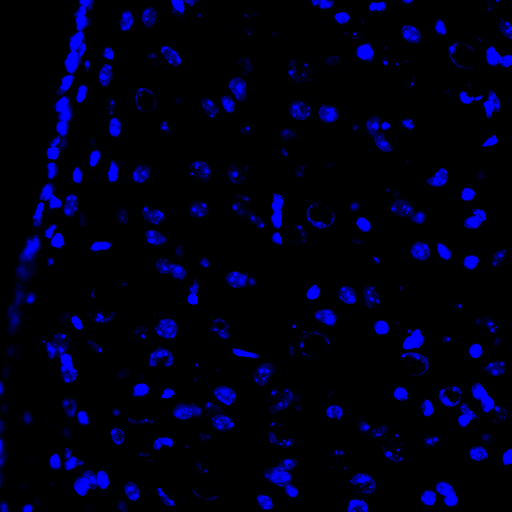

Supplement: Supplementary file 9 — Source data Fig. 7 [file 44319_2025_403_MOESM9_ESM.zip › Figure 7/7F/Context/LSD/Hoechst.tif]

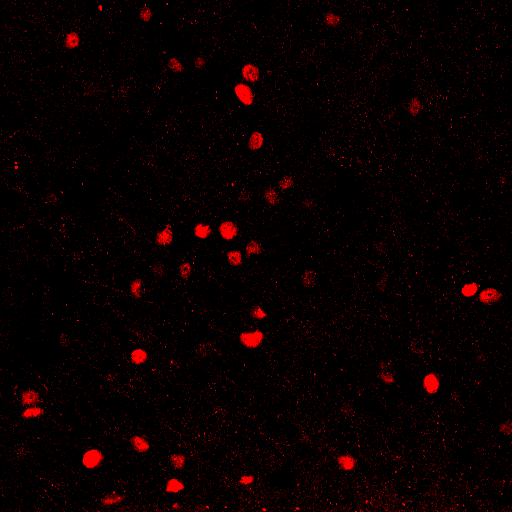

Supplement: Supplementary file 9 — Source data Fig. 7 [file 44319_2025_403_MOESM9_ESM.zip › Figure 7/7F/Context/LSV/c-Fos.tif]

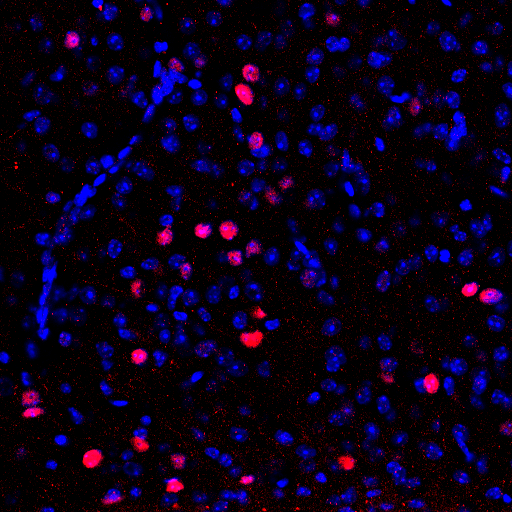

Supplement: Supplementary file 9 — Source data Fig. 7 [file 44319_2025_403_MOESM9_ESM.zip › Figure 7/7F/Context/LSV/overlay.tif]

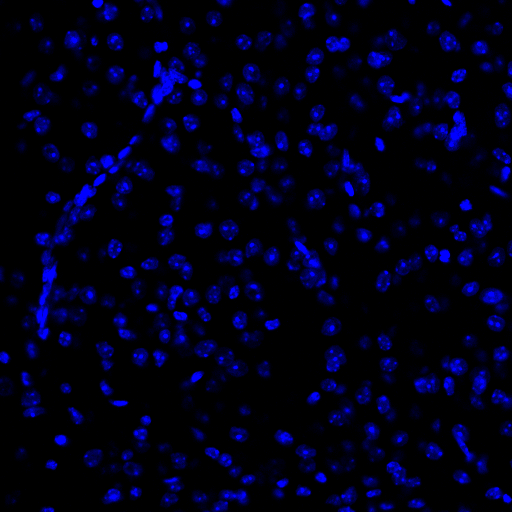

Supplement: Supplementary file 9 — Source data Fig. 7 [file 44319_2025_403_MOESM9_ESM.zip › Figure 7/7F/Context/LSV/Hoechst.tif]

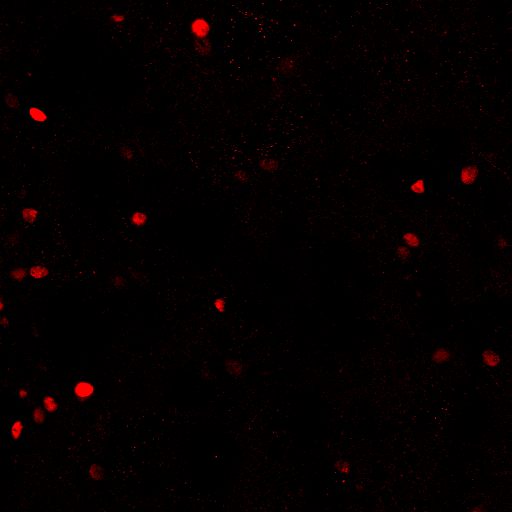

Supplement: Supplementary file 9 — Source data Fig. 7 [file 44319_2025_403_MOESM9_ESM.zip › Figure 7/7F/Context/LSI/c-Fos.tif]

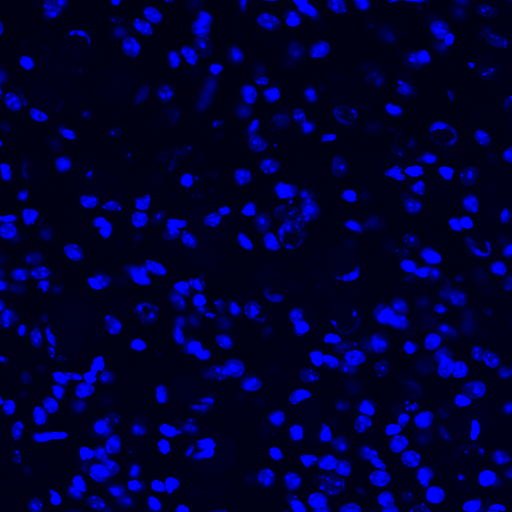

Supplement: Supplementary file 9 — Source data Fig. 7 [file 44319_2025_403_MOESM9_ESM.zip › Figure 7/7F/Context/LSI/Hoschst.tif]

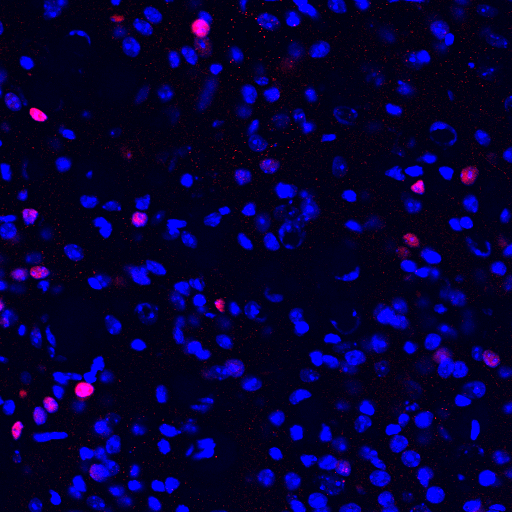

Supplement: Supplementary file 9 — Source data Fig. 7 [file 44319_2025_403_MOESM9_ESM.zip › Figure 7/7F/Context/LSI/overlay.tif]

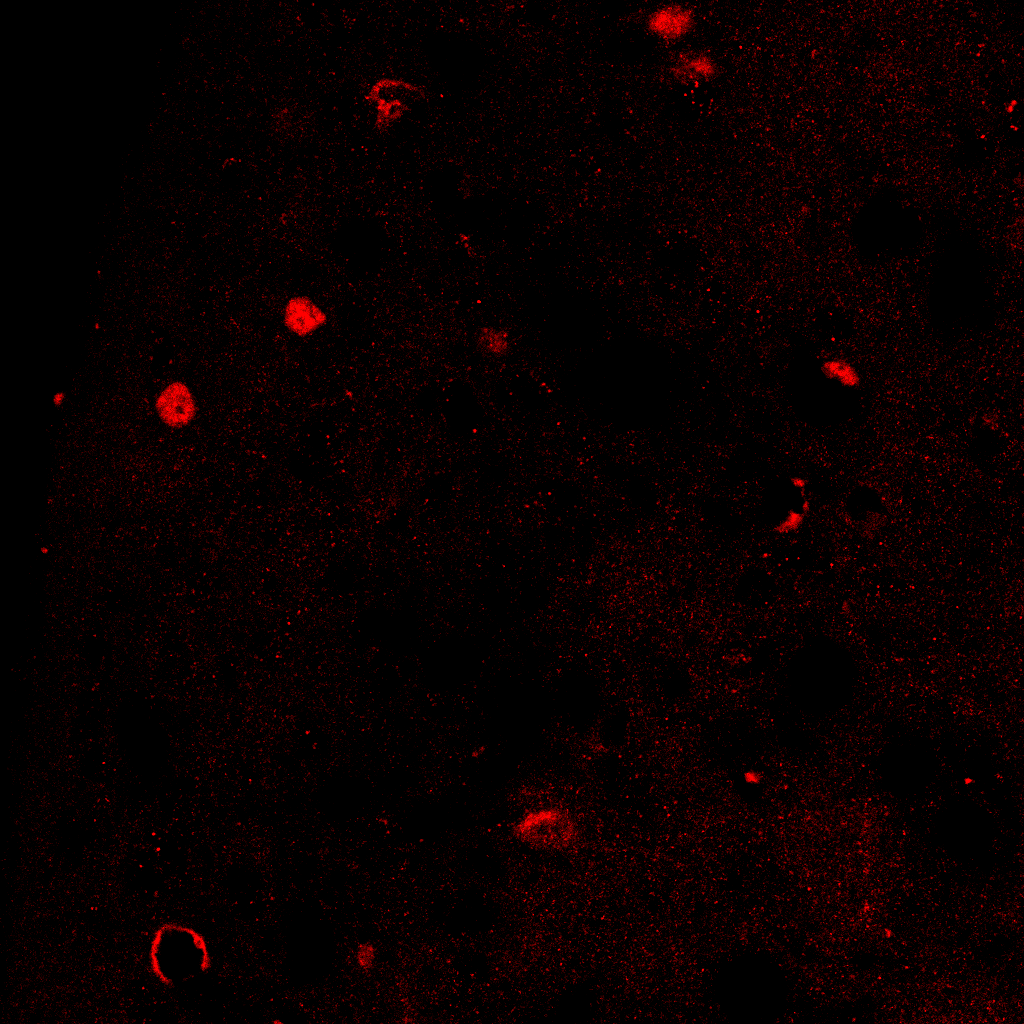

Supplement: Supplementary file 9 — Source data Fig. 7 [file 44319_2025_403_MOESM9_ESM.zip › Figure 7/7F/TMT/LSD/c-Fos.tif]

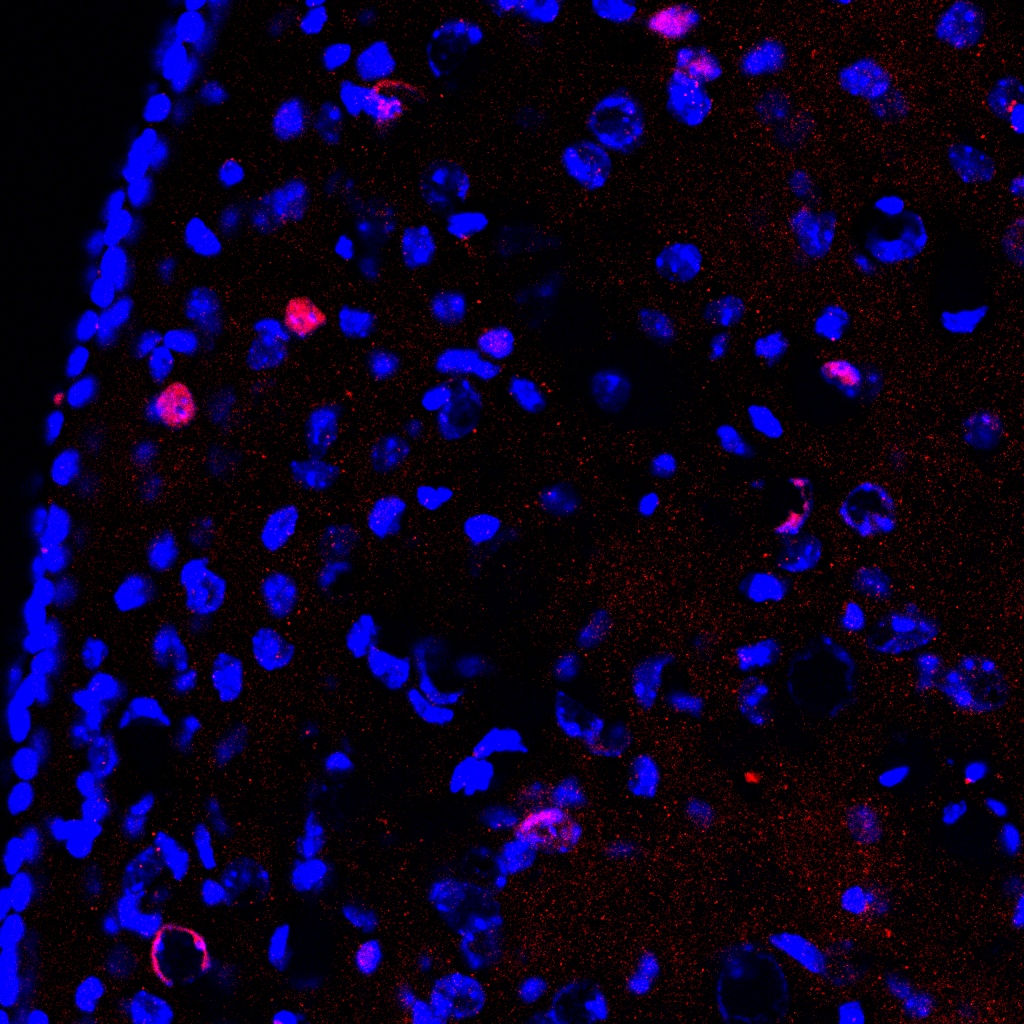

Supplement: Supplementary file 9 — Source data Fig. 7 [file 44319_2025_403_MOESM9_ESM.zip › Figure 7/7F/TMT/LSD/overlay.tif]

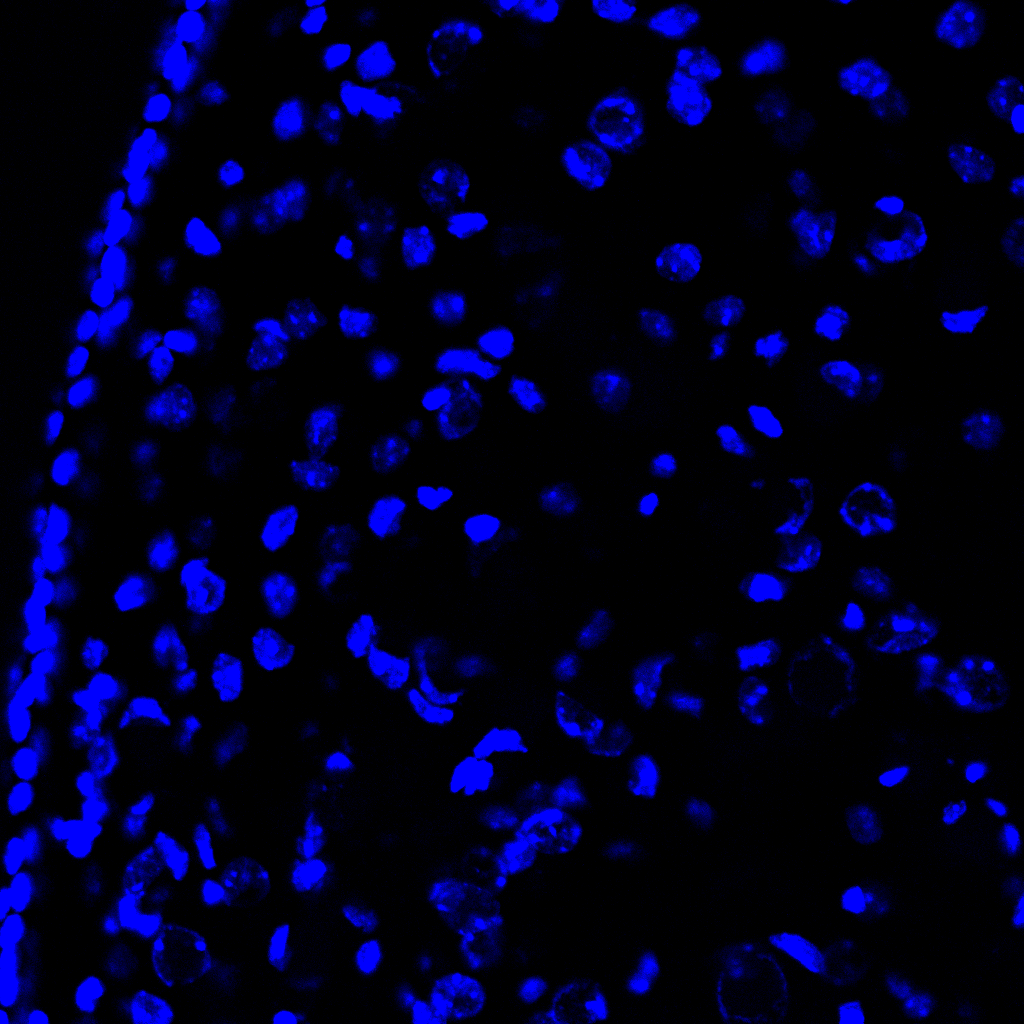

Supplement: Supplementary file 9 — Source data Fig. 7 [file 44319_2025_403_MOESM9_ESM.zip › Figure 7/7F/TMT/LSD/Hoechst.tif]

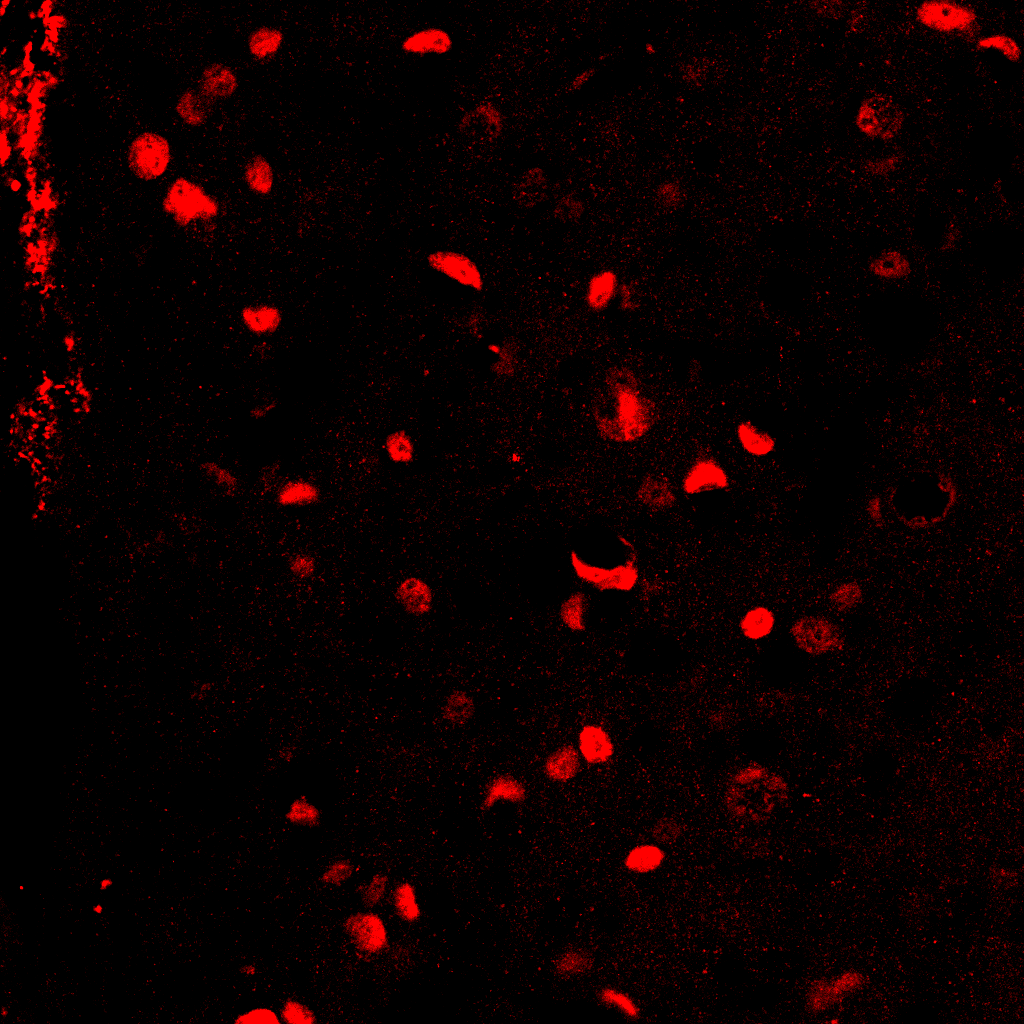

Supplement: Supplementary file 9 — Source data Fig. 7 [file 44319_2025_403_MOESM9_ESM.zip › Figure 7/7F/TMT/LSV/c-Fos.tif]

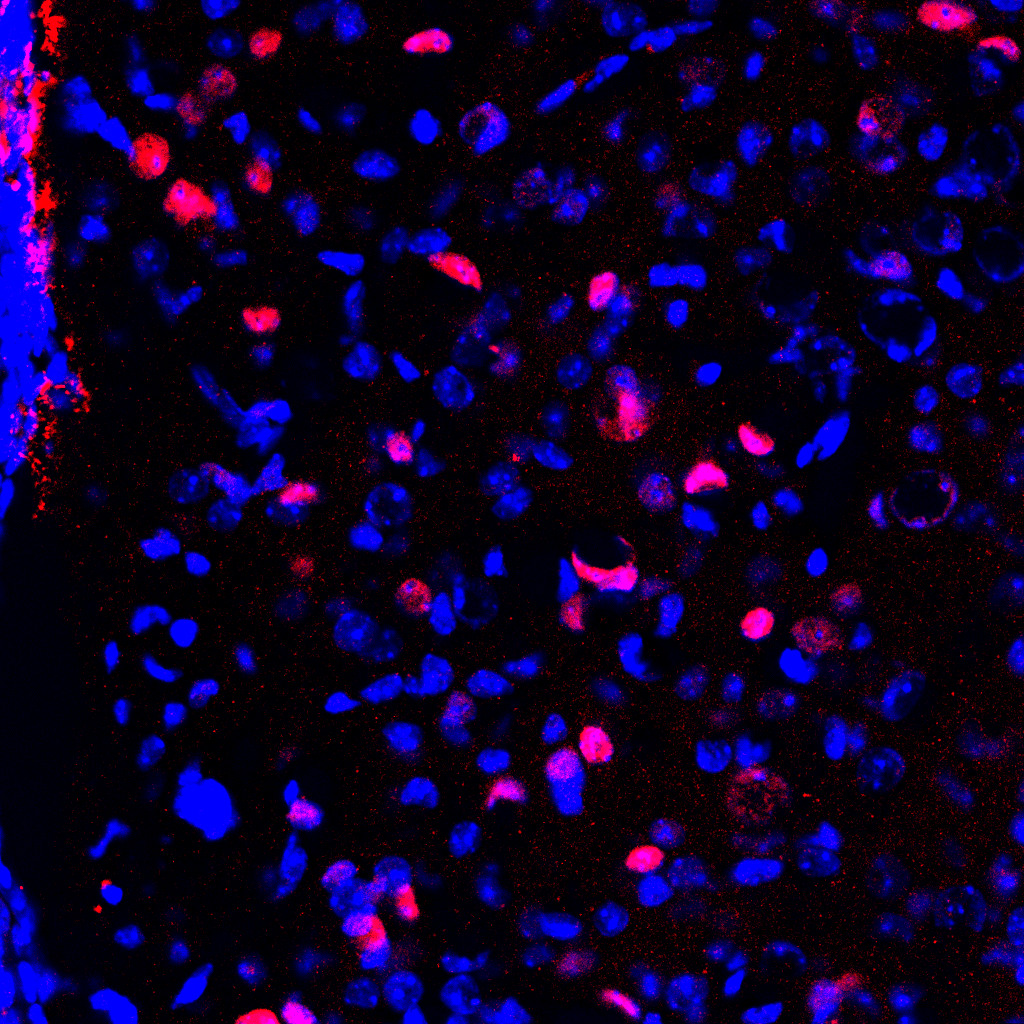

Supplement: Supplementary file 9 — Source data Fig. 7 [file 44319_2025_403_MOESM9_ESM.zip › Figure 7/7F/TMT/LSV/overlay.tif]

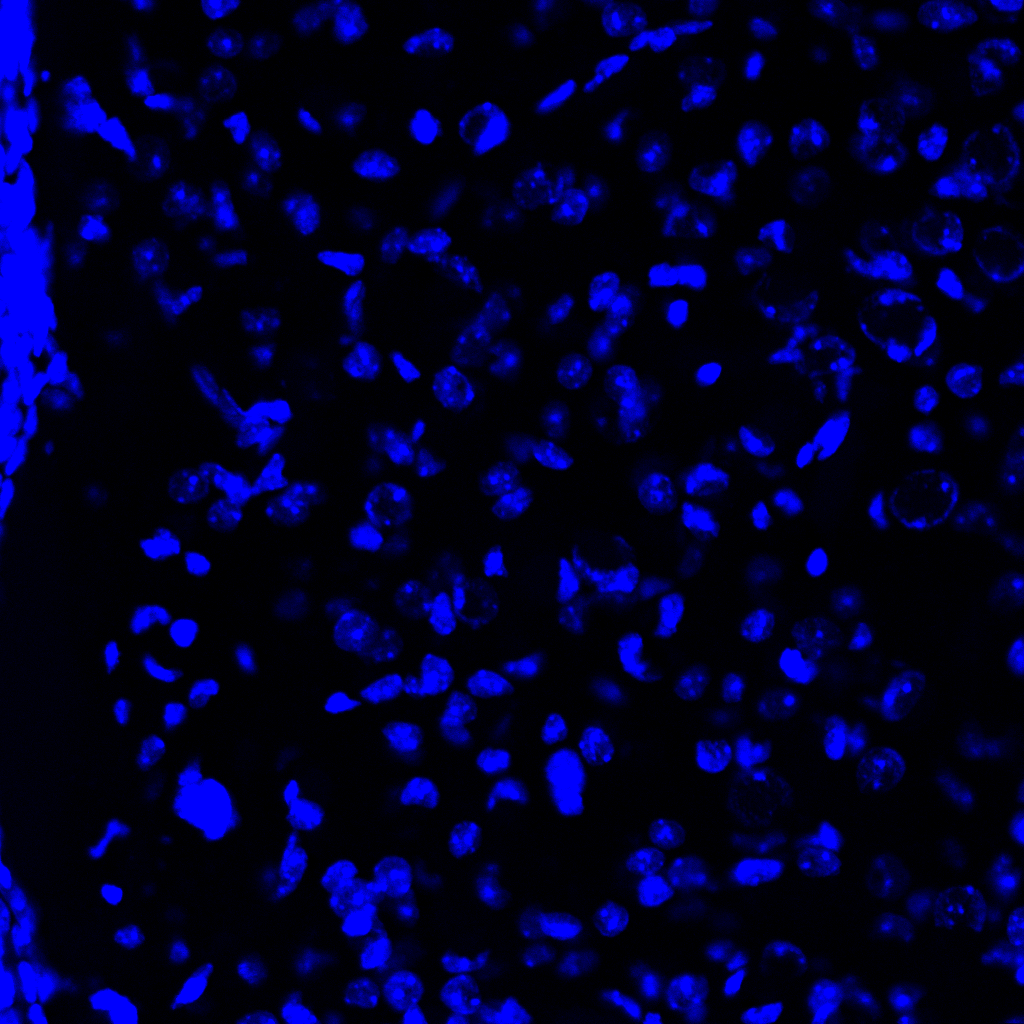

Supplement: Supplementary file 9 — Source data Fig. 7 [file 44319_2025_403_MOESM9_ESM.zip › Figure 7/7F/TMT/LSV/Hoechst.tif]

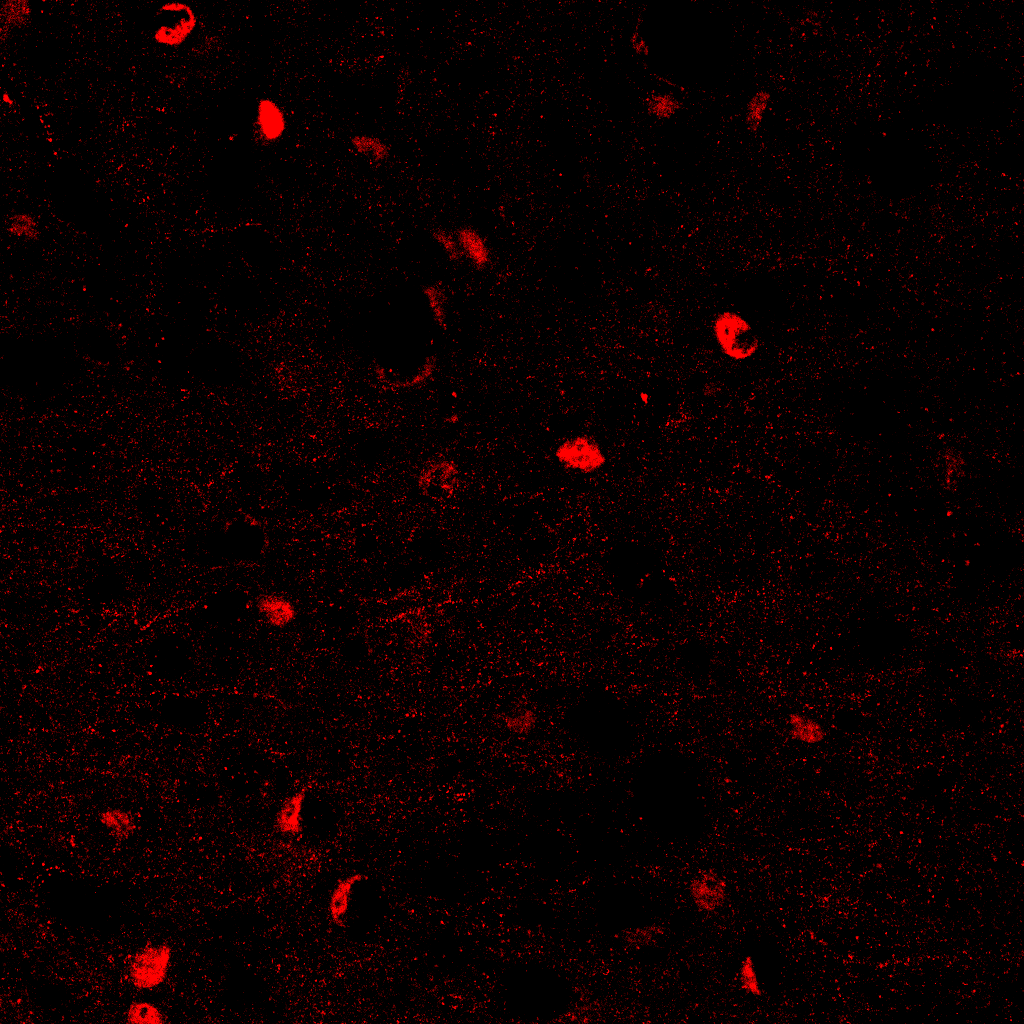

Supplement: Supplementary file 9 — Source data Fig. 7 [file 44319_2025_403_MOESM9_ESM.zip › Figure 7/7F/TMT/LSI/c-Fos.tif]

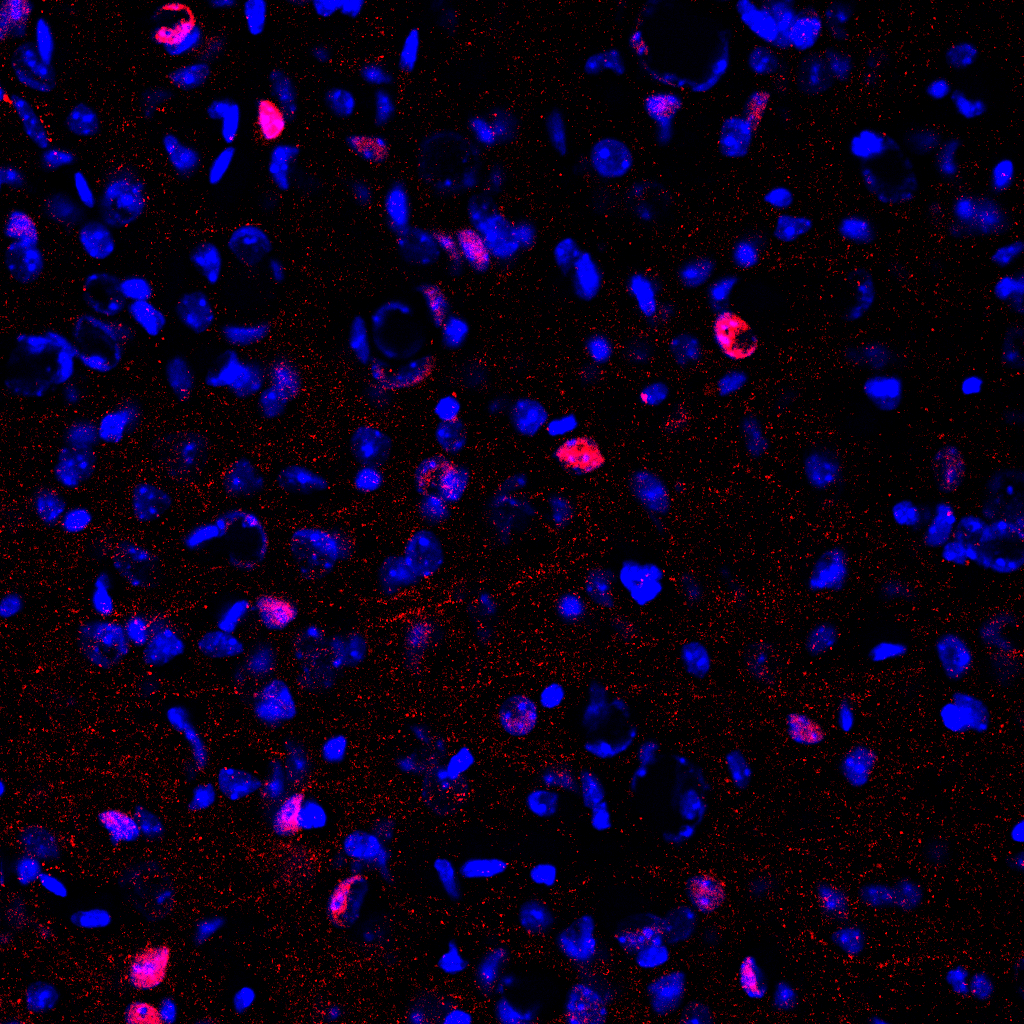

Supplement: Supplementary file 9 — Source data Fig. 7 [file 44319_2025_403_MOESM9_ESM.zip › Figure 7/7F/TMT/LSI/overlay.tif]

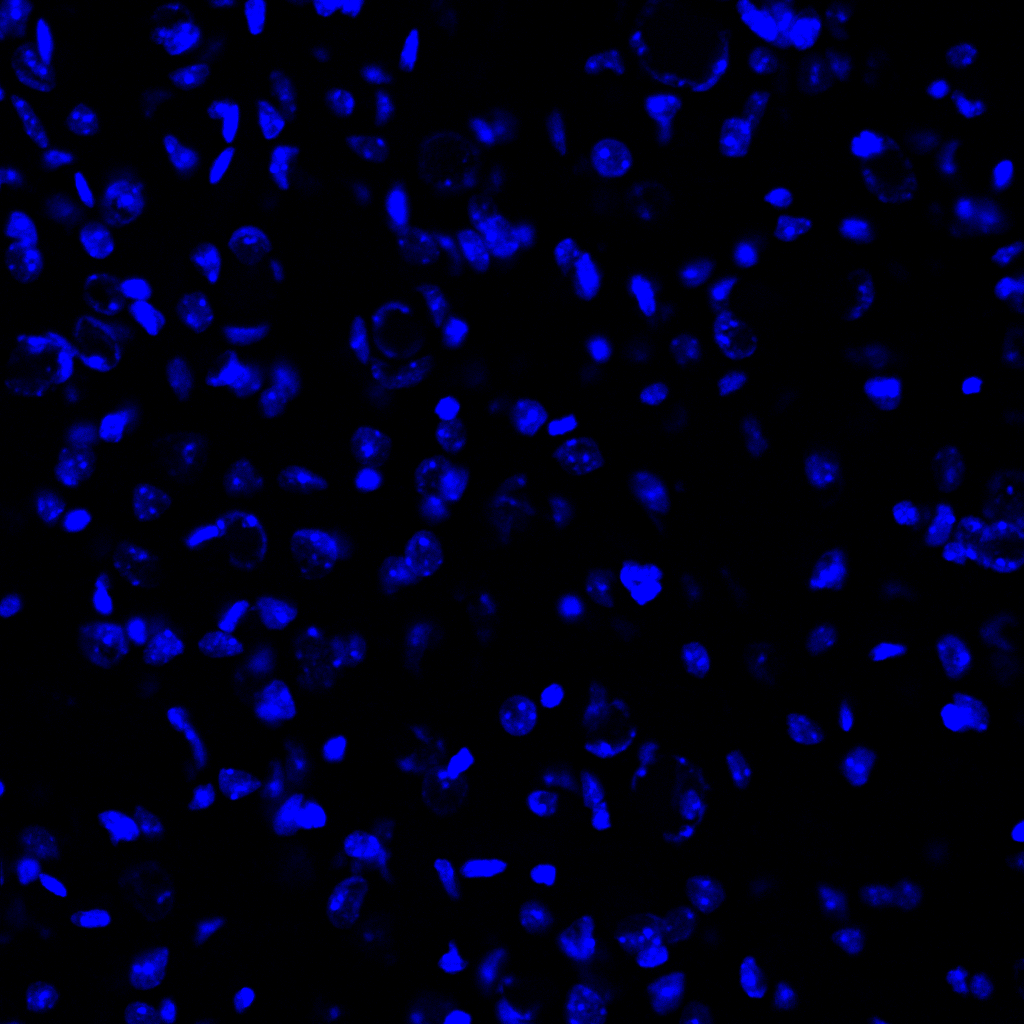

Supplement: Supplementary file 9 — Source data Fig. 7 [file 44319_2025_403_MOESM9_ESM.zip › Figure 7/7F/TMT/LSI/Hoechst.tif]

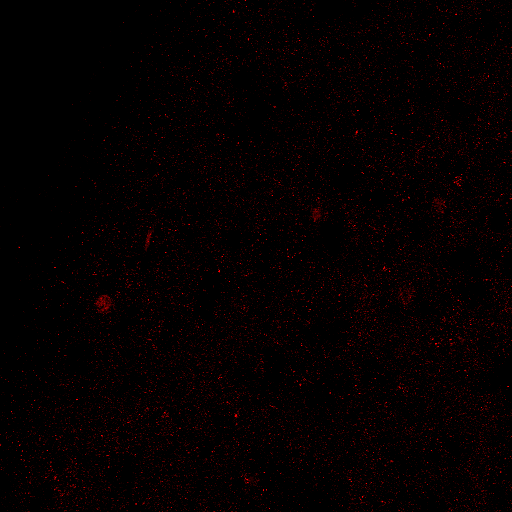

Supplement: Supplementary file 9 — Source data Fig. 7 [file 44319_2025_403_MOESM9_ESM.zip › Figure 7/7F/Control/LSD/c-Fos.tif]

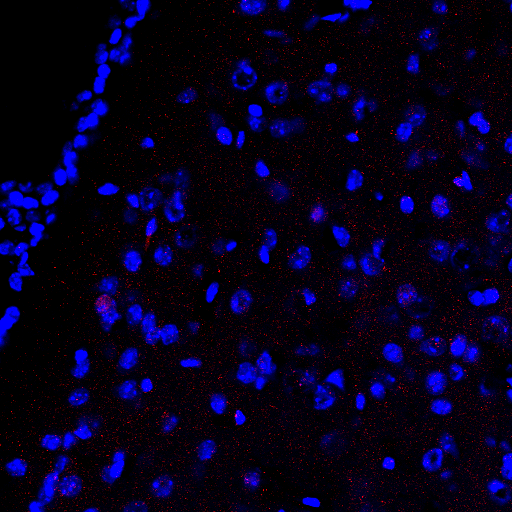

Supplement: Supplementary file 9 — Source data Fig. 7 [file 44319_2025_403_MOESM9_ESM.zip › Figure 7/7F/Control/LSD/overlay.tif]

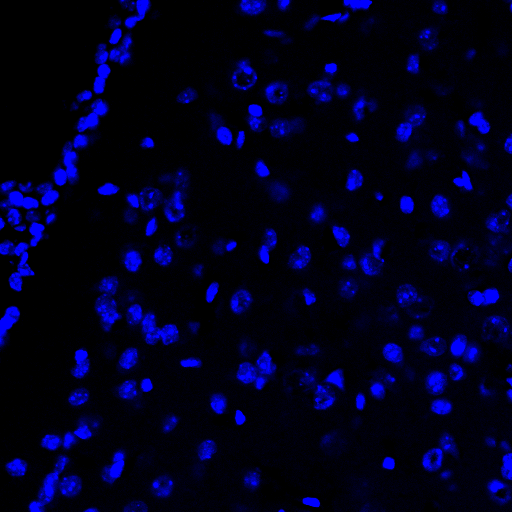

Supplement: Supplementary file 9 — Source data Fig. 7 [file 44319_2025_403_MOESM9_ESM.zip › Figure 7/7F/Control/LSD/Hoechst.tif]

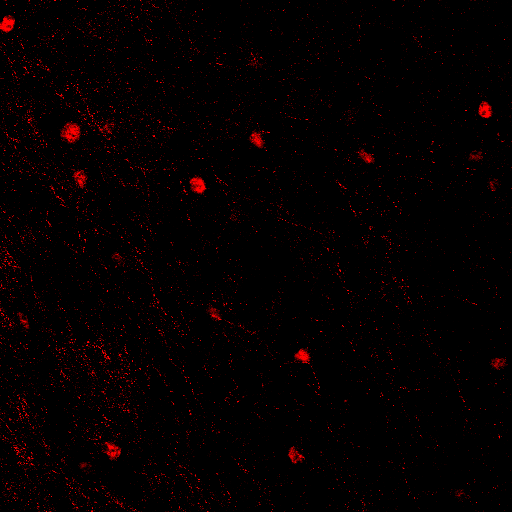

Supplement: Supplementary file 9 — Source data Fig. 7 [file 44319_2025_403_MOESM9_ESM.zip › Figure 7/7F/Control/LSV/c-Fos.tif]

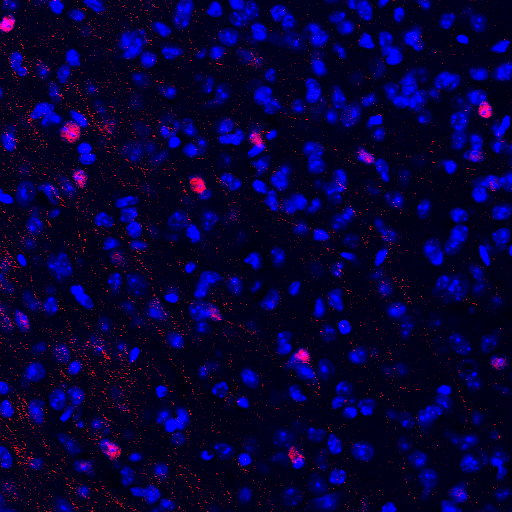

Supplement: Supplementary file 9 — Source data Fig. 7 [file 44319_2025_403_MOESM9_ESM.zip › Figure 7/7F/Control/LSV/overlay.tif]

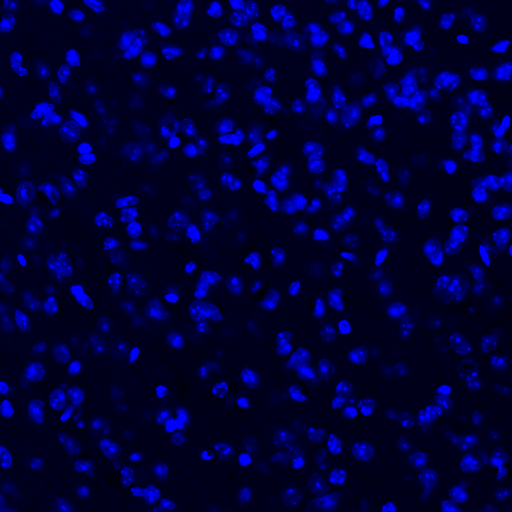

Supplement: Supplementary file 9 — Source data Fig. 7 [file 44319_2025_403_MOESM9_ESM.zip › Figure 7/7F/Control/LSV/Hoechst.tif]

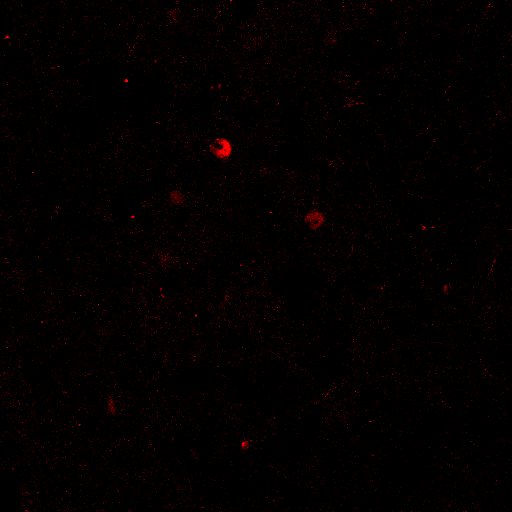

Supplement: Supplementary file 9 — Source data Fig. 7 [file 44319_2025_403_MOESM9_ESM.zip › Figure 7/7F/Control/LSI/c-Fos.tif]

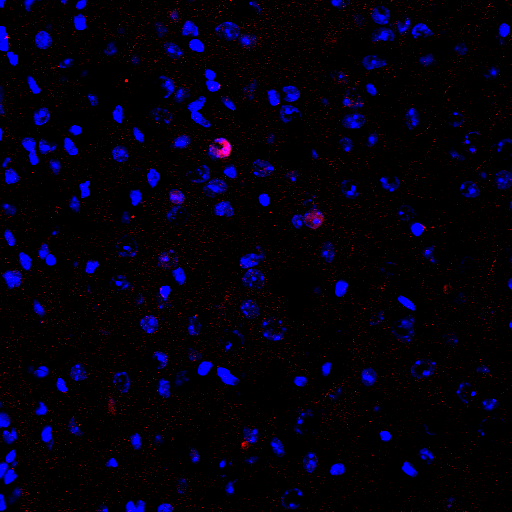

Supplement: Supplementary file 9 — Source data Fig. 7 [file 44319_2025_403_MOESM9_ESM.zip › Figure 7/7F/Control/LSI/overlay.tif]

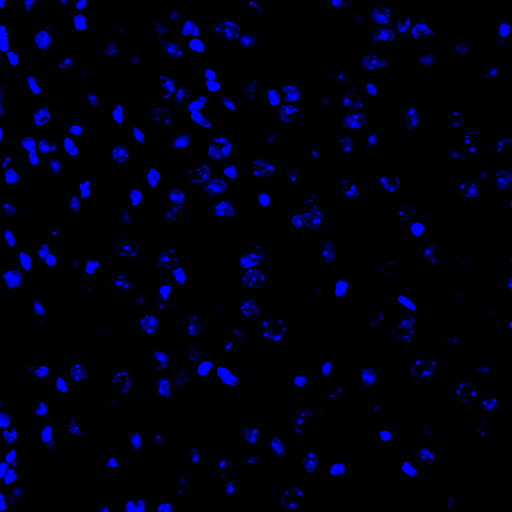

Supplement: Supplementary file 9 — Source data Fig. 7 [file 44319_2025_403_MOESM9_ESM.zip › Figure 7/7F/Control/LSI/Hoechst.tif]

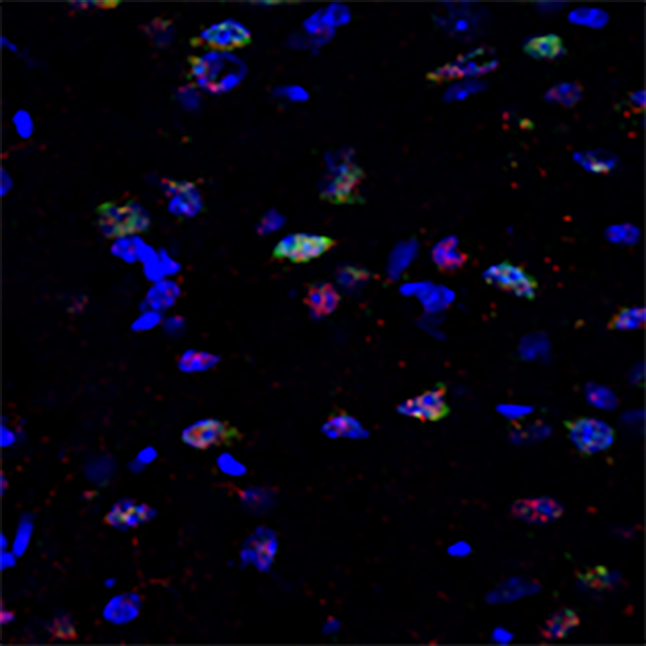

Supplement: Supplementary file 10 — Source data Fig. 8 [file 44319_2025_403_MOESM10_ESM.zip › Figure 8/8D/TCS/overlay 2.tif]

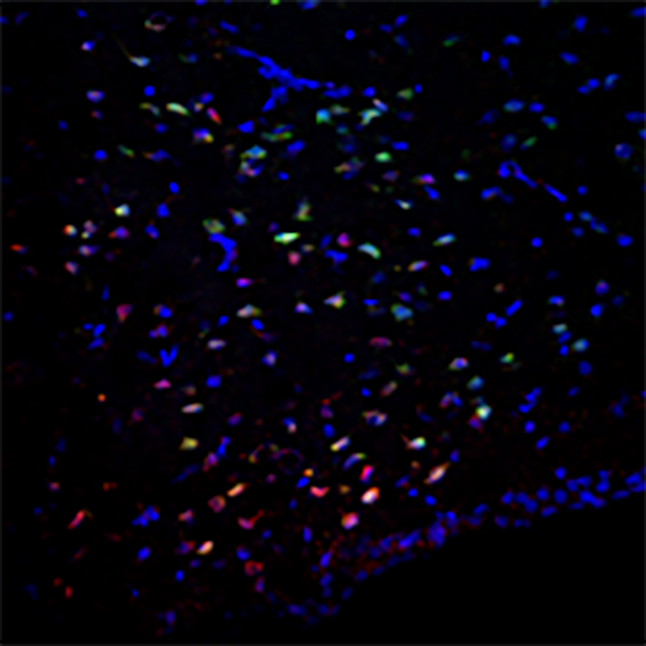

Supplement: Supplementary file 10 — Source data Fig. 8 [file 44319_2025_403_MOESM10_ESM.zip › Figure 8/8D/TCS/overlay 1.tif]

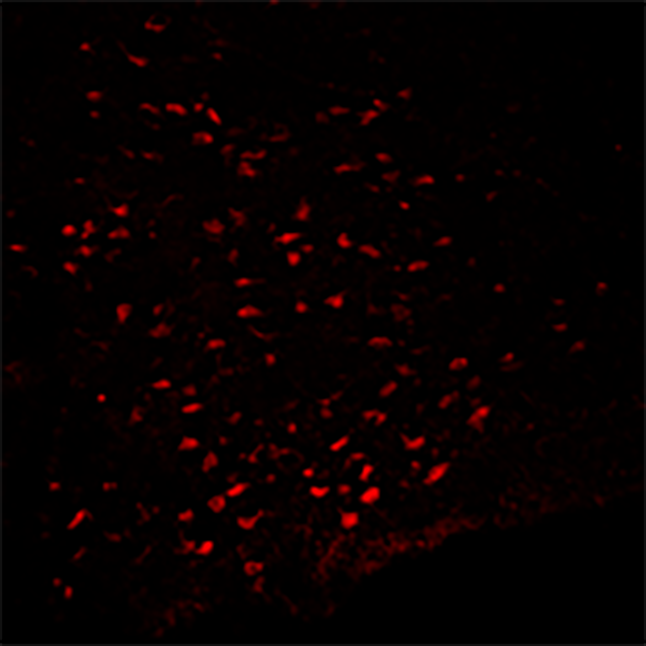

Supplement: Supplementary file 10 — Source data Fig. 8 [file 44319_2025_403_MOESM10_ESM.zip › Figure 8/8D/TCS/c-Fos.tif]

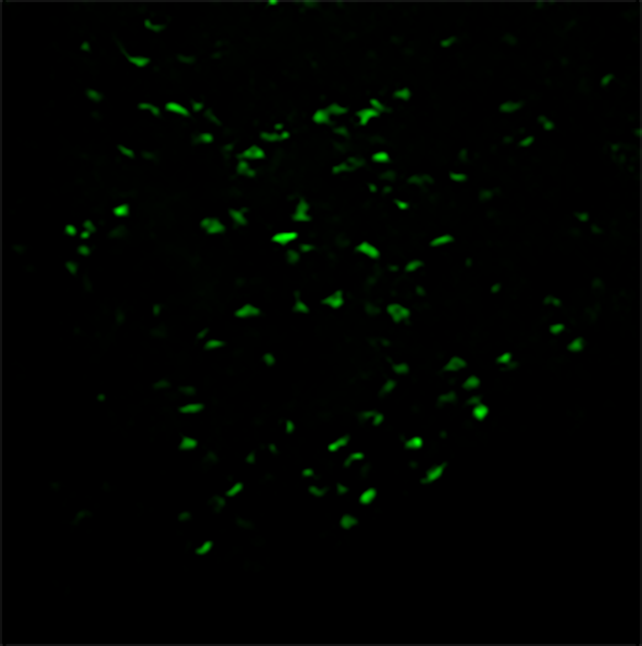

Supplement: Supplementary file 10 — Source data Fig. 8 [file 44319_2025_403_MOESM10_ESM.zip › Figure 8/8D/TCS/NeuN.tif]

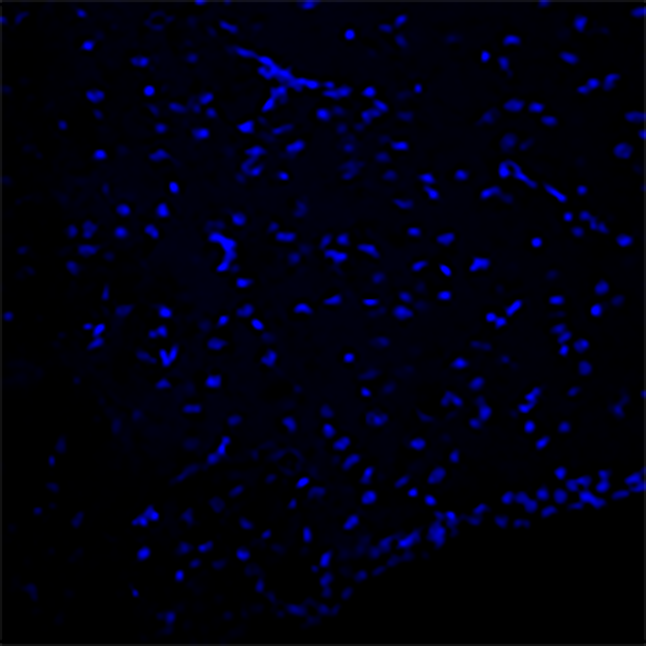

Supplement: Supplementary file 10 — Source data Fig. 8 [file 44319_2025_403_MOESM10_ESM.zip › Figure 8/8D/TCS/Hoechst.tif]

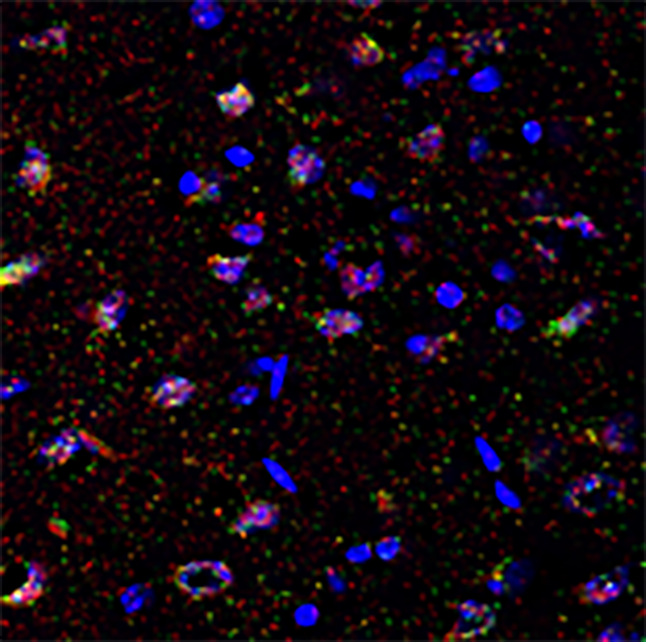

Supplement: Supplementary file 10 — Source data Fig. 8 [file 44319_2025_403_MOESM10_ESM.zip › Figure 8/8D/Heat inactivated antibody/overlay 2.tif]

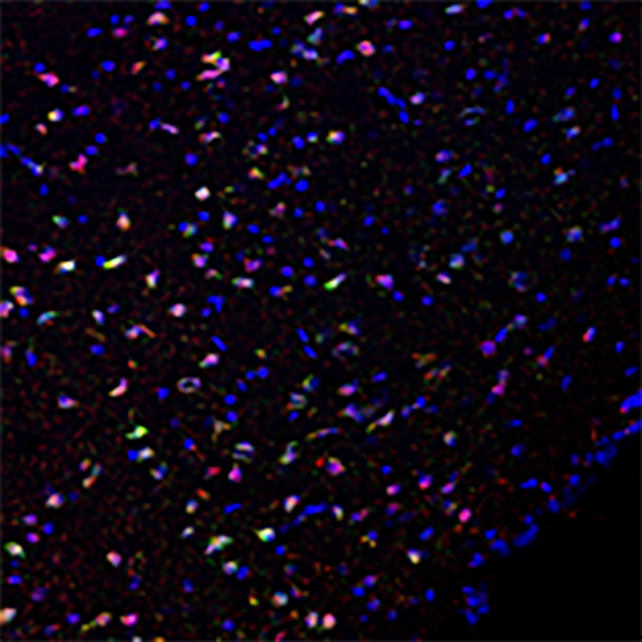

Supplement: Supplementary file 10 — Source data Fig. 8 [file 44319_2025_403_MOESM10_ESM.zip › Figure 8/8D/Heat inactivated antibody/overlay 1.tif]

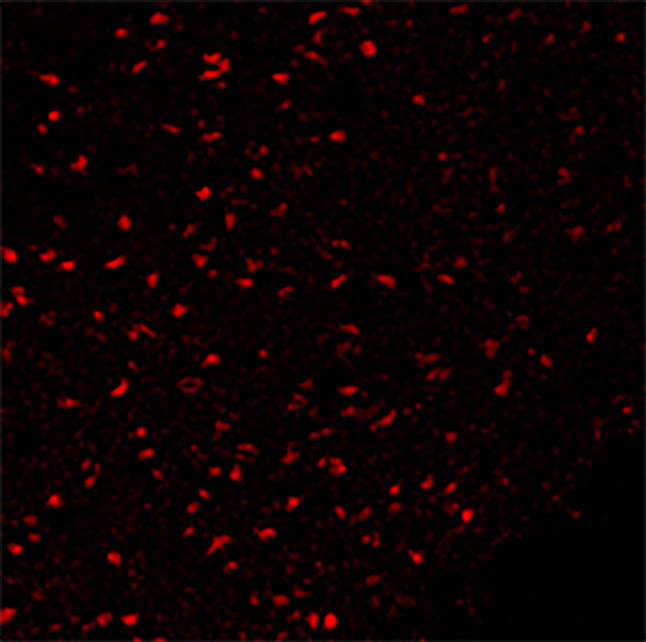

Supplement: Supplementary file 10 — Source data Fig. 8 [file 44319_2025_403_MOESM10_ESM.zip › Figure 8/8D/Heat inactivated antibody/c-Fos.tif]

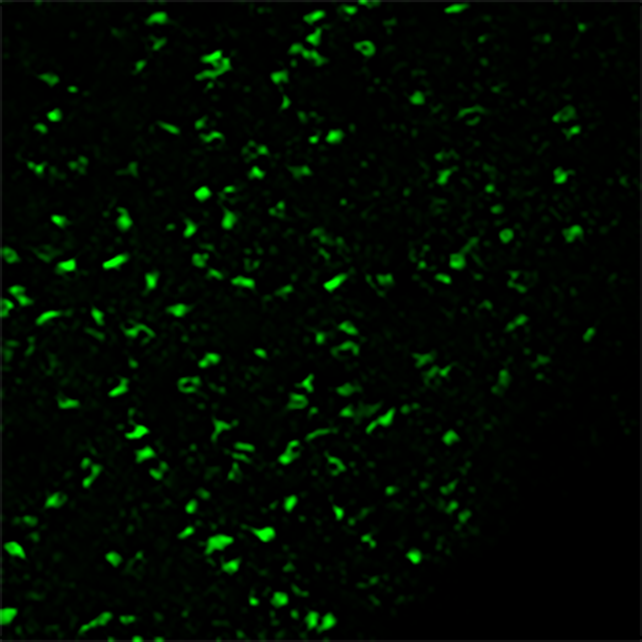

Supplement: Supplementary file 10 — Source data Fig. 8 [file 44319_2025_403_MOESM10_ESM.zip › Figure 8/8D/Heat inactivated antibody/NeuN.tif]

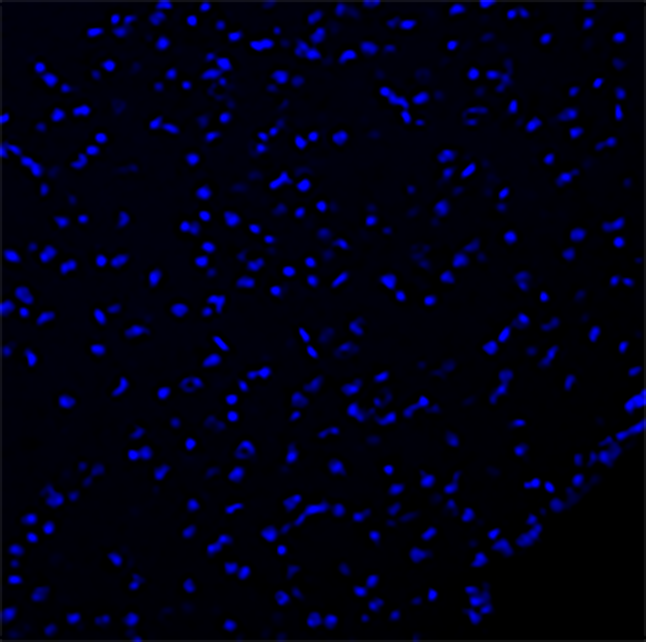

Supplement: Supplementary file 10 — Source data Fig. 8 [file 44319_2025_403_MOESM10_ESM.zip › Figure 8/8D/Heat inactivated antibody/Hoechst.tif]

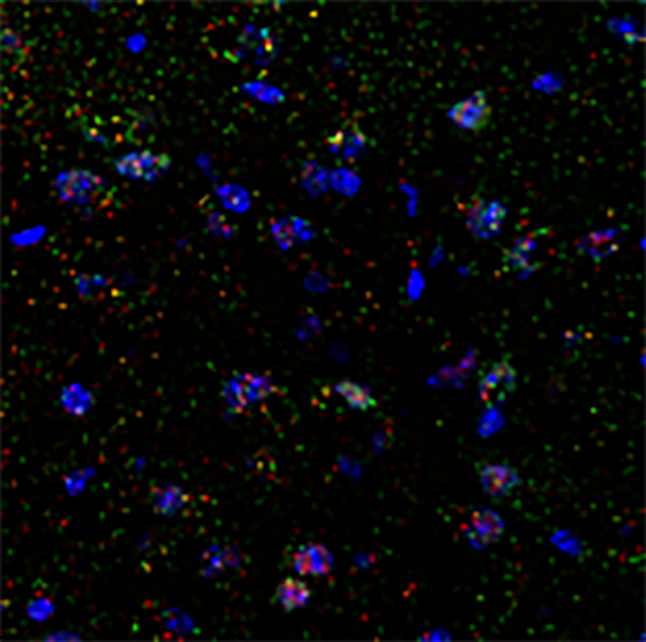

Supplement: Supplementary file 10 — Source data Fig. 8 [file 44319_2025_403_MOESM10_ESM.zip › Figure 8/8D/Vehicle/overlay 2.tif]

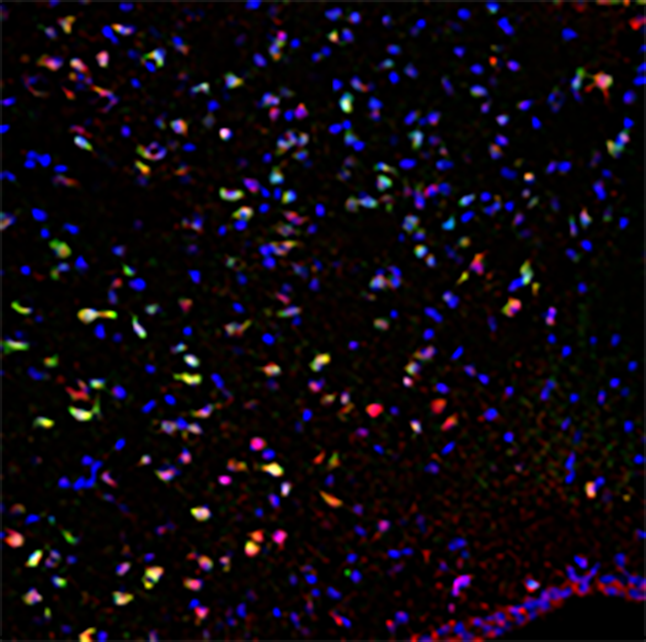

Supplement: Supplementary file 10 — Source data Fig. 8 [file 44319_2025_403_MOESM10_ESM.zip › Figure 8/8D/Vehicle/overlay 1.tif]

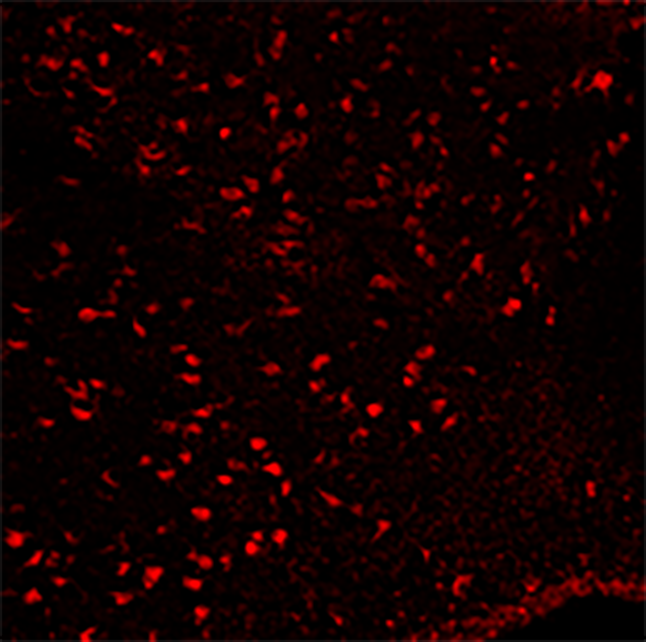

Supplement: Supplementary file 10 — Source data Fig. 8 [file 44319_2025_403_MOESM10_ESM.zip › Figure 8/8D/Vehicle/c-Fos.tif]

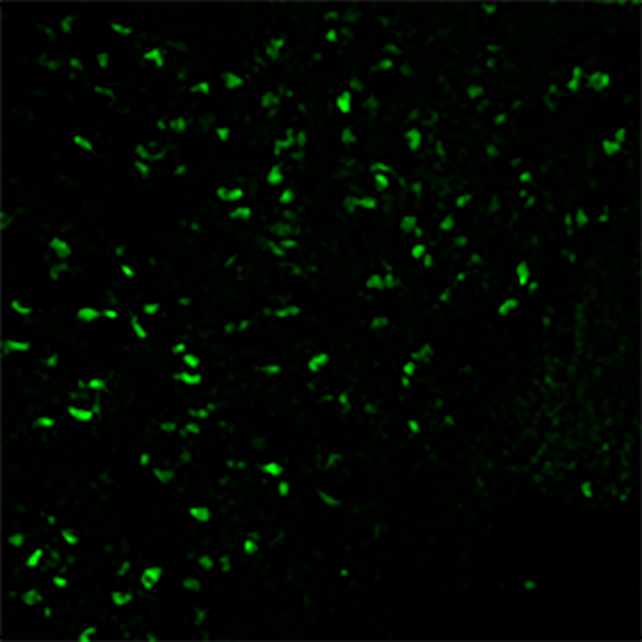

Supplement: Supplementary file 10 — Source data Fig. 8 [file 44319_2025_403_MOESM10_ESM.zip › Figure 8/8D/Vehicle/NeuN.tif]

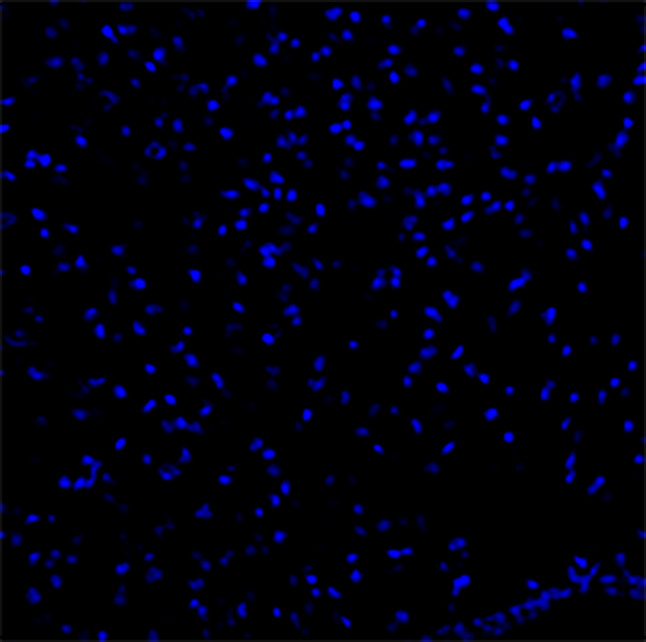

Supplement: Supplementary file 10 — Source data Fig. 8 [file 44319_2025_403_MOESM10_ESM.zip › Figure 8/8D/Vehicle/Hoechst.tif]

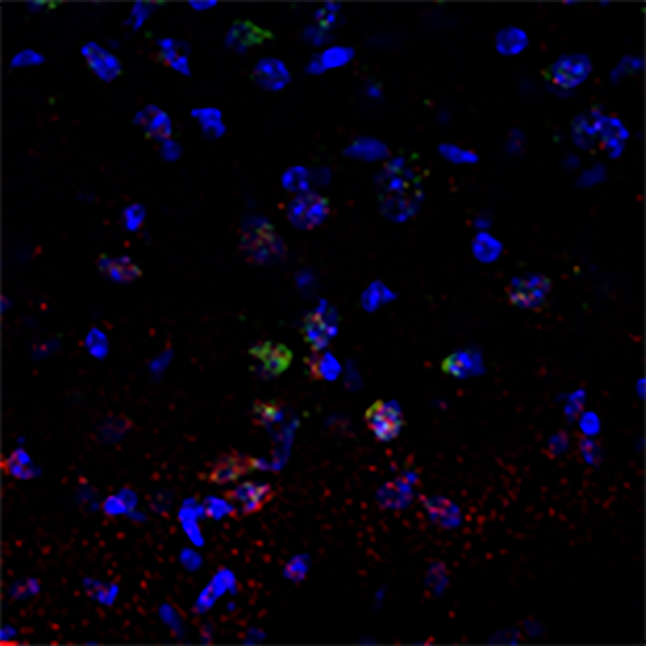

Supplement: Supplementary file 10 — Source data Fig. 8 [file 44319_2025_403_MOESM10_ESM.zip › Figure 8/8D/ORB antibody/overlay 2.tif]

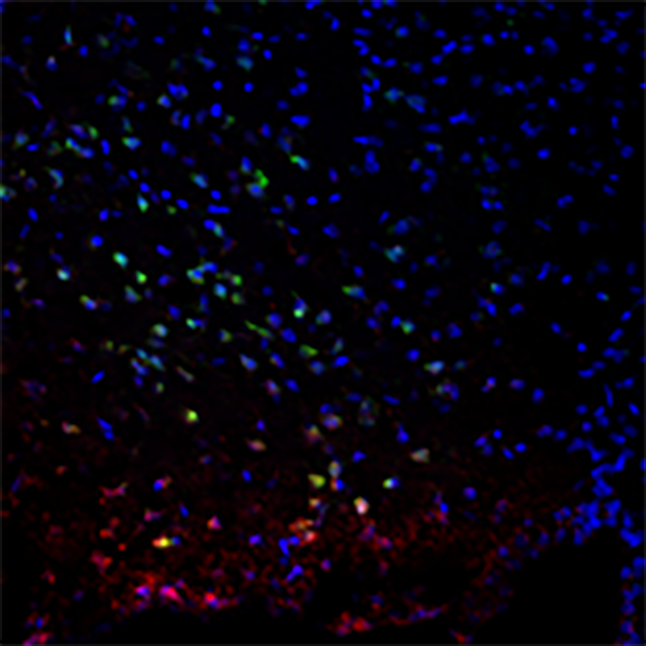

Supplement: Supplementary file 10 — Source data Fig. 8 [file 44319_2025_403_MOESM10_ESM.zip › Figure 8/8D/ORB antibody/overlay 1.tif]

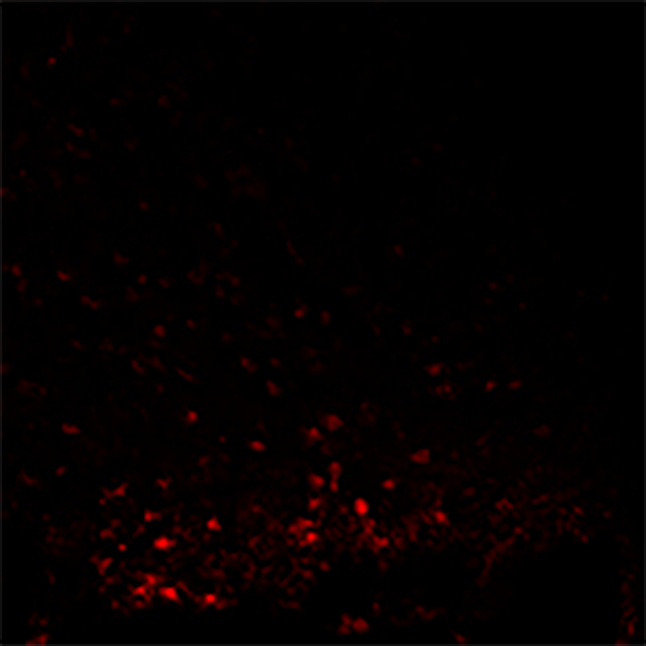

Supplement: Supplementary file 10 — Source data Fig. 8 [file 44319_2025_403_MOESM10_ESM.zip › Figure 8/8D/ORB antibody/c-Fos.tif]

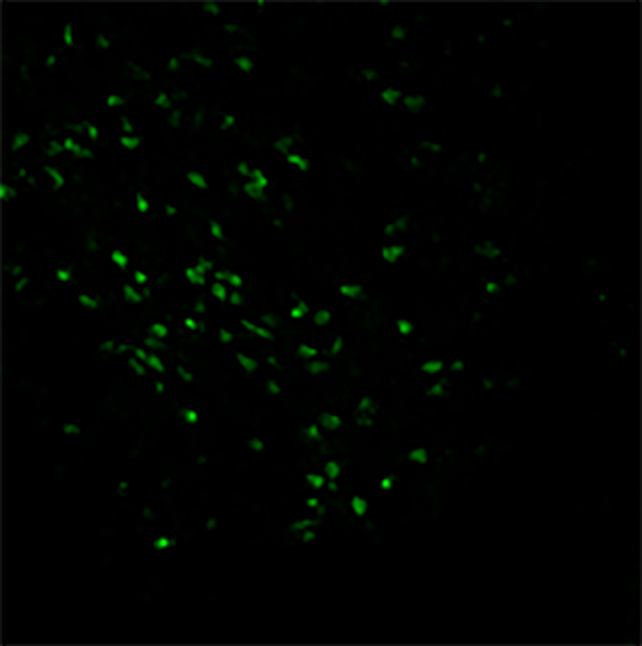

Supplement: Supplementary file 10 — Source data Fig. 8 [file 44319_2025_403_MOESM10_ESM.zip › Figure 8/8D/ORB antibody/NeuN.tif]

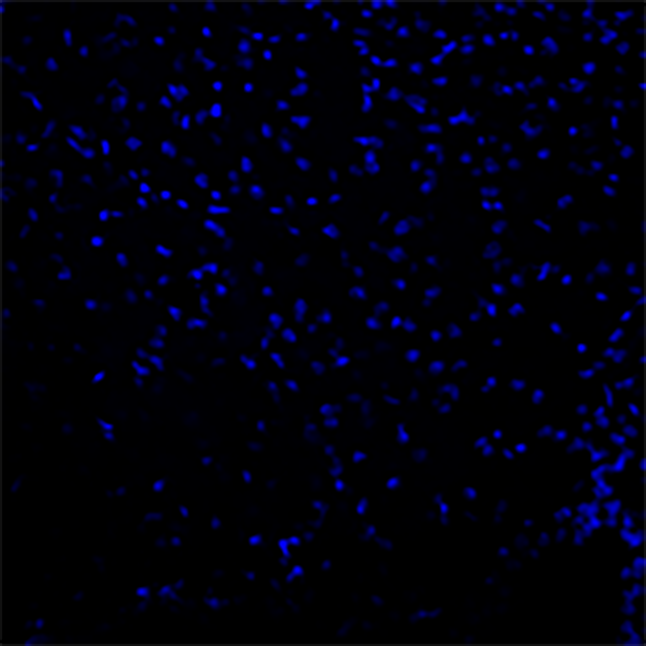

Supplement: Supplementary file 10 — Source data Fig. 8 [file 44319_2025_403_MOESM10_ESM.zip › Figure 8/8D/ORB antibody/Hoechst.tif]

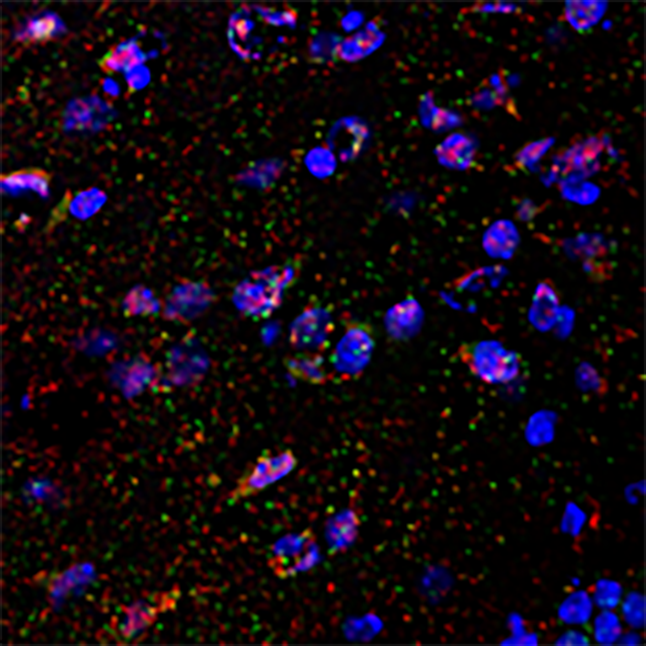

Supplement: Supplementary file 10 — Source data Fig. 8 [file 44319_2025_403_MOESM10_ESM.zip › Figure 8/8D/Control/overlay 2.tif]

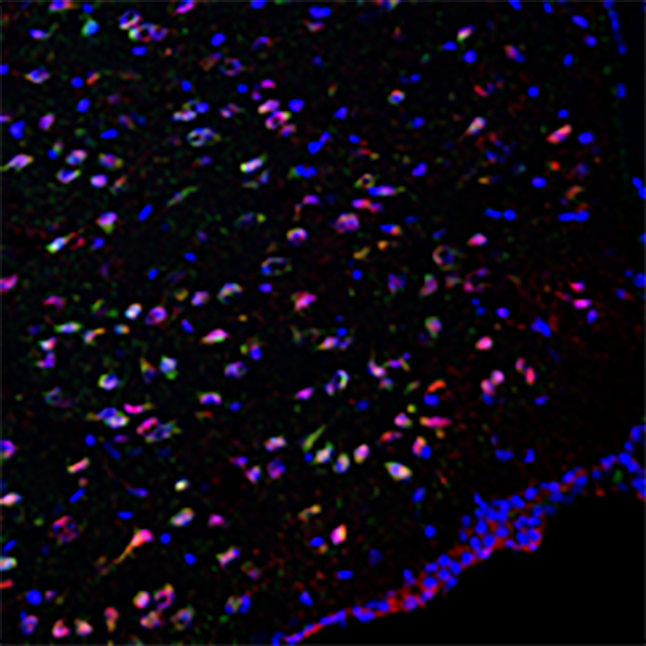

Supplement: Supplementary file 10 — Source data Fig. 8 [file 44319_2025_403_MOESM10_ESM.zip › Figure 8/8D/Control/overlay 1.tif]

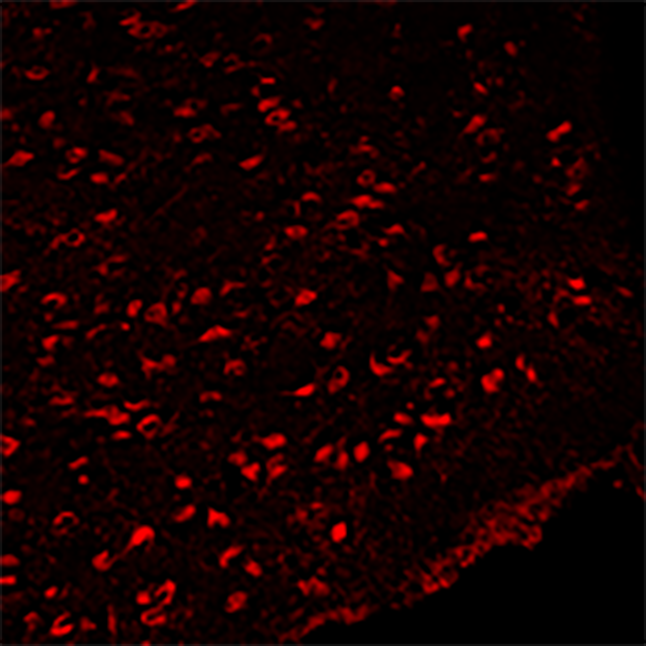

Supplement: Supplementary file 10 — Source data Fig. 8 [file 44319_2025_403_MOESM10_ESM.zip › Figure 8/8D/Control/c-Fos.tif]

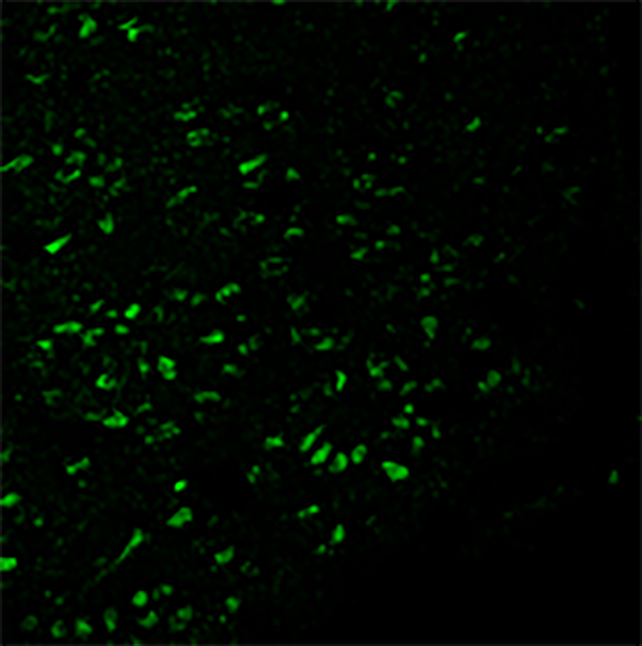

Supplement: Supplementary file 10 — Source data Fig. 8 [file 44319_2025_403_MOESM10_ESM.zip › Figure 8/8D/Control/NeuN.tif]

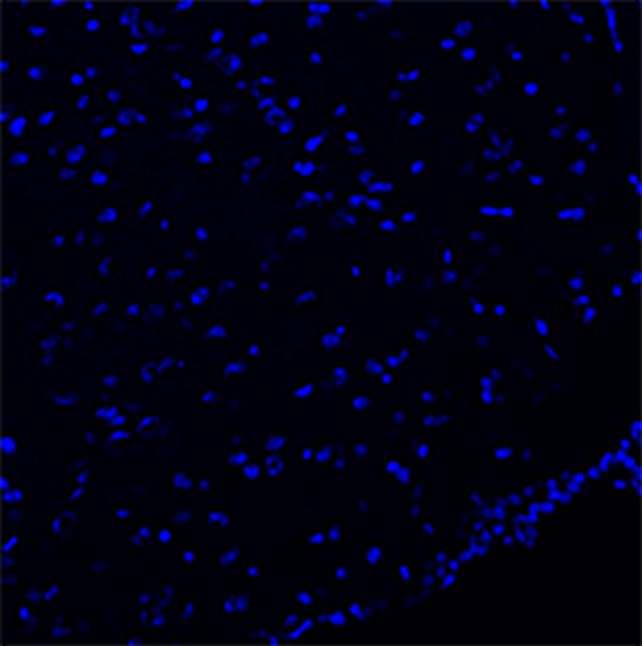

Supplement: Supplementary file 10 — Source data Fig. 8 [file 44319_2025_403_MOESM10_ESM.zip › Figure 8/8D/Control/Hoechst.tif]

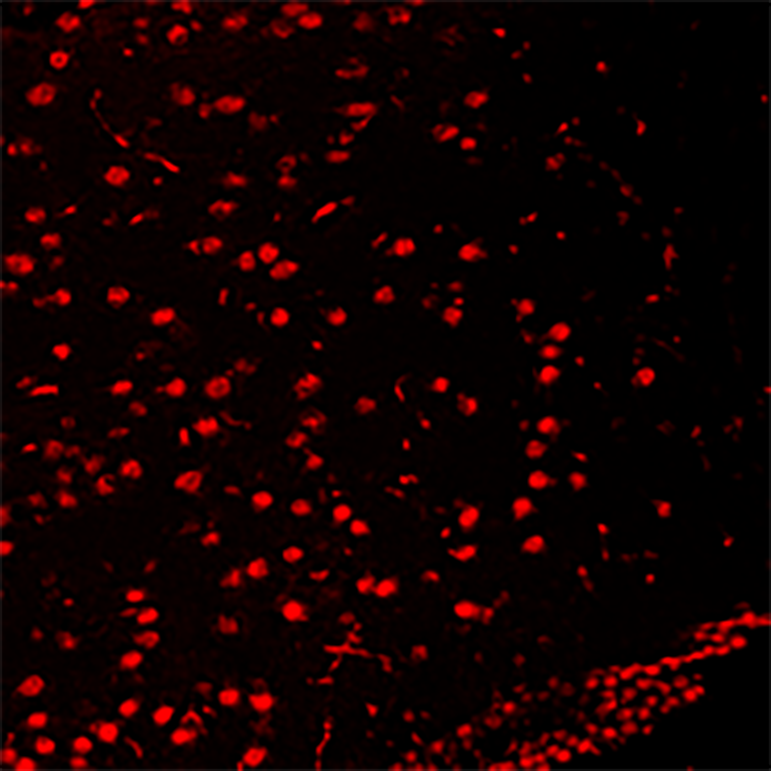

Supplement: Supplementary file 10 — Source data Fig. 8 [file 44319_2025_403_MOESM10_ESM.zip › Figure 8/8A/Orexin B/Tone/Orexin B.tif]

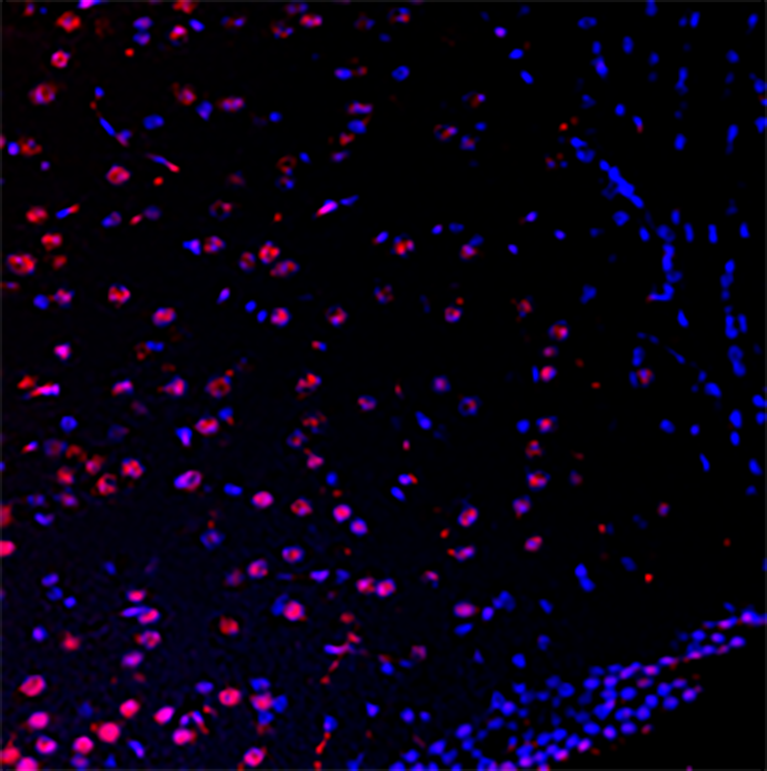

Supplement: Supplementary file 10 — Source data Fig. 8 [file 44319_2025_403_MOESM10_ESM.zip › Figure 8/8A/Orexin B/Tone/overlay 1.tif]

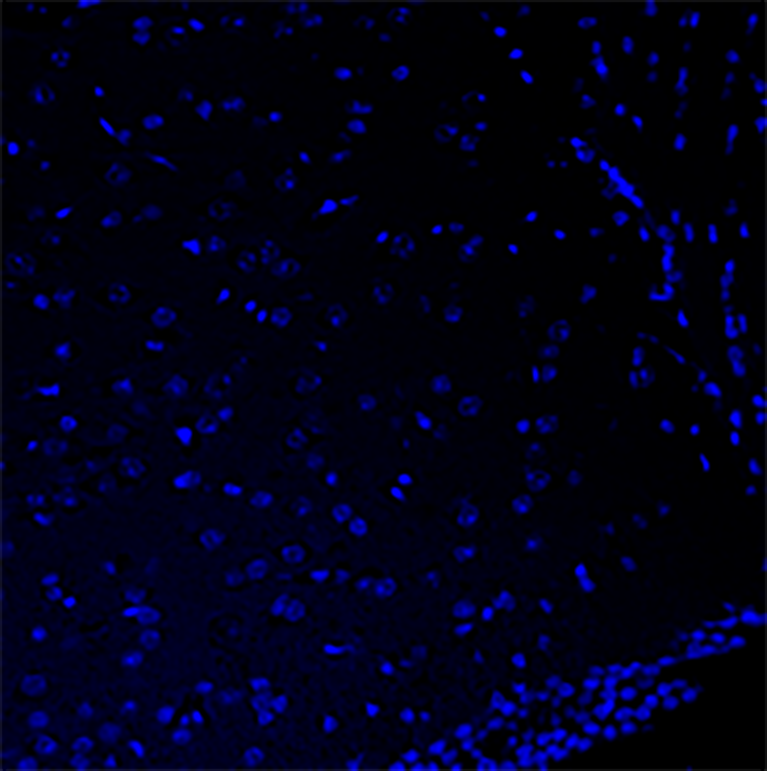

Supplement: Supplementary file 10 — Source data Fig. 8 [file 44319_2025_403_MOESM10_ESM.zip › Figure 8/8A/Orexin B/Tone/Hoechst.tif]

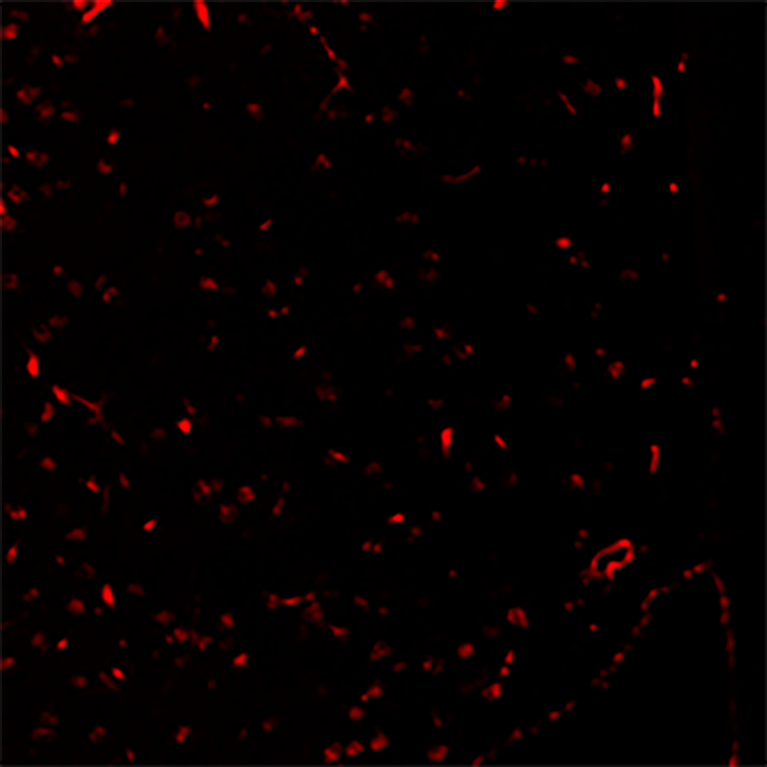

Supplement: Supplementary file 10 — Source data Fig. 8 [file 44319_2025_403_MOESM10_ESM.zip › Figure 8/8A/Orexin B/Control/Orexin B.tif]

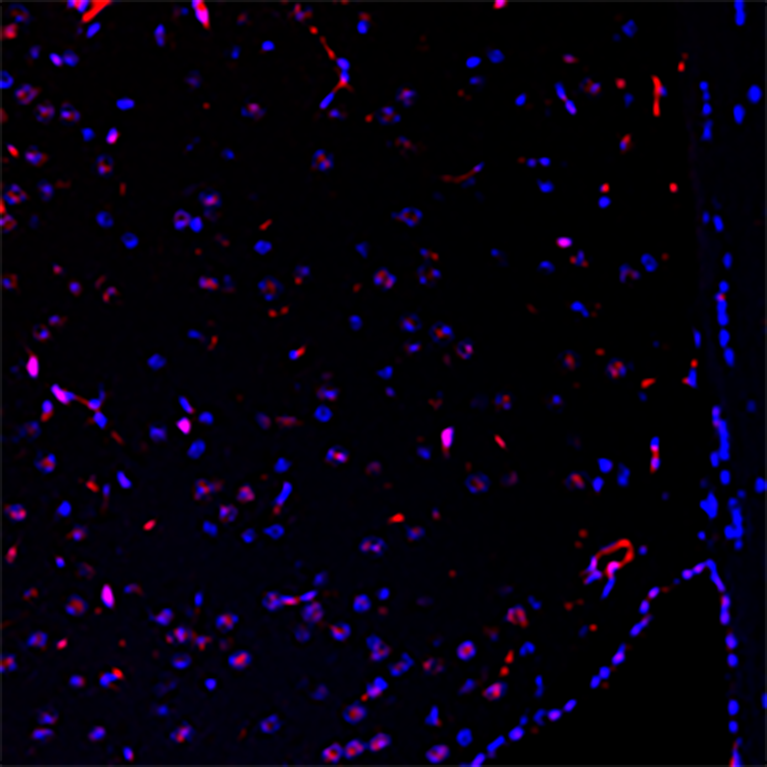

Supplement: Supplementary file 10 — Source data Fig. 8 [file 44319_2025_403_MOESM10_ESM.zip › Figure 8/8A/Orexin B/Control/overlay 1.tif]

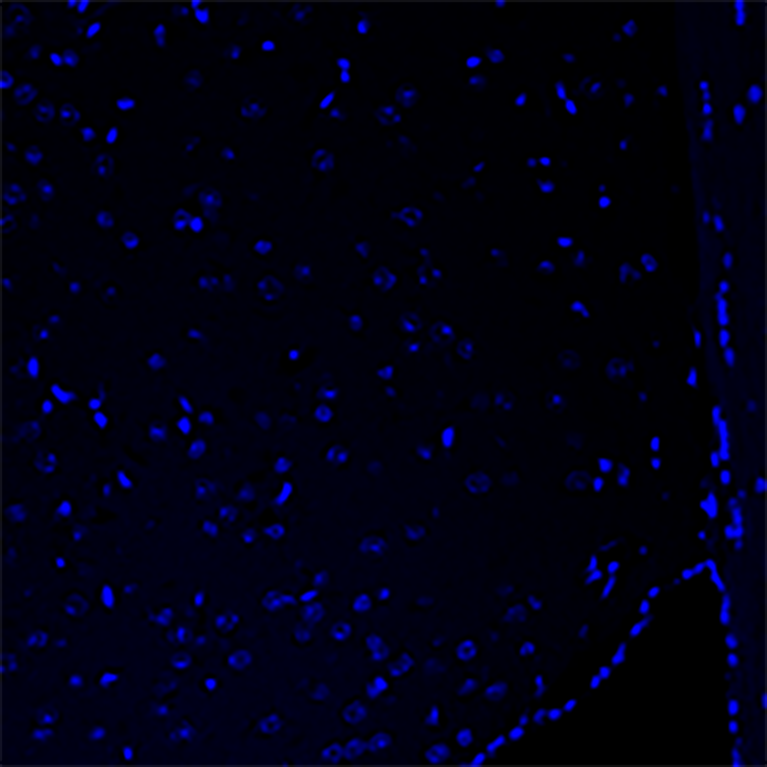

Supplement: Supplementary file 10 — Source data Fig. 8 [file 44319_2025_403_MOESM10_ESM.zip › Figure 8/8A/Orexin B/Control/Hoechst.tif]

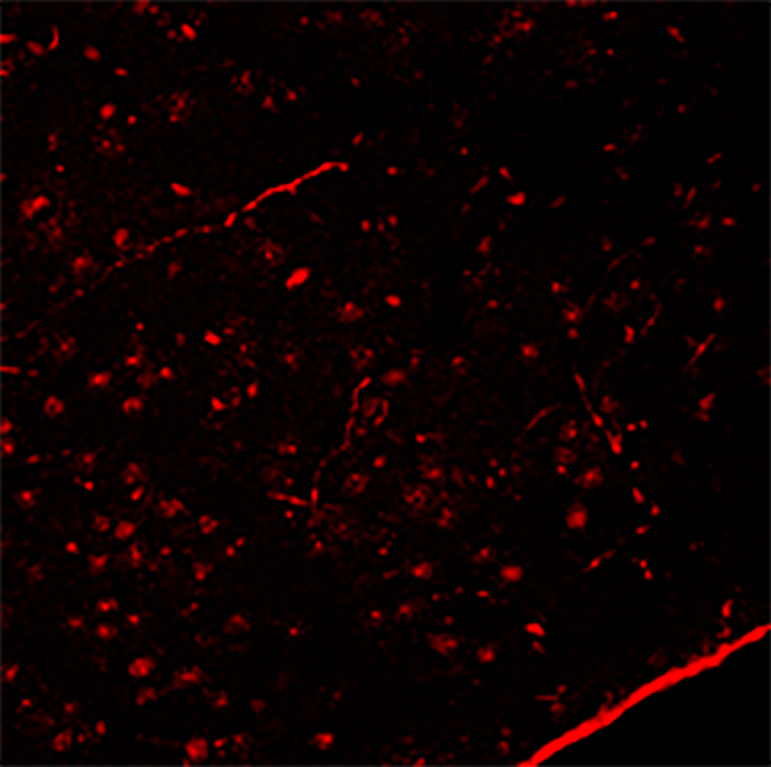

Supplement: Supplementary file 10 — Source data Fig. 8 [file 44319_2025_403_MOESM10_ESM.zip › Figure 8/8A/Orexin A/Tone/Orexin A.tif]

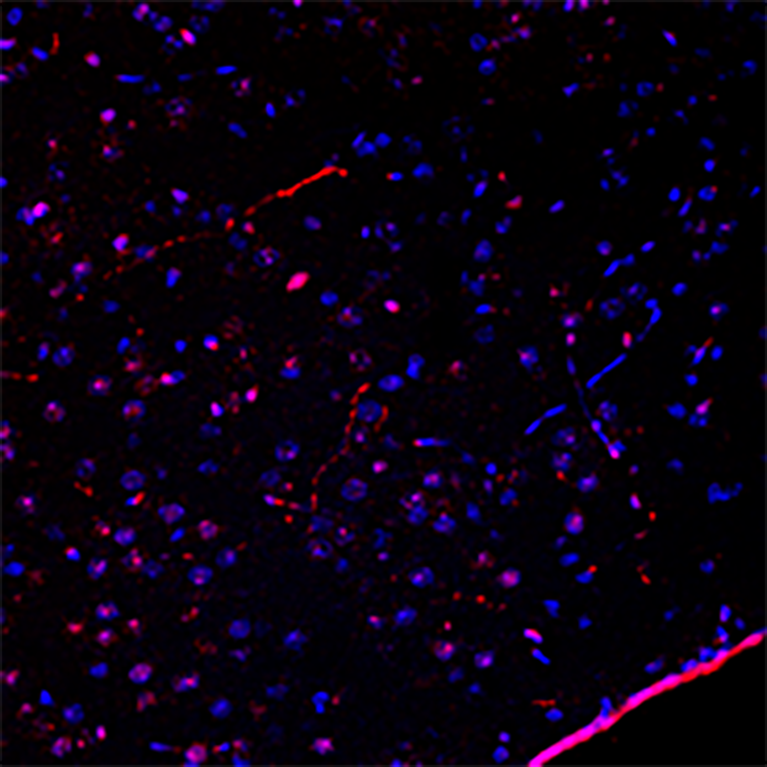

Supplement: Supplementary file 10 — Source data Fig. 8 [file 44319_2025_403_MOESM10_ESM.zip › Figure 8/8A/Orexin A/Tone/overlay 1.tif]

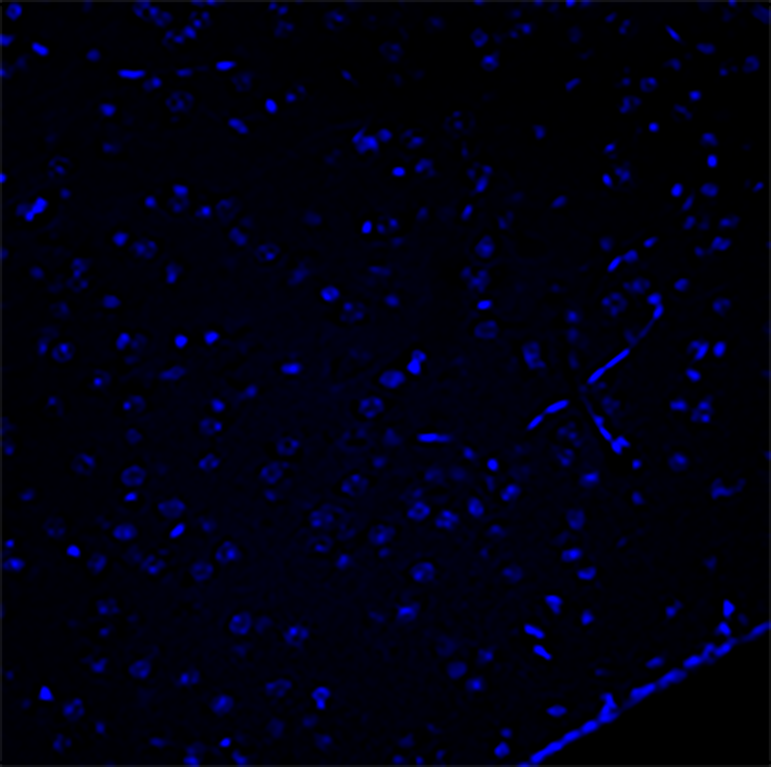

Supplement: Supplementary file 10 — Source data Fig. 8 [file 44319_2025_403_MOESM10_ESM.zip › Figure 8/8A/Orexin A/Tone/Hoechst.tif]

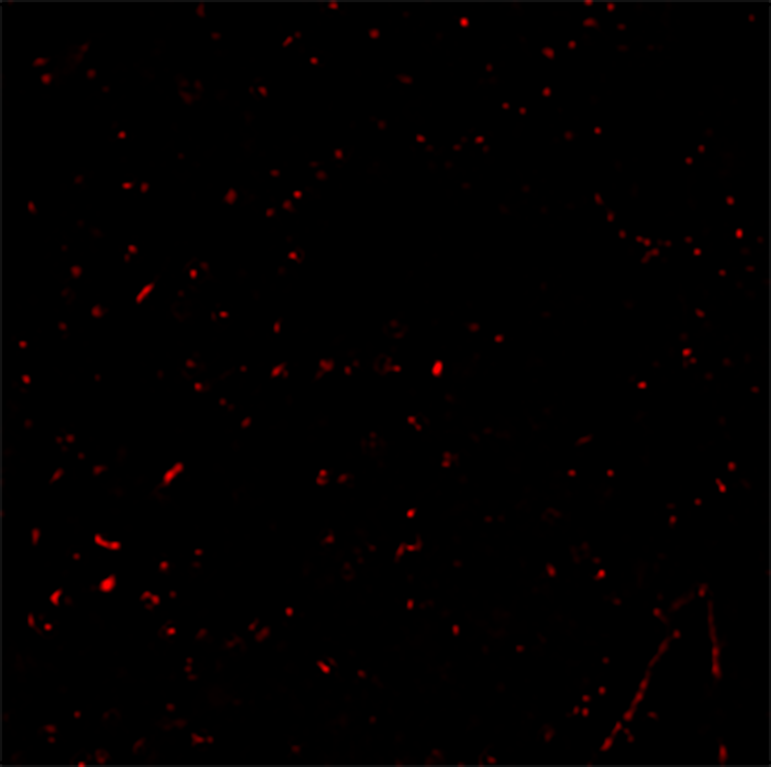

Supplement: Supplementary file 10 — Source data Fig. 8 [file 44319_2025_403_MOESM10_ESM.zip › Figure 8/8A/Orexin A/Control/Orexin A.tif]

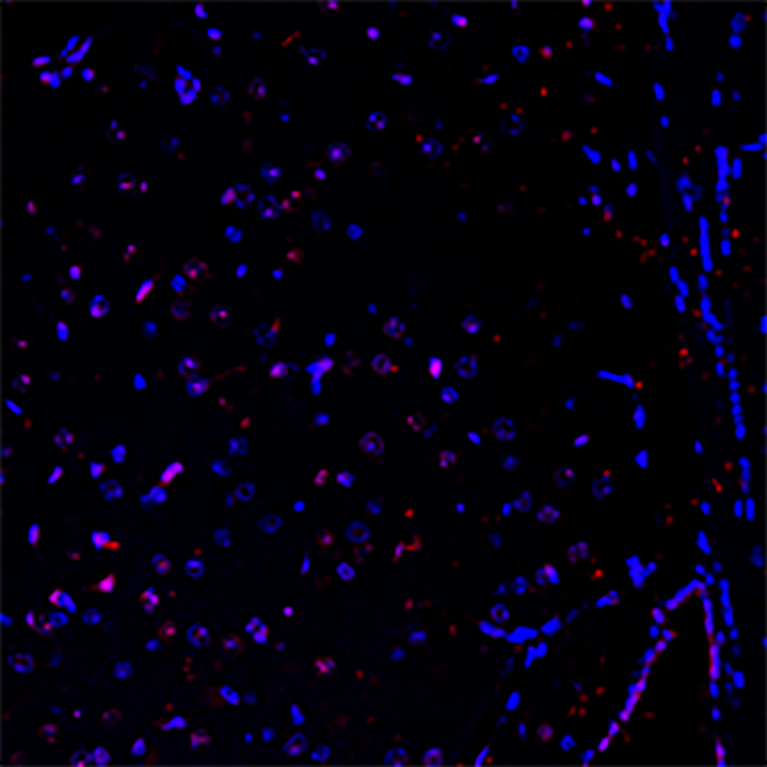

Supplement: Supplementary file 10 — Source data Fig. 8 [file 44319_2025_403_MOESM10_ESM.zip › Figure 8/8A/Orexin A/Control/overlay 1.tif]

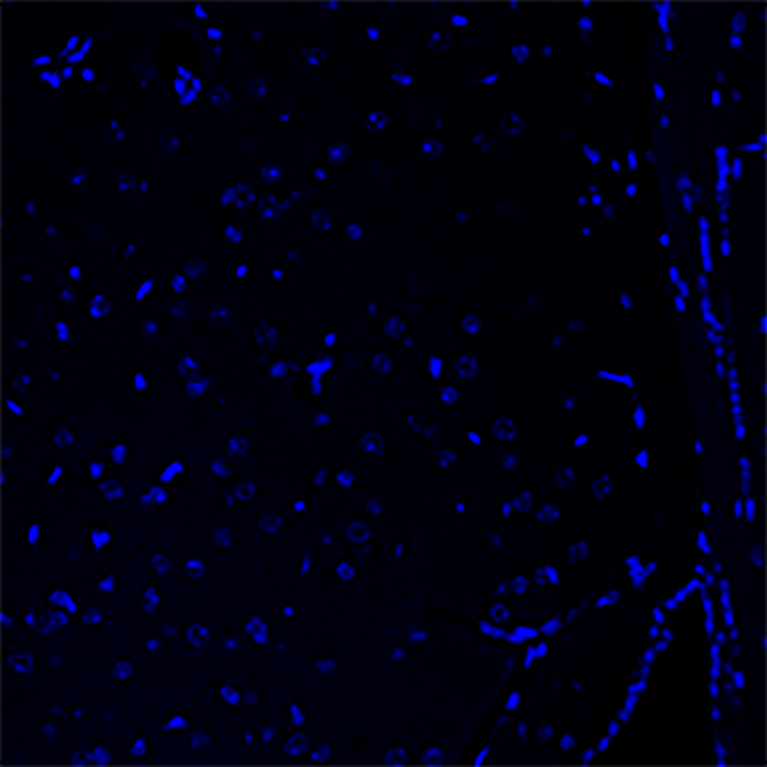

Supplement: Supplementary file 10 — Source data Fig. 8 [file 44319_2025_403_MOESM10_ESM.zip › Figure 8/8A/Orexin A/Control/Hoechst.tif]
