# Supplementary material for: Auditory fear memory retrieval requires BLA-LS and LS-VMH circuitries via GABAergic and dopaminergic neurons
Source: EMBO Rep. 2025 Mar 7;26(7):1816–34. doi: 10.1038/s44319-025-00403-x (PMC11977213; doi:10.1038/s44319-025-00403-x)
Supplement: Supplementary file 1 — Appendix [file 44319_2025_403_MOESM1_ESM.pdf]

Appendix

Table of content

**Items**	**Pages**
Appendix Figure S1	2-3
Appendix Figure S2	4-5
Appendix Figure S3	6
Appendix Figure S4	7-8
Appendix Figure S5	9-10
Appendix Figure S6	11-12
Appendix Figure S7	13-14
Appendix Figure S8	15
Appendix Figure S9	16

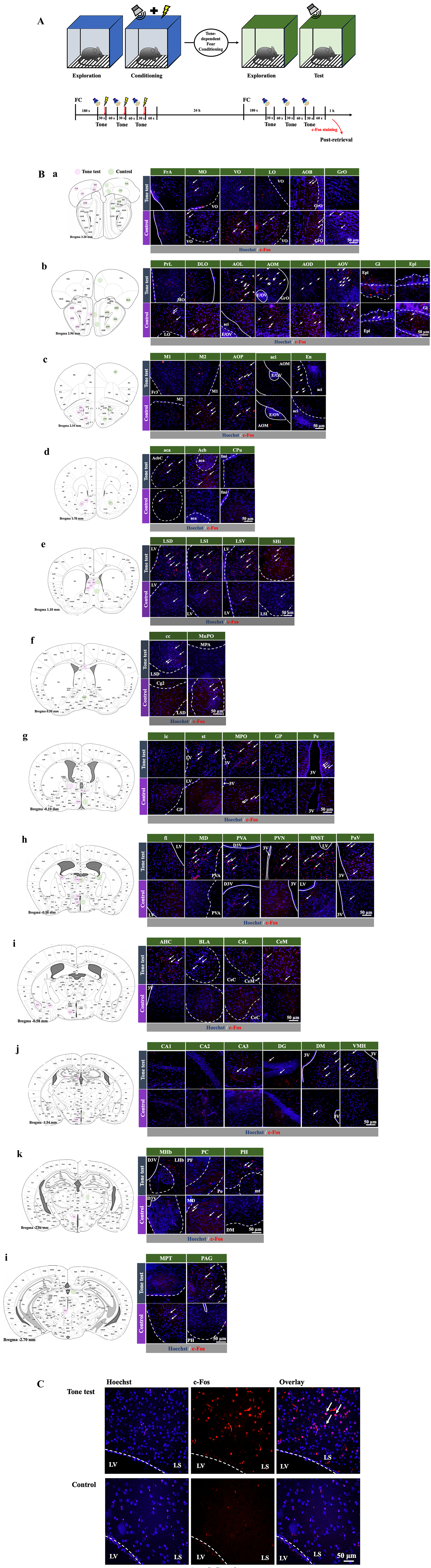

Appendix Figure S1

Appendix Figure S1. Tone stimuli activated various brain regions.

A. A schematic of the experimental design. Mice were sacrificed for the detection of c-Fos positive cells at 1 h after memory retrieval tone stimuli. FC: fear conditioning.

B. Representative images of c-Fos immunohistochemical staining in 54 brain regions (n = 5 mice per group). The pink and green circles in the left panels indicate the brain regions showing in the right panels. The dashed lines in the right panels indicate the boundaries of the brain regions of interest. The white arrows indicate c-Fos positive cells.

C. Representative photomicrographs of c-Fos expression in the LS of mice with tone stimuli and control mice. The dashed lines indicate the boundaries of the LS and LV. The white arrows indicate c-Fos positive cells. LV, lateral ventricle.

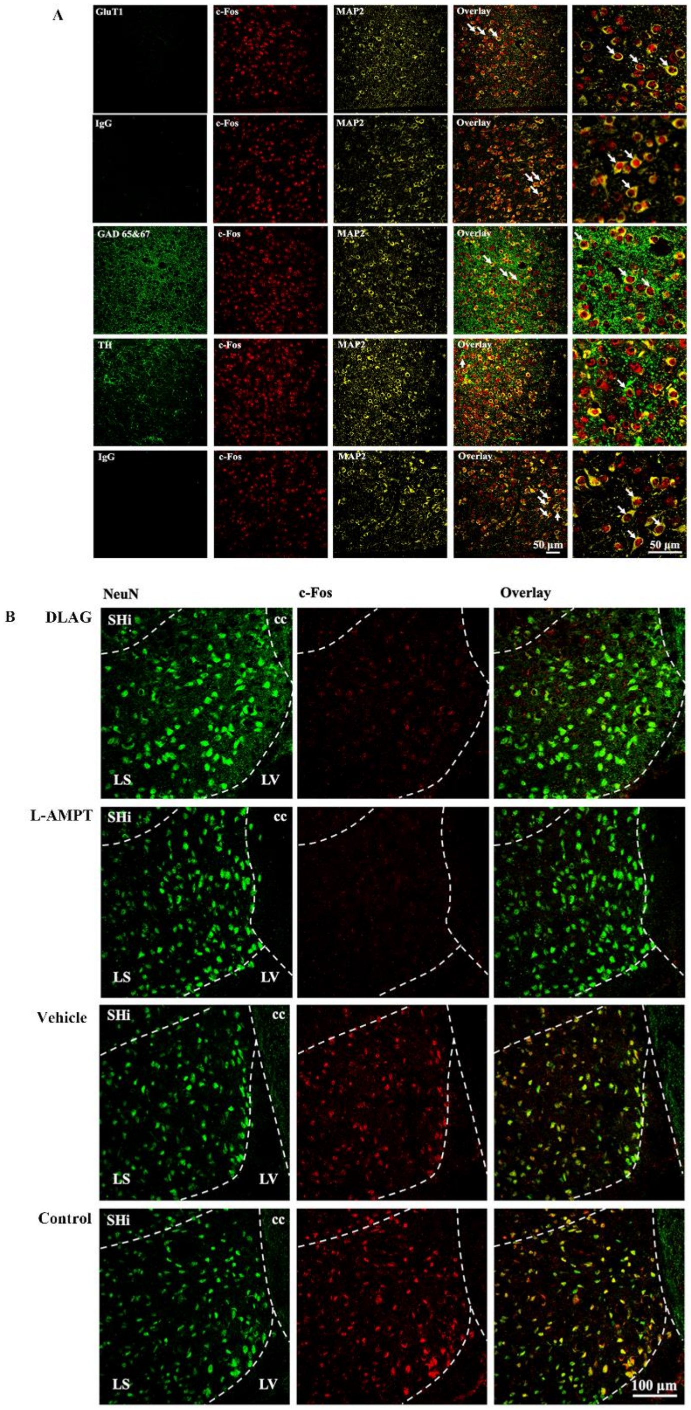

Appendix Figure S2. c-Fos positive neurons in the LS were GABAergic and dopaminergic.

A. Representative images of the LS cells that were labeled by mouse or rabbit serum IgG or an antibody recognizing vesicular GluT1 (vGluT1), GAD 65&67 or TH. The white arrows indicate c-Fos positive cells. vGluT1, vesicular glutamate transporter-1; IgG, immunoglobulin; GAD

65&67, glutamate decarboxylase 65 & glutamate decarboxylase 67; TH, tyrosine hydroxylase; MAP2, Microtubule-associated protein 2.

B. Representative images of immunochemical staining of the LS. The dashed lines indicate the boundary of the LS. SHi, septohippocampal nucleus; cc, corpus callosum; LV, lateral ventricle; DLAG, DL-2-Allylglycine; L-AMPT, α -Methyl-L-tyrosine; NeuN, Neuronal nuclear protein.

Of note, multiple photos in panels A and B are also shown in Figs. 3B and 3D because the experimental conditions for these photos to represent are identical in this figure and Fig. 3.

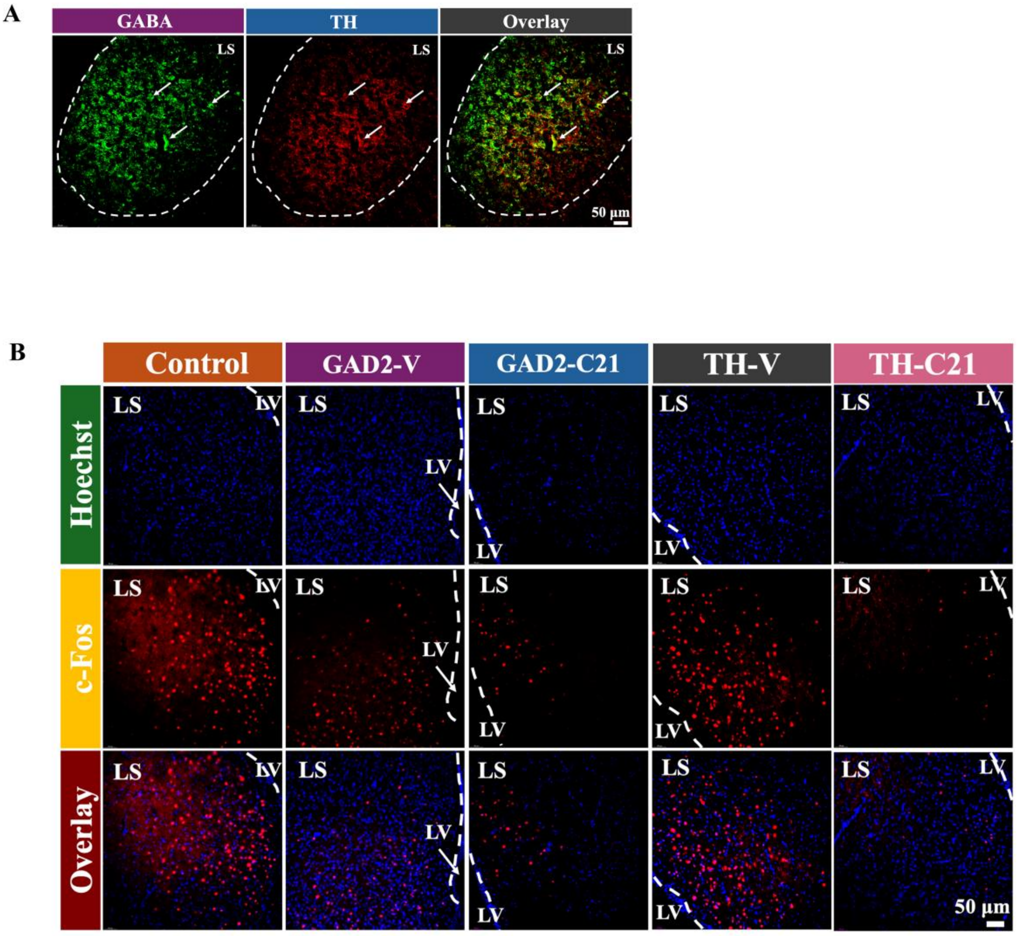

Appendix Figure S3. Co-labeling of GABA and TH in the LS.

A. Representative images of the co-labeling of GABA and TH. The white arrows indicate cells positive for both GABA and TH staining.

B. Representatives of immunofluorescent staining showing c-Fos staining in the LS. C21, compound 21; GAD, glutamate decarboxylase; LV, lateral ventricle; TH, tyrosine hydroxylase; V, vehicle.

Of note, multiple photos in panel B are also shown in Fig. 4E because the experimental conditions for these photos to represent are identical in this figure and Fig. 4.

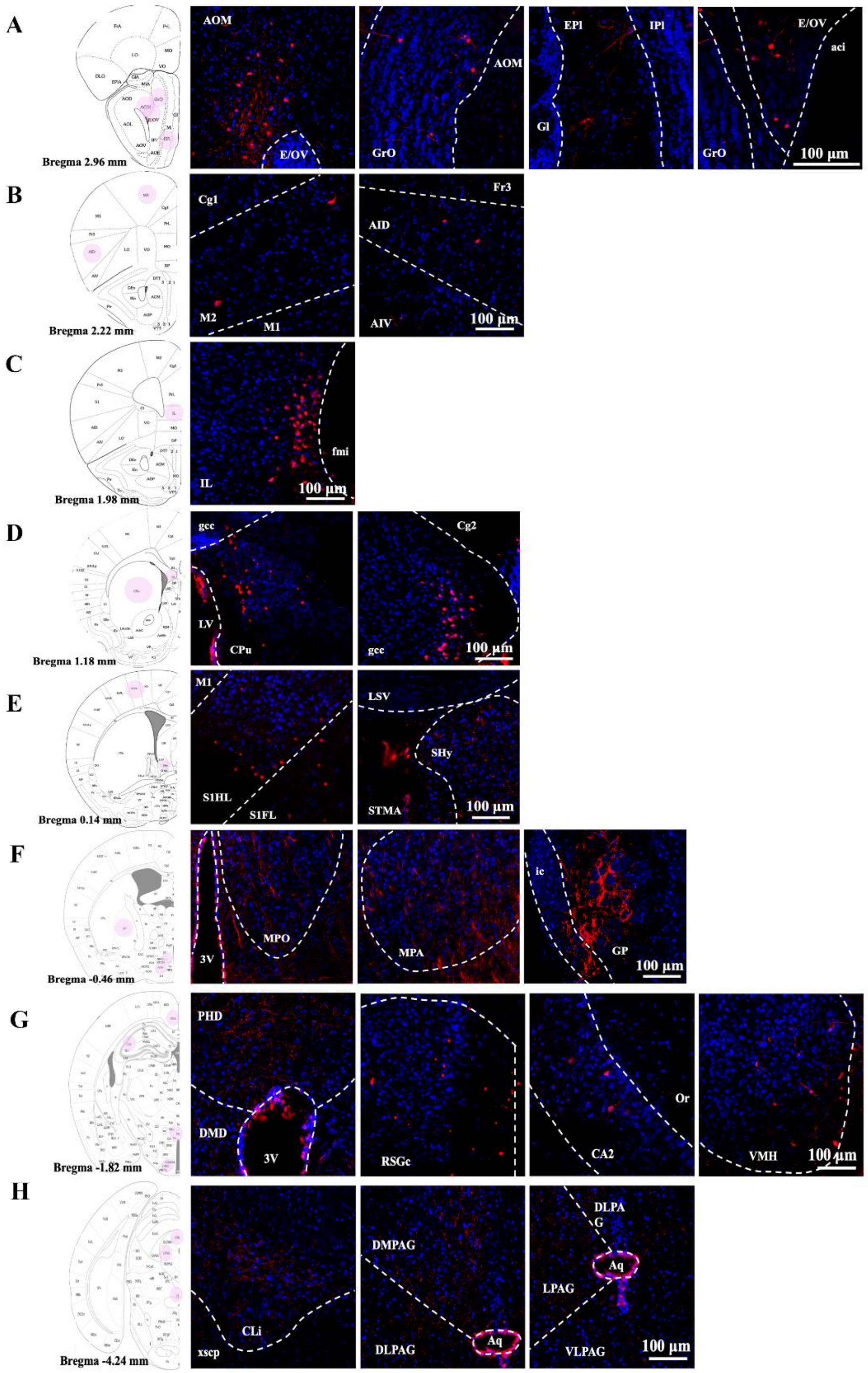
A
Bregma 2.96 mm
AOM
E/OV
GrO
AOM
EPl
IPl
Gl
E/OV
aci
GrO
100 µm
B
Bregma 2.22 mm
Cg1
M2
M1
Fr3
AID
AIV
100 µm
C
Bregma 1.98 mm
fmi
IL
100 µm
D
Bregma 1.18 mm
gcc
LV
CPu
Cg2
gcc
100 µm
E
Bregma 0.14 mm
M1
S1HL
S1FL
LSV
SHy
STMA
100 µm
F
Bregma -0.46 mm
3V
MPO
MPA
ic
GP
100 µm
G
Bregma -1.82 mm
PHD
DMD
3V
RSGc
Or
CA2
VMH
100 µm
H
Bregma -4.24 mm
CLi
xscp
DMPAG
DLPAG
Aq
DLPA
G
Aq
LPAG
VLPAG
100 µm

Appendix Figure S4. Anterograde tracing from the LS.

A to H (from rostral to caudal direction). Representative images of anterograde tracing from the LS in 22 brain regions by using pAAV-CAG-tdTomato (red). Pink circles indicate the brain regions that were projected by LS neurons. The dashed lines indicate the boundaries of brain regions of interest. AID, agranular insular cortex, dorsal part; AOM, anterior olfactory area, medial part; CA2, cornu ammonis region 2 of the hippocampus; CLi, caudal linear nucleus of the raphe; CPu, caudate putamen (striatum); DMPAG, dorsomedial periaqueductal grey; E/OV, ependymal and subendymal layer / olfactory ventricle; EPl, external plexiform layer of the olfactory bulb; gcc, genu corpus callosum; GP, globus pallidus; GrO, granule cell layer of the olfactory bulb; ic, internal capsule; IL, infralimbic cortex; LPAG, lateral periaqueductal grey; M2, secondary motor cortex; MPA, medial preoptic area; MPO, median preoptic nucleus; PHD, post hypothalamic area, dorsal part; RSGc, retrosplenial granular cortex, c; S1HL, primary somatosensory cortex, hindlimb region; SHy, septohypothalamic nucleus; VMH, ventromedial hypothalamic nucleus.

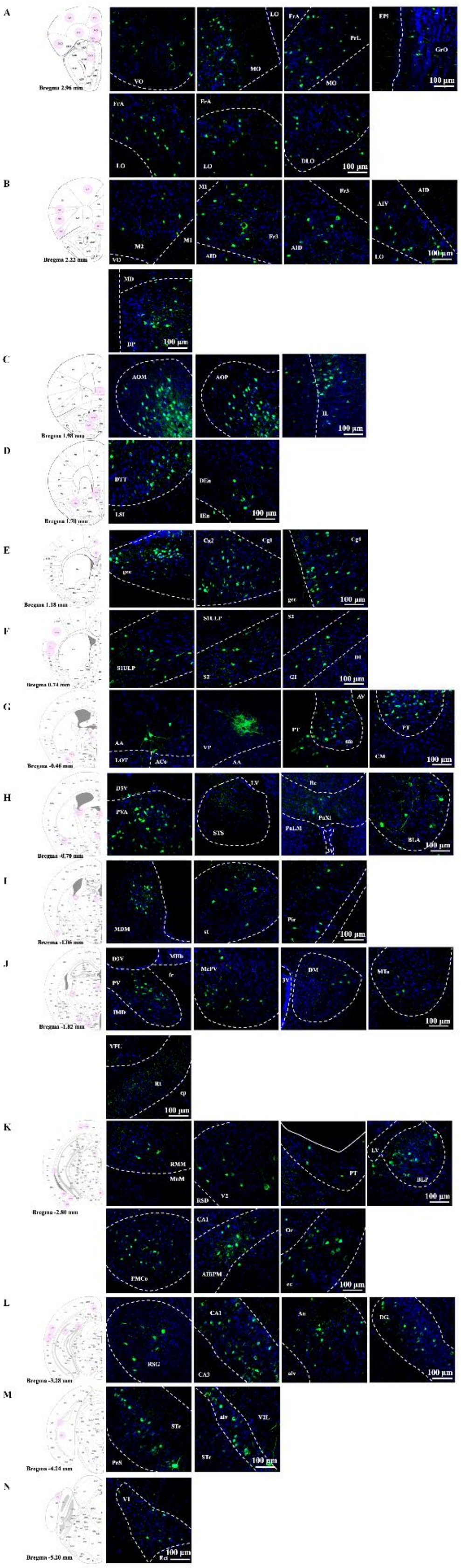

Appendix Figure S5. Retrograde tracing from LS.

A to N (from rostral to caudal direction). Representative images of retrograde tracing from the LS in 53 brain regions by using pAAV-CAG-GFP (green). Pink circles indicate the brain regions that innervate LS neurons. The dashed lines indicate the boundaries of brain regions of interest. AA, anterior amygdaloid area; AHiPM, amygdalohippocampus, posteromedial area; AID, agranular insular cortex, dorsal part; AIV, agranular insular cortex, ventral part; alv, alveus of the hippocampus; AOM, anterior olfactory area, medial part; AOP, anterior olfactory area, posterior part; Au, auditory cortex; BLA, basolateral amygdaloid nucleus; BLP, basolateral amygdala nucleus, post; CA1, cornu ammonis region 1 of the hippocampus; Cg1, cingulate cortex, area 1; Cg2, cingulate cortex, area 2; DEn, dorsal endopiriform claustrum; DG, dentate gyrus; DLO, dorsolateral orbital cortex; DM, dorsomedial hypothalamic nucleus; DP, dorsal peduncular cortex; DTT, dorsal tenia tecta; gcc, genu corpus callosum; ec, external capsule; Fr3, frontal cortex, area 3; FrA, frontal association cortex; GI, granular insular cortex; GrO, granule cell layer of the olfactory bulb; IL, infralimbic cortex; LO, lateral orbital cortex; M2, secondary motor cortex; MDM, mediodorsal thalamic nucleus, medial part; MePV, medial amygdala nucleus, posteroventral part; MO, medial orbital cortex; MTu, medial tuberal nucleus; PaXi, paraxiphoid nucleus of thalamus; PH, posterior hypothalamic nucleus; Pir, piriform cortex; PMCo, posteromedial cortical amygdala nucleus; PrL, prelimbic cortex; PT, paratenial thalamic nucleus; PV, paraventricular thalamic nucleus; PVA, paraventricular thalamic nucleus; RMM, retromammillary nucleus, medial part; RSG, retrosplenial granular cortex; Rt, reticular thalamic nucleus; st, stria terminals; S1ULP, primary somatosensory cortex, upper lip region; S2, secondary somatosensory cortex; sm, stria medullaris; STr, subiculum, transition area; STS, bed nucleus of stria, supracapsular division; V1, primary visual cortex; V2, secondary visual cortex; VO, ventral orbital cortex; VP, ventral pallidum.

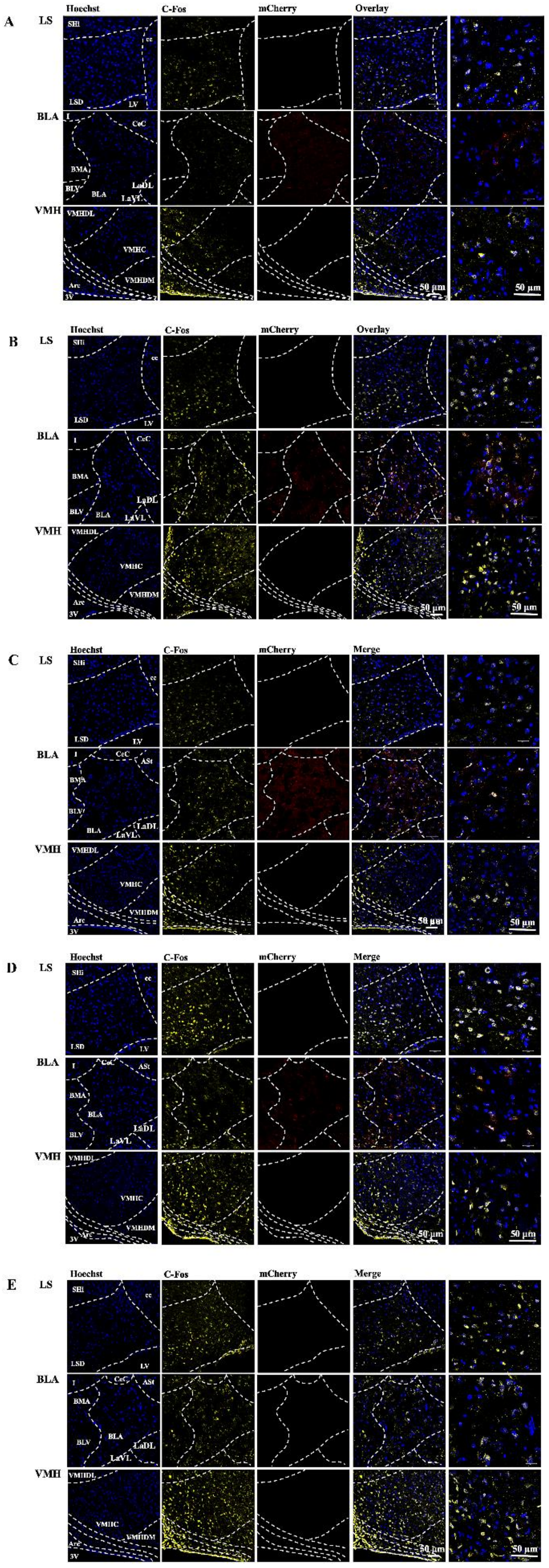
A
B
C
D
E
Hoechst
C-Fos
mCherry
Overlay
Merge
LS
BLA
VMH
SHi
cc
LSD
LV
I
CeC
ASt
BMA
BLV
BLA
LaDL
LaVL
VMHDL
VMHC
VMHDM
Arc
3V
50 μm

Appendix Figure S6. Effects of the inhibition of BLA-LS communication on c-Fos expression.

(A-B) Compound 21 was given before conditioning tone stimuli. **A.** AAV-DIO-hM4Di-mCherry and CAV2-Cre were injected to BLA and LS, respectively. Since mCherry expression was Cre-dependent. Selective mCherry expression in the BLA would indicate that the AAV-DIO-hM4Di-mCherry was successfully expressed in the BLA and the system was good for testing the role of BLA-LS connection in fear conditioning. Right panels are high magnification images of the adjacent panels. The dashed white lines indicate the boundaries of the LS, BLA and VMH. **B.** AAV-DIO-mCherry and CAV2-Cre were injected to the BLA and LS, respectively. Right panels are high magnification images of the adjacent panels. The dashed white lines indicate the boundaries of the LS, BLA and VMH.

(C-D) Compound 21 was given before memory retrieval tone stimuli. **C.** AAV-DIO-hM4Di-mCherry and CAV2-Cre were injected to BLA and LS, respectively. Right panels are high magnification images of the adjacent panels. The dashed white lines indicate the boundaries of the LS, BLA and VMH. **D.** AAV-DIO-mCherry and CAV2-Cre were injected to the BLA and LS, respectively. Right panels are high magnification images of the adjacent panels. The dashed white lines indicate the boundaries of the LS, BLA and VMH.

E. Representatives of immunofluorescence staining showing c-Fos expression in the LS, BLA and VMH of mice without AAV injection. Right panels are high magnification images of the adjacent panels. The dashed white lines indicate the boundaries of the LS, BLA and VMH.

3V, third ventricle; Arc, arcuate hypothalamic nucleus; ASt, amygdalostriatal transition; BMA, basomedial amygdala nucleus, anterior; BLV, basolateral amygdala, ventral; cc, corpus callosum; CeC, central amygdala nucleus, capsular; I, intercalated nuclei amygdala; LaDL, lateral amygdala nucleus, dorsolateral; LaVL, lateral amygdala nucleus, ventrolateral; LV, lateral ventricle; SHi, septohippocampal nucleus.

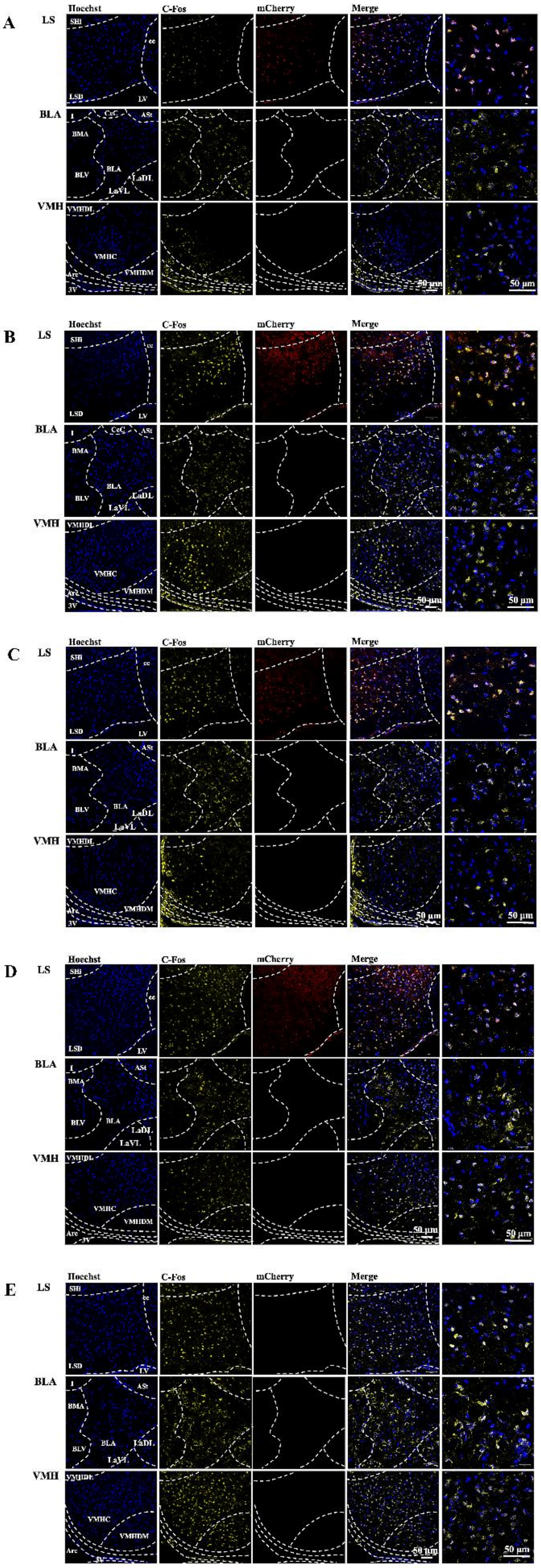
A
B
C
D
E
Hoechst
C-Fos
mCherry
Merge
LS
BLA
VMH
SHi
cc
LSD
LV
I
CeC
ASt
BMA
BLV
BLA
LaDL
LaVL
VMHDL
VMHC
VMHDM
Arc
3V
50 μm

Appendix Figure S7. Effects of the inhibition of LS-VMH communication on c-Fos expression.

(A-B) Compound 21 was given before conditioning tone stimuli. **A.** AAV-DIO-hM4Di-mCherry and CAV2-Cre were injected to the LS and VMH, respectively. Since mCherry expression was Cre-dependent. Selective mCherry expression in the LS would indicate that the AAV-DIO-hM4Di-mCherry was successfully expressed in the LS and the system was good for testing the role of LS-VMH connection in fear conditioning. Right panels are high magnification images of the adjacent panels. Dashed white lines indicate the boundaries of the LS, BLA and VMH.

B. AAV-DIO-mCherry and CAV2-Cre were injected to the LS and VMH, respectively. Right panels are high magnification images of the adjacent panels. Dashed white lines indicate the boundaries of the LS, BLA and VMH.

(C-D) Compound 21 was given before memory retrieval tone stimuli. **C.** AAV-DIO-hM4Di-mCherry and CAV2-Cre were injected to the LS and VMH, respectively. Right panels are high magnification images of the adjacent panels. The dashed white lines indicate the boundaries of the LS, BLA and VMH. **D.** AAV-DIO-mCherry and CAV2-Cre were injected to the LS and VMH, respectively. Right panels are high magnification images of the adjacent panels. The dashed white lines indicate the boundaries of the LS, BLA and VMH.

E. Representatives of immunofluorescence staining showing c-Fos expression in the LS, BLA and VMH of mice without AAV injection. Right panels are high magnification images of the adjacent panels. The dashed white lines indicate the boundaries of the LS, BLA and VMH.

3V, third ventricle; Arc, arcuate hypothalamic nucleus; ASt, amygdalostriatal transition; BMA, basomedial amygdala nucleus, anterior; BLV, basolateral amygdala, ventral; cc, corpus callosum; CeC, central amygdala nucleus, capsular; I, intercalated nuclei amygdala; LaDL, lateral amygdala nucleus, dorsolateral; LaVL, lateral amygdala nucleus, ventrolateral; LV, lateral ventricle; SHi, septohippocampal nucleus.

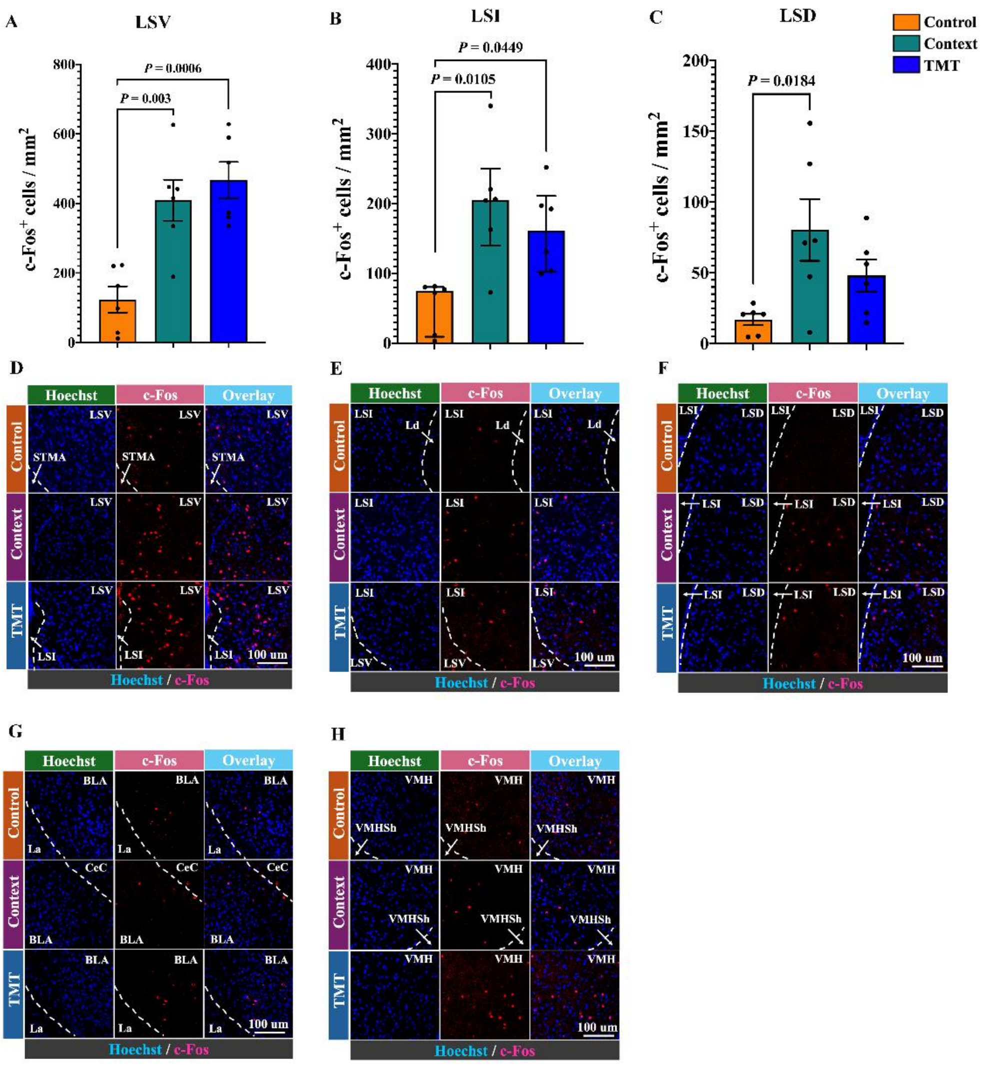

Appendix Figure S8. Fear context or TMT induced activation of LS.

(A-C). Quantification of c-Fos expression in various LS regions. A. Quantification of c-Fos expression in the LSV [n = 6 mice per group; $F_{(2,15)}$ = 13.39, P = 0.0005; one-way analysis of variance test]. B. Quantification of c-Fos expression in the LSI [n = 6 mice per group; H = 9.789 (df = 2) on rank, P = 0.0027; Kruskal-Wallis test]. C. Quantification of c-Fos expression in the LSD [n = 6 mice per group; $F_{(2,15)}$ = 4.849, P = 0.0238; one-way analysis of variance test]. Data are presented as mean ± S.E.M. (normal distribution data) or median ± interquartile range (not normal distribution data) with the presentation of individual animal data in the bar graph.

D-H. Representatives of immunofluorescent staining showing c-Fos staining in the LS, BLA and VMH. CeC, central amygdala nucleus, capsular; La, lateral amygdala nucleus; Ld, lambdoid septal zone; LSD, lateral septal nucleus, dorsal part; LSI, lateral septal nucleus, intermediate part; LSV, lateral septal nucleus, ventral part; LV, lateral ventricle; STMA, bed nucleus of the stria terminalis, medial division, anterior part; VMHSh, ventmedial nucleus hypothalamus shell.

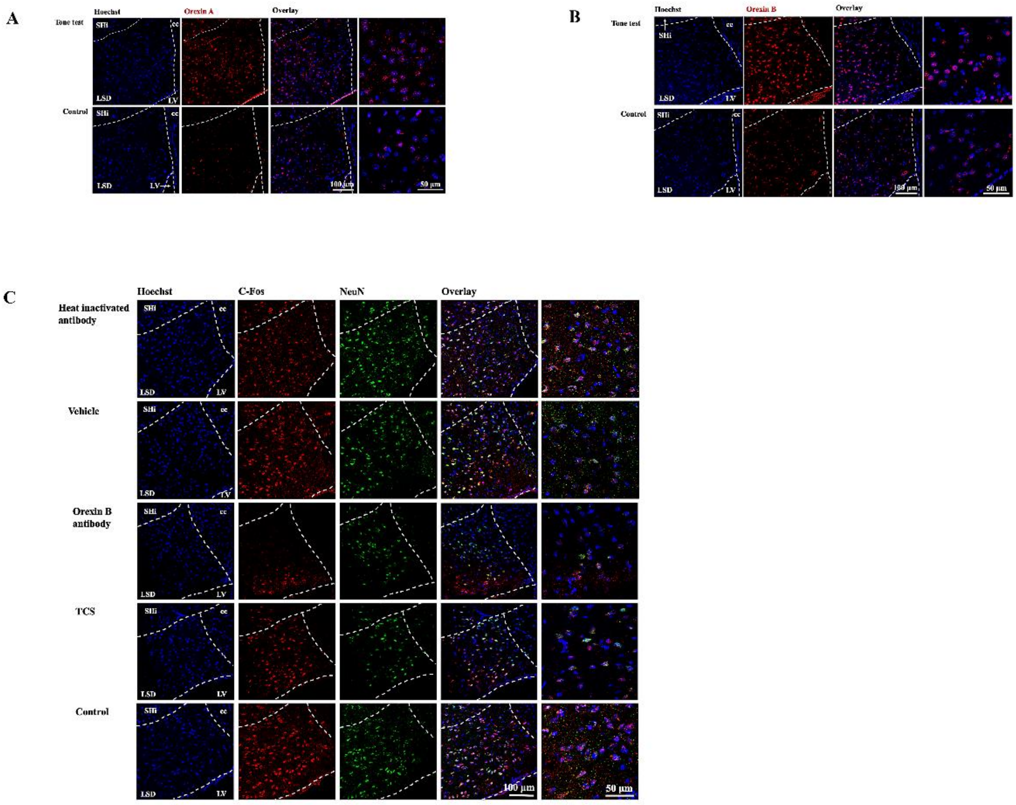

Appendix Figure S9. Effects of tone stimuli on the expression of orexins

A-B Representatives of immunofluorescence staining showing the expression of orexin A and orexin B with or without tone stimuli. Right panels are high magnification images of the adjacent panels. The dashed lines indicate the boundary of the LS. SHi, septohippocampal nucleus; cc, corpus callosum; LV, lateral ventricle.

C. Representative images of LS neurons stained for c-Fos. Right panels are high magnification images of the adjacent panels. The dashed lines indicate the boundaries of the LS. SHi, septohippocampal nucleus; cc, corpus callosum; LV, lateral ventricle; NeuN, Neuronal Nuclei; TCS, orexin B receptor antagonist.
